# Altered erythropoiesis and decreased number of erythrocytes in children with neuroblastoma

**DOI:** 10.18632/oncotarget.18285

**Published:** 2017-05-30

**Authors:** Fabio Morandi, Sebastiano Barco, Sara Stigliani, Michela Croce, Luca Persico, Corrado Lagazio, Francesca Scuderi, Maria Luisa Belli, Mariapina Montera, Giuliana Cangemi, Sarah Pozzi, Valentina Rigo, Paola Scaruffi, Loredana Amoroso, Giovanni Erminio, Vito Pistoia, Silvano Ferrini, Maria Valeria Corrias

**Affiliations:** ^1^ UOC Laboratory of Oncology, IRCCS Istituto Giannina Gaslini, Genoa, Italy; ^2^ UOC Clinical Pathology Laboratory, IRCCS Istituto Giannina Gaslini, Genoa, Italy; ^3^ UOS Physiopathology of Human Reproduction, IRCCS AOU SanMartino-IST, Genoa, Italy; ^4^ UOC Biotherapy, IRCCS AOU SanMartino-IST, Genoa, Italy; ^5^ Department of Economy, University of Genoa, Genoa, Italy; ^6^ UOC Oncology, IRCCS Istituto Giannina Gaslini, Genoa, Italy; ^7^ UOC Immune-Hematology and Transfusion Medicine, IRCCS Istituto Giannina Gaslini, Genoa, Italy; ^8^ UOC Immune-Hematology and Transfusion Medicine, IRCCS AOU SanMartino-IST, Genoa, Italy; ^9^ UOS Epidemiology, Biostatistics and Committees, IRCCS Istituto Giannina Gaslini, Genoa, Italy; ^10^ Immunology Area, IRCCS Bambino Gesù, Rome, Italy

**Keywords:** neuroblastoma, erythrocytes, bone marrow, survival, microenvironment

## Abstract

Neuroblastoma (NB) is a pediatric tumor presenting at diagnosis either as localized or metastatic disease, which mainly involves the bone marrow (BM). The physical occupancy of BM space by metastatic NB cells has been held responsible for impairment of BM function. Here, we investigated whether localized or metastatic NB may alter hematopoietic lineages’ maturation and release of mature cells in the periphery, through gene expression profiling, analysis of BM smears, cell blood count and flow cytometry analysis.

Gene ontology and disease-associated analysis of the genes significantly under-expressed in BM resident cells from children with localized and metastatic NB, as compared to healthy children, indicated anemia, blood group antigens, and heme and porphyrin biosynthesis as major functional annotation clusters. Accordingly, in children with NB there was a selective impairment of erythrocyte maturation at the ortho-chromic stage that resulted in reduced erythrocyte count in the periphery, regardless of the presence of metastatic cells in the BM. By considering all NB patients, low erythrocyte count at diagnosis associated with worse survival. Moreover, in the subset of metastatic patients, low erythrocyte count, hemoglobin and hematocrit and high red cell distribution width at follow-up also associated with worse outcome.

These observations provide an alternative model to the tenet that infiltrating cells inhibit BM functions due to physical occupancy of space and may open a new area of research in NB to understand the mechanism(s) responsible for such selective impairment.

## INTRODUCTION

Neuroblastoma (NB) is a pediatric tumor, whose biology and clinical behavior continuously puzzles researchers and clinicians. NB displays a broad spectrum of clinical features that vary from tumors that spontaneously regress [1] to metastatic disease at onset [2]. A genetic alteration, namely *MYCN* amplification, was demonstrated to control NB aggressiveness [3–5]. However, the number of driving mutations, essential to initiate and/or sustain tumor growth, has been identified only in a fraction of NB [6, 7]. NB was the first solid tumor found to selectively express a surface tumor-specific antigen, the GD2 di-sialoganglioside [8, 9], allowing a “precision medicine approach” for specific immunotherapy [10], now included in standard therapeutic regimen for metastatic NB patients [2, 11]. Notwithstanding, the survival rate of NB patients treated with anti-GD2 antibodies increased only slightly [2].

The deep knowledge of NB tumor genetics and biology have allowed to establish effective treatments for some of NB patients, such as infants and children with localized, *MYCN* not amplified tumors, with 5-year survival rates between 90 and 100%, depending on age and stage [12]. Unfortunately, prognosis of children with either localized *MYCN* amplified tumors and of children with metastatic disease (high-risk patients) is still poor with 5-year survival rates around 65 and 30%, respectively [11, 13, 14]. High-risk NB patients that do not respond to standard chemotherapy or relapse have less than 10% 5-year survival rate [15, 16].

Metastatic patients present massive bone marrow (BM) infiltration by NB cells that were considered identical to the primary tumor cells. However, in the last decade, it has become evident that metastatic cells were not exactly the same as the correspondent primary tumor cells [17, 18]. In addition, many studies demonstrated that the properties of both neoplastic and resident cells were deeply modified by the bi-directional signals occurring within the local microenvironment [19].

Based on these premises, we previously studied the gene expression profiles of BM-infiltrating NB cells as compared to NB primary tumor cells [20]. BM-infiltrating NB cells shared the same neuronal characteristics of primary tumor cells, but acquired expression of surface proteins typical of the hematopoietic cell lineages. BM-infiltrating NB cells showed also different microRNA profile [21], and different rates of potential driving mutations and chromosomal rearrangements [22, 23]. Altogether, these data suggested that the BM environment could modify NB cell features.

As for the resident BM cells, we previously showed that in NB patients their gene expression profile was different from that of healthy children, regardless of the presence of BM-infiltrating NB cells. These data suggested a remote effect operated by the primary tumor cells on the BM resident cells [24]. In particular, the genes over-expressed in NB patients belonged to the *IFN* and *IFRD* signatures, suggestive of innate immunity activation, whereas the most under-expressed gene was *CXCL12 (SDF-1)* [24].

To better characterize the BM microenvironment of NB patients, we here investigated whether the genes under-expressed in BM resident cells belonged to particular functional pathway(s) and/or signature(s) which may relate to impairment of BM function.

## RESULTS

### Gene expression profiles of BM resident cells from NB patients and healthy children

The gene expression profile of BM resident cells from 44 NB patients, whose main features are shown in Table 1, were compared to those of BM resident cells from 13 healthy children (http://www.ncbi.nlm.nih.gov/geo/, GEO accession number GSE90689). By setting the statistical significance for differential expression at 0.01 Bonferroni's adjusted P value, samples from patients with metastatic and localized NB showed to under-express 641 and 1239 genes, respectively (Supplementary Table 1A and 1B), as compared to BM resident cells from healthy children. Twenty-nine genes were found to be differentially expressed between metastatic and localized NB, but statistical significance was not reached (Supplementary Table 1C), confirming that the gene expression profiles of BM resident cells from metastatic and localized NB patients were similar [24]. Thus, by considering all NB patients in our cohort, the genes significantly under-expressed were 1085, of whom 533 in common between metastatic and localized patients (Supplementary Table 1D and 1E, respectively).

**Table 1:** Main features of the cohorts of NB patients analyzed for gene expression profiling, BM smears, flow cytometry and cell blood count (CBC)

	NB patients							
	Gene expression	%	BM smear	%	Flowcytometry	%	CBC	%
**N**	**44**		**70**		**16**		**115**	
**Sex**								
Female	17	38.6	33	47.1	9	56.2	51	44.3
Male	27	61.4	37	52.9	7	43.8	64	55.7
**Age**								
< 18 months	24	54.5	33	47.1	10	62.5	55	47.8
≥18 months	20	45.5	37	52.9	6	37.5	60	52.2
**Stage**								
L1/L2	34	77.3	20	28.6	10	62.5	64	55.7
M	10	22.7	37	52.8	6	37.5	38	33.0
Ms			13	18.6			13	11.3
**MYCN**								
Amplified	6	13.6	22	31.4	4	25.0	28	24.4
Single copy	35	79.6	43	61.5	12	75.0	72	62.6
ND	3	6.8	5	7.1	0		15	13.0

### Gene ontology analysis

To check whether the under-expressed genes clustered for annotation or associated to a specific disease, the list of 533 genes under-expressed in both metastatic and localized NB (Supplementary Table 1E) was run into the DAVID bioinformatics database. In addition to functional annotation clusters containing more than one-hundred genes, such as phosphoprotein, acetylation, cytosol and cytoplasm, the most significant functional annotation clusters referred to genes involved in anemia, blood group antigens, heme and porphyrin biosynthesis (Table 2). Moreover, 63 of the 533 under-expressed genes associated to diseases involving erythrocytes (Table 3). Altogether these data suggested possible alterations of erythropoiesis in NB patients.

**Table 2:** Functional annotation chart with adjusted P value < 0.01 obtained by running the list of genes significantly down-modulated in BM resident cells from patients with metastatic and localized NB as compared to healthy children

Category	Term	Number of genes	%	P value	Benjamini P value
UP_KEYWORDS	Hereditary hemolytic anemia	16	4,2	5,50E-18	9,80E-16
UP_KEYWORDS	Blood group antigen	16	4,2	3,00E-18	1,10E-15
BIOCARTA	Hemoglobin's Chaperone	10	2,6	1,30E-11	1,60E-09
UP_KEYWORDS	Phosphoprotein	217	56,5	1,70E-11	2,00E-09
UP_KEYWORDS	Heme biosynthesis	9	2,3	3,40E-11	3,00E-09
UP_KEYWORDS	Porphyrin biosynthesis	7	1,8	1,00E-09	7,30E-08
GOTERM_BP_DIRECT	heme biosynthetic process	10	2,6	1,40E-10	2,50E-07
UP_KEYWORDS	Acetylation	109	28,4	4,80E-09	2,90E-07
GOTERM_CC_DIRECT	cytosol	115	29,9	1,60E-09	5,60E-07
GOTERM_BP_DIRECT	protoporphyrinogen IX biosynthetic process	7	1,8	5,80E-09	5,10E-06
GOTERM_BP_DIRECT	porphyrin-containing compound biosynthetic process	6	1,6	7,20E-08	4,30E-05
UP_KEYWORDS	Elliptocytosis	5	1,3	7,50E-06	3,80E-04
KEGG_PATHWAY	Porphyrin and chlorophyll metabolism	9	2,3	7,50E-06	1,60E-03
GOTERM_BP_DIRECT	response to methylmercury	6	1,6	4,00E-06	1,80E-03
UP_KEYWORDS	Oxygen transport	5	1,3	7,10E-05	2,80E-03
UP_KEYWORDS	Disease mutation	74	19,3	6,80E-05	3,00E-03
UP_KEYWORDS	Cytoplasm	121	31,5	1,10E-04	3,80E-03
UP_KEYWORDS	Cell cycle	27	7	1,80E-04	5,80E-03
UP_KEYWORDS	Transferase	52	13,5	2,30E-04	6,90E-03
UP_KEYWORDS	Iron	17	4,4	3,60E-04	9,90E-03

**Table 3:** Results of analysis for disease of hematopoietic lineages associated to genes significantly under-expressed in BM resident cells from patients with metastatic and localized NB as compared to healthy children

OMIM_DISEASE	Gene name
Acatalasemia,	CAT
Anemia, hypochromic microcytic, with iron overload 2,	STEAP3
Anemia, sideroblastic, pyridoxine-refractory, autosomal recessive,	GLRX5
Anemia, sideroblastic, pyridoxine-refractory, autosomal recessive,	SLC25A38
Anemia, sideroblastic, X-linked, Protoporphyria, erythropoietic, X-linked,	ALAS2
Bleeding disorder, platelet-type, 17,	GFI1B
Blood group GIL,	AQP3
Blood group, Colton, Aquaporin-1 deficiency,	AQP1
Blood group, Diego, Blood group, Waldner, Blood group, Wright, Ovalocytosis, SA type, Renal tubular acidosis, distal, AD, Cryohydrocytosis, Blood group, Swann, Blood group, Froese, Malaria, resistance to, Renal tubular acidosis, distal, AR, Spherocytosis, type 4,	SLC4A1
Blood group, Dombrock,	ART4
Blood group, Kell,	KEL
Blood group, Kidd,	SLC14A1
Blood group, Landsteiner-Wiener,	ICAM4
Blood group, Langereis system, Pseudohyperkalemia, familial, 2, due to red cell leak, Microphthalmia, isolated, with coloboma 7, Dyschromatosisuniversalishereditaria 3,	ABCB6
Blood group, OK,	BSG
Blood group, Radin, Blood group, Scianna system,	ERMAP
Blood group, Rhesus, Rh-null disease, amorph type,	RHCE
Blood group--Lutheran inhibitor, Hereditary persistence of fetal hemoglobin, Dyserythropoietic anemia, congenital, type IV,	KLF1
Bombay phenotype,	FUT1
C4B deficiency,	C4B
Coproporphyria, Harderoporphyria,	CPOX
D-2-hydroxyglutaric aciduria 2,	IDH2
Elliptocytosis-2, Pyropoikilocytosis, Spherocytosis, type 3,	SPTA1
Erythrocytosis due to bisphosphoglyceratemutase deficiency,	BPGM
Erythrocytosis, familial, 1,	EPOR
Filippi syndrome,	CKAP2L
Glutaricaciduria, type I,	GCDH
Glyoxalase II deficiency,	HAGH
Heinz body anemia, Thalassemia, alpha-, Hemoglobin H disease, nondeletional, Erythrocytosis, Hypochromic microcytic anemia,	HBA2
Heinz body anemias, alpha-, Thalassemias, alpha-, Hemoglobin H disease, nondeletional, Erythremias, alpha-, Methemoglobinemias, alpha-,	HBA1
Hemolytic anemia due to adenylate kinase deficiency,	AK1
Hemolytic anemia due to gamma-glutamylcysteinesynthetase deficiency, Myocardial infarction, susceptibility to,	GCLC
Hermansky-Pudlak syndrome 6,	HPS6
Lead poisoning, susceptibility to, Porphyria, acute hepatic,	ALAD
Leukemia, acute pre-B-cell,	PBX1
Leukemia, megakaryoblastic, with or without Down syndrome, somatic, Thrombocytopenia, X-linked, with or without dyserythropoietic anemia, Anemia, X-linked, with/without neutropenia and/or platelet abnormalities, Thrombocytopenia with beta-thalassemia, X-linked,	GATA1
Leukemia, T-cell acute lymphocytic, somatic,	TAL1
Majeed syndrome,	LPIN2
Malaria, resistance to, Blood group, Gerbich,	GYPC
Malaria, resistance to, Blood group, MN,	GYPA
Malaria, resistance to, Blood group, Ss,	GYPB
McLeod syndrome with or without chronic granulomatous disease,	XK
Mitochondrial complex I deficiency,	NDUFS2
Mitochondrial complex III deficiency, nuclear type 6,	CYC1
Myelodysplastic syndrome, Myelogenous leukemia, acute,	ACSL6
Overhydrated hereditary stomatocytosis, Anemia, hemolytic, Rh-null, regulator type, Rh-mod syndrome,	RHAG
Platelet glycoprotein IV deficiency, Coronary heart disease, susceptibility to, 7, Malaria, cerebral, reduced risk of, Malaria, cerebral, susceptibility to, Macrothrombocytopenia,	CD36
Porphyria cutanea tarda, Porphyria, hepatoerythropoietic,	UROD
Porphyria variegata,	PPOX
Porphyria, acute intermittent, Porphyria, acute intermittent, nonerythroid variant,	HMBS
Porphyria, congenital erythropoietic,	UROS
Protoporphyria, erythropoietic, autosomal recessive,	FECH
Pyruvate carboxylase deficiency,	PC
Ribose 5-phosphate isomerase deficiency,	RPIA
Senior-Loken syndrome 9,	TRAF3IP1
Spherocytosis, type 1,	ANK1
Spherocytosis, type 2, Anemia, neonatal hemolytic, fatal and near-fatal, Elliptocytosis-3,	SPTB
Spherocytosis, type 5,	EPB42
Succinic semialdehyde dehydrogenase deficiency,	ALDH5A1
Symmetric circumferential skin creases, congenital, 1, Cortical dysplasia, complex, with other brain malformations 6,	TUBB
Thalassemia due to Hb Lepore, Thalassemia, delta-,	HBD
Uric acid concentration, serum, QTL1, Junior blood group system,	ABCG2
Warfarin resistance, Vitamin K-dependent clotting factors, combined deficiency of, 2,	VKORC1

Since a cluster of 16 genes belonged to blood group antigens expressed on mature erythrocytes, we checked in a cohort of 115 NB patients (Table 1, CBC) whether the distribution into the ABO system and the frequency of Rh D antigen was in accordance with that of the European population [25, 26]. As shown in Supplementary Figure 1, no difference was observed, thus excluding any type of skewing for NB susceptibility depending on blood group antigens.

### Erythrocyte precursors and erythroblasts are altered in BM samples from NB patients

**Figure 1:**
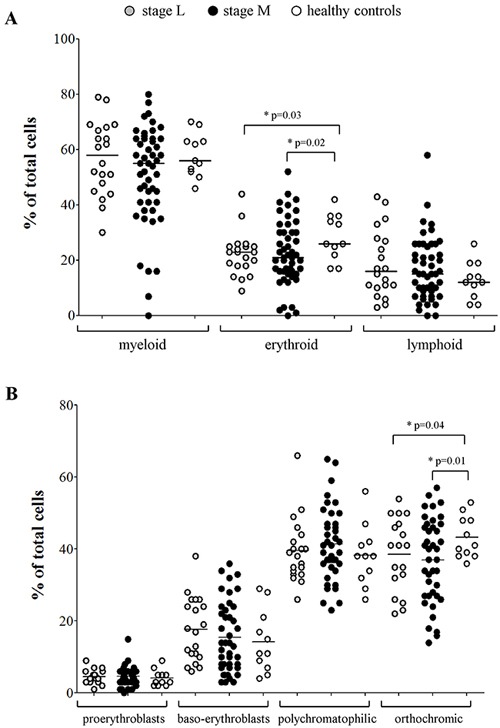
**(A)** Percentages of myeloid, erythroid and lymphoid cell populations present in BM smears, **(B)** percentages of pro-erythroblasts, baso-erythroblasts, polychromatophilic and orthocromic erythroblasts present in BM smears from NB patients with stage L (grey dots, N=20) and stages M and Ms (black dots, N=50) and healthy children (open dots, N=11).

Since the expression profiles of BM resident cells from NB patients suggested a possible alteration in erythrocyte count, we next scored BM smears from 70 NB patients with localized (L, N=20) or metastatic (M, N=50) NB (Table 1, Smears) for total myeloid-, erythroid- and lymphoid-precursors, and compared them to BM smears from 11 healthy children. As shown in Figure 1A, healthy children showed higher proportions of erythrocyte precursors (median±SEM % of total cells: 26±2.48) than patients with localized (23±1.75, p=0.02) or metastatic (21±1.68, p=0.03) NB. Conversely, no difference was detected in count of myeloid (healthy: 56±2.3; L NB: 58±2.9; M NB: 55±2.5) and lymphoid (healthy: 12±2; L NB: 16±2.7; M NB: 15±1.6) precursors.

The same BM smears were then scored for presence of erythroid precursors. No significant difference was detected in the percentage of pro-erythroblasts (mean±SD % of total cells: healthy: 4.1±2.2; L NB: 4.5±1.8; M NB: 4.6±2.5), baso-erythroblasts (healthy: 14.2±8.6; L NB: 17.7±8.8; M NB: 15.4±9.7) and poly-chromatophilic erythroblasts (healthy: 38.2±8.4; L NB: 39.5±8.8; M NB: 41.4±10), whereas the percentage of ortho-chromic erythroblasts was significantly higher in healthy children (43.2±5.8) than in patients with localized (38.6±10.1, p=0.01) and metastatic (36.9±11.7, p=0.04) NB (Figure 1B).

### Maturation of erythrocytes is altered in BM from NB patients

**Figure 2:**
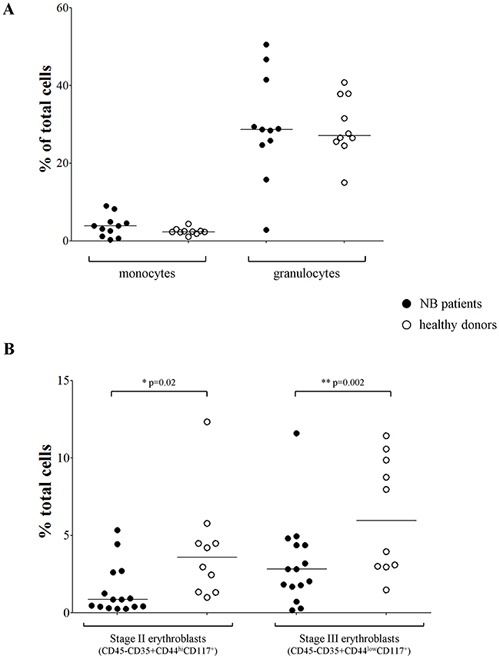
**(A)** Percentages of monocytes and granulocytes, **(B)** percentages of stage II and III erythroblasts present in BM samples from NB patients (closed dots, N=11 for panel A and N=15 for panel B) and healthy subjects (open dots, N=10).

To confirm the data obtained in BM smears suggestive of a selective impairment of late stage erythrocyte maturation, we collected BM aspirates from 16 NB patients (Table 1, flow cytometry) and 10 healthy subjects and analyzed them by flow cytometry for percentage of monocytes, neutrophils and stage II/III erythroblasts. As shown in Figure 2A, the monocyte and granulocyte counts were similar between NB patients (mean±SD % of total cells: monocytes 3.86±2.82; granulocytes 29.38±13.49) and healthy subjects (monocytes 2.46±0.83; granulocytes 29.4±7.76). Conversely, the percentage of stage II (CD45^−^CD35^+^CD44^hi^CD117^+^) and stage III (CD45^−^CD35^+^CD44^low^CD117^+^) erythroblasts was significantly lower in NB patients (median±SEM % of total cells, stage II: 2.82±0.72; stage III: 0.85±0.41) than in healthy subjects (stage II: 5.96±1.18;stage III: 3.58±1.05; p=0.02 and p=0.002, respectively) (Figure 2B), confirming that late erythrocytes’ maturation was indeed altered in NB patients.

### Peripheral blood (PB) samples from NB patients displays several abnormalities in different cell populations

**Figure 3:**
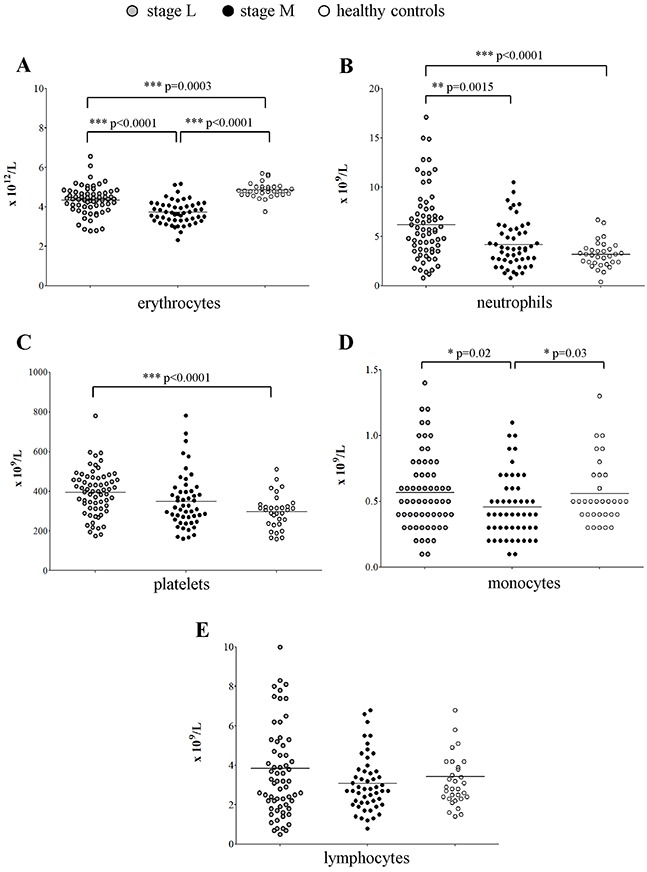
Peripheral cell blood counts obtained from patients with stage L (gray dots, N=64), stage M and Ms (black dots, N=51) and healthy children (open dots, N=32) **(A)** erythrocytes, **(B)** neutrophils, **(C)** platelets, **(D)** monocytes and **(E)** lymphocytes.

To check whether the altered count of BM erythrocyte precursors impacted on the mature cell population present in the periphery, we collected cell blood counts (CBCs) at diagnosis from a cohort of 115 patients with localized and metastatic NB (Table 1, CBC) and compared them to CBCs from 32 age- and sex-matched healthy children. In general, all the red blood cell and leukocyte counts were within the lower limit of the normal range. However, healthy children had significantly higher amount of mature erythrocytes (mean±SD x10^12^cell/L: 4.85±0.38) than children with localized (4.34±0.71; p=0.0003) or metastatic (mean±SD:3.72±0.58; p<0.0001) NB (Figure 3A), confirming the impairment observed in the BM. As for the other mature hematopoietic cell populations, patients with localized NB had significantly higher proportion of neutrophils (median±SEM x10^9^cell/L: 5.7±0.45) than healthy children (3.1±0.23; p<0.0001) or patients with metastatic NB (3.8±0.32; p=0.0015) (Figure 3B). Similarly, patients with localized NB had higher platelet count (mean±SD x10^9^cell/L: 395±114.3) than healthy children (297.8±85.25, p<0.0001) or patients with metastatic NB (349.3±135.2) (Figure 3C). In contrast, the monocyte count was lower in metastatic patients (median±SEM x10^9^cell/L: 0.4±0.03) than in localized patients (0.5±0.03, p=0.02) or healthy children (0.5±0.04, p=0.03) (Figure 3D).

No difference in lymphocyte count (Figure 3E) was observed among patients with localized NB (median±SEMx10^9^cell/L: 3.2±0.31), metastatic NB (2.8±0.19) and healthy children (2.85±0.33).

### Monocyte, neutrophil and erythrocyte count at diagnosis associated with clinical outcome of NB patients

**Figure 4:**
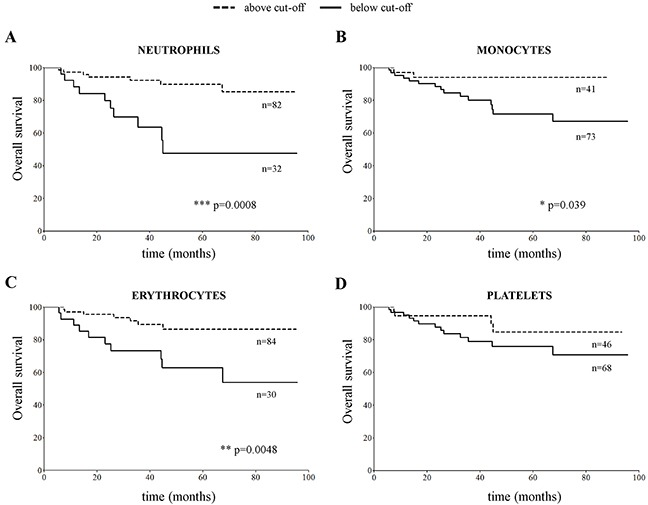
Overall survival curves obtained by stratifying the CBC cohort of NB patients (N=115) with number of **(A)** neutrophils, **(B)** monocytes, **(C)** erythrocytes and **(D)** platelets in PB samples above and below the cut-off value determined for each cell population by ROC curves (5.7×10^9^/L for neutrophils, 0.4×10^9^/L for monocytes, 3.3×10^12^/L for erythrocytes and 400×10^9^/L for platelets).

Taken together the above findings showed that at diagnosis all NB patients had decreased erythrocyte and increased platelet count, as compared to healthy children. Differently from children with metastatic NB, those with localized NB showed a significant increase in neutrophil and a normal monocytes’ count. We thus checked whether the number of mature neutrophils, monocytes, erythrocytes and platelets associated to outcome. NB patients with number of neutrophil, monocyte and erythrocyte counts above the cut-off values determined by ROC curves displayed a significantly better overall survival (OS) than those with counts below the cut-off values (Figure 4A, 4B and 4C, p=0.0008, p=0.039 and p=0.0048, respectively). In contrast, the number of platelets at diagnosis (Figure 4D) did not associate with different OS.

### The outcome of patients with metastatic NB associated with the number of erythrocytes at follow-up

Since prognosis of patients with metastatic NB is poor despite multimodal therapies, we asked whether in this subset of patients erythrocyte count at end of treatment or at follow-up associated with outcome. No association with event-free survival (EFS) and OS was found at the end of the high-risk therapeutic regimen (Supplementary Figure 2A and 2B, respectively), whereas patients with erythrocyte countsat follow-up above the cut-off value displayed significantly better EFS and OS (Supplementary Figure 2C and 2D respectively, p=0.0003).

**Figure 5:**
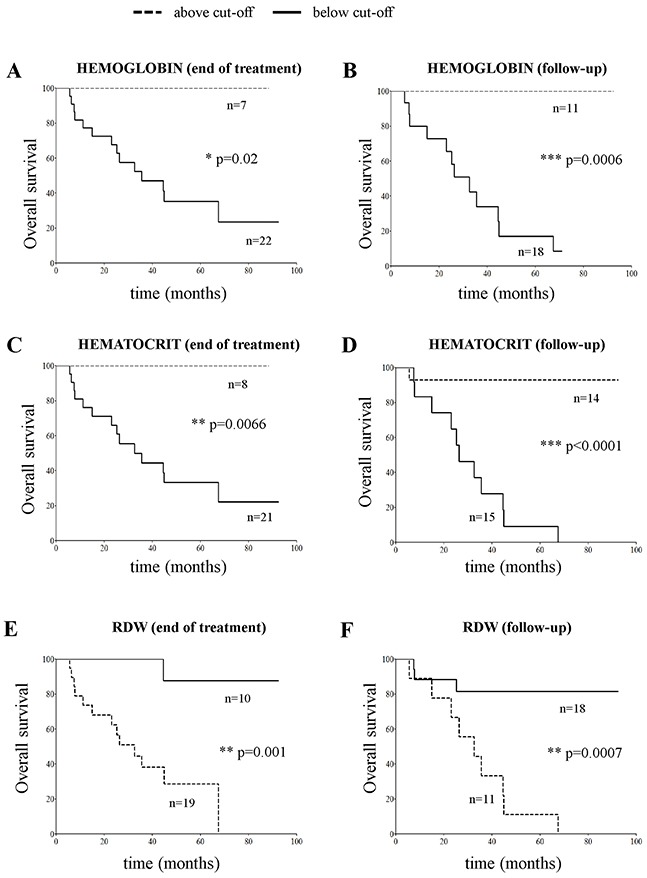
Overall survival plots obtained by stratifying stage M NB patients (N=29) with hemoglobin **(panels A and B)**, hematocrit **(panels C and D)** and RDW values **(panels E and F)** above and below the cut-off value determined by ROC curves at end of cure (11.3 g/dL, 33.7% and 14.4%, panels **A, C** and **E**, respectively) or at follow-up (12.1 g/dL, 35.3% and 15.7 %, panels **B, D**, and **F**, respectively).

Thus, we checked which of the erythrocyte-related analytical parameters, such as mean corpuscular volume (MCV), mean corpuscular hemoglobin (MCH), and mean corpuscular hemoglobin content (MCHC), hemoglobin, hematocrit and red blood cell distribution width (RDW) at end of cure and at follow-up also associated to OS. No association between MCV, MCH and MCHC values was found (data not shown). In contrast, hemoglobin and hematocrit values above the cut-off, and RDW values below the cut-off significantly associated to better OS, both at the end of cure and at follow-up (Figure 5).

Taken together these analyses suggested that maintenance of a low number of dis-homogeneous mature erythrocytes in the periphery may indicate persistence of impaired erythrocyte maturation in the BM. However, none of the growth factors and cytokines involved in erythrocytes’ maturation, such as SCF, IL-3, IL-4, TPO and EPO (http://www.genome.jp/kegg/pathway.html), were under-expressed in BM resident cells from NB patients as compared to healthy children (Supplementary Table 2), suggesting that other, still unknown, factors may have a role.

## DISCUSSION

So far, the physical occupancy of the BM space by metastatic NB cells has been thought to be the cause of impaired BM function observed in children with metastatic NB. Here, we showed that the impairment was selective and occurred only in the erythrocyte lineage, regardless of the presence of metastatic NB cells. Both myeloid- and lymphoid-lineages were normally represented in the BM, as well as the proportion of pro-erythroblasts, baso-erythroblasts and poly-chromatophilic erythroblasts. Conversely, ortho-chromic erythroblast count was significantly lower in NB patients than in healthy children and adults, making it unlikely that flow cytometry results on fresh BM aspirates could be ascribed to difference in age between NB patients and BM donors. We also excluded that the decreased representation of blood group antigens expressed by mature erythrocytes could be ascribed to skewing in NB susceptibility depending on ABO system, as observed in other type of cancers [27–29].

In this study we were unable to identify a potential cause for such a specific impairment of late stage erythrocyte maturation. From the microarray data, no cytokines and/or chemokines implicated in the erythrocyte maturation were significantly under-expressed. The finding of a specific impairment of erythrocyte maturation at late stages may relate to a deficient heme and porphyrin biosynthesis, as suggested by bio-informatics analysis. It is interesting to note that heme biosynthesis occurs in the mitochondria, the site of energetic metabolism and of apoptotic signals [30]. It is well known that oxidative stress is enhanced in NB, and indeed our previous microarray data showed an increase of *SOD* mRNA expression, as well as altered expression of genes involved in glutathione metabolism [24]. Moreover, an ultra-structural study on NB primary tumors recently showed consistent auto-phagic vacuoles and abnormality of mitochondria structure in all undifferentiated NB cells [31]. Therefore, it cannot be excluded that the impaired erythrocyte maturation observed in patients with NB was a peculiar effect consequent to the disruption of mitochondria in this particular lineage, and impairment of mitochondria structure and/or function may be a more general feature of NB tumors.

Since we confirmed here that BM resident cells of patients with NB have similar gene expression profiles and low erythrocyte counts, independently of the physical presence of neoplastic NB cells in the BM space [24], it is conceivable that primary tumor cells produced effects at distance. In this view it is tempting to speculate that the observed impairment could be mediated by extracellular vesicles released by the tumor cells, which transfer protein, mRNA, miRNA, DNA and lipids to other cells [32]. Therefore, further investigations on the potential role of NB-related vesicles are warranted.

Differently from children with metastatic NB, however, children with localized NB had significantly higher platelet and neutrophil counts than healthy children. This finding is in accordance with previous observations in smaller cohorts, showing that the proportion of innate effectors in this subset of patients was higher than in high-risk patients [33–36]. It has been suggested that increased number of innate effectors were responsible for the favorable outcome of patients with localized NB. Here, we showed that the number of neutrophils associated with better survival; however, it remains to be established why children with metastatic disease fail to elicit innate response. Interestingly, in our study no difference was observed in lymphocyte count in both BM and PB, limiting the potential role of this lineage in the natural history of NB.

Our study has some limitations: it is a mono-center, retrospective analysis, which must be confirmed in a prospective cooperative international study. However, the number of patients with localized and metastatic NB, and of controls, is meaningful. In addition, patients were diagnosed throughout a decade at the Italian NB reference center, and they were all treated according to international protocols, thus excluding effects due to different treatment. As stated above, we were unable to identify the possible cause(s) of erythropoiesis impairment in NB. The disruption of mitochondria structure and/or function, as well as the potential role of NB-specific vesicles, as putative player in erythrocyte impairment needs to be further addressed. The study was limited to children with NB; it would be interesting to check whether in children with soft tissue sarcomas, and/or in adult cancers with BM as major site of metastasis, the same selective impairment occurs.

In conclusion, our study showed that NB cells selectively disrupted the late stage of erythrocytes’ maturation, thus decreasing their number in the periphery. Since failure to fully restore erythrocyte count associated with a worse outcome, this observation may be clinically relevant. In addition, future studies may lead to understand the mechanism(s) responsible for the impairment and eventually to a better cure for children with NB.

## MATERIALS AND METHODS

### NB patients

Patients included in the study were diagnosed with NB at the Gaslini Institute between September 2005 and March 2016. Written consent for research use of samples and clinical data was obtained by the legal guardians. The study was approved by the Gaslini Institute Ethical Committee and all analyses were performed according to the Helsinki declaration.

Disease staging was made according to INSS till 2010 [37], and to INRG-SS criteria afterwards [12, 38]. For uniformity, the old INSS stage was converted to the INRG-Staging. After diagnosis, patients were treated according to the European protocol suited for her/his risk category, dependent on age, stage and *MYCN* status.

Demographic, genetic, clinical and follow-up data at 31^st^ December 2016 were retrieved from the Italian NB Registry [39]. The main features of the patients included in the different analysis are summarized in Table 1. All the analyses were made using samples taken at diagnosis.

### Controls

As controls in BM smears’ evaluation and gene expression profiling, samples were obtained from healthy siblings of children admitted at the Gaslini Institute to undergo BM transplants.

As controls in flow cytometer study, BM aspirates were obtained from healthy BM donors, selected according to the Transplant Unit Clinical Protocol of Ematologia 2 at the IRCCS San Martino-IST in Genoa, following a written informed consent at the time of donation. Samples were processed as described [40]. At the end of processing, an aliquot was taken to perform quality control tests. The remaining part was used in this study.

As controls for cell blood counts (CBC), PB samples were obtained from age- and sex-matched healthy children admitted to the Gaslini Institute for accidental injuries.

### RNA extraction, gene expression profiling and gene ontology analysis

Total RNA was extracted from BM aspirates of 13 healthy children, 20 patients with localized NB, 14 GD2-negative fraction of BM aspirates [24] from patients with localized NB and 10 GD2-negative fraction of BM aspirates from patients with metastatic NB. Five-hundred ng of total RNA were hybridized to Human GE 4×44K v2 Microarray Kit (Agilent Technologies, Santa Clara, CA) following the One-color microarray-based gene expression analysis protocol. Slides were scanned by Agilent G2565BA scanner and images were processed by Feature Extraction software v.9.5.3.1. Microarray data are MIAME compliant and are accessible through GEO (http://www.ncbi.nlm.nih.gov/geo/) accession GSE90689.

The genes significantly under-expressed in NB patients were run in the DAVID bioinformatics website (https://david.ncifcrf.gov/) [41].

### Smears analysis, cell blood count (CBC), erythrocyte-related parameters, and blood group antigens

Six May-Grunwald Giemsa-stained smears of BM aspirates were analyzed under light microscope by two independent morphologists (FS and MLB).

CBCs and erythrocyte-related parameters were obtained by using an automated cell counting device (Advia 2120i, Siemens, Milan, Italy).

ABO blood group and Rh negative/positive determinations were made according to certified standard procedures.

### Flow cytometry analysis

Flow cytometer analysis was performed on whole BM samples, using 50 μl of whole blood/tube. Briefly, samples were incubated with specific antibodies (20′ at 4°C in the dark) and then subjected to erythrocytes lysis using BD FACS lysis (BD Biosciences, Milan, Italy) by incubating 15′ at RT in the dark. Cells were washed with PBS 0.5% BSA, suspended in PBS 0.5% BSA and run on Gallios cytometer (Beckman Coulter, Cassina dei Pecchi, Italy), acquiring at least 10^4^ events. Data were analyzed using Kaluza software (Beckman Coulter). The antibodies used were: anti-CD45 PC5 (Beckman Coulter), anti-CD35 FITC (Immunotools, Friesoythe, Germany), anti-CD44 PE (Immunotools), anti-CD117 APC (Miltenyi Biotec, Calderara di Reno, BO, Italy), anti-CD16 PC7 and anti-CD14 FITC (Beckman Coulter), following manufacturer's protocol. PB from 11 NB patients and 10 controls were analyzed for monocytes and granulocytes, whereas PB from 15 NB patients and 10 controls were analyzed for stage II and III erythroblasts.

### Statistical analyses

For gene expression profiling, text files were acquired and analyzed with Rv3.2.1 software using the limma package of Bioconductor [42]. Data were preliminarily corrected for background and normalized in order to have similar distributions across the set of arrays. Ranking of genes by their evidence for differential expression was made using the paired moderated t-statistics based on empirical Bayes moderation of the standard errors.

For other analyses, normal distribution was tested by Kolmogorov-Smirnov. When data distribution was not normal, differences in median between: i) patients and controls, or ii) different groups of patients, were compared by Mann-Whitney test. When data distribution was normal comparison was made by the t Student test. Analyses were made using the Prism software (GraphPad Software Inc., La Jolla, CA).

EFS and OS analyses were performed by Kaplan-Meier method and compared by log-rank test, after determining the cut-off value for each parameter. Cut-off values were determined by ROC curves, using MedCalc Software (version 17.4, MedCalc, Ostend, Belgium) and analysis was performed using method developed by DeLong et al. [43]. ROC curves for each parameter are represented in Supplementary Figure 3.

## SUPPLEMENTARY FIGURES AND TABLES







## Abbreviations

BM: bone marrow
CBC: cell blood counts
EFS: event-free survival
MCH: mean corpuscular hemoglobin
MCHC: mean corpuscular hemoglobin content
MCV: mean corpuscular volume
NB: neuroblastoma
OS: overall survival
PB: peripheral blood
RDW: red blood cell distribution width
