# Supplementary material for: Altered erythropoiesis and decreased number of erythrocytes in children with neuroblastoma
Source: Oncotarget. 2017 May 30;8(32):53194–209. doi: 10.18632/oncotarget.18285 (PMC5581103; doi:10.18632/oncotarget.18285)
Supplement: Supplementary file 1 [file oncotarget-08-53194-s001.pdf]

Altered erythropoiesis and decreased number of erythrocytes in children with neuroblastoma

SUPPLEMENTARY MATERIALS

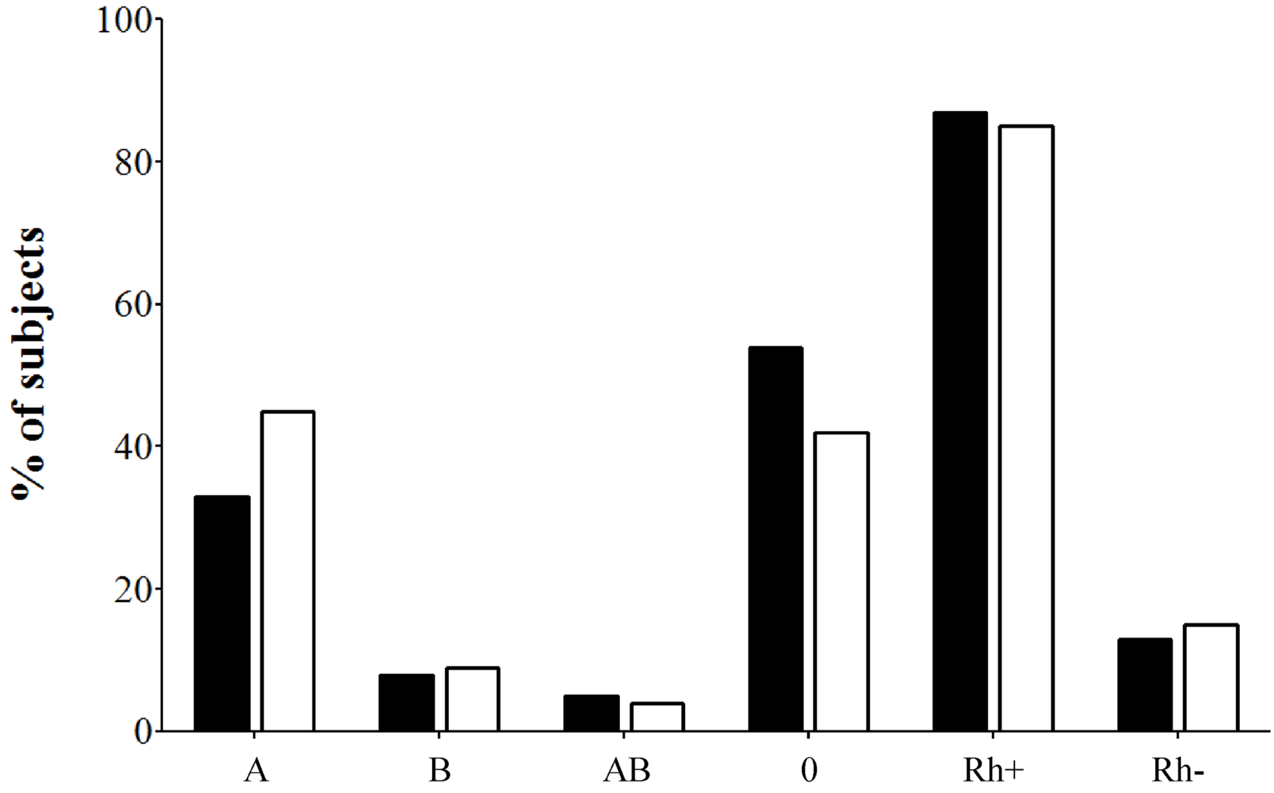

Supplementary Figure 1: Distribution of ABO blood group and RhD antigens in our cohort of patients (black bars) and in the normal European population (white bars).

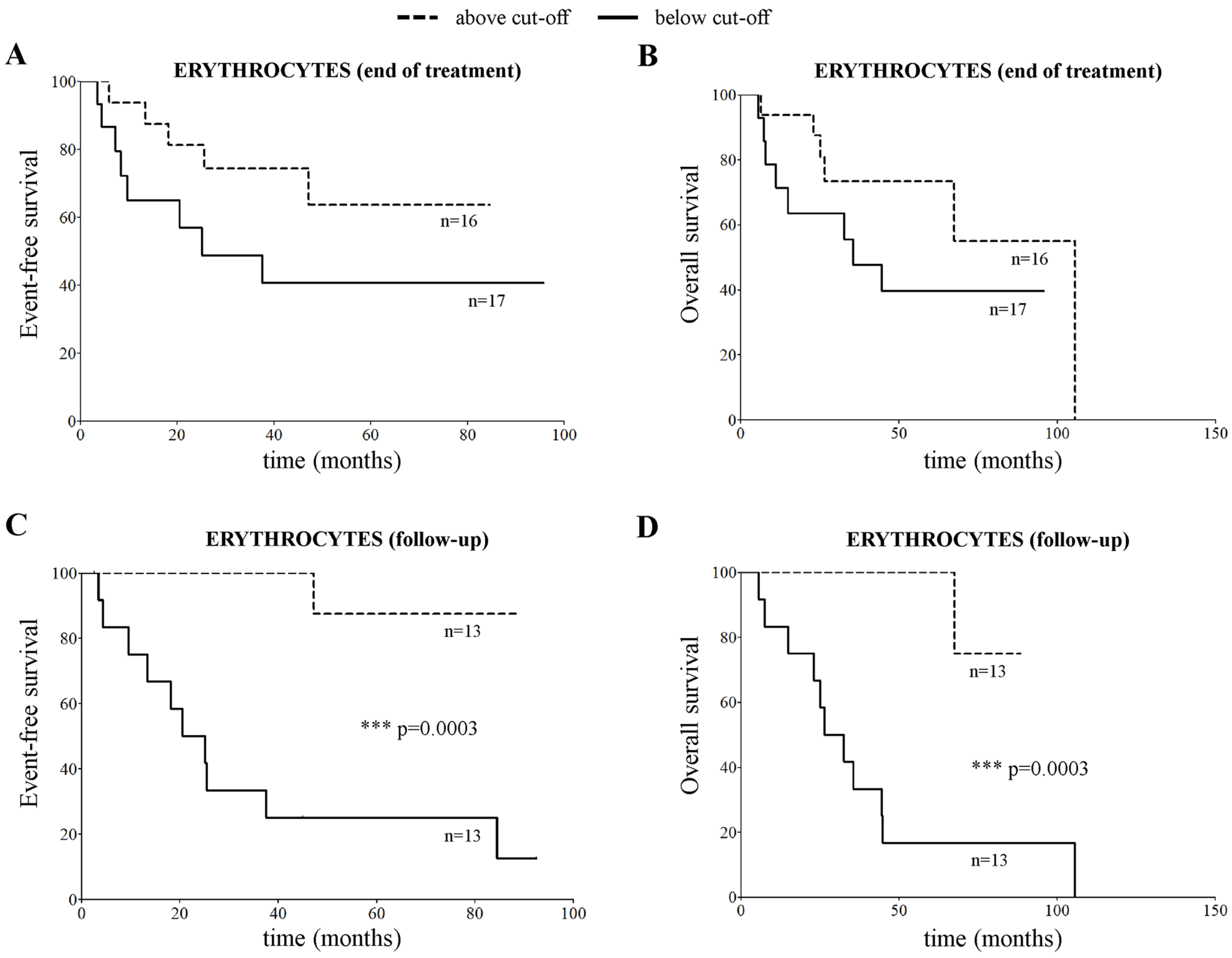

Supplementary Figure 2: Event-free (panels **A** and **C**) and overall (panels **B** and **D**) survival plots obtained by stratifying stage M NB patients according to number of erythrocytes above and below the cut-off value determined by ROC curves at end of cure (3.5x1012erythrocytes/L, panels **A** and **B**) and at follow-up (4.3x1012 erythrocytes/L, panels **C** and **D**).

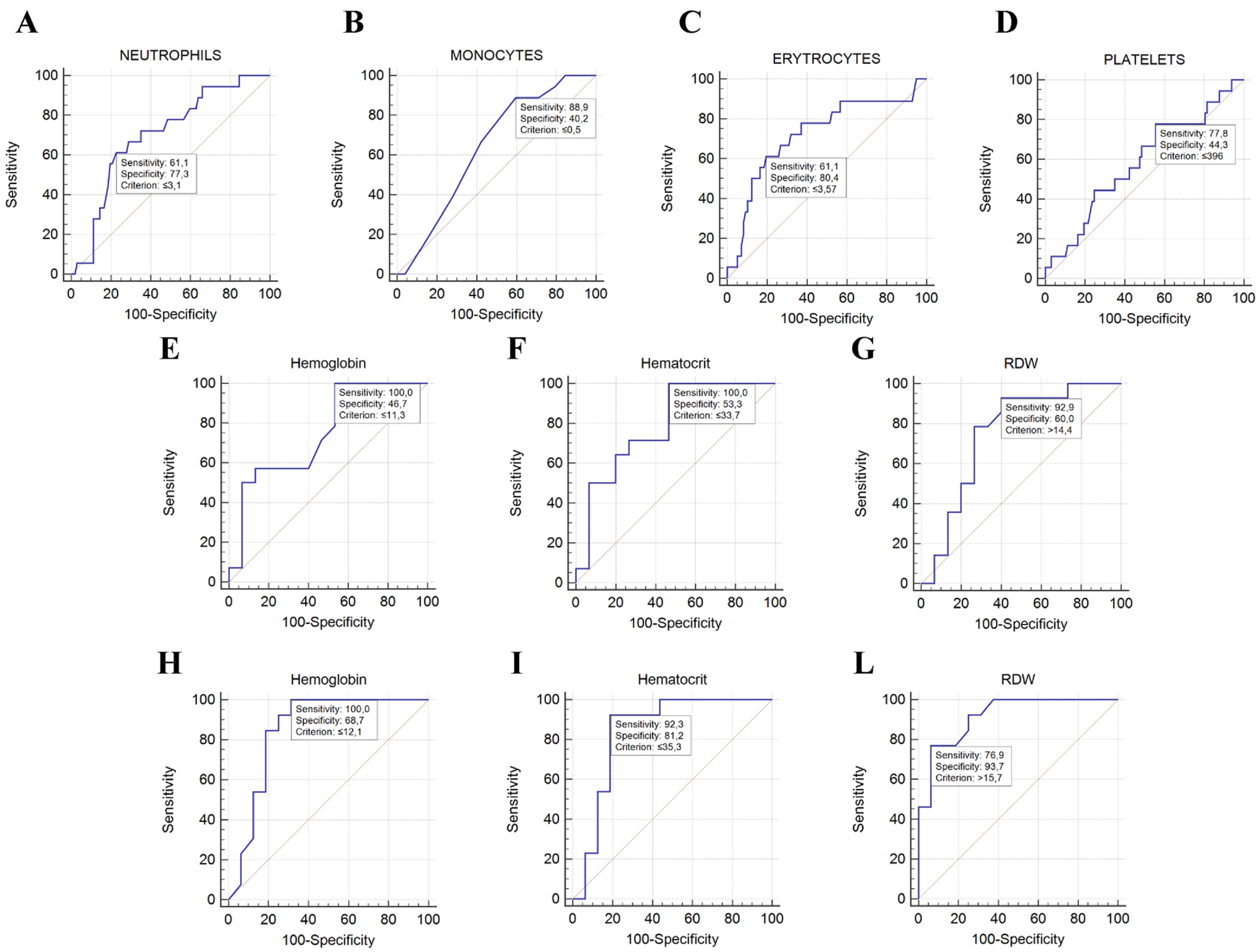

Supplementary Figure 3: ROC curves were generated as described in Materials and Methods section for neutrophils (panel **A**), monocytes (panel **B**), erythrocytes (panel **C**) and platelets (panel **D**). Moreover, ROC curves were generated for hemoglobin (panels **E** and **H**), hematocrit (panels **F** and **I**) and RDW values (panels **G** and **L**) at the end of treatment (panels **E, F** and **G**) and at follow-up (panels **H, I** and **L**).

Supplementary Table 1: (A) Genes significantly (Bonferroni's adjusted P value < 0.01) under-expressed in BM resident cells from patients with metastatic NB as compared to healthy children. (B) Genes significantly (Bonferroni's adjusted P value < 0.01) under-expressedin BM resident cells from patients with localized NB as compared to healthy children. (C) Genes under-expressedin BM resident cells from patients with metastatic NB as compared to patients with localized NB (Bonferroni's adjusted P value was not significant). (D) Genes significantly (Bonferroni's adjusted P value < 0.01) under-expressedin BM resident cells from patients with either metastatic or localized NB as compared to healthy children.E) Genes significantly (Bonferroni's adjusted P value < 0.01)under-expressedin BM resident cells from patients with metastatic and localized NB as compared to healthy children.

See Supplementary File 1

Supplementary Table 2: Functional annotation clustering of genes down-modulated in resident BM cells from NB patients as compared to healthy children.

See Supplementary File 2
