# Supplementary material for: Altered erythropoiesis and decreased number of erythrocytes in children with neuroblastoma
Source: Oncotarget. 2017 May 30;8(32):53194–209. doi: 10.18632/oncotarget.18285 (PMC5581103; doi:10.18632/oncotarget.18285)
Supplement: Supplementary file 2 [file oncotarget-08-53194-s002.docx]

**Supplementary Table 1: A)** Genes significantly (Bonferroni’s adjusted P value < 0.01) down-modulated in BM resident cells from patients with metastatic NB as compared to healthy children.

| **Gene name** | **Systematic name** | **Description** | **Log fold change** | **P value** | **Adjusted P value** |
| --- | --- | --- | --- | --- | --- |
| AQP1 | NM_198098 | Homo sapiens aquaporin 1 (Colton blood group) (AQP1), mRNA [NM_198098] | 3,1684 | 8,58E-11 | 3,73E-07 |
| CTSE | NM_001910 | Homo sapiens cathepsin E (CTSE), transcript variant 1, mRNA [NM_001910] | 1,8313 | 1,74E-09 | 2,72E-06 |
| ENST00000374390 | ENST00000374390 | cellular modulator of immune recognition isoform 7 [Source:RefSeq_peptide;Acc:NP_001002266] [ENST00000374390] | 2,1248 | 2,03E-09 | 2,72E-06 |
| SELENBP1 | NM_003944 | Homo sapiens selenium binding protein 1 (SELENBP1), mRNA [NM_003944] | 2,4938 | 2,21E-09 | 2,72E-06 |
| SLC6A10P | NR_003083 | Homo sapiens solute carrier family 6 (neurotransmitter transporter, creatine), member 10 (pseudogene) (SLC6A10P) on chromosome 16 [NR_003083] | 2,1328 | 1,75E-09 | 2,72E-06 |
| SEPP1 | NM_005410 | Homo sapiens selenoprotein P, plasma, 1 (SEPP1), mRNA [NM_005410] | 1,9872 | 3,27E-09 | 3,19E-06 |
| SLC14A1 | ENST00000321925 | Urea transporter, erythrocyte. [Source:Uniprot/SWISSPROT;Acc:Q13336] [ENST00000321925] | 2,9551 | 2,98E-09 | 3,19E-06 |
| THC2613527 | THC2613527 | Unknown | 1,8692 | 3,33E-09 | 3,19E-06 |
| W60781 | W60781 | W60781 zd26f05.r1 Soares_fetal_heart_NbHH19W Homo sapiens cDNA clone IMAGE:341793 5' similar to gb:J02874 FATTY ACID-BINDING PROTEIN, ADIPOCYTE (HUMAN);, mRNA sequence [W60781] | 2,8533 | 4,52E-09 | 3,54E-06 |
| HEMGN | NM_018437 | Homo sapiens hemogen (HEMGN), transcript variant 1, mRNA [NM_018437] | 3,2740 | 5,55E-09 | 3,83E-06 |
| PAQR9 | NM_198504 | Homo sapiens progestin and adipoQ receptor family member IX (PAQR9), mRNA [NM_198504] | 2,5261 | 5,15E-09 | 3,83E-06 |
| BX537432 | BX537432 | Homo sapiens mRNA; cDNA DKFZp686N09198 (from clone DKFZp686N09198); complete cds. [BX537432] | 2,9787 | 9,03E-09 | 4,53E-06 |
| CA3 | NM_005181 | Homo sapiens carbonic anhydrase III, muscle specific (CA3), mRNA [NM_005181] | 1,2091 | 9,20E-09 | 4,53E-06 |
| CXCL12 | NM_000609 | Homo sapiens chemokine (C-X-C motif) ligand 12 (stromal cell-derived factor 1) (CXCL12), transcript variant 2, mRNA [NM_000609] | 2,0372 | 7,78E-09 | 4,53E-06 |
| FECH | NM_001012515 | Homo sapiens ferrochelatase (protoporphyria) (FECH), nuclear gene encoding mitochondrial protein, transcript variant 1, mRNA [NM_001012515] | 3,5035 | 9,16E-09 | 4,53E-06 |
| THC2669092 | THC2669092 | Unknown | 3,6257 | 8,60E-09 | 4,53E-06 |
| PDZK1IP1 | NM_005764 | Homo sapiens PDZK1 interacting protein 1 (PDZK1IP1), mRNA [NM_005764] | 2,7339 | 1,09E-08 | 4,93E-06 |
| PLEK2 | NM_016445 | Homo sapiens pleckstrin 2 (PLEK2), mRNA [NM_016445] | 2,5339 | 1,12E-08 | 4,95E-06 |
| FAM46C | NM_017709 | Homo sapiens family with sequence similarity 46, member C (FAM46C), mRNA [NM_017709] | 2,1848 | 1,32E-08 | 5,46E-06 |
| THC2633438 | THC2633438 | ALU5_HUMAN (P39192) Alu subfamily SC sequence contamination warning entry, partial (9%) [THC2633438] | 2,0561 | 1,33E-08 | 5,46E-06 |
| SLC25A39 | NM_016016 | Homo sapiens solute carrier family 25, member 39 (SLC25A39), mRNA [NM_016016] | 2,5793 | 1,57E-08 | 6,00E-06 |
| PF4 | NM_002619 | Homo sapiens platelet factor 4 (chemokine (C-X-C motif) ligand 4) (PF4), mRNA [NM_002619] | 3,3678 | 1,96E-08 | 7,17E-06 |
| SNCA | NM_007308 | Homo sapiens synuclein, alpha (non A4 component of amyloid precursor) (SNCA), transcript variant NACP112, mRNA [NM_007308] | 4,1889 | 2,02E-08 | 7,27E-06 |
| GYPB | NM_002100 | Homo sapiens glycophorin B (MNS blood group) (GYPB), mRNA [NM_002100] | 2,2642 | 2,18E-08 | 7,57E-06 |
| NFIX | NM_002501 | Homo sapiens nuclear factor I/X (CCAAT-binding transcription factor) (NFIX), mRNA [NM_002501] | 2,6434 | 2,37E-08 | 8,00E-06 |
| TMOD1 | NM_003275 | Homo sapiens tropomodulin 1 (TMOD1), mRNA [NM_003275] | 1,7091 | 2,65E-08 | 8,47E-06 |
| A_24_P401150 | A_24_P401150 | Unknown | 2,1719 | 3,29E-08 | 8,76E-06 |
| A_32_P78285 | A_32_P78285 | Unknown | 2,4247 | 3,02E-08 | 8,76E-06 |
| C5orf4 | NM_032385 | Homo sapiens chromosome 5 open reading frame 4 (C5orf4), transcript variant 2, mRNA [NM_032385] | 2,9927 | 3,27E-08 | 8,76E-06 |
| FLJ41603 | NM_001001669 | Homo sapiens FLJ41603 protein (FLJ41603), mRNA [NM_001001669] | 1,7713 | 3,18E-08 | 8,76E-06 |
| KIAA1727 | NM_033393 | Homo sapiens KIAA1727 protein (KIAA1727), mRNA [NM_033393] | 1,5328 | 3,35E-08 | 8,76E-06 |
| KLHDC8A | NM_018203 | Homo sapiens kelch domain containing 8A (KLHDC8A), mRNA [NM_018203] | 1,4504 | 3,32E-08 | 8,76E-06 |
| MICAL2 | NM_014632 | Homo sapiens microtubule associated monoxygenase, calponin and LIM domain containing 2 (MICAL2), mRNA [NM_014632] | 2,6707 | 3,13E-08 | 8,76E-06 |
| TNXB | NM_032470 | Homo sapiens tenascin XB (TNXB), transcript variant XB-S, mRNA [NM_032470] | 2,6853 | 3,34E-08 | 8,76E-06 |
| FEM1A | NM_018708 | Homo sapiens fem-1 homolog a (C. elegans) (FEM1A), mRNA [NM_018708] | 1,0544 | 3,54E-08 | 9,01E-06 |
| RHCE | NM_020485 | Homo sapiens Rh blood group, CcEe antigens (RHCE), transcript variant 1, mRNA [NM_020485] | 3,2088 | 3,55E-08 | 9,01E-06 |
| THC2654231 | THC2654231 | ALU5_HUMAN (P39192) Alu subfamily SC sequence contamination warning entry, partial (8%) [THC2654231] | 1,8426 | 3,81E-08 | 9,38E-06 |
| THC2638232 | THC2638232 | Q7RQ28_PLAYO (Q7RQ28) Nuclear protein snf7, partial (7%) [THC2638232] | 1,7815 | 3,93E-08 | 9,54E-06 |
| TRAK2 | NM_015049 | Homo sapiens trafficking protein, kinesin binding 2 (TRAK2), mRNA [NM_015049] | 2,0334 | 4,18E-08 | 1,00E-05 |
| GPR146 | NM_138445 | Homo sapiens G protein-coupled receptor 146 (GPR146), mRNA [NM_138445] | 1,6708 | 4,30E-08 | 1,02E-05 |
| TTC25 | NM_031421 | Homo sapiens tetratricopeptide repeat domain 25 (TTC25), mRNA [NM_031421] | 2,5079 | 4,57E-08 | 1,05E-05 |
| TF | NM_001063 | Homo sapiens transferrin (TF), mRNA [NM_001063] | 2,5178 | 5,13E-08 | 1,13E-05 |
| HAGH | NM_001040427 | Homo sapiens hydroxyacylglutathione hydrolase (HAGH), transcript variant 2, mRNA [NM_001040427] | 2,0226 | 5,61E-08 | 1,18E-05 |
| IL8 | X77737 | H.sapiens mRNA for red cell anion exchanger (EPB3, AE1, Band 3) 3' non-coding region. [X77737] | 3,6423 | 5,79E-08 | 1,19E-05 |
| VCAM1 | NM_001078 | Homo sapiens vascular cell adhesion molecule 1 (VCAM1), transcript variant 1, mRNA [NM_001078] | 1,4784 | 6,68E-08 | 1,31E-05 |
| TBCEL | BC020501 | Homo sapiens leucine rich repeat containing 35, mRNA (cDNA clone IMAGE:3913004). [BC020501] | 1,6505 | 7,02E-08 | 1,34E-05 |
| BPGM | NM_199186 | Homo sapiens 2,3-bisphosphoglycerate mutase (BPGM), transcript variant 2, mRNA [NM_199186] | 3,4983 | 7,63E-08 | 1,41E-05 |
| EPB42 | NM_000119 | Homo sapiens erythrocyte membrane protein band 4.2 (EPB42), mRNA [NM_000119] | 3,6084 | 8,37E-08 | 1,53E-05 |
| MYBL2 | NM_002466 | Homo sapiens v-myb myeloblastosis viral oncogene homolog (avian)-like 2 (MYBL2), mRNA [NM_002466] | 1,2685 | 8,88E-08 | 1,61E-05 |
| SLC1A5 | NM_005628 | Homo sapiens solute carrier family 1 (neutral amino acid transporter), member 5 (SLC1A5), mRNA [NM_005628] | 1,5319 | 9,03E-08 | 1,62E-05 |
| TSPAN5 | NM_005723 | Homo sapiens tetraspanin 5 (TSPAN5), mRNA [NM_005723] | 2,4439 | 1,01E-07 | 1,78E-05 |
| CENPO | AK027859 | Homo sapiens cDNA FLJ14953 fis, clone PLACE3000160. [AK027859] | 1,1191 | 1,06E-07 | 1,83E-05 |
| SLC2A1 | NM_006516 | Homo sapiens solute carrier family 2 (facilitated glucose transporter), member 1 (SLC2A1), mRNA [NM_006516] | 2,8736 | 1,07E-07 | 1,83E-05 |
| WDR40A | NM_015397 | Homo sapiens WD repeat domain 40A (WDR40A), mRNA [NM_015397] | 2,2804 | 1,18E-07 | 1,97E-05 |
| KRT1 | NM_006121 | Homo sapiens keratin 1 (epidermolytic hyperkeratosis) (KRT1), mRNA [NM_006121] | 4,0358 | 1,20E-07 | 1,98E-05 |
| ABCB6 | NM_005689 | Homo sapiens ATP-binding cassette, sub-family B (MDR/TAP), member 6 (ABCB6), nuclear gene encoding mitochondrial protein, mRNA [NM_005689] | 1,8341 | 1,31E-07 | 2,15E-05 |
| ALS2CR2 | NM_018571 | Homo sapiens amyotrophic lateral sclerosis 2 (juvenile) chromosome region, candidate 2 (ALS2CR2), mRNA [NM_018571] | 2,1228 | 1,44E-07 | 2,23E-05 |
| TRIM10 | NM_006778 | Homo sapiens tripartite motif-containing 10 (TRIM10), transcript variant 1, mRNA [NM_006778] | 1,2830 | 1,42E-07 | 2,23E-05 |
| KLF1 | NM_006563 | Homo sapiens Kruppel-like factor 1 (erythroid) (KLF1), mRNA [NM_006563] | 1,7708 | 1,48E-07 | 2,26E-05 |
| THC2688497 | THC2688497 | Q59GX2_HUMAN (Q59GX2) Solute carrier family 2 (Facilitated glucose transporter), member 1 variant (Fragment), partial (6%) [THC2564899] | 2,1725 | 1,56E-07 | 2,32E-05 |
| GMPR | NM_006877 | Homo sapiens guanosine monophosphate reductase (GMPR), mRNA [NM_006877] | 2,3806 | 1,89E-07 | 2,73E-05 |
| THC2656519 | THC2656519 | Unknown | 1,4787 | 1,97E-07 | 2,83E-05 |
| FBXO7 | NM_012179 | Homo sapiens F-box protein 7 (FBXO7), transcript variant 1, mRNA [NM_012179] | 1,7966 | 2,12E-07 | 2,95E-05 |
| SLC4A1 | NM_000342 | Homo sapiens solute carrier family 4, anion exchanger, member 1 (erythrocyte membrane protein band 3, Diego blood group) (SLC4A1), mRNA [NM_000342] | 2,1314 | 2,12E-07 | 2,95E-05 |
| ACSL6 | NM_001009185 | Homo sapiens acyl-CoA synthetase long-chain family member 6 (ACSL6), transcript variant 2, mRNA [NM_001009185] | 2,5007 | 2,21E-07 | 2,97E-05 |
| SEC14L4 | NM_174977 | Homo sapiens SEC14-like 4 (S. cerevisiae) (SEC14L4), mRNA [NM_174977] | 2,4430 | 2,19E-07 | 2,97E-05 |
| HNRPAB | NM_004499 | Homo sapiens heterogeneous nuclear ribonucleoprotein A/B (HNRPAB), transcript variant 2, mRNA [NM_004499] | 1,0689 | 2,46E-07 | 3,26E-05 |
| TRIM58 | NM_015431 | Homo sapiens tripartite motif-containing 58 (TRIM58), mRNA [NM_015431] | 2,2770 | 2,90E-07 | 3,78E-05 |
| CCS | NM_005125 | Homo sapiens copper chaperone for superoxide dismutase (CCS), mRNA [NM_005125] | 1,2512 | 2,96E-07 | 3,81E-05 |
| NAP1L4 | NM_005969 | Homo sapiens nucleosome assembly protein 1-like 4 (NAP1L4), mRNA [NM_005969] | 0,9611 | 2,96E-07 | 3,81E-05 |
| C16orf35 | NM_001039476 | Homo sapiens chromosome 16 open reading frame 35 (C16orf35), transcript variant 2, mRNA [NM_001039476] | 2,0886 | 3,01E-07 | 3,81E-05 |
| THC2588392 | THC2588392 | 1ABW_A Chain A, Deoxy Rhb1.1 (Recombinant Hemoglobin). {synthetic construct} (exp=-1; wgp=-1; cg=-1), partial (18%) [THC2588392] | 3,9880 | 3,06E-07 | 3,85E-05 |
| LOC644462 | XM_930312 | PREDICTED: Homo sapiens similar to amyotrophic lateral sclerosis 2 (juvenile) chromosome region, candidate 2 (LOC644462), mRNA [XM_930312] | 1,8805 | 3,44E-07 | 4,17E-05 |
| XK | NM_021083 | Homo sapiens X-linked Kx blood group (McLeod syndrome) (XK), mRNA [NM_021083] | 2,7244 | 3,39E-07 | 4,17E-05 |
| XPO7 | NM_015024 | Homo sapiens exportin 7 (XPO7), mRNA [NM_015024] | 1,4794 | 3,43E-07 | 4,17E-05 |
| CR601260 | CR601260 | full-length cDNA clone CS0DM001YA20 of Fetal liver of Homo sapiens (human). [CR601260] | 1,6160 | 3,51E-07 | 4,21E-05 |
| GAS2L1 | NM_152237 | Homo sapiens growth arrest-specific 2 like 1 (GAS2L1), transcript variant 3, mRNA [NM_152237] | 2,2944 | 3,52E-07 | 4,21E-05 |
| C14orf45 | NM_025057 | Homo sapiens chromosome 14 open reading frame 45 (C14orf45), mRNA [NM_025057] | 1,2013 | 3,62E-07 | 4,28E-05 |
| RILP | NM_031430 | Homo sapiens Rab interacting lysosomal protein (RILP), mRNA [NM_031430] | 1,7798 | 3,66E-07 | 4,29E-05 |
| EIF2AK1 | NM_014413 | Homo sapiens eukaryotic translation initiation factor 2-alpha kinase 1 (EIF2AK1), mRNA [NM_014413] | 1,4725 | 3,97E-07 | 4,54E-05 |
| TACC3 | NM_006342 | Homo sapiens transforming, acidic coiled-coil containing protein 3 (TACC3), mRNA [NM_006342] | 0,8204 | 4,02E-07 | 4,56E-05 |
| ARHGAP23 | XM_290799 | PREDICTED: Homo sapiens Rho GTPase activating protein 23, transcript variant 1 (ARHGAP23), mRNA [XM_290799] | 1,8323 | 4,39E-07 | 4,92E-05 |
| ATP1B2 | NM_001678 | Homo sapiens ATPase, Na+/K+ transporting, beta 2 polypeptide (ATP1B2), mRNA [NM_001678] | 1,9418 | 4,44E-07 | 4,94E-05 |
| KEL | NM_000420 | Homo sapiens Kell blood group, metallo-endopeptidase (KEL), mRNA [NM_000420] | 1,4690 | 4,47E-07 | 4,94E-05 |
| VWCE | NM_152718 | Homo sapiens von Willebrand factor C and EGF domains (VWCE), mRNA [NM_152718] | 1,9423 | 4,69E-07 | 5,09E-05 |
| ARHGEF12 | NM_015313 | Homo sapiens Rho guanine nucleotide exchange factor (GEF) 12 (ARHGEF12), mRNA [NM_015313] | 1,2901 | 4,75E-07 | 5,11E-05 |
| PHLPPL | NM_015020 | Homo sapiens PH domain and leucine rich repeat protein phosphatase-like (PHLPPL), mRNA [NM_015020] | 1,4243 | 4,81E-07 | 5,11E-05 |
| CLDN5 | NM_003277 | Homo sapiens claudin 5 (transmembrane protein deleted in velocardiofacial syndrome) (CLDN5), mRNA [NM_003277] | 1,7091 | 4,84E-07 | 5,12E-05 |
| ARHGAP19 | NM_032900 | Homo sapiens Rho GTPase activating protein 19 (ARHGAP19), mRNA [NM_032900] | 0,7681 | 5,28E-07 | 5,48E-05 |
| CA1 | NM_001738 | Homo sapiens carbonic anhydrase I (CA1), mRNA [NM_001738] | 3,1381 | 5,50E-07 | 5,57E-05 |
| OR2W3 | NM_001001957 | Homo sapiens olfactory receptor, family 2, subfamily W, member 3 (OR2W3), mRNA [NM_001001957] | 1,5533 | 5,90E-07 | 5,85E-05 |
| EPOR | NM_000121 | Homo sapiens erythropoietin receptor (EPOR), mRNA [NM_000121] | 1,5617 | 6,31E-07 | 6,11E-05 |
| TMCC2 | NM_014858 | Homo sapiens transmembrane and coiled-coil domain family 2 (TMCC2), mRNA [NM_014858] | 1,6254 | 6,40E-07 | 6,13E-05 |
| C13orf8 | NM_032436 | Homo sapiens chromosome 13 open reading frame 8 (C13orf8), mRNA [NM_032436] | 0,9207 | 7,36E-07 | 6,86E-05 |
| GP1BA | J02940 | Human platelet glycoprotein Ib alpha chain mRNA, complete cds. [J02940] | 1,6506 | 7,33E-07 | 6,86E-05 |
| MBP | NM_001025100 | Homo sapiens myelin basic protein (MBP), transcript variant 8, mRNA [NM_001025100] | 1,0712 | 7,33E-07 | 6,86E-05 |
| MICALCL | NM_032867 | Homo sapiens MICAL C-terminal like (MICALCL), mRNA [NM_032867] | 1,2606 | 7,33E-07 | 6,86E-05 |
| TUBB | NM_178014 | Homo sapiens tubulin, beta (TUBB), mRNA [NM_178014] | 0,8993 | 7,53E-07 | 6,98E-05 |
| OSBP2 | NM_030758 | Homo sapiens oxysterol binding protein 2 (OSBP2), transcript variant 1, mRNA [NM_030758] | 1,5629 | 7,77E-07 | 7,13E-05 |
| RGS16 | NM_002928 | Homo sapiens regulator of G-protein signalling 16 (RGS16), mRNA [NM_002928] | 1,4893 | 7,88E-07 | 7,19E-05 |
| LMAN2 | NM_006816 | Homo sapiens lectin, mannose-binding 2 (LMAN2), mRNA [NM_006816] | 0,8950 | 8,00E-07 | 7,24E-05 |
| HMBS | NM_000190 | Homo sapiens hydroxymethylbilane synthase (HMBS), transcript variant 1, mRNA [NM_000190] | 2,7142 | 8,07E-07 | 7,24E-05 |
| GATA1 | NM_002049 | Homo sapiens GATA binding protein 1 (globin transcription factor 1) (GATA1), mRNA [NM_002049] | 1,4390 | 8,25E-07 | 7,33E-05 |
| HBQ1 | NM_005331 | Homo sapiens hemoglobin, theta 1 (HBQ1), mRNA [NM_005331] | 3,8912 | 8,32E-07 | 7,36E-05 |
| CA2 | NM_000067 | Homo sapiens carbonic anhydrase II (CA2), mRNA [NM_000067] | 2,9648 | 8,50E-07 | 7,44E-05 |
| RBM38 | NM_017495 | Homo sapiens RNA binding motif protein 38 (RBM38), transcript variant 1, mRNA [NM_017495] | 1,7083 | 8,70E-07 | 7,54E-05 |
| PRDX2 | NM_005809 | Homo sapiens peroxiredoxin 2 (PRDX2), nuclear gene encoding mitochondrial protein, transcript variant 1, mRNA [NM_005809] | 1,9379 | 9,02E-07 | 7,70E-05 |
| ANKRD25 | NM_015493 | Homo sapiens ankyrin repeat domain 25 (ANKRD25), mRNA [NM_015493] | 1,1075 | 9,19E-07 | 7,74E-05 |
| ENST00000370857 | ENST00000370857 | Muscleblind-like X-linked protein (Muscleblind-like protein 3) (Cys3His CCG1-required protein) (Protein HCHCR). [Source:Uniprot/SWISSPROT;Acc:Q9NUK0] [ENST00000370857] | 1,2113 | 9,20E-07 | 7,74E-05 |
| ART4 | NM_021071 | Homo sapiens ADP-ribosyltransferase 4 (Dombrock blood group) (ART4), mRNA [NM_021071] | 1,9865 | 9,72E-07 | 8,02E-05 |
| DPF3 | AK124946 | Homo sapiens cDNA FLJ42956 fis, clone BRSTN2009899. [AK124946] | 1,5265 | 9,95E-07 | 8,17E-05 |
| ANK1 | NM_000037 | Homo sapiens ankyrin 1, erythrocytic (ANK1), transcript variant 3, mRNA [NM_000037] | 2,3423 | 1,04E-06 | 8,53E-05 |
| ANKRD9 | NM_152326 | Homo sapiens ankyrin repeat domain 9 (ANKRD9), mRNA [NM_152326] | 1,9211 | 1,06E-06 | 8,59E-05 |
| EPB49 | NM_001978 | Homo sapiens erythrocyte membrane protein band 4.9 (dematin) (EPB49), mRNA [NM_001978] | 1,6655 | 1,07E-06 | 8,59E-05 |
| EPN2 | NM_014964 | Homo sapiens epsin 2 (EPN2), transcript variant 2, mRNA [NM_014964] | 1,7207 | 1,09E-06 | 8,77E-05 |
| KIF26A | BC009415 | Homo sapiens kinesin family member 26A, mRNA (cDNA clone IMAGE:3502885), complete cds. [BC009415] | 1,6219 | 1,11E-06 | 8,78E-05 |
| RHAG | NM_000324 | Homo sapiens Rh-associated glycoprotein (RHAG), mRNA [NM_000324] | 2,4501 | 1,10E-06 | 8,78E-05 |
| A_24_P25020 | A_24_P25020 | Unknown | 1,7962 | 1,13E-06 | 8,87E-05 |
| PTPRF | NM_002840 | Homo sapiens protein tyrosine phosphatase, receptor type, F (PTPRF), transcript variant 1, mRNA [NM_002840] | 1,5779 | 1,15E-06 | 9,04E-05 |
| C6orf89 | NM_152734 | Homo sapiens chromosome 6 open reading frame 89 (C6orf89), mRNA [NM_152734] | 0,7086 | 1,16E-06 | 9,06E-05 |
| NT5M | NM_020201 | Homo sapiens 5',3'-nucleotidase, mitochondrial (NT5M), nuclear gene encoding mitochondrial protein, mRNA [NM_020201] | 1,1368 | 1,18E-06 | 9,15E-05 |
| GYPC | NM_002101 | Homo sapiens glycophorin C (Gerbich blood group) (GYPC), transcript variant 1, mRNA [NM_002101] | 1,9539 | 1,20E-06 | 9,25E-05 |
| IDH2 | NM_002168 | Homo sapiens isocitrate dehydrogenase 2 (NADP+), mitochondrial (IDH2), mRNA [NM_002168] | 0,9825 | 1,21E-06 | 9,25E-05 |
| PVRL1 | NM_002855 | Homo sapiens poliovirus receptor-related 1 (herpesvirus entry mediator C; nectin) (PVRL1), transcript variant 1, mRNA [NM_002855] | 1,1301 | 1,21E-06 | 9,25E-05 |
| LOC442239 | XR_018980 | PREDICTED: Homo sapiens similar to Peroxiredoxin-2 (Thioredoxin peroxidase 1) (Thioredoxin-dependent peroxide reductase 1) (Thiol-specific antioxidant protein) (TSA) (PRP) (Natural killer cell-enhancing factor B) (NKEF-B) (LOC442239), mRNA [XR_018980] | 1,8551 | 1,22E-06 | 9,27E-05 |
| HBA2 | NM_000517 | Homo sapiens hemoglobin, alpha 2 (HBA2), mRNA [NM_000517] | 3,0108 | 1,23E-06 | 9,33E-05 |
| ERMAP | NM_001017922 | Homo sapiens erythroblast membrane-associated protein (Scianna blood group) (ERMAP), transcript variant 1, mRNA [NM_001017922] | 1,5953 | 1,25E-06 | 9,37E-05 |
| FZR1 | NM_016263 | Homo sapiens fizzy/cell division cycle 20 related 1 (Drosophila) (FZR1), mRNA [NM_016263] | 0,9545 | 1,25E-06 | 9,37E-05 |
| OPA1 | NM_130837 | Homo sapiens optic atrophy 1 (autosomal dominant) (OPA1), nuclear gene encoding mitochondrial protein, transcript variant 8, mRNA [NM_130837] | 0,9550 | 1,25E-06 | 9,37E-05 |
| A_24_P147849 | A_24_P147849 | Unknown | 4,7259 | 1,30E-06 | 9,61E-05 |
| HBM | NM_001003938 | Homo sapiens hemoglobin, mu (HBM), mRNA [NM_001003938] | 4,2034 | 1,30E-06 | 9,61E-05 |
| PPBP | NM_002704 | Homo sapiens pro-platelet basic protein (chemokine (C-X-C motif) ligand 7) (PPBP), mRNA [NM_002704] | 2,6591 | 1,40E-06 | 1,02E-04 |
| CKAP2L | NM_152515 | Homo sapiens cytoskeleton associated protein 2-like (CKAP2L), mRNA [NM_152515] | 1,1999 | 1,45E-06 | 1,04E-04 |
| CTNNA1 | NM_001903 | Homo sapiens catenin (cadherin-associated protein), alpha 1, 102kDa (CTNNA1), mRNA [NM_001903] | 0,9374 | 1,51E-06 | 1,07E-04 |
| GNPDA1 | NM_005471 | Homo sapiens glucosamine-6-phosphate deaminase 1 (GNPDA1), mRNA [NM_005471] | 0,5706 | 1,51E-06 | 1,07E-04 |
| HBA1 | NM_000558 | Homo sapiens hemoglobin, alpha 1 (HBA1), mRNA [NM_000558] | 2,9567 | 1,63E-06 | 1,14E-04 |
| GYPE | NM_002102 | Homo sapiens glycophorin E (GYPE), transcript variant 1, mRNA [NM_002102] | 1,3812 | 1,65E-06 | 1,14E-04 |
| CMAS | NM_018686 | Homo sapiens cytidine monophosphate N-acetylneuraminic acid synthetase (CMAS), mRNA [NM_018686] | 1,4577 | 1,75E-06 | 1,21E-04 |
| NFIA | NM_005595 | Homo sapiens nuclear factor I/A (NFIA), mRNA [NM_005595] | 1,7816 | 1,83E-06 | 1,24E-04 |
| WDR34 | NM_052844 | Homo sapiens WD repeat domain 34 (WDR34), mRNA [NM_052844] | 1,3666 | 1,83E-06 | 1,24E-04 |
| PRPS1 | NM_002764 | Homo sapiens phosphoribosyl pyrophosphate synthetase 1 (PRPS1), mRNA [NM_002764] | 1,5415 | 1,89E-06 | 1,26E-04 |
| ITGB3 | NM_000212 | Homo sapiens integrin, beta 3 (platelet glycoprotein IIIa, antigen CD61) (ITGB3), mRNA [NM_000212] | 1,4298 | 2,13E-06 | 1,39E-04 |
| CHST2 | NM_004267 | Homo sapiens carbohydrate (N-acetylglucosamine-6-O) sulfotransferase 2 (CHST2), mRNA [NM_004267] | 1,7225 | 2,17E-06 | 1,42E-04 |
| DPM2 | NM_003863 | Homo sapiens dolichyl-phosphate mannosyltransferase polypeptide 2, regulatory subunit (DPM2), mRNA [NM_003863] | 1,2720 | 2,21E-06 | 1,44E-04 |
| AQP3 | NM_004925 | Homo sapiens aquaporin 3 (Gill blood group) (AQP3), mRNA [NM_004925] | 1,4153 | 2,25E-06 | 1,46E-04 |
| SPTA1 | NM_003126 | Homo sapiens spectrin, alpha, erythrocytic 1 (elliptocytosis 2) (SPTA1), mRNA [NM_003126] | 2,9221 | 2,35E-06 | 1,51E-04 |
| GTF3C4 | ENST00000372146 | General transcription factor 3C polypeptide 4 (EC 2.3.1.48) (Transcription factor IIIC subunit delta) (TF3C-delta) (TFIIIC 90 kDa subunit) (TFIIIC 90). [Source:Uniprot/SWISSPROT;Acc:Q9UKN8] [ENST00000372146] | 0,9956 | 2,37E-06 | 1,52E-04 |
| KLHDC3 | NM_057161 | Homo sapiens kelch domain containing 3 (KLHDC3), mRNA [NM_057161] | 0,6048 | 2,38E-06 | 1,52E-04 |
| ABCG2 | NM_004827 | Homo sapiens ATP-binding cassette, sub-family G (WHITE), member 2 (ABCG2), mRNA [NM_004827] | 1,5508 | 2,40E-06 | 1,53E-04 |
| DHRS13 | NM_144683 | Homo sapiens dehydrogenase/reductase (SDR family) member 13 (DHRS13), mRNA [NM_144683] | 1,1653 | 2,44E-06 | 1,54E-04 |
| SPBC24 | NM_182513 | Homo sapiens spindle pole body component 24 homolog (S. cerevisiae) (SPBC24), mRNA [NM_182513] | 0,9951 | 2,60E-06 | 1,62E-04 |
| AK024898 | AK024898 | Homo sapiens cDNA: FLJ21245 fis, clone COL01184. [AK024898] | 0,8737 | 2,62E-06 | 1,63E-04 |
| CDC25A | NM_001789 | Homo sapiens cell division cycle 25 homolog A (S. cerevisiae) (CDC25A), transcript variant 1, mRNA [NM_001789] | 1,2239 | 2,63E-06 | 1,63E-04 |
| GLRX5 | NM_016417 | Homo sapiens glutaredoxin 5 homolog (S. cerevisiae) (GLRX5), mRNA [NM_016417] | 2,2424 | 2,62E-06 | 1,63E-04 |
| A_32_P93894 | A_32_P93894 | Unknown | 0,7274 | 2,68E-06 | 1,64E-04 |
| TMEM23 | NM_147156 | Homo sapiens transmembrane protein 23 (TMEM23), mRNA [NM_147156] | 0,9722 | 2,66E-06 | 1,64E-04 |
| DLC1 | NM_182643 | Homo sapiens deleted in liver cancer 1 (DLC1), transcript variant 1, mRNA [NM_182643] | 1,5804 | 2,75E-06 | 1,66E-04 |
| MCM2 | NM_004526 | Homo sapiens MCM2 minichromosome maintenance deficient 2, mitotin (S. cerevisiae) (MCM2), mRNA [NM_004526] | 1,3904 | 2,86E-06 | 1,72E-04 |
| PRR5 | NM_015366 | Homo sapiens proline rich 5 (renal) (PRR5), transcript variant 2, mRNA [NM_015366] | 1,4070 | 2,88E-06 | 1,73E-04 |
| CLEC1B | NM_016509 | Homo sapiens C-type lectin domain family 1, member B (CLEC1B), mRNA [NM_016509] | 1,1925 | 3,00E-06 | 1,80E-04 |
| ERAF | NM_016633 | Homo sapiens erythroid associated factor (ERAF), mRNA [NM_016633] | 2,8584 | 3,11E-06 | 1,84E-04 |
| ANKH | ENST00000382327 | Progressive ankylosis protein homolog (ANK). [Source:Uniprot/SWISSPROT;Acc:Q9HCJ1] [ENST00000382327] | 1,0591 | 3,32E-06 | 1,95E-04 |
| LTBP1 | NM_206943 | Homo sapiens latent transforming growth factor beta binding protein 1 (LTBP1), transcript variant 1, mRNA [NM_206943] | 0,9113 | 3,34E-06 | 1,96E-04 |
| PGM2L1 | NM_173582 | Homo sapiens phosphoglucomutase 2-like 1 (PGM2L1), mRNA [NM_173582] | 0,7185 | 3,37E-06 | 1,97E-04 |
| ZNF264 | NM_003417 | Homo sapiens zinc finger protein 264 (ZNF264), mRNA [NM_003417] | 0,7350 | 3,73E-06 | 2,15E-04 |
| ITLN1 | NM_017625 | Homo sapiens intelectin 1 (galactofuranose binding) (ITLN1), mRNA [NM_017625] | 2,0350 | 4,07E-06 | 2,31E-04 |
| BC035146 | BC035146 | Homo sapiens cDNA clone IMAGE:5264735. [BC035146] | 1,7062 | 4,19E-06 | 2,35E-04 |
| TIMD4 | NM_138379 | Homo sapiens T-cell immunoglobulin and mucin domain containing 4 (TIMD4), mRNA [NM_138379] | 1,7037 | 4,18E-06 | 2,35E-04 |
| CDYL | NM_170752 | Homo sapiens chromodomain protein, Y-like (CDYL), transcript variant 3, mRNA [NM_170752] | 0,9482 | 4,35E-06 | 2,44E-04 |
| PBX1 | ENST00000328681 | Pre-B-cell leukemia transcription factor 1 (Homeobox protein PBX1) (Homeobox protein PRL). [Source:Uniprot/SWISSPROT;Acc:P40424] [ENST00000328681] | 1,2526 | 4,49E-06 | 2,49E-04 |
| SLC25A38 | NM_017875 | Homo sapiens solute carrier family 25, member 38 (SLC25A38), mRNA [NM_017875] | 1,0453 | 4,53E-06 | 2,50E-04 |
| ENDOD1 | ENST00000278505 | Endonuclease domain-containing 1 protein precursor (EC 3.1.30.-). [Source:Uniprot/SWISSPROT;Acc:O94919] [ENST00000278505] | 1,5554 | 4,64E-06 | 2,54E-04 |
| RAB3IL1 | NM_013401 | Homo sapiens RAB3A interacting protein (rabin3)-like 1 (RAB3IL1), mRNA [NM_013401] | 1,1103 | 4,70E-06 | 2,57E-04 |
| JAZF1 | NM_175061 | Homo sapiens JAZF zinc finger 1 (JAZF1), mRNA [NM_175061] | 1,2666 | 4,77E-06 | 2,59E-04 |
| DCUN1D1 | AF292100 | Homo sapiens RP42 protein mRNA, complete cds. [AF292100] | 1,0267 | 4,80E-06 | 2,60E-04 |
| FLJ36208 | NM_176677 | Homo sapiens hypothetical protein FLJ36208 (FLJ36208), mRNA [NM_176677] | 1,0541 | 4,91E-06 | 2,65E-04 |
| DNM3 | NM_015569 | Homo sapiens dynamin 3 (DNM3), mRNA [NM_015569] | 0,8233 | 4,94E-06 | 2,66E-04 |
| SLC43A1 | NM_003627 | Homo sapiens solute carrier family 43, member 1 (SLC43A1), mRNA [NM_003627] | 0,9965 | 5,08E-06 | 2,71E-04 |
| TAL1 | NM_003189 | Homo sapiens T-cell acute lymphocytic leukemia 1 (TAL1), mRNA [NM_003189] | 1,3839 | 5,51E-06 | 2,89E-04 |
| MOBKL1A | NM_173468 | Homo sapiens MOB1, Mps One Binder kinase activator-like 1A (yeast) (MOBKL1A), mRNA [NM_173468] | 1,7438 | 5,61E-06 | 2,93E-04 |
| DDB1 | NM_001923 | Homo sapiens damage-specific DNA binding protein 1, 127kDa (DDB1), mRNA [NM_001923] | 1,2231 | 5,81E-06 | 3,00E-04 |
| USP12 | ENST00000258451 | Ubiquitin carboxyl-terminal hydrolase 12 (EC 3.1.2.15) (Ubiquitin thioesterase 12) (Ubiquitin-specific-processing protease 12) (Deubiquitinating enzyme 12) (Ubiquitin-hydrolyzing enzyme 1). [Source:Uniprot/SWISSPROT;Acc:O75317] [ENST00000258451] | 1,5367 | 5,80E-06 | 3,00E-04 |
| GNAZ | NM_002073 | Homo sapiens guanine nucleotide binding protein (G protein), alpha z polypeptide (GNAZ), mRNA [NM_002073] | 1,8968 | 6,03E-06 | 3,10E-04 |
| AK1 | NM_000476 | Homo sapiens adenylate kinase 1 (AK1), mRNA [NM_000476] | 1,4010 | 6,19E-06 | 3,18E-04 |
| CD242823 | CD242823 | AGENCOURT_14126724 NIH_MGC_179 Homo sapiens cDNA clone IMAGE:30385216 5', mRNA sequence [CD242823] | 1,2659 | 6,24E-06 | 3,19E-04 |
| THC2663668 | THC2663668 | Unknown | 0,8436 | 6,31E-06 | 3,21E-04 |
| KIFC1 | NM_002263 | Homo sapiens kinesin family member C1 (KIFC1), mRNA [NM_002263] | 1,1662 | 6,38E-06 | 3,24E-04 |
| KCNH2 | NM_172056 | Homo sapiens potassium voltage-gated channel, subfamily H (eag-related), member 2 (KCNH2), transcript variant 2, mRNA [NM_172056] | 1,2653 | 6,70E-06 | 3,35E-04 |
| THC2522223 | THC2522223 | AF192784 makorin 1 {Homo sapiens} (exp=-1; wgp=0; cg=0), partial (15%) [THC2522223] | 1,0009 | 6,71E-06 | 3,35E-04 |
| AY358510 | AY358510 | Homo sapiens clone DNA57836 GLPG464 (UNQ464) mRNA, complete cds. [AY358510] | 1,1998 | 6,78E-06 | 3,35E-04 |
| RNF182 | NM_152737 | Homo sapiens ring finger protein 182 (RNF182), mRNA [NM_152737] | 2,0135 | 6,77E-06 | 3,35E-04 |
| TMEM111 | ENST00000383810 | Transmembrane protein 111. [Source:Uniprot/SWISSPROT;Acc:Q9P0I2] [ENST00000383810] | 1,5334 | 6,74E-06 | 3,35E-04 |
| TGM2 | NM_198951 | Homo sapiens transglutaminase 2 (C polypeptide, protein-glutamine-gamma-glutamyltransferase) (TGM2), transcript variant 2, mRNA [NM_198951] | 1,4898 | 6,88E-06 | 3,37E-04 |
| RCL1 | NM_005772 | Homo sapiens RNA terminal phosphate cyclase-like 1 (RCL1), mRNA [NM_005772] | 1,2480 | 7,01E-06 | 3,41E-04 |
| BLVRB | NM_000713 | Homo sapiens biliverdin reductase B (flavin reductase (NADPH)) (BLVRB), mRNA [NM_000713] | 1,8800 | 7,55E-06 | 3,65E-04 |
| ALAS2 | NM_000032 | Homo sapiens aminolevulinate, delta-, synthase 2 (sideroblastic/hypochromic anemia) (ALAS2), nuclear gene encoding mitochondrial protein, transcript variant 1, mRNA [NM_000032] | 3,9494 | 7,60E-06 | 3,66E-04 |
| BRD3 | NM_007371 | Homo sapiens bromodomain containing 3 (BRD3), mRNA [NM_007371] | 0,9463 | 7,88E-06 | 3,74E-04 |
| TREML1 | AY358357 | Homo sapiens clone DNA82364 GLTL1825 (UNQ1825) mRNA, complete cds. [AY358357] | 1,1775 | 7,86E-06 | 3,74E-04 |
| GYPA | NM_002099 | Homo sapiens glycophorin A (MNS blood group) (GYPA), mRNA [NM_002099] | 1,4525 | 8,17E-06 | 3,86E-04 |
| HBD | NM_000519 | Homo sapiens hemoglobin, delta (HBD), mRNA [NM_000519] | 1,9664 | 8,48E-06 | 3,99E-04 |
| VKORC1 | NM_206824 | Homo sapiens vitamin K epoxide reductase complex, subunit 1 (VKORC1), transcript variant 2, mRNA [NM_206824] | 1,1955 | 8,73E-06 | 4,09E-04 |
| UBE2O | NM_022066 | Homo sapiens ubiquitin-conjugating enzyme E2O (UBE2O), mRNA [NM_022066] | 1,7098 | 8,98E-06 | 4,19E-04 |
| NCOA4 | NM_005437 | Homo sapiens nuclear receptor coactivator 4 (NCOA4), mRNA [NM_005437] | 1,2508 | 9,02E-06 | 4,19E-04 |
| WDR23 | NM_025230 | Homo sapiens WD repeat domain 23 (WDR23), transcript variant 1, mRNA [NM_025230] | 1,6369 | 9,10E-06 | 4,22E-04 |
| GCLC | M90656 | Human gamma-glutamylcysteine synthetase (GCS) mRNA, complete cds. [M90656] | 1,3022 | 9,25E-06 | 4,26E-04 |
| GSPT1 | NM_002094 | Homo sapiens G1 to S phase transition 1 (GSPT1), mRNA [NM_002094] | 1,2071 | 9,35E-06 | 4,29E-04 |
| C1orf128 | NM_020362 | Homo sapiens chromosome 1 open reading frame 128 (C1orf128), mRNA [NM_020362] | 1,2612 | 9,72E-06 | 4,41E-04 |
| UROD | NM_000374 | Homo sapiens uroporphyrinogen decarboxylase (UROD), mRNA [NM_000374] | 2,0202 | 9,79E-06 | 4,43E-04 |
| DAB2 | NM_001343 | Homo sapiens disabled homolog 2, mitogen-responsive phosphoprotein (Drosophila) (DAB2), mRNA [NM_001343] | 0,7509 | 9,85E-06 | 4,45E-04 |
| THC2478531 | THC2478531 | Unknown | 3,3898 | 1,01E-05 | 4,51E-04 |
| CPOX | NM_000097 | Homo sapiens coproporphyrinogen oxidase (CPOX), mRNA [NM_000097] | 1,8058 | 1,01E-05 | 4,53E-04 |
| HPS6 | NM_024747 | Homo sapiens Hermansky-Pudlak syndrome 6 (HPS6), mRNA [NM_024747] | 1,6741 | 1,02E-05 | 4,56E-04 |
| THC2663297 | THC2663297 | Q5VWT3_HUMAN (Q5VWT3) Complement component (3b/4b) receptor 1-like, partial (27%) [THC2663297] | 1,7523 | 1,06E-05 | 4,70E-04 |
| RAB6B | NM_016577 | Homo sapiens RAB6B, member RAS oncogene family (RAB6B), mRNA [NM_016577] | 1,4146 | 1,08E-05 | 4,79E-04 |
| OPTN | NM_001008211 | Homo sapiens optineurin (OPTN), transcript variant 1, mRNA [NM_001008211] | 1,7806 | 1,11E-05 | 4,87E-04 |
| HES5 | NM_001010926 | Homo sapiens hairy and enhancer of split 5 (Drosophila) (HES5), mRNA [NM_001010926] | 1,1238 | 1,16E-05 | 5,04E-04 |
| FBL | NM_001436 | Homo sapiens fibrillarin (FBL), mRNA [NM_001436] | 0,8901 | 1,17E-05 | 5,07E-04 |
| DNAJC7 | NM_003315 | Homo sapiens DnaJ (Hsp40) homolog, subfamily C, member 7 (DNAJC7), mRNA [NM_003315] | 1,0893 | 1,19E-05 | 5,14E-04 |
| ENST00000371189 | ENST00000371189 | Nuclear factor 1 A-type (Nuclear factor 1/A) (NF1-A) (NFI-A) (NF-I/A) (CCAAT-box-binding transcription factor) (CTF) (TGGCA-binding protein). [Source:Uniprot/SWISSPROT;Acc:Q12857] [ENST00000371189] | 1,0484 | 1,20E-05 | 5,15E-04 |
| CCNE1 | NM_001238 | Homo sapiens cyclin E1 (CCNE1), transcript variant 1, mRNA [NM_001238] | 1,1303 | 1,20E-05 | 5,17E-04 |
| FRMD4A | AK057828 | Homo sapiens cDNA FLJ25099 fis, clone CBR01272. [AK057828] | 1,6986 | 1,25E-05 | 5,37E-04 |
| BC039021 | BC039021 | Homo sapiens cDNA clone IMAGE:6043059, partial cds. [BC039021] | 0,9246 | 1,26E-05 | 5,37E-04 |
| FAM83D | NM_030919 | Homo sapiens family with sequence similarity 83, member D (FAM83D), mRNA [NM_030919] | 1,2491 | 1,29E-05 | 5,48E-04 |
| AK125361 | AK125361 | Homo sapiens cDNA FLJ43371 fis, clone NTONG2005969. [AK125361] | 1,6384 | 1,32E-05 | 5,54E-04 |
| MGC17403 | ENST00000314720 | TFS2-M domain-containing protein 1. [Source:Uniprot/SWISSPROT;Acc:Q8N8B7] [ENST00000314720] | 1,0807 | 1,31E-05 | 5,54E-04 |
| LOC253012 | NM_001039372 | Homo sapiens hypothetical protein LOC253012 (LOC253012), transcript variant 1, mRNA [NM_001039372] | 1,9259 | 1,34E-05 | 5,61E-04 |
| BSG | NM_001728 | Homo sapiens basigin (Ok blood group) (BSG), transcript variant 1, mRNA [NM_001728] | 1,3008 | 1,37E-05 | 5,64E-04 |
| C20orf108 | NM_080821 | Homo sapiens chromosome 20 open reading frame 108 (C20orf108), mRNA [NM_080821] | 1,7847 | 1,35E-05 | 5,64E-04 |
| TSPAN17 | NM_012171 | Homo sapiens tetraspanin 17 (TSPAN17), transcript variant 1, mRNA [NM_012171] | 1,0886 | 1,36E-05 | 5,64E-04 |
| BC031344 | BC031344 | Homo sapiens, Similar to makorin, ring finger protein, 1, clone IMAGE:5556543, mRNA. [BC031344] | 1,4016 | 1,39E-05 | 5,72E-04 |
| IFT122 | NM_018262 | Homo sapiens intraflagellar transport 122 homolog (Chlamydomonas) (IFT122), transcript variant 3, mRNA [NM_018262] | 0,5553 | 1,43E-05 | 5,83E-04 |
| WNK1 | AB002342 | Human mRNA for KIAA0344 gene, partial cds. [AB002342] | 1,1360 | 1,43E-05 | 5,83E-04 |
| CDCA4 | NM_017955 | Homo sapiens cell division cycle associated 4 (CDCA4), transcript variant 13, mRNA [NM_017955] | 0,6720 | 1,45E-05 | 5,89E-04 |
| HDLBP | NM_005336 | Homo sapiens high density lipoprotein binding protein (vigilin) (HDLBP), mRNA [NM_005336] | 0,7032 | 1,46E-05 | 5,91E-04 |
| KLF3 | ENST00000381956 | Krueppel-like factor 3 (Basic krueppel-like factor) (CACCC-box-binding protein BKLF) (TEF-2). [Source:Uniprot/SWISSPROT;Acc:P57682] [ENST00000381956] | 1,8085 | 1,49E-05 | 6,03E-04 |
| FLJ30092 | AB014514 | Homo sapiens mRNA for KIAA0614 protein, partial cds. [AB014514] | 1,1043 | 1,55E-05 | 6,23E-04 |
| SLC22A16 | NM_033125 | Homo sapiens solute carrier family 22 (organic cation transporter), member 16 (SLC22A16), mRNA [NM_033125] | 1,6871 | 1,57E-05 | 6,28E-04 |
| UROS | NM_000375 | Homo sapiens uroporphyrinogen III synthase (congenital erythropoietic porphyria) (UROS), mRNA [NM_000375] | 1,0488 | 1,60E-05 | 6,35E-04 |
| C22orf13 | NM_031444 | Homo sapiens chromosome 22 open reading frame 13 (C22orf13), mRNA [NM_031444] | 0,9174 | 1,65E-05 | 6,49E-04 |
| POLR3H | NM_001018051 | Homo sapiens polymerase (RNA) III (DNA directed) polypeptide H (22.9kD) (POLR3H), transcript variant 4, mRNA [NM_001018051] | 1,0171 | 1,67E-05 | 6,54E-04 |
| PPOX | NM_000309 | Homo sapiens protoporphyrinogen oxidase (PPOX), nuclear gene encoding mitochondrial protein, mRNA [NM_000309] | 1,0839 | 1,67E-05 | 6,54E-04 |
| FEN1 | NM_004111 | Homo sapiens flap structure-specific endonuclease 1 (FEN1), mRNA [NM_004111] | 0,9924 | 1,69E-05 | 6,58E-04 |
| CDC27 | NM_001256 | Homo sapiens cell division cycle 27 homolog (S. cerevisiae) (CDC27), mRNA [NM_001256] | 1,2131 | 1,72E-05 | 6,63E-04 |
| CMTM5 | NM_001037288 | Homo sapiens CKLF-like MARVEL transmembrane domain containing 5 (CMTM5), transcript variant 3, mRNA [NM_001037288] | 1,4531 | 1,72E-05 | 6,63E-04 |
| GDPD5 | NM_030792 | Homo sapiens glycerophosphodiester phosphodiesterase domain containing 5 (GDPD5), mRNA [NM_030792] | 1,1895 | 1,72E-05 | 6,63E-04 |
| ACO2 | NM_001098 | Homo sapiens aconitase 2, mitochondrial (ACO2), nuclear gene encoding mitochondrial protein, mRNA [NM_001098] | 0,6723 | 1,76E-05 | 6,78E-04 |
| EFNB3 | NM_001406 | Homo sapiens ephrin-B3 (EFNB3), mRNA [NM_001406] | 3,5802 | 1,77E-05 | 6,80E-04 |
| SIPA1L1 | CR936651 | Homo sapiens mRNA; cDNA DKFZp686G1344 (from clone DKFZp686G1344). [CR936651] | 1,7045 | 1,81E-05 | 6,91E-04 |
| A_32_P208713 | A_32_P208713 | Unknown | 2,3938 | 1,82E-05 | 6,94E-04 |
| ALAD | NM_001003945 | Homo sapiens aminolevulinate, delta-, dehydratase (ALAD), transcript variant 1, mRNA [NM_001003945] | 1,2425 | 1,83E-05 | 6,97E-04 |
| A_23_P84791 | A_23_P84791 | Unknown | 1,5642 | 1,85E-05 | 7,00E-04 |
| BNIP3L | NM_004331 | Homo sapiens BCL2/adenovirus E1B 19kDa interacting protein 3-like (BNIP3L), mRNA [NM_004331] | 1,9910 | 1,89E-05 | 7,14E-04 |
| AADACL1 | NM_020792 | Homo sapiens arylacetamide deacetylase-like 1 (AADACL1), mRNA [NM_020792] | 1,5472 | 1,93E-05 | 7,25E-04 |
| CENPF | NM_016343 | Homo sapiens centromere protein F, 350/400ka (mitosin) (CENPF), mRNA [NM_016343] | 0,7575 | 1,97E-05 | 7,38E-04 |
| MARCH3 | NM_178450 | Homo sapiens membrane-associated ring finger (C3HC4) 3 (MARCH3), mRNA [NM_178450] | 1,2344 | 2,04E-05 | 7,61E-04 |
| KIAA1191 | NM_020444 | Homo sapiens KIAA1191 (KIAA1191), transcript variant 1, mRNA [NM_020444] | 1,0065 | 2,12E-05 | 7,78E-04 |
| YOD1 | NM_018566 | Homo sapiens YOD1 OTU deubiquinating enzyme 1 homolog (S. cerevisiae) (YOD1), mRNA [NM_018566] | 0,8750 | 2,15E-05 | 7,90E-04 |
| FTSJ2 | NM_013393 | Homo sapiens FtsJ homolog 2 (E. coli) (FTSJ2), mRNA [NM_013393] | 0,8823 | 2,22E-05 | 8,13E-04 |
| CDKN2C | NM_078626 | Homo sapiens cyclin-dependent kinase inhibitor 2C (p18, inhibits CDK4) (CDKN2C), transcript variant 2, mRNA [NM_078626] | 1,1639 | 2,25E-05 | 8,20E-04 |
| AKAP7 | NM_016377 | Homo sapiens A kinase (PRKA) anchor protein 7 (AKAP7), transcript variant gamma, mRNA [NM_016377] | 0,7847 | 2,26E-05 | 8,22E-04 |
| EIF4G1 | NM_182917 | Homo sapiens eukaryotic translation initiation factor 4 gamma, 1 (EIF4G1), transcript variant 1, mRNA [NM_182917] | 0,7512 | 2,38E-05 | 8,61E-04 |
| DNAJA4 | NM_018602 | Homo sapiens DnaJ (Hsp40) homolog, subfamily A, member 4 (DNAJA4), mRNA [NM_018602] | 1,1289 | 2,42E-05 | 8,71E-04 |
| C6orf85 | BC022217 | Homo sapiens chromosome 6 open reading frame 85, mRNA (cDNA clone IMAGE:3846727), complete cds. [BC022217] | 2,0389 | 2,47E-05 | 8,84E-04 |
| NEDD4L | NM_015277 | Homo sapiens neural precursor cell expressed, developmentally down-regulated 4-like (NEDD4L), mRNA [NM_015277] | 0,9542 | 2,54E-05 | 9,00E-04 |
| SPTB | NM_001024858 | Homo sapiens spectrin, beta, erythrocytic (includes spherocytosis, clinical type I) (SPTB), transcript variant 1, mRNA [NM_001024858] | 0,8819 | 2,56E-05 | 9,03E-04 |
| FAHD1 | NM_031208 | Homo sapiens fumarylacetoacetate hydrolase domain containing 1 (FAHD1), transcript variant 2, mRNA [NM_031208] | 0,8686 | 2,58E-05 | 9,10E-04 |
| A_24_P7330 | A_24_P7330 | Unknown | 1,5612 | 2,59E-05 | 9,11E-04 |
| ZNF416 | NM_017879 | Homo sapiens zinc finger protein 416 (ZNF416), mRNA [NM_017879] | 0,7526 | 2,59E-05 | 9,11E-04 |
| CDC20 | NM_001255 | Homo sapiens cell division cycle 20 homolog (S. cerevisiae) (CDC20), mRNA [NM_001255] | 1,0183 | 2,62E-05 | 9,17E-04 |
| PAFAH1B1 | NM_000430 | Homo sapiens platelet-activating factor acetylhydrolase, isoform Ib, alpha subunit 45kDa (PAFAH1B1), mRNA [NM_000430] | 1,5454 | 2,65E-05 | 9,23E-04 |
| A_24_P281504 | A_24_P281504 | Unknown | 0,7249 | 2,70E-05 | 9,37E-04 |
| THC2551769 | THC2551769 | AA411302 zv24g06.r1 Soares_NhHMPu_S1 Homo sapiens cDNA clone IMAGE:754618 5', mRNA sequence [AA411302] | 1,2092 | 2,70E-05 | 9,38E-04 |
| LPIN2 | NM_014646 | Homo sapiens lipin 2 (LPIN2), mRNA [NM_014646] | 0,9743 | 2,73E-05 | 9,41E-04 |
| ABCC13 | NR_003088 | Homo sapiens ATP-binding cassette, sub-family C (CFTR/MRP), member 13 (ABCC13) on chromosome 21 [NR_003088] | 1,4432 | 2,76E-05 | 9,47E-04 |
| AFF1 | NM_005935 | Homo sapiens AF4/FMR2 family, member 1 (AFF1), mRNA [NM_005935] | 0,7221 | 2,76E-05 | 9,47E-04 |
| BMP2K | NM_017593 | Homo sapiens BMP2 inducible kinase (BMP2K), transcript variant 2, mRNA [NM_017593] | 0,8589 | 2,80E-05 | 9,58E-04 |
| BC040991 | BC040991 | Homo sapiens cDNA clone IMAGE:4817695. [BC040991] | 0,7737 | 2,86E-05 | 9,74E-04 |
| A_24_P341408 | A_24_P341408 | Unknown | 0,8315 | 2,87E-05 | 9,75E-04 |
| FBXO30 | NM_032145 | Homo sapiens F-box protein 30 (FBXO30), mRNA [NM_032145] | 1,3232 | 2,87E-05 | 9,75E-04 |
| RANBP10 | NM_020850 | Homo sapiens RAN binding protein 10 (RANBP10), mRNA [NM_020850] | 1,4738 | 2,99E-05 | 1,00E-03 |
| MTMR12 | NM_001040446 | Homo sapiens myotubularin related protein 12 (MTMR12), mRNA [NM_001040446] | 0,8502 | 3,04E-05 | 1,02E-03 |
| C19orf57 | NM_024323 | Homo sapiens chromosome 19 open reading frame 57 (C19orf57), mRNA [NM_024323] | 0,7319 | 3,05E-05 | 1,02E-03 |
| CHID1 | NM_023947 | Homo sapiens chitinase domain containing 1 (CHID1), mRNA [NM_023947] | 0,6283 | 3,06E-05 | 1,02E-03 |
| C1orf198 | NM_032800 | Homo sapiens chromosome 1 open reading frame 198 (C1orf198), mRNA [NM_032800] | 1,2754 | 3,08E-05 | 1,02E-03 |
| LOC643960 | XR_019250 | PREDICTED: Homo sapiens similar to ribosomal protein S2 (LOC643960), mRNA [XR_019250] | 0,9512 | 3,22E-05 | 1,06E-03 |
| SFRS2B | NM_032102 | Homo sapiens splicing factor, arginine/serine-rich 2B (SFRS2B), mRNA [NM_032102] | 0,8675 | 3,23E-05 | 1,06E-03 |
| CR603982 | CR603982 | full-length cDNA clone CS0DF021YL03 of Fetal brain of Homo sapiens (human). [CR603982] | 1,3306 | 3,27E-05 | 1,07E-03 |
| LCMT2 | NM_014793 | Homo sapiens leucine carboxyl methyltransferase 2 (LCMT2), mRNA [NM_014793] | 0,7592 | 3,30E-05 | 1,08E-03 |
| PC | NM_001040716 | Homo sapiens pyruvate carboxylase (PC), nuclear gene encoding mitochondrial protein, transcript variant 3, mRNA [NM_001040716] | 1,0257 | 3,31E-05 | 1,08E-03 |
| PKLR | NM_000298 | Homo sapiens pyruvate kinase, liver and RBC (PKLR), nuclear gene encoding mitochondrial protein, transcript variant 1, mRNA [NM_000298] | 0,9025 | 3,31E-05 | 1,08E-03 |
| CCRL2 | NM_003965 | Homo sapiens chemokine (C-C motif) receptor-like 2 (CCRL2), mRNA [NM_003965] | 1,4129 | 3,42E-05 | 1,11E-03 |
| RNF26 | NM_032015 | Homo sapiens ring finger protein 26 (RNF26), mRNA [NM_032015] | 0,7388 | 3,47E-05 | 1,12E-03 |
| RNF187 | BC012758 | Homo sapiens ring finger protein 187, mRNA (cDNA clone IMAGE:3633225), partial cds. [BC012758] | 0,7885 | 3,49E-05 | 1,13E-03 |
| PIP5K1B | NM_003558 | Homo sapiens phosphatidylinositol-4-phosphate 5-kinase, type I, beta (PIP5K1B), transcript variant 2, mRNA [NM_003558] | 1,1404 | 3,50E-05 | 1,13E-03 |
| CSDA | NM_003651 | Homo sapiens cold shock domain protein A (CSDA), mRNA [NM_003651] | 0,9726 | 3,63E-05 | 1,16E-03 |
| C20orf175 | NM_080829 | Homo sapiens chromosome 20 open reading frame 175 (C20orf175), mRNA [NM_080829] | 1,3416 | 3,78E-05 | 1,20E-03 |
| C6orf25 | ENST00000375806 | G6b protein precursor. [Source:Uniprot/SWISSPROT;Acc:O95866] [ENST00000375806] | 1,0755 | 4,07E-05 | 1,29E-03 |
| AL117621 | AL117621 | Homo sapiens mRNA; cDNA DKFZp564M0264 (from clone DKFZp564M0264). [AL117621] | 0,9394 | 4,09E-05 | 1,29E-03 |
| E2F7 | NM_203394 | Homo sapiens E2F transcription factor 7 (E2F7), mRNA [NM_203394] | 1,0028 | 4,10E-05 | 1,29E-03 |
| ARL2 | NM_001667 | Homo sapiens ADP-ribosylation factor-like 2 (ARL2), mRNA [NM_001667] | 0,6028 | 4,12E-05 | 1,29E-03 |
| MYL4 | NM_002476 | Homo sapiens myosin, light chain 4, alkali; atrial, embryonic (MYL4), transcript variant 2, mRNA [NM_002476] | 0,8431 | 4,14E-05 | 1,29E-03 |
| X92493 | X92493 | H.sapiens mRNA for STM-7 protein. [X92493] | 1,0693 | 4,15E-05 | 1,30E-03 |
| FGFR3 | NM_000142 | Homo sapiens fibroblast growth factor receptor 3 (achondroplasia, thanatophoric dwarfism) (FGFR3), transcript variant 1, mRNA [NM_000142] | 0,8633 | 4,25E-05 | 1,32E-03 |
| STEAP3 | NM_182915 | Homo sapiens STEAP family member 3 (STEAP3), transcript variant 1, mRNA [NM_182915] | 0,7427 | 4,26E-05 | 1,32E-03 |
| DCK | NM_000788 | Homo sapiens deoxycytidine kinase (DCK), mRNA [NM_000788] | 1,3175 | 4,30E-05 | 1,33E-03 |
| AK095108 | AK095108 | Homo sapiens cDNA FLJ37789 fis, clone BRHIP3000081. [AK095108] | 0,4548 | 4,32E-05 | 1,33E-03 |
| ASF1A | NM_014034 | Homo sapiens ASF1 anti-silencing function 1 homolog A (S. cerevisiae) (ASF1A), mRNA [NM_014034] | 0,9013 | 4,41E-05 | 1,36E-03 |
| GCDH | NM_013976 | Homo sapiens glutaryl-Coenzyme A dehydrogenase (GCDH), nuclear gene encoding mitochondrial protein, transcript variant 2, mRNA [NM_013976] | 0,6221 | 4,52E-05 | 1,39E-03 |
| PSKH1 | NM_006742 | Homo sapiens protein serine kinase H1 (PSKH1), mRNA [NM_006742] | 0,6862 | 4,56E-05 | 1,40E-03 |
| CAPN1 | NM_005186 | Homo sapiens calpain 1, (mu/I) large subunit (CAPN1), mRNA [NM_005186] | 0,7765 | 4,60E-05 | 1,41E-03 |
| ENST00000383048 | ENST00000383048 | Ig gamma-1 chain C region. [Source:Uniprot/SWISSPROT;Acc:P01857] [ENST00000383048] | 1,4335 | 4,70E-05 | 1,42E-03 |
| RNF123 | NM_022064 | Homo sapiens ring finger protein 123 (RNF123), mRNA [NM_022064] | 1,3459 | 4,68E-05 | 1,42E-03 |
| THC2527772 | THC2527772 | HUMC4AA2 complement component C4A {Homo sapiens} (exp=-1; wgp=0; cg=0), partial (6%) [THC2527772] | 0,8365 | 4,68E-05 | 1,42E-03 |
| SLC7A1 | NM_003045 | Homo sapiens solute carrier family 7 (cationic amino acid transporter, y+ system), member 1 (SLC7A1), mRNA [NM_003045] | 0,8900 | 4,82E-05 | 1,46E-03 |
| ANKRD41 | NM_152363 | Homo sapiens ankyrin repeat domain 41 (ANKRD41), mRNA [NM_152363] | 1,2395 | 4,86E-05 | 1,47E-03 |
| BCL2L1 | NM_138578 | Homo sapiens BCL2-like 1 (BCL2L1), nuclear gene encoding mitochondrial protein, transcript variant 1, mRNA [NM_138578] | 1,6723 | 4,97E-05 | 1,49E-03 |
| CUL4A | NM_001008895 | Homo sapiens cullin 4A (CUL4A), transcript variant 1, mRNA [NM_001008895] | 0,8882 | 5,09E-05 | 1,52E-03 |
| C22orf9 | NM_015264 | Homo sapiens chromosome 22 open reading frame 9 (C22orf9), transcript variant 1, mRNA [NM_015264] | 0,7489 | 5,28E-05 | 1,56E-03 |
| TMEM15 | NM_014908 | Homo sapiens transmembrane protein 15 (TMEM15), mRNA [NM_014908] | 0,6555 | 5,29E-05 | 1,56E-03 |
| AW804491 | AW804491 | AW804491 QV0-UM0093-170400-191-d05 UM0093 Homo sapiens cDNA, mRNA sequence [AW804491] | 0,7267 | 5,32E-05 | 1,57E-03 |
| ACOT7 | NM_007274 | Homo sapiens acyl-CoA thioesterase 7 (ACOT7), transcript variant hBACHa, mRNA [NM_007274] | 0,8619 | 5,38E-05 | 1,58E-03 |
| SNX22 | NM_024798 | Homo sapiens sorting nexin 22 (SNX22), mRNA [NM_024798] | 1,4724 | 5,42E-05 | 1,59E-03 |
| MARCH8 | NM_001002265 | Homo sapiens membrane-associated ring finger (C3HC4) 8 (MARCH8), transcript variant 6, mRNA [NM_001002265] | 0,7747 | 5,48E-05 | 1,61E-03 |
| PRKAR2B | NM_002736 | Homo sapiens protein kinase, cAMP-dependent, regulatory, type II, beta (PRKAR2B), mRNA [NM_002736] | 1,1330 | 5,48E-05 | 1,61E-03 |
| LOC442308 | XR_018043 | PREDICTED: Homo sapiens similar to tubulin, beta 5 (LOC442308), mRNA [XR_018043] | 0,9654 | 5,72E-05 | 1,67E-03 |
| VWF | NM_000552 | Homo sapiens von Willebrand factor (VWF), mRNA [NM_000552] | 1,0615 | 5,79E-05 | 1,68E-03 |
| NUS1 | NM_138459 | Homo sapiens nuclear undecaprenyl pyrophosphate synthase 1 homolog (S. cerevisiae) (NUS1), mRNA [NM_138459] | 0,7093 | 5,83E-05 | 1,69E-03 |
| PIM1 | NM_002648 | Homo sapiens pim-1 oncogene (PIM1), mRNA [NM_002648] | 1,5744 | 5,90E-05 | 1,70E-03 |
| CD36 | S67044 | CD36=collagen type I/thrombospondin receptor {one exon} [human, mRNA Partial, 369 nt]. [S67044] | 1,1920 | 6,05E-05 | 1,74E-03 |
| ODC1 | NM_002539 | Homo sapiens ornithine decarboxylase 1 (ODC1), mRNA [NM_002539] | 1,2389 | 6,11E-05 | 1,76E-03 |
| ZNF526 | NM_133444 | Homo sapiens zinc finger protein 526 (ZNF526), mRNA [NM_133444] | 0,7682 | 6,16E-05 | 1,77E-03 |
| LOC643992 | XR_018270 | PREDICTED: Homo sapiens hypothetical LOC643992 (LOC643992), mRNA [XR_018270] | 0,8737 | 6,37E-05 | 1,82E-03 |
| AF289562 | AF289562 | Homo sapiens clone pp6337 unknown mRNA. [AF289562] | 1,7782 | 6,43E-05 | 1,83E-03 |
| ITGA2B | NM_000419 | Homo sapiens integrin, alpha 2b (platelet glycoprotein IIb of IIb/IIIa complex, antigen CD41) (ITGA2B), mRNA [NM_000419] | 0,8619 | 6,42E-05 | 1,83E-03 |
| TOP1 | NM_003286 | Homo sapiens topoisomerase (DNA) I (TOP1), mRNA [NM_003286] | 0,7892 | 6,48E-05 | 1,84E-03 |
| TFDP1 | NM_007111 | Homo sapiens transcription factor Dp-1 (TFDP1), mRNA [NM_007111] | 0,6318 | 6,51E-05 | 1,84E-03 |
| PPP1R8 | NM_138558 | Homo sapiens protein phosphatase 1, regulatory (inhibitor) subunit 8 (PPP1R8), transcript variant 2, mRNA [NM_138558] | 0,7079 | 6,54E-05 | 1,85E-03 |
| PIP5K2A | NM_005028 | Homo sapiens phosphatidylinositol-4-phosphate 5-kinase, type II, alpha (PIP5K2A), mRNA [NM_005028] | 0,9724 | 6,56E-05 | 1,85E-03 |
| TUBB6 | NM_032525 | Homo sapiens tubulin, beta 6 (TUBB6), mRNA [NM_032525] | 0,6919 | 6,59E-05 | 1,86E-03 |
| VPS41 | BX648347 | Homo sapiens mRNA; cDNA DKFZp686I08170 (from clone DKFZp686I08170). [BX648347] | 0,7106 | 6,84E-05 | 1,91E-03 |
| ZNF776 | AK095607 | Homo sapiens cDNA FLJ38288 fis, clone FCBBF3008382, weakly similar to Homo sapiens C2H2 (Kruppel-type) zinc finger protein mRNA. [AK095607] | 0,5890 | 6,91E-05 | 1,93E-03 |
| CDC34 | NM_004359 | Homo sapiens cell division cycle 34 homolog (S. cerevisiae) (CDC34), mRNA [NM_004359] | 1,2506 | 7,00E-05 | 1,94E-03 |
| PGRMC2 | NM_006320 | Homo sapiens progesterone receptor membrane component 2 (PGRMC2), mRNA [NM_006320] | 1,4610 | 6,99E-05 | 1,94E-03 |
| BTRC | NM_033637 | Homo sapiens beta-transducin repeat containing (BTRC), transcript variant 1, mRNA [NM_033637] | 1,0565 | 7,07E-05 | 1,96E-03 |
| PYCRL | NM_023078 | Homo sapiens pyrroline-5-carboxylate reductase-like (PYCRL), mRNA [NM_023078] | 1,0673 | 7,08E-05 | 1,96E-03 |
| MKRN1 | NM_013446 | Homo sapiens makorin, ring finger protein, 1 (MKRN1), mRNA [NM_013446] | 1,2192 | 7,11E-05 | 1,96E-03 |
| A_24_P325533 | A_24_P325533 | Unknown | 0,7519 | 7,18E-05 | 1,98E-03 |
| TUBA3 | NM_006009 | Homo sapiens tubulin, alpha 3 (TUBA3), mRNA [NM_006009] | 0,9801 | 7,22E-05 | 1,98E-03 |
| A_24_P204604 | A_24_P204604 | Unknown | 0,7128 | 7,24E-05 | 1,99E-03 |
| PET112L | NM_004564 | Homo sapiens PET112-like (yeast) (PET112L), mRNA [NM_004564] | 0,6970 | 7,34E-05 | 2,01E-03 |
| RASIP1 | NM_017805 | Homo sapiens Ras interacting protein 1 (RASIP1), mRNA [NM_017805] | 1,1273 | 7,37E-05 | 2,01E-03 |
| RCCD1 | NM_033544 | Homo sapiens RCC1 domain containing 1 (RCCD1), transcript variant 1, mRNA [NM_033544] | 0,7917 | 7,37E-05 | 2,01E-03 |
| ZBTB3 | NM_024784 | Homo sapiens zinc finger and BTB domain containing 3 (ZBTB3), mRNA [NM_024784] | 0,9820 | 7,36E-05 | 2,01E-03 |
| SHCBP1 | NM_024745 | Homo sapiens SHC SH2-domain binding protein 1 (SHCBP1), mRNA [NM_024745] | 0,8772 | 7,43E-05 | 2,02E-03 |
| COASY | NM_025233 | Homo sapiens Coenzyme A synthase (COASY), transcript variant 1, mRNA [NM_025233] | 1,0717 | 7,45E-05 | 2,02E-03 |
| MINPP1 | NM_004897 | Homo sapiens multiple inositol polyphosphate histidine phosphatase, 1 (MINPP1), mRNA [NM_004897] | 1,2302 | 7,47E-05 | 2,02E-03 |
| IQWD1 | NM_018442 | Homo sapiens IQ motif and WD repeats 1 (IQWD1), transcript variant 1, mRNA [NM_018442] | 0,7254 | 7,58E-05 | 2,05E-03 |
| FUT1 | NM_000148 | Homo sapiens fucosyltransferase 1 (galactoside 2-alpha-L-fucosyltransferase, H blood group) (FUT1), mRNA [NM_000148] | 1,1783 | 7,64E-05 | 2,05E-03 |
| FAM117A | NM_030802 | Homo sapiens family with sequence similarity 117, member A (FAM117A), mRNA [NM_030802] | 1,8450 | 7,88E-05 | 2,10E-03 |
| WBSCR16 | NM_030798 | Homo sapiens Williams-Beuren syndrome chromosome region 16 (WBSCR16), mRNA [NM_030798] | 0,7124 | 8,04E-05 | 2,14E-03 |
| SEC24C | NM_004922 | Homo sapiens SEC24 related gene family, member C (S. cerevisiae) (SEC24C), transcript variant 1, mRNA [NM_004922] | 0,4933 | 8,12E-05 | 2,16E-03 |
| CDC42BPB | NM_006035 | Homo sapiens CDC42 binding protein kinase beta (DMPK-like) (CDC42BPB), mRNA [NM_006035] | 0,6001 | 8,26E-05 | 2,18E-03 |
| DKFZP761M1511 | AK096661 | Homo sapiens cDNA FLJ39342 fis, clone OCBBF2018873. [AK096661] | 1,1151 | 8,31E-05 | 2,19E-03 |
| C4B | NM_001002029 | Homo sapiens complement component 4B (Childo blood group) (C4B), mRNA [NM_001002029] | 1,0472 | 8,37E-05 | 2,20E-03 |
| FAM104A | NM_032837 | Homo sapiens family with sequence similarity 104, member A (FAM104A), mRNA [NM_032837] | 1,7635 | 8,36E-05 | 2,20E-03 |
| SACS | NM_014363 | Homo sapiens spastic ataxia of Charlevoix-Saguenay (sacsin) (SACS), mRNA [NM_014363] | 1,1267 | 8,42E-05 | 2,21E-03 |
| FBXO34 | NM_017943 | Homo sapiens F-box protein 34 (FBXO34), mRNA [NM_017943] | 1,2109 | 8,46E-05 | 2,21E-03 |
| ZDHHC5 | NM_015457 | Homo sapiens zinc finger, DHHC-type containing 5 (ZDHHC5), mRNA [NM_015457] | 0,5973 | 8,71E-05 | 2,26E-03 |
| C14orf169 | NM_024644 | Homo sapiens chromosome 14 open reading frame 169 (C14orf169), mRNA [NM_024644] | 0,9940 | 8,78E-05 | 2,26E-03 |
| CTSB | NM_147780 | Homo sapiens cathepsin B (CTSB), transcript variant 2, mRNA [NM_147780] | 0,8482 | 8,77E-05 | 2,26E-03 |
| CYC1 | NM_001916 | Homo sapiens cytochrome c-1 (CYC1), mRNA [NM_001916] | 0,7446 | 8,76E-05 | 2,26E-03 |
| LNX2 | NM_153371 | Homo sapiens ligand of numb-protein X 2 (LNX2), mRNA [NM_153371] | 0,9312 | 8,91E-05 | 2,28E-03 |
| TNS1 | NM_022648 | Homo sapiens tensin 1 (TNS1), mRNA [NM_022648] | 2,1047 | 8,90E-05 | 2,28E-03 |
| C9orf5 | NM_032012 | Homo sapiens chromosome 9 open reading frame 5 (C9orf5), mRNA [NM_032012] | 1,2820 | 8,98E-05 | 2,29E-03 |
| THC2530075 | THC2530075 | Unknown | 0,7436 | 9,05E-05 | 2,31E-03 |
| THC2649313 | THC2649313 | Unknown | 0,6911 | 9,15E-05 | 2,33E-03 |
| UBE2H | NM_003344 | Homo sapiens ubiquitin-conjugating enzyme E2H (UBC8 homolog, yeast) (UBE2H), transcript variant 1, mRNA [NM_003344] | 0,9997 | 9,28E-05 | 2,35E-03 |
| KIAA1542 | NM_020901 | Homo sapiens CTD-binding SR-like protein rA9 (KIAA1542), mRNA [NM_020901] | 1,2205 | 9,31E-05 | 2,36E-03 |
| SLC25A37 | AK093931 | Homo sapiens cDNA FLJ36612 fis, clone TRACH2016131, highly similar to Homo sapiens mitochondrial solute carrier mRNA. [AK093931] | 0,8333 | 9,34E-05 | 2,36E-03 |
| SDHC | NM_003001 | Homo sapiens succinate dehydrogenase complex, subunit C, integral membrane protein, 15kDa (SDHC), nuclear gene encoding mitochondrial protein, transcript variant 1, mRNA [NM_003001] | 0,8373 | 9,45E-05 | 2,39E-03 |
| ST6GALNAC4 | NM_175039 | Homo sapiens ST6 (alpha-N-acetyl-neuraminyl-2,3-beta-galactosyl-1,3)-N-acetylgalactosaminide alpha-2,6-sialyltransferase 4 (ST6GALNAC4), transcript variant 1, mRNA [NM_175039] | 1,1121 | 9,68E-05 | 2,43E-03 |
| ADD2 | NM_017488 | Homo sapiens adducin 2 (beta) (ADD2), transcript variant beta-4, mRNA [NM_017488] | 0,8919 | 9,75E-05 | 2,45E-03 |
| TUBG1 | NM_001070 | Homo sapiens tubulin, gamma 1 (TUBG1), mRNA [NM_001070] | 1,2408 | 9,78E-05 | 2,45E-03 |
| QSCN6L1 | NM_181701 | Homo sapiens quiescin Q6-like 1 (QSCN6L1), mRNA [NM_181701] | 0,8894 | 9,90E-05 | 2,47E-03 |
| TMPRSS9 | AK131261 | Homo sapiens cDNA FLJ16193 fis, clone BRTHA2018011, weakly similar to EPITHIN (EC 3.4.21.-). [AK131261] | 1,0138 | 1,02E-04 | 2,54E-03 |
| MXI1 | NM_130439 | Homo sapiens MAX interactor 1 (MXI1), transcript variant 2, mRNA [NM_130439] | 1,3925 | 1,02E-04 | 2,54E-03 |
| FZD1 | NM_003505 | Homo sapiens frizzled homolog 1 (Drosophila) (FZD1), mRNA [NM_003505] | 0,6690 | 1,03E-04 | 2,55E-03 |
| NDUFS2 | NM_004550 | Homo sapiens NADH dehydrogenase (ubiquinone) Fe-S protein 2, 49kDa (NADH-coenzyme Q reductase) (NDUFS2), mRNA [NM_004550] | 0,5575 | 1,03E-04 | 2,55E-03 |
| SLC7A5 | NM_003486 | Homo sapiens solute carrier family 7 (cationic amino acid transporter, y+ system), member 5 (SLC7A5), mRNA [NM_003486] | 1,5916 | 1,03E-04 | 2,55E-03 |
| DIAPH3 | NM_001042517 | Homo sapiens diaphanous homolog 3 (Drosophila) (DIAPH3), transcript variant 1, mRNA [NM_001042517] | 0,7358 | 1,05E-04 | 2,58E-03 |
| KIAA0133 | NM_014777 | Homo sapiens KIAA0133 (KIAA0133), mRNA [NM_014777] | 0,5998 | 1,05E-04 | 2,58E-03 |
| PRPF19 | NM_014502 | Homo sapiens PRP19/PSO4 pre-mRNA processing factor 19 homolog (S. cerevisiae) (PRPF19), mRNA [NM_014502] | 0,8958 | 1,05E-04 | 2,58E-03 |
| TUBA6 | NM_032704 | Homo sapiens tubulin, alpha 6 (TUBA6), mRNA [NM_032704] | 0,9843 | 1,06E-04 | 2,59E-03 |
| RPIA | NM_144563 | Homo sapiens ribose 5-phosphate isomerase A (ribose 5-phosphate epimerase) (RPIA), mRNA [NM_144563] | 1,5262 | 1,06E-04 | 2,60E-03 |
| RRM2 | NM_001034 | Homo sapiens ribonucleotide reductase M2 polypeptide (RRM2), mRNA [NM_001034] | 0,9518 | 1,06E-04 | 2,61E-03 |
| DECR2 | NM_020664 | Homo sapiens 2,4-dienoyl CoA reductase 2, peroxisomal (DECR2), mRNA [NM_020664] | 1,1582 | 1,07E-04 | 2,62E-03 |
| RAP2A | NM_021033 | Homo sapiens RAP2A, member of RAS oncogene family (RAP2A), mRNA [NM_021033] | 0,6625 | 1,07E-04 | 2,62E-03 |
| RPUSD2 | NM_152260 | Homo sapiens RNA pseudouridylate synthase domain containing 2 (RPUSD2), mRNA [NM_152260] | 1,1511 | 1,08E-04 | 2,63E-03 |
| C10orf12 | AK025166 | Homo sapiens cDNA: FLJ21513 fis, clone COL05778. [AK025166] | 1,4476 | 1,09E-04 | 2,64E-03 |
| TBC1D14 | NM_020773 | Homo sapiens TBC1 domain family, member 14 (TBC1D14), mRNA [NM_020773] | 0,7106 | 1,10E-04 | 2,65E-03 |
| ALDH5A1 | NM_170740 | Homo sapiens aldehyde dehydrogenase 5 family, member A1 (succinate-semialdehyde dehydrogenase) (ALDH5A1), nuclear gene encoding mitochondrial protein, transcript variant 1, mRNA [NM_170740] | 0,8584 | 1,10E-04 | 2,66E-03 |
| MKI67 | NM_002417 | Homo sapiens antigen identified by monoclonal antibody Ki-67 (MKI67), mRNA [NM_002417] | 0,8928 | 1,11E-04 | 2,66E-03 |
| MGST3 | NM_004528 | Homo sapiens microsomal glutathione S-transferase 3 (MGST3), mRNA [NM_004528] | 1,8191 | 1,14E-04 | 2,72E-03 |
| GPR132 | NM_013345 | Homo sapiens G protein-coupled receptor 132 (GPR132), mRNA [NM_013345] | 1,1503 | 1,14E-04 | 2,72E-03 |
| ENST00000252134 | ENST00000252134 | Uncharacterized protein KIAA0819. [Source:Uniprot/SWISSPROT;Acc:O94909] [ENST00000252134] | 0,8985 | 1,15E-04 | 2,75E-03 |
| HIP2 | NM_005339 | Homo sapiens huntingtin interacting protein 2 (HIP2), mRNA [NM_005339] | 0,6240 | 1,15E-04 | 2,75E-03 |
| AK026372 | AK026372 | Homo sapiens cDNA: FLJ22719 fis, clone HSI14307. [AK026372] | 0,7468 | 1,16E-04 | 2,76E-03 |
| RMND5A | NM_022780 | Homo sapiens required for meiotic nuclear division 5 homolog A (S. cerevisiae) (RMND5A), mRNA [NM_022780] | 0,8053 | 1,16E-04 | 2,76E-03 |
| TH1L | NM_198976 | Homo sapiens TH1-like (Drosophila) (TH1L), transcript variant 1, mRNA [NM_198976] | 0,6661 | 1,16E-04 | 2,76E-03 |
| MCAT | NM_173467 | Homo sapiens malonyl CoA:ACP acyltransferase (mitochondrial) (MCAT), nuclear gene encoding mitochondrial protein, transcript variant 1, mRNA [NM_173467] | 0,6792 | 1,18E-04 | 2,79E-03 |
| AF332145 | AF332145 | Homo sapiens anti-pneumococcal antibody NAD light chain variable region mRNA, partial cds. [AF332145] | 1,5760 | 1,19E-04 | 2,81E-03 |
| CBX7 | NM_175709 | Homo sapiens chromobox homolog 7 (CBX7), mRNA [NM_175709] | 0,8696 | 1,20E-04 | 2,83E-03 |
| LOC388588 | ENST00000378266 | Homo sapiens, clone IMAGE:5162922, mRNA. [BC035379] | 1,6310 | 1,20E-04 | 2,83E-03 |
| ALDH4A1 | NM_003748 | Homo sapiens aldehyde dehydrogenase 4 family, member A1 (ALDH4A1), nuclear gene encoding mitochondrial protein, transcript variant P5CDhL, mRNA [NM_003748] | 0,6516 | 1,21E-04 | 2,84E-03 |
| EGFL8 | NM_030652 | Homo sapiens EGF-like-domain, multiple 8 (EGFL8), mRNA [NM_030652] | 1,1759 | 1,21E-04 | 2,84E-03 |
| TMEM48 | AK091439 | Homo sapiens cDNA FLJ34120 fis, clone FCBBF3009541. [AK091439] | 0,7345 | 1,21E-04 | 2,84E-03 |
| C3orf39 | NM_032806 | Homo sapiens chromosome 3 open reading frame 39 (C3orf39), mRNA [NM_032806] | 1,7317 | 1,23E-04 | 2,88E-03 |
| TTLL12 | NM_015140 | Homo sapiens tubulin tyrosine ligase-like family, member 12 (TTLL12), mRNA [NM_015140] | 1,0781 | 1,25E-04 | 2,91E-03 |
| SLC25A21 | NM_030631 | Homo sapiens solute carrier family 25 (mitochondrial oxodicarboxylate carrier), member 21 (SLC25A21), mRNA [NM_030631] | 0,7612 | 1,26E-04 | 2,93E-03 |
| C6orf59 | AK026765 | Homo sapiens cDNA: FLJ23112 fis, clone LNG07874. [AK026765] | 1,0958 | 1,26E-04 | 2,94E-03 |
| BG259864 | BG259864 | BG259864 602371819F1 NIH_MGC_93 Homo sapiens cDNA clone IMAGE:4479749 5', mRNA sequence [BG259864] | 1,5816 | 1,27E-04 | 2,95E-03 |
| MOBKL2C | NM_145279 | Homo sapiens MOB1, Mps One Binder kinase activator-like 2C (yeast) (MOBKL2C), transcript variant 1, mRNA [NM_145279] | 0,7728 | 1,27E-04 | 2,95E-03 |
| FZD5 | NM_003468 | Homo sapiens frizzled homolog 5 (Drosophila) (FZD5), mRNA [NM_003468] | 1,1953 | 1,30E-04 | 2,99E-03 |
| C20orf121 | NM_024331 | Homo sapiens chromosome 20 open reading frame 121 (C20orf121), transcript variant 1, mRNA [NM_024331] | 1,4208 | 1,33E-04 | 3,05E-03 |
| NP | NM_000270 | Homo sapiens nucleoside phosphorylase (NP), mRNA [NM_000270] | 1,4146 | 1,35E-04 | 3,09E-03 |
| APEH | NM_001640 | Homo sapiens N-acylaminoacyl-peptide hydrolase (APEH), mRNA [NM_001640] | 0,8054 | 1,36E-04 | 3,11E-03 |
| LOC146346 | AK057359 | Homo sapiens cDNA FLJ32797 fis, clone TESTI2002467. [AK057359] | 0,7533 | 1,36E-04 | 3,11E-03 |
| VCP | NM_007126 | Homo sapiens valosin-containing protein (VCP), mRNA [NM_007126] | 0,6782 | 1,37E-04 | 3,11E-03 |
| ECH1 | NM_001398 | Homo sapiens enoyl Coenzyme A hydratase 1, peroxisomal (ECH1), mRNA [NM_001398] | 0,5795 | 1,37E-04 | 3,12E-03 |
| CLCN3 | NM_173872 | Homo sapiens chloride channel 3 (CLCN3), transcript variant e, mRNA [NM_173872] | 0,7317 | 1,38E-04 | 3,13E-03 |
| C5orf30 | NM_033211 | Homo sapiens chromosome 5 open reading frame 30 (C5orf30), mRNA [NM_033211] | 1,1936 | 1,41E-04 | 3,19E-03 |
| TCF20 | NM_005650 | Homo sapiens transcription factor 20 (AR1) (TCF20), transcript variant 1, mRNA [NM_005650] | 0,6817 | 1,42E-04 | 3,20E-03 |
| AMMECR1 | NM_015365 | Homo sapiens Alport syndrome, mental retardation, midface hypoplasia and elliptocytosis chromosomal region, gene 1 (AMMECR1), transcript variant 1, mRNA [NM_015365] | 0,8754 | 1,43E-04 | 3,22E-03 |
| PCAF | NM_003884 | Homo sapiens p300/CBP-associated factor (PCAF), mRNA [NM_003884] | 0,6954 | 1,45E-04 | 3,26E-03 |
| SLC6A8 | NM_005629 | Homo sapiens solute carrier family 6 (neurotransmitter transporter, creatine), member 8 (SLC6A8), mRNA [NM_005629] | 1,4432 | 1,45E-04 | 3,26E-03 |
| WHSC1 | NM_133336 | Homo sapiens Wolf-Hirschhorn syndrome candidate 1 (WHSC1), transcript variant 9, mRNA [NM_133336] | 0,7895 | 1,45E-04 | 3,26E-03 |
| C14orf139 | BC008299 | Homo sapiens chromosome 14 open reading frame 139, mRNA (cDNA clone MGC:14946 IMAGE:3532035), complete cds. [BC008299] | 0,8038 | 1,45E-04 | 3,26E-03 |
| AK130930 | AK130930 | Homo sapiens cDNA FLJ27420 fis, clone WMC07143. [AK130930] | 0,6374 | 1,56E-04 | 3,46E-03 |
| TMED10 | NM_006827 | Homo sapiens transmembrane emp24-like trafficking protein 10 (yeast) (TMED10), mRNA [NM_006827] | 0,8259 | 1,57E-04 | 3,47E-03 |
| SMOX | NM_175839 | Homo sapiens spermine oxidase (SMOX), transcript variant 1, mRNA [NM_175839] | 1,6529 | 1,57E-04 | 3,47E-03 |
| ZNF592 | NM_014630 | Homo sapiens zinc finger protein 592 (ZNF592), mRNA [NM_014630] | 0,5944 | 1,58E-04 | 3,48E-03 |
| COMT | NM_000754 | Homo sapiens catechol-O-methyltransferase (COMT), transcript variant MB-COMT, mRNA [NM_000754] | 0,7529 | 1,60E-04 | 3,52E-03 |
| PPP2R1B | NM_002716 | Homo sapiens protein phosphatase 2 (formerly 2A), regulatory subunit A (PR 65), beta isoform (PPP2R1B), transcript variant 1, mRNA [NM_002716] | 0,6661 | 1,62E-04 | 3,56E-03 |
| CAT | NM_001752 | Homo sapiens catalase (CAT), mRNA [NM_001752] | 1,2672 | 1,63E-04 | 3,57E-03 |
| MRFAP1L1 | NM_152301 | Homo sapiens Morf4 family associated protein 1-like 1 (MRFAP1L1), transcript variant 1, mRNA [NM_152301] | 0,9041 | 1,64E-04 | 3,58E-03 |
| SLC38A5 | NM_033518 | Homo sapiens solute carrier family 38, member 5 (SLC38A5), mRNA [NM_033518] | 1,2830 | 1,65E-04 | 3,59E-03 |
| E2F8 | NM_024680 | Homo sapiens E2F transcription factor 8 (E2F8), mRNA [NM_024680] | 0,9328 | 1,67E-04 | 3,62E-03 |
| HEXA | S76980 | HEXA {HEXA4bpDeltaA mutation, exon 11} [human, Tay-Sachs disease patient, mRNA Partial Mutant, 78 nt]. [S76980] | 0,8910 | 1,69E-04 | 3,66E-03 |
| IGKV1-5 | BC034142 | Homo sapiens immunoglobulin kappa variable 1-5, mRNA (cDNA clone MGC:32715 IMAGE:4694346), complete cds. [BC034142] | 1,6128 | 1,69E-04 | 3,67E-03 |
| THC2506002 | THC2506002 | Unknown | 0,5352 | 1,72E-04 | 3,71E-03 |
| TCEA1 | NM_006756 | Homo sapiens transcription elongation factor A (SII), 1 (TCEA1), transcript variant 1, mRNA [NM_006756] | 0,7728 | 1,72E-04 | 3,72E-03 |
| CR617018 | CR617018 | full-length cDNA clone CS0DG001YH13 of B cells (Ramos cell line) of Homo sapiens (human). [CR617018] | 0,7908 | 1,73E-04 | 3,72E-03 |
| PRMT6 | NM_018137 | Homo sapiens protein arginine methyltransferase 6 (PRMT6), mRNA [NM_018137] | 0,9967 | 1,73E-04 | 3,72E-03 |
| UBADC1 | NM_016172 | Homo sapiens ubiquitin associated domain containing 1 (UBADC1), mRNA [NM_016172] | 1,4078 | 1,73E-04 | 3,73E-03 |
| DOHH | NM_031304 | Homo sapiens deoxyhypusine hydroxylase/monooxygenase (DOHH), mRNA [NM_031304] | 0,9006 | 1,77E-04 | 3,78E-03 |
| BC014395 | BC014395 | Homo sapiens, clone IMAGE:3029191, mRNA. [BC014395] | 0,9881 | 1,77E-04 | 3,79E-03 |
| C18orf24 | NM_001039535 | Homo sapiens chromosome 18 open reading frame 24 (C18orf24), transcript variant 1, mRNA [NM_001039535] | 0,7374 | 1,79E-04 | 3,81E-03 |
| MAP2K3 | NM_145109 | Homo sapiens mitogen-activated protein kinase kinase 3 (MAP2K3), transcript variant B, mRNA [NM_145109] | 1,0821 | 1,81E-04 | 3,86E-03 |
| APOBEC3F | NM_145298 | Homo sapiens apolipoprotein B mRNA editing enzyme, catalytic polypeptide-like 3F (APOBEC3F), transcript variant 1, mRNA [NM_145298] | 0,5064 | 1,82E-04 | 3,86E-03 |
| ZFYVE21 | NM_024071 | Homo sapiens zinc finger, FYVE domain containing 21 (ZFYVE21), mRNA [NM_024071] | 1,6615 | 1,82E-04 | 3,86E-03 |
| CLTC | NM_004859 | Homo sapiens clathrin, heavy chain (Hc) (CLTC), mRNA [NM_004859] | 1,4319 | 1,86E-04 | 3,91E-03 |
| RPP38 | NM_183005 | Homo sapiens ribonuclease P/MRP 38kDa subunit (RPP38), transcript variant 1, mRNA [NM_183005] | 0,7041 | 1,86E-04 | 3,92E-03 |
| GUK1 | NM_000858 | Homo sapiens guanylate kinase 1 (GUK1), mRNA [NM_000858] | 1,0720 | 1,88E-04 | 3,94E-03 |
| SLC12A7 | NM_006598 | Homo sapiens solute carrier family 12 (potassium/chloride transporters), member 7 (SLC12A7), mRNA [NM_006598] | 1,1456 | 1,88E-04 | 3,95E-03 |
| EPC2 | NM_015630 | Homo sapiens enhancer of polycomb homolog 2 (Drosophila) (EPC2), mRNA [NM_015630] | 1,1160 | 1,91E-04 | 3,99E-03 |
| MYH10 | NM_005964 | Homo sapiens myosin, heavy chain 10, non-muscle (MYH10), mRNA [NM_005964] | 0,7637 | 1,93E-04 | 4,03E-03 |
| ABCB10 | NM_012089 | Homo sapiens ATP-binding cassette, sub-family B (MDR/TAP), member 10 (ABCB10), nuclear gene encoding mitochondrial protein, mRNA [NM_012089] | 0,7727 | 1,95E-04 | 4,07E-03 |
| SEPHS1 | NM_012247 | Homo sapiens selenophosphate synthetase 1 (SEPHS1), mRNA [NM_012247] | 0,4423 | 1,96E-04 | 4,07E-03 |
| FHL2 | NM_001039492 | Homo sapiens four and a half LIM domains 2 (FHL2), transcript variant 5, mRNA [NM_001039492] | 1,6207 | 1,97E-04 | 4,09E-03 |
| PIGC | AL035301 | H.sapiens gene from PAC 106H8. [AL035301] | 1,8141 | 2,00E-04 | 4,14E-03 |
| PAGE5 | NM_130467 | Homo sapiens P antigen family, member 5 (prostate associated) (PAGE5), transcript variant 1, mRNA [NM_130467] | 1,1671 | 2,01E-04 | 4,14E-03 |
| FBXW11 | NM_012300 | Homo sapiens F-box and WD-40 domain protein 11 (FBXW11), transcript variant 3, mRNA [NM_012300] | 0,9602 | 2,02E-04 | 4,17E-03 |
| ICMT | NM_012405 | Homo sapiens isoprenylcysteine carboxyl methyltransferase (ICMT), mRNA [NM_012405] | 0,7600 | 2,02E-04 | 4,17E-03 |
| AF086448 | AF086448 | Homo sapiens full length insert cDNA clone ZD82B02. [AF086448] | 1,0710 | 2,05E-04 | 4,20E-03 |
| A_32_P53670 | A_32_P53670 | Unknown | 0,7794 | 2,07E-04 | 4,24E-03 |
| C17orf71 | NM_018149 | Homo sapiens chromosome 17 open reading frame 71 (C17orf71), mRNA [NM_018149] | 0,8214 | 2,08E-04 | 4,25E-03 |
| PIGQ | BC010094 | Homo sapiens phosphatidylinositol glycan, class Q, mRNA (cDNA clone IMAGE:3357878), partial cds. [BC010094] | 1,2152 | 2,08E-04 | 4,25E-03 |
| IFRD2 | NM_006764 | Homo sapiens interferon-related developmental regulator 2 (IFRD2), mRNA [NM_006764] | 0,8282 | 2,10E-04 | 4,28E-03 |
| SON | NM_138927 | Homo sapiens SON DNA binding protein (SON), transcript variant f, mRNA [NM_138927] | 0,8047 | 2,13E-04 | 4,33E-03 |
| GFI1B | NM_004188 | Homo sapiens growth factor independent 1B (potential regulator of CDKN1A, translocated in CML) (GFI1B), mRNA [NM_004188] | 0,7090 | 2,19E-04 | 4,43E-03 |
| TRIM59 | NM_173084 | Homo sapiens tripartite motif-containing 59 (TRIM59), mRNA [NM_173084] | 1,1163 | 2,19E-04 | 4,43E-03 |
| WASF2 | NM_006990 | Homo sapiens WAS protein family, member 2 (WASF2), mRNA [NM_006990] | 0,9429 | 2,19E-04 | 4,44E-03 |
| DNAJB2 | NM_006736 | Homo sapiens DnaJ (Hsp40) homolog, subfamily B, member 2 (DNAJB2), transcript variant 2, mRNA [NM_006736] | 1,1484 | 2,22E-04 | 4,47E-03 |
| FAM122A | NM_138333 | Homo sapiens family with sequence similarity 122A (FAM122A), mRNA [NM_138333] | 0,7929 | 2,23E-04 | 4,48E-03 |
| FBXO18 | NM_178150 | Homo sapiens F-box protein, helicase, 18 (FBXO18), transcript variant 2, mRNA [NM_178150] | 0,6929 | 2,23E-04 | 4,48E-03 |
| SLC22A4 | NM_003059 | Homo sapiens solute carrier family 22 (organic cation transporter), member 4 (SLC22A4), mRNA [NM_003059] | 0,9996 | 2,24E-04 | 4,49E-03 |
| CTB-1048E9.5 | NM_001013694 | Homo sapiens similar to SRR1-like protein (LOC402055), mRNA [NM_001013694] | 0,7197 | 2,30E-04 | 4,59E-03 |
| SLC2A4RG | NM_020062 | Homo sapiens SLC2A4 regulator (SLC2A4RG), mRNA [NM_020062] | 0,5887 | 2,30E-04 | 4,59E-03 |
| WDR13 | NM_017883 | Homo sapiens WD repeat domain 13 (WDR13), mRNA [NM_017883] | 0,6151 | 2,30E-04 | 4,59E-03 |
| A_32_P108592 | A_32_P108592 | Unknown | 0,6642 | 2,34E-04 | 4,63E-03 |
| CR605719 | CR605719 | full-length cDNA clone CS0DK002YG10 of HeLa cells Cot 25-normalized of Homo sapiens (human). [CR605719] | 0,8615 | 2,34E-04 | 4,63E-03 |
| HSMPP8 | NM_017520 | Homo sapiens M-phase phosphoprotein, mpp8 (HSMPP8), mRNA [NM_017520] | 0,7559 | 2,34E-04 | 4,63E-03 |
| WDR90 | NM_145294 | Homo sapiens WD repeat domain 90 (WDR90), mRNA [NM_145294] | 0,5657 | 2,34E-04 | 4,63E-03 |
| PSMF1 | NM_006814 | Homo sapiens proteasome (prosome, macropain) inhibitor subunit 1 (PI31) (PSMF1), transcript variant 1, mRNA [NM_006814] | 1,2944 | 2,35E-04 | 4,64E-03 |
| FARSLA | NM_004461 | Homo sapiens phenylalanine-tRNA synthetase-like, alpha subunit (FARSLA), mRNA [NM_004461] | 0,5014 | 2,36E-04 | 4,64E-03 |
| CHCHD3 | NM_017812 | Homo sapiens coiled-coil-helix-coiled-coil-helix domain containing 3 (CHCHD3), mRNA [NM_017812] | 0,5425 | 2,37E-04 | 4,65E-03 |
| SMCR7L | NM_019008 | Homo sapiens Smith-Magenis syndrome chromosome region, candidate 7-like (SMCR7L), mRNA [NM_019008] | 0,5527 | 2,37E-04 | 4,65E-03 |
| TTC4 | NM_004623 | Homo sapiens tetratricopeptide repeat domain 4 (TTC4), mRNA [NM_004623] | 0,5014 | 2,37E-04 | 4,65E-03 |
| LOC283177 | AK095081 | Homo sapiens cDNA FLJ37762 fis, clone BRHIP2024347, weakly similar to GALECTIN-3. [AK095081] | 2,3107 | 2,40E-04 | 4,68E-03 |
| USP14 | NM_005151 | Homo sapiens ubiquitin specific peptidase 14 (tRNA-guanine transglycosylase) (USP14), transcript variant 1, mRNA [NM_005151] | 0,7806 | 2,45E-04 | 4,78E-03 |
| CKAP2 | NM_018204 | Homo sapiens cytoskeleton associated protein 2 (CKAP2), mRNA [NM_018204] | 1,3464 | 2,47E-04 | 4,82E-03 |
| MST1 | NM_020998 | Homo sapiens macrophage stimulating 1 (hepatocyte growth factor-like) (MST1), mRNA [NM_020998] | 1,0332 | 2,48E-04 | 4,82E-03 |
| PLEKHH3 | NM_024927 | Homo sapiens pleckstrin homology domain containing, family H (with MyTH4 domain) member 3 (PLEKHH3), mRNA [NM_024927] | 1,1599 | 2,49E-04 | 4,84E-03 |
| CALM3 | NM_005184 | Homo sapiens calmodulin 3 (phosphorylase kinase, delta) (CALM3), mRNA [NM_005184] | 0,6628 | 2,50E-04 | 4,85E-03 |
| A_24_P552987 | A_24_P552987 | Unknown | 0,8151 | 2,51E-04 | 4,87E-03 |
| A_23_P28743 | A_23_P28743 | Unknown | 0,6428 | 2,52E-04 | 4,87E-03 |
| TMEPAI | NM_020182 | Homo sapiens transmembrane, prostate androgen induced RNA (TMEPAI), transcript variant 1, mRNA [NM_020182] | 1,0083 | 2,56E-04 | 4,94E-03 |
| CCR5 | NM_000579 | Homo sapiens chemokine (C-C motif) receptor 5 (CCR5), mRNA [NM_000579] | 0,6843 | 2,61E-04 | 5,02E-03 |
| THC2685096 | THC2685096 | Q8HNY5_9NEOP (Q8HNY5) Cytochrome oxidase I (Fragment), partial (9%) [THC2685096] | 0,4869 | 2,70E-04 | 5,17E-03 |
| ALG2 | NM_033087 | Homo sapiens asparagine-linked glycosylation 2 homolog (S. cerevisiae, alpha-1,3-mannosyltransferase) (ALG2), mRNA [NM_033087] | 0,5604 | 2,72E-04 | 5,19E-03 |
| BCL2L11 | NM_138621 | Homo sapiens BCL2-like 11 (apoptosis facilitator) (BCL2L11), transcript variant 1, mRNA [NM_138621] | 1,2083 | 2,72E-04 | 5,19E-03 |
| YPEL4 | NM_145008 | Homo sapiens yippee-like 4 (Drosophila) (YPEL4), mRNA [NM_145008] | 1,1820 | 2,79E-04 | 5,32E-03 |
| TUBB4 | NM_006087 | Homo sapiens tubulin, beta 4 (TUBB4), mRNA [NM_006087] | 0,7354 | 2,79E-04 | 5,32E-03 |
| PSMC5 | NM_002805 | Homo sapiens proteasome (prosome, macropain) 26S subunit, ATPase, 5 (PSMC5), mRNA [NM_002805] | 0,5204 | 2,81E-04 | 5,33E-03 |
| PPAP2B | NM_003713 | Homo sapiens phosphatidic acid phosphatase type 2B (PPAP2B), transcript variant 1, mRNA [NM_003713] | 0,9957 | 2,83E-04 | 5,36E-03 |
| ZNF652 | NM_014897 | Homo sapiens zinc finger protein 652 (ZNF652), mRNA [NM_014897] | 0,6059 | 2,83E-04 | 5,36E-03 |
| BACE2 | NM_012105 | Homo sapiens beta-site APP-cleaving enzyme 2 (BACE2), transcript variant a, mRNA [NM_012105] | 0,8161 | 2,97E-04 | 5,57E-03 |
| ENST00000311061 | ENST00000311061 | qf66c04.x1 Soares_testis_NHT Homo sapiens cDNA clone IMAGE:1754982 3' similar to gb:M96982 SPLICING FACTOR U2AF 35 KD SUBUNIT (HUMAN);, mRNA sequence [AI198876] | 1,0810 | 2,98E-04 | 5,58E-03 |
| FNBP1L | NM_001024948 | Homo sapiens formin binding protein 1-like (FNBP1L), transcript variant 1, mRNA [NM_001024948] | 0,7177 | 3,02E-04 | 5,63E-03 |
| LRBA | NM_006726 | Homo sapiens LPS-responsive vesicle trafficking, beach and anchor containing (LRBA), mRNA [NM_006726] | 0,7062 | 3,06E-04 | 5,69E-03 |
| RGS10 | NM_001005339 | Homo sapiens regulator of G-protein signalling 10 (RGS10), transcript variant 1, mRNA [NM_001005339] | 1,1506 | 3,07E-04 | 5,69E-03 |
| THC2618446 | THC2618446 | Q213Y3_RHOPA (Q213Y3) Single-strand binding protein, partial (9%) [THC2618446] | 0,9106 | 3,10E-04 | 5,74E-03 |
| MGC11102 | NM_032325 | Homo sapiens hypothetical protein MGC11102 (MGC11102), mRNA [NM_032325] | 0,6202 | 3,15E-04 | 5,81E-03 |
| FN3KRP | NM_024619 | Homo sapiens fructosamine-3-kinase-related protein (FN3KRP), mRNA [NM_024619] | 0,7650 | 3,15E-04 | 5,81E-03 |
| PCGF5 | NM_032373 | Homo sapiens polycomb group ring finger 5 (PCGF5), mRNA [NM_032373] | 0,9281 | 3,15E-04 | 5,81E-03 |
| BTBD7 | NM_001002860 | Homo sapiens BTB (POZ) domain containing 7 (BTBD7), transcript variant 1, mRNA [NM_001002860] | 0,9306 | 3,18E-04 | 5,84E-03 |
| PLK1 | NM_005030 | Homo sapiens polo-like kinase 1 (Drosophila) (PLK1), mRNA [NM_005030] | 0,5750 | 3,18E-04 | 5,84E-03 |
| ALDH16A1 | NM_153329 | Homo sapiens aldehyde dehydrogenase 16 family, member A1 (ALDH16A1), mRNA [NM_153329] | 0,5145 | 3,19E-04 | 5,84E-03 |
| GRRP1 | NM_024869 | Homo sapiens glycine/arginine rich protein 1 (GRRP1), mRNA [NM_024869] | 0,7700 | 3,23E-04 | 5,90E-03 |
| C1orf164 | NM_018150 | Homo sapiens chromosome 1 open reading frame 164 (C1orf164), mRNA [NM_018150] | 0,6994 | 3,24E-04 | 5,92E-03 |
| AW302758 | AW302758 | AW302758 xr55g08.x1 NCI_CGAP_Ov26 Homo sapiens cDNA clone IMAGE:2764094 3', mRNA sequence [AW302758] | 1,2747 | 3,27E-04 | 5,96E-03 |
| UBXD1 | NM_025241 | Homo sapiens UBX domain containing 1 (UBXD1), mRNA [NM_025241] | 1,1520 | 3,31E-04 | 6,01E-03 |
| ASCC2 | NM_032204 | Homo sapiens activating signal cointegrator 1 complex subunit 2 (ASCC2), mRNA [NM_032204] | 0,8951 | 3,34E-04 | 6,05E-03 |
| ICAM4 | NM_001544 | Homo sapiens intercellular adhesion molecule 4 (Landsteiner-Wiener blood group) (ICAM4), transcript variant 1, mRNA [NM_001544] | 0,7942 | 3,34E-04 | 6,05E-03 |
| LIG1 | NM_000234 | Homo sapiens ligase I, DNA, ATP-dependent (LIG1), mRNA [NM_000234] | 1,0838 | 3,35E-04 | 6,05E-03 |
| A_24_P298179 | A_24_P298179 | Unknown | 0,9416 | 3,41E-04 | 6,12E-03 |
| ITSN1 | NM_001001132 | Homo sapiens intersectin 1 (SH3 domain protein) (ITSN1), transcript variant 2, mRNA [NM_001001132] | 0,7200 | 3,45E-04 | 6,20E-03 |
| KLF13 | NM_015995 | Homo sapiens Kruppel-like factor 13 (KLF13), mRNA [NM_015995] | 1,1325 | 3,46E-04 | 6,20E-03 |
| GPR137B | AL832142 | Homo sapiens mRNA; cDNA DKFZp686A22111 (from clone DKFZp686A22111). [AL832142] | 0,9650 | 3,46E-04 | 6,20E-03 |
| NUP93 | NM_014669 | Homo sapiens nucleoporin 93kDa (NUP93), mRNA [NM_014669] | 0,6590 | 3,49E-04 | 6,23E-03 |
| C20orf55 | NM_001042353 | Homo sapiens chromosome 20 open reading frame 55 (C20orf55), transcript variant 3, mRNA [NM_001042353] | 0,8620 | 3,53E-04 | 6,29E-03 |
| A2M | NM_000014 | Homo sapiens alpha-2-macroglobulin (A2M), mRNA [NM_000014] | 1,0599 | 3,57E-04 | 6,36E-03 |
| COL6A1 | NM_001848 | Homo sapiens collagen, type VI, alpha 1 (COL6A1), mRNA [NM_001848] | 0,7702 | 3,61E-04 | 6,40E-03 |
| KIF4A | NM_012310 | Homo sapiens kinesin family member 4A (KIF4A), mRNA [NM_012310] | 0,8909 | 3,67E-04 | 6,50E-03 |
| PARVB | NM_001003828 | Homo sapiens parvin, beta (PARVB), transcript variant 1, mRNA [NM_001003828] | 0,7728 | 3,70E-04 | 6,54E-03 |
| ENST00000283657 | ENST00000283657 | V kappa 4=immunoglobulin light chain variable region {complementarity determining regions} [human, CD5+ tonsillar B cells, mRNA PartialMutant, 303 nt]. [S62210] | 1,4439 | 3,71E-04 | 6,56E-03 |
| HDAC4 | NM_006037 | Homo sapiens histone deacetylase 4 (HDAC4), mRNA [NM_006037] | 0,9626 | 3,72E-04 | 6,56E-03 |
| TAF5L | NM_014409 | Homo sapiens TAF5-like RNA polymerase II, p300/CBP-associated factor (PCAF)-associated factor, 65kDa (TAF5L), transcript variant 1, mRNA [NM_014409] | 0,9080 | 3,73E-04 | 6,56E-03 |
| TMEM86B | NM_173804 | Homo sapiens transmembrane protein 86B (TMEM86B), mRNA [NM_173804] | 0,8733 | 3,73E-04 | 6,56E-03 |
| LRRC20 | NM_018205 | Homo sapiens leucine rich repeat containing 20 (LRRC20), transcript variant 3, mRNA [NM_018205] | 0,8046 | 3,74E-04 | 6,56E-03 |
| E2F2 | NM_004091 | Homo sapiens E2F transcription factor 2 (E2F2), mRNA [NM_004091] | 2,1222 | 3,76E-04 | 6,60E-03 |
| EIF3S9 | NM_001037283 | Homo sapiens eukaryotic translation initiation factor 3, subunit 9 eta, 116kDa (EIF3S9), transcript variant 2, mRNA [NM_001037283] | 0,9052 | 3,78E-04 | 6,61E-03 |
| LOC645000 | XR_016848 | PREDICTED: Homo sapiens similar to 40S ribosomal protein S3 (LOC645000), mRNA [XR_016848] | 0,6352 | 3,78E-04 | 6,61E-03 |
| CTCF | NM_006565 | Homo sapiens CCCTC-binding factor (zinc finger protein) (CTCF), mRNA [NM_006565] | 0,7523 | 3,79E-04 | 6,62E-03 |
| BC000986 | BC000986 | Homo sapiens cDNA clone IMAGE:3446313, complete cds. [BC000986] | 0,9988 | 3,80E-04 | 6,63E-03 |
| PAGE2 | NM_207339 | Homo sapiens P antigen family, member 2 (prostate associated) (PAGE2), mRNA [NM_207339] | 1,1143 | 3,80E-04 | 6,63E-03 |
| NPAL3 | NM_020448 | Homo sapiens NIPA-like domain containing 3 (NPAL3), mRNA [NM_020448] | 0,9447 | 3,82E-04 | 6,65E-03 |
| MLLT7 | NM_005938 | Homo sapiens myeloid/lymphoid or mixed-lineage leukemia (trithorax homolog, Drosophila); translocated to, 7 (MLLT7), mRNA [NM_005938] | 0,6771 | 3,83E-04 | 6,66E-03 |
| NUDT4 | NM_199040 | Homo sapiens nudix (nucleoside diphosphate linked moiety X)-type motif 4 (NUDT4), transcript variant 2, mRNA [NM_199040] | 0,8288 | 3,86E-04 | 6,69E-03 |
| PSMD2 | NM_002808 | Homo sapiens proteasome (prosome, macropain) 26S subunit, non-ATPase, 2 (PSMD2), mRNA [NM_002808] | 0,6215 | 3,87E-04 | 6,69E-03 |
| GINS3 | NM_022770 | Homo sapiens GINS complex subunit 3 (Psf3 homolog) (GINS3), mRNA [NM_022770] | 1,3034 | 3,88E-04 | 6,70E-03 |
| ST7 | NM_018412 | Homo sapiens suppression of tumorigenicity 7 (ST7), transcript variant a, mRNA [NM_018412] | 0,6295 | 3,88E-04 | 6,70E-03 |
| C18orf10 | NM_015476 | Homo sapiens chromosome 18 open reading frame 10 (C18orf10), mRNA [NM_015476] | 0,8265 | 3,90E-04 | 6,73E-03 |
| DYRK3 | NM_001004023 | Homo sapiens dual-specificity tyrosine-(Y)-phosphorylation regulated kinase 3 (DYRK3), transcript variant 2, mRNA [NM_001004023] | 0,7234 | 3,95E-04 | 6,80E-03 |
| MFSD5 | NM_032889 | Homo sapiens major facilitator superfamily domain containing 5 (MFSD5), mRNA [NM_032889] | 0,8412 | 3,97E-04 | 6,82E-03 |
| C9orf40 | NM_017998 | Homo sapiens chromosome 9 open reading frame 40 (C9orf40), mRNA [NM_017998] | 1,3682 | 3,99E-04 | 6,84E-03 |
| FAM82C | NM_018145 | Homo sapiens family with sequence similarity 82, member C (FAM82C), mRNA [NM_018145] | 1,1259 | 4,03E-04 | 6,90E-03 |
| TAF4 | NM_003185 | Homo sapiens TAF4 RNA polymerase II, TATA box binding protein (TBP)-associated factor, 135kDa (TAF4), mRNA [NM_003185] | 0,5176 | 4,08E-04 | 6,98E-03 |
| CCDC47 | NM_020198 | Homo sapiens coiled-coil domain containing 47 (CCDC47), mRNA [NM_020198] | 1,1420 | 4,15E-04 | 7,05E-03 |
| RAP2B | NM_002886 | Homo sapiens RAP2B, member of RAS oncogene family (RAP2B), mRNA [NM_002886] | 0,4663 | 4,15E-04 | 7,05E-03 |
| GMEB1 | ENST00000373816 | Glucocorticoid modulatory element-binding protein 1 (GMEB-1) (Parvovirus initiation factor p96) (PIF p96) (DNA-binding protein p96PIF). [Source:Uniprot/SWISSPROT;Acc:Q9Y692] [ENST00000373816] | 0,8645 | 4,17E-04 | 7,08E-03 |
| THC2664480 | THC2664480 | ALU1_HUMAN (P39188) Alu subfamily J sequence contamination warning entry, partial (6%) [THC2664480] | 0,5027 | 4,19E-04 | 7,10E-03 |
| FLJ20489 | NM_017842 | Homo sapiens hypothetical protein FLJ20489 (FLJ20489), mRNA [NM_017842] | 0,7725 | 4,21E-04 | 7,13E-03 |
| AF086139 | AF086139 | Homo sapiens full length insert cDNA clone ZA91F08. [AF086139] | 1,0060 | 4,22E-04 | 7,14E-03 |
| TCF19 | NM_007109 | Homo sapiens transcription factor 19 (SC1) (TCF19), transcript variant 1, mRNA [NM_007109] | 0,4540 | 4,22E-04 | 7,14E-03 |
| ARMC1 | NM_018120 | Homo sapiens armadillo repeat containing 1 (ARMC1), mRNA [NM_018120] | 0,6895 | 4,24E-04 | 7,16E-03 |
| BC038512 | BC038512 | Homo sapiens cDNA clone IMAGE:5262734. [BC038512] | 0,5747 | 4,26E-04 | 7,18E-03 |
| HSPC142 | NM_001033549 | Homo sapiens HSPC142 protein (HSPC142), transcript variant 1, mRNA [NM_001033549] | 0,5326 | 4,27E-04 | 7,18E-03 |
| HNRPUL2 | XM_495877 | PREDICTED: Homo sapiens heterogeneous nuclear ribonucleoprotein U-like 2 (HNRPUL2), mRNA [XM_495877] | 0,6904 | 4,28E-04 | 7,18E-03 |
| CKAP5 | NM_001008938 | Homo sapiens cytoskeleton associated protein 5 (CKAP5), transcript variant 1, mRNA [NM_001008938] | 1,1547 | 4,32E-04 | 7,25E-03 |
| CR606637 | CR606637 | full-length cDNA clone CS0DI007YA21 of Placenta Cot 25-normalized of Homo sapiens (human). [CR606637] | 0,6307 | 4,35E-04 | 7,26E-03 |
| TERF2IP | NM_018975 | Homo sapiens telomeric repeat binding factor 2, interacting protein (TERF2IP), mRNA [NM_018975] | 1,1080 | 4,34E-04 | 7,26E-03 |
| MXRA8 | NM_032348 | Homo sapiens matrix-remodelling associated 8 (MXRA8), mRNA [NM_032348] | 0,8786 | 4,42E-04 | 7,35E-03 |
| SDC1 | NM_001006946 | Homo sapiens syndecan 1 (SDC1), transcript variant 1, mRNA [NM_001006946] | 1,3373 | 4,42E-04 | 7,35E-03 |
| TMEM64 | AK095472 | Homo sapiens cDNA FLJ38153 fis, clone DFNES1000083. [AK095472] | 0,5890 | 4,42E-04 | 7,35E-03 |
| PFDN6 | NM_014260 | Homo sapiens prefoldin subunit 6 (PFDN6), mRNA [NM_014260] | 1,1456 | 4,54E-04 | 7,50E-03 |
| FIS1 | NM_016068 | Homo sapiens fission 1 (mitochondrial outer membrane) homolog (S. cerevisiae) (FIS1), mRNA [NM_016068] | 0,6624 | 4,55E-04 | 7,52E-03 |
| RABGAP1L | NM_014857 | Homo sapiens RAB GTPase activating protein 1-like (RABGAP1L), transcript variant 1, mRNA [NM_014857] | 0,4538 | 4,56E-04 | 7,52E-03 |
| AK090416 | AK090416 | Homo sapiens mRNA for FLJ00318 protein. [AK090416] | 0,5736 | 4,62E-04 | 7,60E-03 |
| BM455859 | BM455859 | AGENCOURT_6409185 NIH_MGC_85 Homo sapiens cDNA clone IMAGE:5498310 5', mRNA sequence [BM455859] | 0,4705 | 4,63E-04 | 7,60E-03 |
| BC062753 | BC062753 | Homo sapiens cDNA clone IMAGE:3933366, partial cds. [BC062753] | 0,7578 | 4,65E-04 | 7,62E-03 |
| TOP2A | NM_001067 | Homo sapiens topoisomerase (DNA) II alpha 170kDa (TOP2A), mRNA [NM_001067] | 1,3724 | 4,65E-04 | 7,62E-03 |
| APRIN | NM_015032 | Homo sapiens androgen-induced proliferation inhibitor (APRIN), mRNA [NM_015032] | 0,5392 | 4,67E-04 | 7,64E-03 |
| FLJ20105 | NM_001009954 | Homo sapiens FLJ20105 protein (FLJ20105), transcript variant 2, mRNA [NM_001009954] | 0,6487 | 4,68E-04 | 7,65E-03 |
| GNA12 | NM_007353 | Homo sapiens guanine nucleotide binding protein (G protein) alpha 12 (GNA12), mRNA [NM_007353] | 0,8584 | 4,73E-04 | 7,72E-03 |
| METAP2 | NM_006838 | Homo sapiens methionyl aminopeptidase 2 (METAP2), mRNA [NM_006838] | 0,9235 | 4,73E-04 | 7,72E-03 |
| MGC5139 | BC004815 | Homo sapiens hypothetical protein MGC5139, mRNA (cDNA clone IMAGE:3448346), complete cds. [BC004815] | 0,6569 | 4,74E-04 | 7,73E-03 |
| RNF14 | NM_004290 | Homo sapiens ring finger protein 14 (RNF14), transcript variant 1, mRNA [NM_004290] | 0,6559 | 4,80E-04 | 7,81E-03 |
| PUS1 | NM_025215 | Homo sapiens pseudouridylate synthase 1 (PUS1), transcript variant 1, mRNA [NM_025215] | 0,6878 | 4,82E-04 | 7,83E-03 |
| MRFAP1 | NM_033296 | Homo sapiens Mof4 family associated protein 1 (MRFAP1), mRNA [NM_033296] | 0,7528 | 4,83E-04 | 7,84E-03 |
| ENST00000322032 | ENST00000322032 | Ig alpha-2 chain C region. [Source:Uniprot/SWISSPROT;Acc:P01877] [ENST00000322032] | 1,8476 | 4,88E-04 | 7,90E-03 |
| RFESD | NM_173362 | Homo sapiens Rieske (Fe-S) domain containing (RFESD), mRNA [NM_173362] | 1,4613 | 4,89E-04 | 7,91E-03 |
| RNF5 | NM_006913 | Homo sapiens ring finger protein 5 (RNF5), mRNA [NM_006913] | 0,5808 | 4,89E-04 | 7,91E-03 |
| DPP3 | NM_130443 | Homo sapiens dipeptidyl-peptidase 3 (DPP3), transcript variant 2, mRNA [NM_130443] | 0,6298 | 4,92E-04 | 7,94E-03 |
| GRPEL2 | NM_152407 | Homo sapiens GrpE-like 2, mitochondrial (E. coli) (GRPEL2), nuclear gene encoding mitochondrial protein, mRNA [NM_152407] | 0,7828 | 4,93E-04 | 7,94E-03 |
| AGPAT3 | NM_020132 | Homo sapiens 1-acylglycerol-3-phosphate O-acyltransferase 3 (AGPAT3), transcript variant 1, mRNA [NM_020132] | 0,9202 | 4,94E-04 | 7,95E-03 |
| GSG2 | AK056691 | Homo sapiens cDNA FLJ32129 fis, clone PEBLM2000213, weakly similar to Mus musculus genes for integrin aM290, hapsin. [AK056691] | 0,9880 | 4,95E-04 | 7,95E-03 |
| IGHA1 | AK128476 | Homo sapiens cDNA FLJ46621 fis, clone TLUNG2001445, highly similar to Ig alpha-1 chain C region. [AK128476] | 1,3703 | 5,05E-04 | 8,09E-03 |
| WDR81 | NM_152348 | Homo sapiens WD repeat domain 81 (WDR81), mRNA [NM_152348] | 0,8456 | 5,09E-04 | 8,15E-03 |
| PDCL | NM_005388 | Homo sapiens phosducin-like (PDCL), mRNA [NM_005388] | 0,5954 | 5,17E-04 | 8,26E-03 |
| CIT | NM_007174 | Homo sapiens citron (rho-interacting, serine/threonine kinase 21) (CIT), mRNA [NM_007174] | 1,3232 | 5,19E-04 | 8,28E-03 |
| CTSL | NM_001912 | Homo sapiens cathepsin L (CTSL), transcript variant 1, mRNA [NM_001912] | 1,8382 | 5,31E-04 | 8,43E-03 |
| FOXM1 | NM_202002 | Homo sapiens forkhead box M1 (FOXM1), transcript variant 1, mRNA [NM_202002] | 0,5827 | 5,36E-04 | 8,47E-03 |
| WIPI2 | NM_015610 | Homo sapiens WD repeat domain, phosphoinositide interacting 2 (WIPI2), transcript variant 1, mRNA [NM_015610] | 0,7733 | 5,36E-04 | 8,47E-03 |
| C10orf61 | NM_015631 | Homo sapiens chromosome 10 open reading frame 61 (C10orf61), transcript variant 2, mRNA [NM_015631] | 0,8785 | 5,39E-04 | 8,50E-03 |
| ZRANB1 | ENST00000359653 | Zinc finger Ran-binding domain-containing protein 1 (Protein TRABID). [Source:Uniprot/SWISSPROT;Acc:Q9UGI0] [ENST00000359653] | 0,6138 | 5,39E-04 | 8,50E-03 |
| CCDC117 | NM_173510 | Homo sapiens coiled-coil domain containing 117 (CCDC117), mRNA [NM_173510] | 0,6790 | 5,42E-04 | 8,55E-03 |
| CTDSPL | NM_001008392 | Homo sapiens CTD (carboxy-terminal domain, RNA polymerase II, polypeptide A) small phosphatase-like (CTDSPL), transcript variant 1, mRNA [NM_001008392] | 0,5312 | 5,44E-04 | 8,56E-03 |
| AP2B1 | NM_001030006 | Homo sapiens adaptor-related protein complex 2, beta 1 subunit (AP2B1), transcript variant 1, mRNA [NM_001030006] | 0,5309 | 5,50E-04 | 8,62E-03 |
| STK25 | NM_006374 | Homo sapiens serine/threonine kinase 25 (STE20 homolog, yeast) (STK25), mRNA [NM_006374] | 0,4548 | 5,50E-04 | 8,62E-03 |
| MSTP9 | NR_002729 | Homo sapiens macrophage stimulating, pseudogene 9 (MSTP9) on chromosome 1 [NR_002729] | 1,0098 | 5,52E-04 | 8,63E-03 |
| RBM23 | NM_001077351 | Homo sapiens RNA binding motif protein 23 (RBM23), transcript variant 1, mRNA [NM_001077351] | 0,7327 | 5,52E-04 | 8,64E-03 |
| BC087732 | BC087732 | Homo sapiens cDNA clone IMAGE:6253289, **** WARNING: chimeric clone ****. [BC087732] | 0,8727 | 5,53E-04 | 8,64E-03 |
| IMP3 | NM_018285 | Homo sapiens IMP3, U3 small nucleolar ribonucleoprotein, homolog (yeast) (IMP3), mRNA [NM_018285] | 0,8649 | 5,58E-04 | 8,70E-03 |
| NCAPH | NM_015341 | Homo sapiens non-SMC condensin I complex, subunit H (NCAPH), mRNA [NM_015341] | 1,2830 | 5,59E-04 | 8,72E-03 |
| EIF4EBP2 | ENST00000373218 | Eukaryotic translation initiation factor 4E-binding protein 2 (4E-BP2) (eIF4E-binding protein 2). [Source:Uniprot/SWISSPROT;Acc:Q13542] [ENST00000373218] | 0,4921 | 5,67E-04 | 8,81E-03 |
| DAAM1 | NM_014992 | Homo sapiens dishevelled associated activator of morphogenesis 1 (DAAM1), mRNA [NM_014992] | 0,7498 | 5,69E-04 | 8,83E-03 |
| MAP3K7IP3 | NM_152787 | Homo sapiens mitogen-activated protein kinase kinase kinase 7 interacting protein 3 (MAP3K7IP3), mRNA [NM_152787] | 0,5668 | 5,73E-04 | 8,86E-03 |
| MOSPD1 | NM_019556 | Homo sapiens motile sperm domain containing 1 (MOSPD1), mRNA [NM_019556] | 0,8923 | 5,72E-04 | 8,86E-03 |
| ZNF23 | NM_145911 | Homo sapiens zinc finger protein 23 (KOX 16) (ZNF23), mRNA [NM_145911] | 0,6723 | 5,85E-04 | 9,01E-03 |
| A_24_P203886 | A_24_P203886 | Unknown | 0,9381 | 5,86E-04 | 9,02E-03 |
| TRAF3IP1 | BC059174 | Homo sapiens TNF receptor-associated factor 3 interacting protein 1, mRNA (cDNA clone MGC:54069 IMAGE:6191726), complete cds. [BC059174] | 0,6003 | 5,91E-04 | 9,07E-03 |
| KATNAL1 | NM_032116 | Homo sapiens katanin p60 subunit A-like 1 (KATNAL1), transcript variant 1, mRNA [NM_032116] | 0,9168 | 5,94E-04 | 9,12E-03 |
| MGC27348 | BC026177 | Homo sapiens ribosomal protein S2 pseudogene, mRNA (cDNA clone MGC:27348 IMAGE:4671259), complete cds. [BC026177] | 0,7018 | 6,07E-04 | 9,28E-03 |
| PRKCBP1 | AL137703 | Homo sapiens mRNA; cDNA DKFZp564P1772 (from clone DKFZp564P1772). [AL137703] | 0,6890 | 6,10E-04 | 9,31E-03 |
| TXNDC5 | NM_022085 | Homo sapiens thioredoxin domain containing 5 (TXNDC5), transcript variant 2, mRNA [NM_022085] | 1,3088 | 6,19E-04 | 9,40E-03 |
| TUBA8 | NM_018943 | Homo sapiens tubulin, alpha 8 (TUBA8), mRNA [NM_018943] | 0,8818 | 6,26E-04 | 9,50E-03 |
| E2F4 | NM_001950 | Homo sapiens E2F transcription factor 4, p107/p130-binding (E2F4), mRNA [NM_001950] | 0,9278 | 6,28E-04 | 9,53E-03 |
| ZNF672 | NM_024836 | Homo sapiens zinc finger protein 672 (ZNF672), mRNA [NM_024836] | 0,8527 | 6,45E-04 | 9,76E-03 |
| TLOC1 | NM_003262 | Homo sapiens translocation protein 1 (TLOC1), mRNA [NM_003262] | 0,8770 | 6,50E-04 | 9,82E-03 |
| AGPAT4 | NM_020133 | Homo sapiens 1-acylglycerol-3-phosphate O-acyltransferase 4 (lysophosphatidic acid acyltransferase, delta) (AGPAT4), mRNA [NM_020133] | 0,9842 | 6,54E-04 | 9,88E-03 |

**Supplementary Table 1: B)** Genes significantly (Bonferroni’s adjusted P value < 0.01) under-expressed in BM resident cells from patients with localized NB as compared to healthy children.

| **Gene name** | **Systematic name** | **Description** | **Log fold change** | **P value** | **Adjusted P value** |
| --- | --- | --- | --- | --- | --- |
| AQP1 | NM_198098 | Homo sapiens aquaporin 1 (Colton blood group) (AQP1), mRNA [NM_198098] | 3,4704 | 1,99E-13 | 3,62E-10 |
| ENST00000374390 | ENST00000374390 | cellular modulator of immune recognition isoform 7 [Source:RefSeq_peptide;Acc:NP_001002266] [ENST00000374390] | 2,7739 | 4,82E-14 | 3,62E-10 |
| EPB49 | NM_001978 | Homo sapiens erythrocyte membrane protein band 4.9 (dematin) (EPB49), mRNA [NM_001978] | 2,4059 | 2,49E-14 | 3,62E-10 |
| FECH | NM_001012515 | Homo sapiens ferrochelatase (protoporphyria) (FECH), nuclear gene encoding mitochondrial protein, transcript variant 1, mRNA [NM_001012515] | 3,2118 | 7,40E-14 | 3,62E-10 |
| PLEK2 | NM_016445 | Homo sapiens pleckstrin 2 (PLEK2), mRNA [NM_016445] | 2,9526 | 1,18E-13 | 3,62E-10 |
| SLC6A10P | NR_003083 | Homo sapiens solute carrier family 6 (neurotransmitter transporter, creatine), member 10 (pseudogene) (SLC6A10P) on chromosome 16 [NR_003083] | 2,6645 | 1,77E-13 | 3,62E-10 |
| SNCA | NM_007308 | Homo sapiens synuclein, alpha (non A4 component of amyloid precursor) (SNCA), transcript variant NACP112, mRNA [NM_007308] | 3,8899 | 2,13E-13 | 3,62E-10 |
| THC2613527 | THC2613527 | Unknown | 2,2322 | 1,39E-13 | 3,62E-10 |
| TMCC2 | NM_014858 | Homo sapiens transmembrane and coiled-coil domain family 2 (TMCC2), mRNA [NM_014858] | 4,5099 | 2,54E-13 | 3,80E-10 |
| TRIM58 | NM_015431 | Homo sapiens tripartite motif-containing 58 (TRIM58), mRNA [NM_015431] | 2,9495 | 2,62E-13 | 3,80E-10 |
| THC2638232 | THC2638232 | Q7RQ28_PLAYO (Q7RQ28) Nuclear protein snf7, partial (7%) [THC2638232] | 2,3973 | 4,21E-13 | 5,03E-10 |
| KIAA1727 | NM_033393 | Homo sapiens KIAA1727 protein (KIAA1727), mRNA [NM_033393] | 2,0590 | 8,43E-13 | 8,58E-10 |
| PDZK1IP1 | NM_005764 | Homo sapiens PDZK1 interacting protein 1 (PDZK1IP1), mRNA [NM_005764] | 3,2945 | 8,30E-13 | 8,58E-10 |
| A_32_P78285 | A_32_P78285 | Unknown | 2,8195 | 9,46E-13 | 8,75E-10 |
| GMPR | NM_006877 | Homo sapiens guanosine monophosphate reductase (GMPR), mRNA [NM_006877] | 2,7326 | 9,09E-13 | 8,75E-10 |
| KRT1 | NM_006121 | Homo sapiens keratin 1 (epidermolytic hyperkeratosis) (KRT1), mRNA [NM_006121] | 4,5048 | 1,41E-12 | 1,22E-09 |
| THC2478531 | THC2478531 | Unknown | 4,3306 | 1,44E-12 | 1,22E-09 |
| BX537432 | BX537432 | Homo sapiens mRNA; cDNA DKFZp686N09198 (from clone DKFZp686N09198); complete cds. [BX537432] | 3,4026 | 1,59E-12 | 1,28E-09 |
| SELENBP1 | NM_003944 | Homo sapiens selenium binding protein 1 (SELENBP1), mRNA [NM_003944] | 2,7214 | 1,75E-12 | 1,28E-09 |
| SLC25A39 | NM_016016 | Homo sapiens solute carrier family 25, member 39 (SLC25A39), mRNA [NM_016016] | 3,2218 | 1,77E-12 | 1,28E-09 |
| EPB42 | NM_000119 | Homo sapiens erythrocyte membrane protein band 4.2 (EPB42), mRNA [NM_000119] | 3,2701 | 2,20E-12 | 1,54E-09 |
| TNS1 | NM_022648 | Homo sapiens tensin 1 (TNS1), mRNA [NM_022648] | 2,8297 | 2,45E-12 | 1,66E-09 |
| RILP | NM_031430 | Homo sapiens Rab interacting lysosomal protein (RILP), mRNA [NM_031430] | 2,0699 | 2,79E-12 | 1,83E-09 |
| GPR146 | NM_138445 | Homo sapiens G protein-coupled receptor 146 (GPR146), mRNA [NM_138445] | 2,0499 | 3,13E-12 | 1,99E-09 |
| ACSL6 | NM_001009185 | Homo sapiens acyl-CoA synthetase long-chain family member 6 (ACSL6), transcript variant 2, mRNA [NM_001009185] | 3,0454 | 7,41E-12 | 4,57E-09 |
| BCL2L1 | NM_138578 | Homo sapiens BCL2-like 1 (BCL2L1), nuclear gene encoding mitochondrial protein, transcript variant 1, mRNA [NM_138578] | 2,3419 | 1,12E-11 | 6,31E-09 |
| TRAK2 | NM_015049 | Homo sapiens trafficking protein, kinesin binding 2 (TRAK2), mRNA [NM_015049] | 2,0748 | 1,18E-11 | 6,34E-09 |
| SLC6A8 | NM_005629 | Homo sapiens solute carrier family 6 (neurotransmitter transporter, creatine), member 8 (SLC6A8), mRNA [NM_005629] | 2,0922 | 1,34E-11 | 6,97E-09 |
| FAM46C | NM_017709 | Homo sapiens family with sequence similarity 46, member C (FAM46C), mRNA [NM_017709] | 2,6409 | 1,49E-11 | 7,59E-09 |
| HMBS | NM_000190 | Homo sapiens hydroxymethylbilane synthase (HMBS), transcript variant 1, mRNA [NM_000190] | 2,4209 | 1,87E-11 | 8,69E-09 |
| WDR40A | NM_015397 | Homo sapiens WD repeat domain 40A (WDR40A), mRNA [NM_015397] | 2,4097 | 1,88E-11 | 8,69E-09 |
| XK | NM_021083 | Homo sapiens X-linked Kx blood group (McLeod syndrome) (XK), mRNA [NM_021083] | 2,3511 | 1,86E-11 | 8,69E-09 |
| SLC4A1 | NM_000342 | Homo sapiens solute carrier family 4, anion exchanger, member 1 (erythrocyte membrane protein band 3, Diego blood group) (SLC4A1), mRNA [NM_000342] | 2,6975 | 2,11E-11 | 9,53E-09 |
| HAGH | NM_001040427 | Homo sapiens hydroxyacylglutathione hydrolase (HAGH), transcript variant 2, mRNA [NM_001040427] | 1,9623 | 2,24E-11 | 9,79E-09 |
| MICALCL | NM_032867 | Homo sapiens MICAL C-terminal like (MICALCL), mRNA [NM_032867] | 1,8649 | 2,41E-11 | 1,02E-08 |
| A_24_P147849 | A_24_P147849 | Unknown | 5,3214 | 2,51E-11 | 1,04E-08 |
| EFNB3 | NM_001406 | Homo sapiens ephrin-B3 (EFNB3), mRNA [NM_001406] | 4,5700 | 2,74E-11 | 1,11E-08 |
| PAQR9 | NM_198504 | Homo sapiens progestin and adipoQ receptor family member IX (PAQR9), mRNA [NM_198504] | 2,5164 | 2,78E-11 | 1,11E-08 |
| THC2669092 | THC2669092 | Unknown | 3,6047 | 2,96E-11 | 1,14E-08 |
| MICAL2 | NM_014632 | Homo sapiens microtubule associated monoxygenase, calponin and LIM domain containing 2 (MICAL2), mRNA [NM_014632] | 2,8737 | 3,02E-11 | 1,14E-08 |
| CCRL2 | NM_003965 | Homo sapiens chemokine (C-C motif) receptor-like 2 (CCRL2), mRNA [NM_003965] | 2,1117 | 3,34E-11 | 1,24E-08 |
| SLC14A1 | ENST00000321925 | Urea transporter, erythrocyte. [Source:Uniprot/SWISSPROT;Acc:Q13336] [ENST00000321925] | 3,1232 | 3,61E-11 | 1,31E-08 |
| BPGM | NM_199186 | Homo sapiens 2,3-bisphosphoglycerate mutase (BPGM), transcript variant 2, mRNA [NM_199186] | 3,5542 | 4,88E-11 | 1,68E-08 |
| OSBP2 | NM_030758 | Homo sapiens oxysterol binding protein 2 (OSBP2), transcript variant 1, mRNA [NM_030758] | 2,1387 | 4,88E-11 | 1,68E-08 |
| HBQ1 | NM_005331 | Homo sapiens hemoglobin, theta 1 (HBQ1), mRNA [NM_005331] | 4,1379 | 5,16E-11 | 1,72E-08 |
| TBCEL | BC020501 | Homo sapiens leucine rich repeat containing 35, mRNA (cDNA clone IMAGE:3913004). [BC020501] | 1,8901 | 5,17E-11 | 1,72E-08 |
| AY358510 | AY358510 | Homo sapiens clone DNA57836 GLPG464 (UNQ464) mRNA, complete cds. [AY358510] | 1,5642 | 6,86E-11 | 2,15E-08 |
| TSPAN5 | NM_005723 | Homo sapiens tetraspanin 5 (TSPAN5), mRNA [NM_005723] | 2,6356 | 7,80E-11 | 2,40E-08 |
| ALAS2 | NM_000032 | Homo sapiens aminolevulinate, delta-, synthase 2 (sideroblastic/hypochromic anemia) (ALAS2), nuclear gene encoding mitochondrial protein, transcript variant 1, mRNA [NM_000032] | 3,2667 | 8,08E-11 | 2,42E-08 |
| NFIX | NM_002501 | Homo sapiens nuclear factor I/X (CCAAT-binding transcription factor) (NFIX), mRNA [NM_002501] | 2,6486 | 8,00E-11 | 2,42E-08 |
| GYPB | NM_002100 | Homo sapiens glycophorin B (MNS blood group) (GYPB), mRNA [NM_002100] | 2,4558 | 9,44E-11 | 2,74E-08 |
| A_23_P84791 | A_23_P84791 | Unknown | 2,3245 | 9,99E-11 | 2,76E-08 |
| ASCC2 | NM_032204 | Homo sapiens activating signal cointegrator 1 complex subunit 2 (ASCC2), mRNA [NM_032204] | 1,2199 | 9,99E-11 | 2,76E-08 |
| C5orf4 | NM_032385 | Homo sapiens chromosome 5 open reading frame 4 (C5orf4), transcript variant 2, mRNA [NM_032385] | 3,0390 | 9,83E-11 | 2,76E-08 |
| THC2654231 | THC2654231 | ALU5_HUMAN (P39192) Alu subfamily SC sequence contamination warning entry, partial (8%) [THC2654231] | 1,9906 | 1,00E-10 | 2,76E-08 |
| KLF1 | NM_006563 | Homo sapiens Kruppel-like factor 1 (erythroid) (KLF1), mRNA [NM_006563] | 1,9418 | 1,15E-10 | 3,08E-08 |
| RHCE | NM_020485 | Homo sapiens Rh blood group, CcEe antigens (RHCE), transcript variant 1, mRNA [NM_020485] | 3,1804 | 1,16E-10 | 3,08E-08 |
| GYPC | NM_002101 | Homo sapiens glycophorin C (Gerbich blood group) (GYPC), transcript variant 1, mRNA [NM_002101] | 2,0632 | 1,21E-10 | 3,16E-08 |
| CD709370 | CD709370 | CD709370 EST25897 human nasopharynx Homo sapiens cDNA, mRNA sequence [CD709370] | 1,8278 | 1,26E-10 | 3,24E-08 |
| FLJ41603 | NM_001001669 | Homo sapiens FLJ41603 protein (FLJ41603), mRNA [NM_001001669] | 2,0181 | 1,38E-10 | 3,47E-08 |
| SLC2A1 | NM_006516 | Homo sapiens solute carrier family 2 (facilitated glucose transporter), member 1 (SLC2A1), mRNA [NM_006516] | 2,8287 | 1,37E-10 | 3,47E-08 |
| DPM2 | NM_003863 | Homo sapiens dolichyl-phosphate mannosyltransferase polypeptide 2, regulatory subunit (DPM2), mRNA [NM_003863] | 1,6839 | 1,49E-10 | 3,69E-08 |
| GLRX5 | NM_016417 | Homo sapiens glutaredoxin 5 homolog (S. cerevisiae) (GLRX5), mRNA [NM_016417] | 1,8999 | 1,51E-10 | 3,70E-08 |
| KLHDC8A | NM_018203 | Homo sapiens kelch domain containing 8A (KLHDC8A), mRNA [NM_018203] | 1,8164 | 1,57E-10 | 3,75E-08 |
| THC2588392 | THC2588392 | 1ABW_A Chain A, Deoxy Rhb1.1 (Recombinant Hemoglobin). {synthetic construct} (exp=-1; wgp=-1; cg=-1), partial (18%) [THC2588392] | 4,5668 | 1,56E-10 | 3,75E-08 |
| FBXO7 | NM_012179 | Homo sapiens F-box protein 7 (FBXO7), transcript variant 1, mRNA [NM_012179] | 2,0097 | 2,53E-10 | 5,66E-08 |
| SLC7A5 | NM_003486 | Homo sapiens solute carrier family 7 (cationic amino acid transporter, y+ system), member 5 (SLC7A5), mRNA [NM_003486] | 2,5941 | 2,77E-10 | 6,13E-08 |
| KIF26A | BC009415 | Homo sapiens kinesin family member 26A, mRNA (cDNA clone IMAGE:3502885), complete cds. [BC009415] | 2,1443 | 3,02E-10 | 6,53E-08 |
| ST6GALNAC4 | NM_175039 | Homo sapiens ST6 (alpha-N-acetyl-neuraminyl-2,3-beta-galactosyl-1,3)-N-acetylgalactosaminide alpha-2,6-sialyltransferase 4 (ST6GALNAC4), transcript variant 1, mRNA [NM_175039] | 1,4607 | 3,20E-10 | 6,78E-08 |
| ALS2CR2 | NM_018571 | Homo sapiens amyotrophic lateral sclerosis 2 (juvenile) chromosome region, candidate 2 (ALS2CR2), mRNA [NM_018571] | 2,4856 | 4,18E-10 | 8,67E-08 |
| THC2656519 | THC2656519 | Unknown | 1,9520 | 4,15E-10 | 8,67E-08 |
| RNF123 | NM_022064 | Homo sapiens ring finger protein 123 (RNF123), mRNA [NM_022064] | 1,7893 | 4,29E-10 | 8,82E-08 |
| TUBG1 | NM_001070 | Homo sapiens tubulin, gamma 1 (TUBG1), mRNA [NM_001070] | 1,7024 | 4,69E-10 | 9,54E-08 |
| CTSL | NM_001912 | Homo sapiens cathepsin L (CTSL), transcript variant 1, mRNA [NM_001912] | 2,1491 | 5,77E-10 | 1,15E-07 |
| ITLN1 | NM_017625 | Homo sapiens intelectin 1 (galactofuranose binding) (ITLN1), mRNA [NM_017625] | 2,5476 | 6,06E-10 | 1,20E-07 |
| BC012876 | BC012876 | Homo sapiens cDNA clone MGC:17259 IMAGE:4149333, complete cds. [BC012876] | 2,6853 | 6,17E-10 | 1,21E-07 |
| ENDOD1 | ENST00000278505 | Endonuclease domain-containing 1 protein precursor (EC 3.1.30.-). [Source:Uniprot/SWISSPROT;Acc:O94919] [ENST00000278505] | 1,6593 | 6,41E-10 | 1,23E-07 |
| PRDX2 | NM_005809 | Homo sapiens peroxiredoxin 2 (PRDX2), nuclear gene encoding mitochondrial protein, transcript variant 1, mRNA [NM_005809] | 2,0099 | 6,86E-10 | 1,29E-07 |
| GAS2L1 | NM_152237 | Homo sapiens growth arrest-specific 2 like 1 (GAS2L1), transcript variant 3, mRNA [NM_152237] | 1,9043 | 6,95E-10 | 1,30E-07 |
| FAM117A | NM_030802 | Homo sapiens family with sequence similarity 117, member A (FAM117A), mRNA [NM_030802] | 1,7501 | 7,69E-10 | 1,40E-07 |
| HBM | NM_001003938 | Homo sapiens hemoglobin, mu (HBM), mRNA [NM_001003938] | 3,3097 | 9,71E-10 | 1,73E-07 |
| AQP3 | NM_004925 | Homo sapiens aquaporin 3 (Gill blood group) (AQP3), mRNA [NM_004925] | 1,9168 | 1,04E-09 | 1,84E-07 |
| BSG | NM_001728 | Homo sapiens basigin (Ok blood group) (BSG), transcript variant 1, mRNA [NM_001728] | 1,6837 | 1,06E-09 | 1,87E-07 |
| PTPRF | NM_002840 | Homo sapiens protein tyrosine phosphatase, receptor type, F (PTPRF), transcript variant 1, mRNA [NM_002840] | 2,0072 | 1,10E-09 | 1,91E-07 |
| THC2633438 | THC2633438 | ALU5_HUMAN (P39192) Alu subfamily SC sequence contamination warning entry, partial (9%) [THC2633438] | 2,0757 | 1,12E-09 | 1,91E-07 |
| NT5M | NM_020201 | Homo sapiens 5',3'-nucleotidase, mitochondrial (NT5M), nuclear gene encoding mitochondrial protein, mRNA [NM_020201] | 1,2632 | 1,14E-09 | 1,93E-07 |
| ERMAP | NM_001017922 | Homo sapiens erythroblast membrane-associated protein (Scianna blood group) (ERMAP), transcript variant 1, mRNA [NM_001017922] | 1,9481 | 1,16E-09 | 1,94E-07 |
| UROD | NM_000374 | Homo sapiens uroporphyrinogen decarboxylase (UROD), mRNA [NM_000374] | 1,6694 | 1,15E-09 | 1,94E-07 |
| THC2688497 | THC2688497 | Q59GX2_HUMAN (Q59GX2) Solute carrier family 2 (Facilitated glucose transporter), member 1 variant (Fragment), partial (6%) [THC2564899] | 2,5973 | 1,23E-09 | 2,03E-07 |
| IL8 | X77737 | H.sapiens mRNA for red cell anion exchanger (EPB3, AE1, Band 3) 3' non-coding region. [X77737] | 3,0830 | 1,27E-09 | 2,08E-07 |
| ENST00000379913 | ENST00000379913 | Ig gamma-4 chain C region. [Source:Uniprot/SWISSPROT;Acc:P01861] [ENST00000379913] | 4,3388 | 1,33E-09 | 2,14E-07 |
| HEMGN | NM_018437 | Homo sapiens hemogen (HEMGN), transcript variant 1, mRNA [NM_018437] | 2,9927 | 1,34E-09 | 2,14E-07 |
| LOC283177 | AK095081 | Homo sapiens cDNA FLJ37762 fis, clone BRHIP2024347, weakly similar to GALECTIN-3. [AK095081] | 3,2710 | 1,34E-09 | 2,14E-07 |
| LOC644462 | XM_930312 | PREDICTED: Homo sapiens similar to amyotrophic lateral sclerosis 2 (juvenile) chromosome region, candidate 2 (LOC644462), mRNA [XM_930312] | 2,3047 | 1,42E-09 | 2,23E-07 |
| ARHGEF12 | NM_015313 | Homo sapiens Rho guanine nucleotide exchange factor (GEF) 12 (ARHGEF12), mRNA [NM_015313] | 1,5923 | 1,62E-09 | 2,48E-07 |
| XPO7 | NM_015024 | Homo sapiens exportin 7 (XPO7), mRNA [NM_015024] | 1,8471 | 1,61E-09 | 2,48E-07 |
| AY998685 | AY998685 | Homo sapiens isolate 13K immunoglobulin kappa light chain variable region (IGKV4) mRNA, IGKV4-1*01 allele, partial cds. [AY998685] | 1,6311 | 1,66E-09 | 2,48E-07 |
| C1orf198 | NM_032800 | Homo sapiens chromosome 1 open reading frame 198 (C1orf198), mRNA [NM_032800] | 1,4508 | 1,64E-09 | 2,48E-07 |
| NP | NM_000270 | Homo sapiens nucleoside phosphorylase (NP), mRNA [NM_000270] | 1,3036 | 1,65E-09 | 2,48E-07 |
| TMEM15 | NM_014908 | Homo sapiens transmembrane protein 15 (TMEM15), mRNA [NM_014908] | 1,1305 | 1,68E-09 | 2,50E-07 |
| ENST00000283657 | ENST00000283657 | V kappa 4=immunoglobulin light chain variable region {complementarity determining regions} [human, CD5+ tonsillar B cells, mRNA PartialMutant, 303 nt]. [S62210] | 2,6285 | 1,72E-09 | 2,54E-07 |
| LOC442239 | XR_018980 | PREDICTED: Homo sapiens similar to Peroxiredoxin-2 (Thioredoxin peroxidase 1) (Thioredoxin-dependent peroxide reductase 1) (Thiol-specific antioxidant protein) (TSA) (PRP) (Natural killer cell-enhancing factor B) (NKEF-B) (LOC442239), mRNA [XR_018980] | 1,8944 | 1,88E-09 | 2,74E-07 |
| PHLPPL | NM_015020 | Homo sapiens PH domain and leucine rich repeat protein phosphatase-like (PHLPPL), mRNA [NM_015020] | 1,5514 | 1,95E-09 | 2,80E-07 |
| S76132 | S76132 | Ig V lambda II=IgG rheumatoid factor [human, hybridoma AEE111F, mRNA Partial, 315 nt]. [S76132] | 2,6734 | 2,02E-09 | 2,88E-07 |
| BLVRB | NM_000713 | Homo sapiens biliverdin reductase B (flavin reductase (NADPH)) (BLVRB), mRNA [NM_000713] | 2,0523 | 2,22E-09 | 3,11E-07 |
| RHAG | NM_000324 | Homo sapiens Rh-associated glycoprotein (RHAG), mRNA [NM_000324] | 2,5343 | 2,36E-09 | 3,29E-07 |
| A_24_P401150 | A_24_P401150 | Unknown | 2,1978 | 2,39E-09 | 3,31E-07 |
| ENST00000331195 | ENST00000331195 | V1-16 protein (Fragment). [Source:Uniprot/SPTREMBL;Acc:Q5NV81] [ENST00000331195] | 2,2998 | 2,44E-09 | 3,32E-07 |
| GATA1 | NM_002049 | Homo sapiens GATA binding protein 1 (globin transcription factor 1) (GATA1), mRNA [NM_002049] | 1,6537 | 2,56E-09 | 3,41E-07 |
| PRR5 | NM_015366 | Homo sapiens proline rich 5 (renal) (PRR5), transcript variant 2, mRNA [NM_015366] | 1,7805 | 2,55E-09 | 3,41E-07 |
| STEAP3 | NM_182915 | Homo sapiens STEAP family member 3 (STEAP3), transcript variant 1, mRNA [NM_182915] | 1,1728 | 2,93E-09 | 3,83E-07 |
| YPEL4 | NM_145008 | Homo sapiens yippee-like 4 (Drosophila) (YPEL4), mRNA [NM_145008] | 1,8502 | 2,94E-09 | 3,83E-07 |
| CR603982 | CR603982 | full-length cDNA clone CS0DF021YL03 of Fetal brain of Homo sapiens (human). [CR603982] | 1,1111 | 3,09E-09 | 3,96E-07 |
| ANK1 | NM_000037 | Homo sapiens ankyrin 1, erythrocytic (ANK1), transcript variant 3, mRNA [NM_000037] | 2,4776 | 3,16E-09 | 4,02E-07 |
| EIF2AK1 | NM_014413 | Homo sapiens eukaryotic translation initiation factor 2-alpha kinase 1 (EIF2AK1), mRNA [NM_014413] | 1,9142 | 3,19E-09 | 4,03E-07 |
| OPTN | NM_001008211 | Homo sapiens optineurin (OPTN), transcript variant 1, mRNA [NM_001008211] | 2,2207 | 3,24E-09 | 4,07E-07 |
| A_32_P208713 | A_32_P208713 | Unknown | 3,1275 | 3,68E-09 | 4,59E-07 |
| TNXB | NM_032470 | Homo sapiens tenascin XB (TNXB), transcript variant XB-S, mRNA [NM_032470] | 2,2772 | 3,75E-09 | 4,63E-07 |
| TMPRSS9 | AK131261 | Homo sapiens cDNA FLJ16193 fis, clone BRTHA2018011, weakly similar to EPITHIN (EC 3.4.21.-). [AK131261] | 1,5718 | 3,91E-09 | 4,79E-07 |
| ABCG2 | NM_004827 | Homo sapiens ATP-binding cassette, sub-family G (WHITE), member 2 (ABCG2), mRNA [NM_004827] | 1,8927 | 3,93E-09 | 4,79E-07 |
| A_24_P25020 | A_24_P25020 | Unknown | 1,6935 | 4,18E-09 | 5,06E-07 |
| ANKRD41 | NM_152363 | Homo sapiens ankyrin repeat domain 41 (ANKRD41), mRNA [NM_152363] | 1,6983 | 4,43E-09 | 5,33E-07 |
| ENST00000322032 | ENST00000322032 | Ig alpha-2 chain C region. [Source:Uniprot/SWISSPROT;Acc:P01877] [ENST00000322032] | 3,0843 | 4,65E-09 | 5,56E-07 |
| GFI1B | NM_004188 | Homo sapiens growth factor independent 1B (potential regulator of CDKN1A, translocated in CML) (GFI1B), mRNA [NM_004188] | 1,1417 | 5,10E-09 | 6,00E-07 |
| CXCR6 | NM_006564 | Homo sapiens chemokine (C-X-C motif) receptor 6 (CXCR6), mRNA [NM_006564] | 1,0983 | 5,17E-09 | 6,04E-07 |
| DDB1 | NM_001923 | Homo sapiens damage-specific DNA binding protein 1, 127kDa (DDB1), mRNA [NM_001923] | 1,1122 | 5,35E-09 | 6,15E-07 |
| KEL | NM_000420 | Homo sapiens Kell blood group, metallo-endopeptidase (KEL), mRNA [NM_000420] | 1,7940 | 5,35E-09 | 6,15E-07 |
| C16orf35 | NM_001039476 | Homo sapiens chromosome 16 open reading frame 35 (C16orf35), transcript variant 2, mRNA [NM_001039476] | 1,4941 | 6,02E-09 | 6,77E-07 |
| ERAF | NM_016633 | Homo sapiens erythroid associated factor (ERAF), mRNA [NM_016633] | 2,2992 | 6,32E-09 | 6,99E-07 |
| SPTA1 | NM_003126 | Homo sapiens spectrin, alpha, erythrocytic 1 (elliptocytosis 2) (SPTA1), mRNA [NM_003126] | 2,2887 | 6,52E-09 | 7,17E-07 |
| AF332145 | AF332145 | Homo sapiens anti-pneumococcal antibody NAD light chain variable region mRNA, partial cds. [AF332145] | 1,7836 | 6,60E-09 | 7,18E-07 |
| SIPA1L1 | CR936651 | Homo sapiens mRNA; cDNA DKFZp686G1344 (from clone DKFZp686G1344). [CR936651] | 2,2678 | 6,79E-09 | 7,35E-07 |
| CA2 | NM_000067 | Homo sapiens carbonic anhydrase II (CA2), mRNA [NM_000067] | 2,8171 | 6,91E-09 | 7,44E-07 |
| SLC1A5 | NM_005628 | Homo sapiens solute carrier family 1 (neutral amino acid transporter), member 5 (SLC1A5), mRNA [NM_005628] | 1,7935 | 7,25E-09 | 7,72E-07 |
| UBADC1 | NM_016172 | Homo sapiens ubiquitin associated domain containing 1 (UBADC1), mRNA [NM_016172] | 1,3550 | 7,24E-09 | 7,72E-07 |
| IGLV6-57 | BC023973 | Homo sapiens immunoglobulin lambda variable 6-57, mRNA (cDNA clone MGC:34845 IMAGE:5223747), complete cds. [BC023973] | 2,1065 | 7,82E-09 | 8,26E-07 |
| TUBG2 | NM_016437 | Homo sapiens tubulin, gamma 2 (TUBG2), mRNA [NM_016437] | 1,6080 | 7,84E-09 | 8,26E-07 |
| EPOR | NM_000121 | Homo sapiens erythropoietin receptor (EPOR), mRNA [NM_000121] | 1,6388 | 7,99E-09 | 8,38E-07 |
| ANKRD9 | NM_152326 | Homo sapiens ankyrin repeat domain 9 (ANKRD9), mRNA [NM_152326] | 2,1747 | 8,09E-09 | 8,45E-07 |
| MCM2 | NM_004526 | Homo sapiens MCM2 minichromosome maintenance deficient 2, mitotin (S. cerevisiae) (MCM2), mRNA [NM_004526] | 1,1458 | 8,48E-09 | 8,63E-07 |
| A_32_P93894 | A_32_P93894 | Unknown | 0,9911 | 9,09E-09 | 9,17E-07 |
| IGKV1-5 | BC034142 | Homo sapiens immunoglobulin kappa variable 1-5, mRNA (cDNA clone MGC:32715 IMAGE:4694346), complete cds. [BC034142] | 2,0017 | 9,30E-09 | 9,27E-07 |
| C9orf114 | NM_016390 | Homo sapiens chromosome 9 open reading frame 114 (C9orf114), mRNA [NM_016390] | 1,0362 | 1,02E-08 | 9,98E-07 |
| RNF187 | BC012758 | Homo sapiens ring finger protein 187, mRNA (cDNA clone IMAGE:3633225), partial cds. [BC012758] | 1,3380 | 1,03E-08 | 9,98E-07 |
| IFRD2 | NM_006764 | Homo sapiens interferon-related developmental regulator 2 (IFRD2), mRNA [NM_006764] | 1,1892 | 1,14E-08 | 1,08E-06 |
| MOBKL1A | NM_173468 | Homo sapiens MOB1, Mps One Binder kinase activator-like 1A (yeast) (MOBKL1A), mRNA [NM_173468] | 1,8448 | 1,14E-08 | 1,08E-06 |
| TTC25 | NM_031421 | Homo sapiens tetratricopeptide repeat domain 25 (TTC25), mRNA [NM_031421] | 2,5449 | 1,18E-08 | 1,11E-06 |
| IGKV2-24 | BC063599 | Homo sapiens immunoglobulin kappa variable 2-24, mRNA (cDNA clone MGC:75493 IMAGE:4776392), complete cds. [BC063599] | 1,2421 | 1,23E-08 | 1,15E-06 |
| TXNDC5 | NM_022085 | Homo sapiens thioredoxin domain containing 5 (TXNDC5), transcript variant 2, mRNA [NM_022085] | 1,5690 | 1,32E-08 | 1,21E-06 |
| A_24_P152315 | A_24_P152315 | Unknown | 1,1358 | 1,33E-08 | 1,21E-06 |
| ATP1B2 | NM_001678 | Homo sapiens ATPase, Na+/K+ transporting, beta 2 polypeptide (ATP1B2), mRNA [NM_001678] | 1,9768 | 1,44E-08 | 1,29E-06 |
| GCLC | M90656 | Human gamma-glutamylcysteine synthetase (GCS) mRNA, complete cds. [M90656] | 1,5461 | 1,51E-08 | 1,35E-06 |
| ENST00000370857 | ENST00000370857 | Muscleblind-like X-linked protein (Muscleblind-like protein 3) (Cys3His CCG1-required protein) (Protein HCHCR). [Source:Uniprot/SWISSPROT;Acc:Q9NUK0] [ENST00000370857] | 1,4317 | 1,54E-08 | 1,37E-06 |
| WDR23 | NM_025230 | Homo sapiens WD repeat domain 23 (WDR23), transcript variant 1, mRNA [NM_025230] | 1,2373 | 1,62E-08 | 1,43E-06 |
| ANKH | NM_054027 | Homo sapiens ankylosis, progressive homolog (mouse) (ANKH), mRNA [NM_054027] | 1,2364 | 1,67E-08 | 1,47E-06 |
| AADACL1 | NM_020792 | Homo sapiens arylacetamide deacetylase-like 1 (AADACL1), mRNA [NM_020792] | 1,8711 | 1,84E-08 | 1,59E-06 |
| ENST00000312946 | ENST00000312946 | AY320849 immunoglobulin kappa chain variable region {Homo sapiens} (exp=-1; wgp=0; cg=0), complete [THC2568849] | 1,5792 | 1,83E-08 | 1,59E-06 |
| A_24_P298179 | A_24_P298179 | Unknown | 1,5640 | 1,91E-08 | 1,65E-06 |
| RCL1 | NM_005772 | Homo sapiens RNA terminal phosphate cyclase-like 1 (RCL1), mRNA [NM_005772] | 1,2420 | 1,97E-08 | 1,69E-06 |
| TSPAN17 | NM_012171 | Homo sapiens tetraspanin 17 (TSPAN17), transcript variant 1, mRNA [NM_012171] | 1,3688 | 2,05E-08 | 1,75E-06 |
| C20orf108 | NM_080821 | Homo sapiens chromosome 20 open reading frame 108 (C20orf108), mRNA [NM_080821] | 2,1570 | 2,16E-08 | 1,83E-06 |
| RPUSD2 | NM_152260 | Homo sapiens RNA pseudouridylate synthase domain containing 2 (RPUSD2), mRNA [NM_152260] | 1,3116 | 2,21E-08 | 1,85E-06 |
| M87790 | M87790 | Human (hybridoma H210) anti-hepatitis A immunoglobulin lambda chain variable region, constant region, complementarity-determining regions mRNA, complete cds. [M87790] | 1,8915 | 2,23E-08 | 1,85E-06 |
| CLTC | NM_004859 | Homo sapiens clathrin, heavy chain (Hc) (CLTC), mRNA [NM_004859] | 1,0662 | 2,35E-08 | 1,95E-06 |
| PGRMC2 | NM_006320 | Homo sapiens progesterone receptor membrane component 2 (PGRMC2), mRNA [NM_006320] | 1,0782 | 2,47E-08 | 2,03E-06 |
| ENST00000359488 | ENST00000359488 | Ig kappa chain V-I region Walker precursor. [Source:Uniprot/SWISSPROT;Acc:P04431] [ENST00000359488] | 2,3195 | 2,56E-08 | 2,08E-06 |
| HBA2 | NM_000517 | Homo sapiens hemoglobin, alpha 2 (HBA2), mRNA [NM_000517] | 2,1393 | 2,55E-08 | 2,08E-06 |
| CHST2 | NM_004267 | Homo sapiens carbohydrate (N-acetylglucosamine-6-O) sulfotransferase 2 (CHST2), mRNA [NM_004267] | 1,9125 | 2,61E-08 | 2,11E-06 |
| GYPA | NM_002099 | Homo sapiens glycophorin A (MNS blood group) (GYPA), mRNA [NM_002099] | 1,7910 | 2,65E-08 | 2,13E-06 |
| SMOX | NM_175839 | Homo sapiens spermine oxidase (SMOX), transcript variant 1, mRNA [NM_175839] | 2,2912 | 2,76E-08 | 2,21E-06 |
| ART4 | NM_021071 | Homo sapiens ADP-ribosyltransferase 4 (Dombrock blood group) (ART4), mRNA [NM_021071] | 2,2326 | 2,92E-08 | 2,31E-06 |
| AL522622 | AL522622 | AL522622 AL522622 Homo sapiens NEUROBLASTOMA COT 10-NORMALIZED Homo sapiens cDNA clone CS0DB009YE01 3-PRIME, mRNA sequence [AL522622] | 1,5504 | 2,96E-08 | 2,33E-06 |
| RAB3IL1 | NM_013401 | Homo sapiens RAB3A interacting protein (rabin3)-like 1 (RAB3IL1), mRNA [NM_013401] | 1,4630 | 2,97E-08 | 2,33E-06 |
| SLC30A1 | ENST00000367001 | Zinc transporter 1 (ZnT-1) (Solute carrier family 30 member 1). [Source:Uniprot/SWISSPROT;Acc:Q9Y6M5] [ENST00000367001] | 1,6792 | 2,97E-08 | 2,33E-06 |
| THC2530075 | THC2530075 | Unknown | 1,2926 | 3,00E-08 | 2,33E-06 |
| ENST00000296873 | ENST00000296873 | Septin-8. [Source:Uniprot/SWISSPROT;Acc:Q92599] [ENST00000296873] | 0,9230 | 3,07E-08 | 2,37E-06 |
| PIP5K2A | NM_005028 | Homo sapiens phosphatidylinositol-4-phosphate 5-kinase, type II, alpha (PIP5K2A), mRNA [NM_005028] | 1,3950 | 3,15E-08 | 2,42E-06 |
| SEC14L4 | NM_174977 | Homo sapiens SEC14-like 4 (S. cerevisiae) (SEC14L4), mRNA [NM_174977] | 2,5198 | 3,15E-08 | 2,42E-06 |
| CTB-1048E9.5 | NM_001013694 | Homo sapiens similar to SRR1-like protein (LOC402055), mRNA [NM_001013694] | 1,0542 | 3,21E-08 | 2,44E-06 |
| HK1 | NM_033500 | Homo sapiens hexokinase 1 (HK1), nuclear gene encoding mitochondrial protein, transcript variant 5, mRNA [NM_033500] | 1,4656 | 3,31E-08 | 2,51E-06 |
| LOC253012 | NM_001039372 | Homo sapiens hypothetical protein LOC253012 (LOC253012), transcript variant 1, mRNA [NM_001039372] | 2,2885 | 3,55E-08 | 2,63E-06 |
| MGST3 | NM_004528 | Homo sapiens microsomal glutathione S-transferase 3 (MGST3), mRNA [NM_004528] | 1,4572 | 3,74E-08 | 2,77E-06 |
| AF471454 | AF471454 | Homo sapiens clone 68-46a Ig heavy chain variable region, VH3 family mRNA, partial cds. [AF471454] | 1,9464 | 3,77E-08 | 2,77E-06 |
| MYH10 | NM_005964 | Homo sapiens myosin, heavy chain 10, non-muscle (MYH10), mRNA [NM_005964] | 1,1113 | 3,77E-08 | 2,77E-06 |
| TRIM10 | NM_006778 | Homo sapiens tripartite motif-containing 10 (TRIM10), transcript variant 1, mRNA [NM_006778] | 1,5529 | 3,79E-08 | 2,77E-06 |
| C14orf45 | NM_025057 | Homo sapiens chromosome 14 open reading frame 45 (C14orf45), mRNA [NM_025057] | 1,5159 | 3,96E-08 | 2,88E-06 |
| EPB41 | NM_004437 | Homo sapiens erythrocyte membrane protein band 4.1 (elliptocytosis 1, RH-linked) (EPB41), transcript variant 3, mRNA [NM_004437] | 1,3775 | 4,00E-08 | 2,90E-06 |
| AY172962 | AY172962 | Homo sapiens anti-rabies SOJB immunoglobulin lambda light chain mRNA, complete cds. [AY172962] | 2,2612 | 4,32E-08 | 3,11E-06 |
| CD242823 | CD242823 | AGENCOURT_14126724 NIH_MGC_179 Homo sapiens cDNA clone IMAGE:30385216 5', mRNA sequence [CD242823] | 1,5259 | 4,78E-08 | 3,39E-06 |
| A_24_P755069 | A_24_P755069 | Unknown | 2,0965 | 4,98E-08 | 3,49E-06 |
| LOC652254 | XR_019518 | PREDICTED: Homo sapiens similar to 60S ribosomal protein L8 (LOC652254), mRNA [XR_019518] | 1,2604 | 5,22E-08 | 3,63E-06 |
| ENST00000295410 | ENST00000295410 | Ig kappa chain V-I region HK101 precursor (Fragment). [Source:Uniprot/SWISSPROT;Acc:P01601] [ENST00000377228] | 2,1396 | 5,35E-08 | 3,69E-06 |
| AF038185 | AF038185 | Homo sapiens clone 23700 mRNA sequence. [AF038185] | 0,9841 | 5,40E-08 | 3,71E-06 |
| IL15RA | NM_172200 | Homo sapiens interleukin 15 receptor, alpha (IL15RA), transcript variant 2, mRNA [NM_172200] | 1,3490 | 5,52E-08 | 3,77E-06 |
| ENST00000371189 | ENST00000371189 | Nuclear factor 1 A-type (Nuclear factor 1/A) (NF1-A) (NFI-A) (NF-I/A) (CCAAT-box-binding transcription factor) (CTF) (TGGCA-binding protein). [Source:Uniprot/SWISSPROT;Acc:Q12857] [ENST00000371189] | 1,2707 | 5,76E-08 | 3,91E-06 |
| HBA1 | NM_000558 | Homo sapiens hemoglobin, alpha 1 (HBA1), mRNA [NM_000558] | 2,0607 | 5,97E-08 | 4,03E-06 |
| NFIA | NM_005595 | Homo sapiens nuclear factor I/A (NFIA), mRNA [NM_005595] | 1,8800 | 6,00E-08 | 4,03E-06 |
| ZNF416 | NM_017879 | Homo sapiens zinc finger protein 416 (ZNF416), mRNA [NM_017879] | 1,0478 | 6,00E-08 | 4,03E-06 |
| RKHD1 | NM_203304 | Homo sapiens ring finger and KH domain containing 1 (RKHD1), mRNA [NM_203304] | 0,7958 | 6,46E-08 | 4,31E-06 |
| VKORC1 | NM_206824 | Homo sapiens vitamin K epoxide reductase complex, subunit 1 (VKORC1), transcript variant 2, mRNA [NM_206824] | 1,3726 | 6,57E-08 | 4,36E-06 |
| C8orf55 | NM_016647 | Homo sapiens chromosome 8 open reading frame 55 (C8orf55), mRNA [NM_016647] | 0,9724 | 6,68E-08 | 4,41E-06 |
| UBE2O | NM_022066 | Homo sapiens ubiquitin-conjugating enzyme E2O (UBE2O), mRNA [NM_022066] | 1,7904 | 6,77E-08 | 4,45E-06 |
| CA3 | NM_005181 | Homo sapiens carbonic anhydrase III, muscle specific (CA3), mRNA [NM_005181] | 1,3293 | 6,92E-08 | 4,53E-06 |
| BC032451 | BC032451 | Homo sapiens cDNA clone MGC:40426 IMAGE:5178085, complete cds. [BC032451] | 1,6531 | 7,89E-08 | 5,07E-06 |
| BC000986 | BC000986 | Homo sapiens cDNA clone IMAGE:3446313, complete cds. [BC000986] | 1,1742 | 8,05E-08 | 5,12E-06 |
| A_24_P7330 | A_24_P7330 | Unknown | 2,0040 | 8,57E-08 | 5,38E-06 |
| ARHGAP23 | XM_290799 | PREDICTED: Homo sapiens Rho GTPase activating protein 23, transcript variant 1 (ARHGAP23), mRNA [XM_290799] | 1,8367 | 8,64E-08 | 5,41E-06 |
| BC031344 | BC031344 | Homo sapiens, Similar to makorin, ring finger protein, 1, clone IMAGE:5556543, mRNA. [BC031344] | 1,7140 | 9,28E-08 | 5,72E-06 |
| PROP1 | NM_006261 | Homo sapiens prophet of Pit1, paired-like homeodomain transcription factor (PROP1), mRNA [NM_006261] | 1,8345 | 9,28E-08 | 5,72E-06 |
| CDC34 | NM_004359 | Homo sapiens cell division cycle 34 homolog (S. cerevisiae) (CDC34), mRNA [NM_004359] | 1,3057 | 9,62E-08 | 5,92E-06 |
| PSMF1 | NM_006814 | Homo sapiens proteasome (prosome, macropain) inhibitor subunit 1 (PI31) (PSMF1), transcript variant 1, mRNA [NM_006814] | 1,2147 | 1,01E-07 | 6,20E-06 |
| ANKRD33 | NM_182608 | Homo sapiens ankyrin repeat domain 33 (ANKRD33), mRNA [NM_182608] | 1,7653 | 1,03E-07 | 6,31E-06 |
| AJ319669 | AJ319669 | Homo sapiens germline mRNA for immunoglobulin lambda-2 chain constant region, Daudi cell line. [AJ319669] | 1,6291 | 1,08E-07 | 6,56E-06 |
| RPIA | NM_144563 | Homo sapiens ribose 5-phosphate isomerase A (ribose 5-phosphate epimerase) (RPIA), mRNA [NM_144563] | 1,1205 | 1,09E-07 | 6,60E-06 |
| A_24_P203886 | A_24_P203886 | Unknown | 1,4682 | 1,10E-07 | 6,61E-06 |
| C3orf39 | NM_032806 | Homo sapiens chromosome 3 open reading frame 39 (C3orf39), mRNA [NM_032806] | 1,3464 | 1,12E-07 | 6,75E-06 |
| PRPS1 | NM_002764 | Homo sapiens phosphoribosyl pyrophosphate synthetase 1 (PRPS1), mRNA [NM_002764] | 1,4513 | 1,13E-07 | 6,78E-06 |
| ACHE | NM_000665 | Homo sapiens acetylcholinesterase (Yt blood group) (ACHE), transcript variant E4-E6, mRNA [NM_000665] | 1,3802 | 1,14E-07 | 6,82E-06 |
| FLJ36208 | NM_176677 | Homo sapiens hypothetical protein FLJ36208 (FLJ36208), mRNA [NM_176677] | 1,1128 | 1,21E-07 | 7,22E-06 |
| PGM2L1 | NM_173582 | Homo sapiens phosphoglucomutase 2-like 1 (PGM2L1), mRNA [NM_173582] | 0,9485 | 1,35E-07 | 7,95E-06 |
| AY062331 | AY062331 | Homo sapiens clone 105/707 immunoglobulin light chain kappa variable region mRNA, partial cds. [AY062331] | 1,5648 | 1,38E-07 | 8,05E-06 |
| ENST00000377233 | ENST00000377233 | Human clone 120Pa immunoglobulin light chain variable region (VkJ) mRNA, partial cds. [U21012] | 1,2885 | 1,38E-07 | 8,05E-06 |
| SLC22A16 | NM_033125 | Homo sapiens solute carrier family 22 (organic cation transporter), member 16 (SLC22A16), mRNA [NM_033125] | 1,6374 | 1,40E-07 | 8,10E-06 |
| NAP1L4 | NM_005969 | Homo sapiens nucleosome assembly protein 1-like 4 (NAP1L4), mRNA [NM_005969] | 1,1474 | 1,42E-07 | 8,17E-06 |
| ABCC13 | NR_003088 | Homo sapiens ATP-binding cassette, sub-family C (CFTR/MRP), member 13 (ABCC13) on chromosome 21 [NR_003088] | 1,9382 | 1,43E-07 | 8,24E-06 |
| GDPD5 | NM_030792 | Homo sapiens glycerophosphodiester phosphodiesterase domain containing 5 (GDPD5), mRNA [NM_030792] | 1,0787 | 1,51E-07 | 8,57E-06 |
| SLC38A5 | NM_033518 | Homo sapiens solute carrier family 38, member 5 (SLC38A5), mRNA [NM_033518] | 1,3114 | 1,52E-07 | 8,63E-06 |
| MYL4 | NM_002476 | Homo sapiens myosin, light chain 4, alkali; atrial, embryonic (MYL4), transcript variant 2, mRNA [NM_002476] | 1,1608 | 1,53E-07 | 8,64E-06 |
| ABCB6 | NM_005689 | Homo sapiens ATP-binding cassette, sub-family B (MDR/TAP), member 6 (ABCB6), nuclear gene encoding mitochondrial protein, mRNA [NM_005689] | 1,7364 | 1,55E-07 | 8,74E-06 |
| FREQ | NM_014286 | Homo sapiens frequenin homolog (Drosophila) (FREQ), mRNA [NM_014286] | 1,6572 | 1,56E-07 | 8,74E-06 |
| CTSE | NM_001910 | Homo sapiens cathepsin E (CTSE), transcript variant 1, mRNA [NM_001910] | 1,6363 | 1,70E-07 | 9,46E-06 |
| UCP2 | NM_003355 | Homo sapiens uncoupling protein 2 (mitochondrial, proton carrier) (UCP2), nuclear gene encoding mitochondrial protein, mRNA [NM_003355] | 1,6565 | 1,75E-07 | 9,73E-06 |
| ENST00000259219 | ENST00000259219 | Homo sapiens clone 63a12 anti-tetanus toxoid immunoglobulin light chain variable region (IGL@) mRNA, partial cds. [AY867113] | 1,6653 | 1,82E-07 | 1,00E-05 |
| ENST00000383048 | ENST00000383048 | Ig gamma-1 chain C region. [Source:Uniprot/SWISSPROT;Acc:P01857] [ENST00000383048] | 1,7754 | 1,83E-07 | 1,00E-05 |
| CCNE1 | NM_001238 | Homo sapiens cyclin E1 (CCNE1), transcript variant 1, mRNA [NM_001238] | 1,1588 | 1,84E-07 | 1,01E-05 |
| ENST00000331696 | ENST00000331696 | HUMIGKPB Ig kappa chain {Homo sapiens} (exp=-1; wgp=0; cg=0), complete [THC2557512] | 1,8422 | 1,85E-07 | 1,01E-05 |
| ENST00000327926 | ENST00000327926 | Homo sapiens isolate donor Z clone Z1K immunoglobulin kappa light chain variable region mRNA, partial cds. [AF103555] | 1,5191 | 1,92E-07 | 1,05E-05 |
| X57818 | X57818 | Human rearranged immunoglobulin lambda light chain mRNA. [X57818] | 1,7933 | 1,95E-07 | 1,06E-05 |
| THC2618446 | THC2618446 | Q213Y3_RHOPA (Q213Y3) Single-strand binding protein, partial (9%) [THC2618446] | 1,1616 | 1,96E-07 | 1,06E-05 |
| FZD1 | NM_003505 | Homo sapiens frizzled homolog 1 (Drosophila) (FZD1), mRNA [NM_003505] | 1,1405 | 1,97E-07 | 1,06E-05 |
| SDC1 | NM_001006946 | Homo sapiens syndecan 1 (SDC1), transcript variant 1, mRNA [NM_001006946] | 1,8191 | 2,00E-07 | 1,08E-05 |
| GABRP | NM_014211 | Homo sapiens gamma-aminobutyric acid (GABA) A receptor, pi (GABRP), mRNA [NM_014211] | 1,8770 | 2,02E-07 | 1,09E-05 |
| SFRS2B | NM_032102 | Homo sapiens splicing factor, arginine/serine-rich 2B (SFRS2B), mRNA [NM_032102] | 1,0784 | 2,07E-07 | 1,11E-05 |
| SLC25A38 | NM_017875 | Homo sapiens solute carrier family 25, member 38 (SLC25A38), mRNA [NM_017875] | 1,3123 | 2,17E-07 | 1,15E-05 |
| FLJ20489 | NM_017842 | Homo sapiens hypothetical protein FLJ20489 (FLJ20489), mRNA [NM_017842] | 0,9479 | 2,25E-07 | 1,18E-05 |
| LNX2 | NM_153371 | Homo sapiens ligand of numb-protein X 2 (LNX2), mRNA [NM_153371] | 0,9704 | 2,27E-07 | 1,19E-05 |
| BC030813 | BC030813 | Homo sapiens cDNA clone MGC:22645 IMAGE:4700961, complete cds. [BC030813] | 1,8300 | 2,33E-07 | 1,21E-05 |
| C20orf175 | NM_080829 | Homo sapiens chromosome 20 open reading frame 175 (C20orf175), mRNA [NM_080829] | 1,6326 | 2,33E-07 | 1,21E-05 |
| AF267875 | AF267875 | Homo sapiens amyloid lambda 6 light chain variable region SAR mRNA, partial cds. [AF267875] | 1,5664 | 2,39E-07 | 1,23E-05 |
| FRMD4A | AK057828 | Homo sapiens cDNA FLJ25099 fis, clone CBR01272. [AK057828] | 2,0298 | 2,43E-07 | 1,25E-05 |
| CPOX | NM_000097 | Homo sapiens coproporphyrinogen oxidase (CPOX), mRNA [NM_000097] | 1,7232 | 2,58E-07 | 1,32E-05 |
| PAGE5 | NM_130467 | Homo sapiens P antigen family, member 5 (prostate associated) (PAGE5), transcript variant 1, mRNA [NM_130467] | 1,6440 | 2,58E-07 | 1,32E-05 |
| BAG1 | NM_004323 | Homo sapiens BCL2-associated athanogene (BAG1), mRNA [NM_004323] | 1,3259 | 2,82E-07 | 1,44E-05 |
| MARCH3 | NM_178450 | Homo sapiens membrane-associated ring finger (C3HC4) 3 (MARCH3), mRNA [NM_178450] | 1,6803 | 2,83E-07 | 1,44E-05 |
| X57802 | X57802 | Human rearranged immunoglobulin lambda light chain mRNA. [X57802] | 2,6621 | 2,82E-07 | 1,44E-05 |
| TAL1 | NM_003189 | Homo sapiens T-cell acute lymphocytic leukemia 1 (TAL1), mRNA [NM_003189] | 1,6212 | 3,03E-07 | 1,53E-05 |
| SNX22 | NM_024798 | Homo sapiens sorting nexin 22 (SNX22), mRNA [NM_024798] | 1,6469 | 3,06E-07 | 1,54E-05 |
| DHRS13 | NM_144683 | Homo sapiens dehydrogenase/reductase (SDR family) member 13 (DHRS13), mRNA [NM_144683] | 1,6433 | 3,26E-07 | 1,63E-05 |
| FAM83D | NM_030919 | Homo sapiens family with sequence similarity 83, member D (FAM83D), mRNA [NM_030919] | 1,3566 | 3,42E-07 | 1,70E-05 |
| CA1 | NM_001738 | Homo sapiens carbonic anhydrase I (CA1), mRNA [NM_001738] | 2,8037 | 3,49E-07 | 1,73E-05 |
| A_23_P435390 | A_23_P435390 | Unknown | 2,5908 | 3,72E-07 | 1,82E-05 |
| C10orf12 | AK025166 | Homo sapiens cDNA: FLJ21513 fis, clone COL05778. [AK025166] | 1,2037 | 3,94E-07 | 1,92E-05 |
| RASGRP3 | NM_170672 | Homo sapiens RAS guanyl releasing protein 3 (calcium and DAG-regulated) (RASGRP3), mRNA [NM_170672] | 0,6679 | 3,97E-07 | 1,93E-05 |
| RSC1A1 | NM_006511 | Homo sapiens regulatory solute carrier protein, family 1, member 1 (RSC1A1), mRNA [NM_006511] | 0,8796 | 3,98E-07 | 1,93E-05 |
| ENST00000295339 | ENST00000295339 | Ig kappa chain V-III region VH precursor (Fragment). [Source:Uniprot/SWISSPROT;Acc:P04434] [ENST00000295339] | 1,8604 | 4,04E-07 | 1,95E-05 |
| THC2663668 | THC2663668 | Unknown | 1,0400 | 4,09E-07 | 1,97E-05 |
| TGM2 | NM_004613 | Homo sapiens transglutaminase 2 (C polypeptide, protein-glutamine-gamma-glutamyltransferase) (TGM2), transcript variant 1, mRNA [NM_004613] | 1,0742 | 4,41E-07 | 2,12E-05 |
| A_24_P490109 | A_24_P490109 | Unknown | 1,9621 | 4,46E-07 | 2,14E-05 |
| AW804491 | AW804491 | AW804491 QV0-UM0093-170400-191-d05 UM0093 Homo sapiens cDNA, mRNA sequence [AW804491] | 1,1637 | 4,59E-07 | 2,19E-05 |
| IGH@ | AK130614 | Homo sapiens cDNA FLJ27104 fis, clone SPL04981, highly similar to Ig gamma-2 chain C region. [AK130614] | 4,2121 | 4,63E-07 | 2,20E-05 |
| CDC27 | NM_001256 | Homo sapiens cell division cycle 27 homolog (S. cerevisiae) (CDC27), mRNA [NM_001256] | 1,2081 | 4,95E-07 | 2,34E-05 |
| TMEM23 | NM_147156 | Homo sapiens transmembrane protein 23 (TMEM23), mRNA [NM_147156] | 0,8723 | 4,95E-07 | 2,34E-05 |
| LRRC8A | NM_019594 | Homo sapiens leucine rich repeat containing 8 family, member A (LRRC8A), mRNA [NM_019594] | 1,0632 | 5,15E-07 | 2,43E-05 |
| DLC1 | NM_182643 | Homo sapiens deleted in liver cancer 1 (DLC1), transcript variant 1, mRNA [NM_182643] | 1,4854 | 5,19E-07 | 2,44E-05 |
| LOC134357 | XR_018355 | PREDICTED: Homo sapiens similar to aconitase 2 precursor (LOC134357), mRNA [XR_018355] | 1,1649 | 5,24E-07 | 2,44E-05 |
| PARVB | NM_001003828 | Homo sapiens parvin, beta (PARVB), transcript variant 1, mRNA [NM_001003828] | 1,0271 | 5,33E-07 | 2,48E-05 |
| A_24_P341126 | A_24_P341126 | Unknown | 1,9164 | 5,41E-07 | 2,50E-05 |
| RBM38 | NM_017495 | Homo sapiens RNA binding motif protein 38 (RBM38), transcript variant 1, mRNA [NM_017495] | 1,4665 | 5,41E-07 | 2,50E-05 |
| MINPP1 | NM_004897 | Homo sapiens multiple inositol polyphosphate histidine phosphatase, 1 (MINPP1), mRNA [NM_004897] | 1,4806 | 5,45E-07 | 2,51E-05 |
| UBL4A | NM_014235 | Homo sapiens ubiquitin-like 4A (UBL4A), mRNA [NM_014235] | 1,0293 | 5,69E-07 | 2,61E-05 |
| FGFR3 | NM_000142 | Homo sapiens fibroblast growth factor receptor 3 (achondroplasia, thanatophoric dwarfism) (FGFR3), transcript variant 1, mRNA [NM_000142] | 0,9366 | 5,85E-07 | 2,66E-05 |
| THC2674354 | THC2674354 | Q36LB5_MARHY (Q36LB5) Nucleoside-diphosphate-sugar epimerases, partial (8%) [THC2674354] | 0,8245 | 5,86E-07 | 2,66E-05 |
| PEX10 | NM_153818 | Homo sapiens peroxisome biogenesis factor 10 (PEX10), transcript variant 1, mRNA [NM_153818] | 0,6571 | 6,13E-07 | 2,75E-05 |
| THC2663297 | THC2663297 | Q5VWT3_HUMAN (Q5VWT3) Complement component (3b/4b) receptor 1-like, partial (27%) [THC2663297] | 1,8690 | 6,20E-07 | 2,78E-05 |
| AK125361 | AK125361 | Homo sapiens cDNA FLJ43371 fis, clone NTONG2005969. [AK125361] | 1,6849 | 6,28E-07 | 2,80E-05 |
| APOBEC3C | NM_014508 | Homo sapiens apolipoprotein B mRNA editing enzyme, catalytic polypeptide-like 3C (APOBEC3C), mRNA [NM_014508] | 0,9538 | 6,29E-07 | 2,80E-05 |
| CYC1 | NM_001916 | Homo sapiens cytochrome c-1 (CYC1), mRNA [NM_001916] | 0,9612 | 6,27E-07 | 2,80E-05 |
| PIGQ | BC010094 | Homo sapiens phosphatidylinositol glycan, class Q, mRNA (cDNA clone IMAGE:3357878), partial cds. [BC010094] | 1,4875 | 6,52E-07 | 2,89E-05 |
| IGHG1 | BC092518 | Homo sapiens immunoglobulin heavy constant gamma 1 (G1m marker), mRNA (cDNA clone MGC:105004 IMAGE:3056327), complete cds. [BC092518] | 1,9217 | 6,68E-07 | 2,96E-05 |
| BMP2K | NM_017593 | Homo sapiens BMP2 inducible kinase (BMP2K), transcript variant 2, mRNA [NM_017593] | 1,0217 | 6,73E-07 | 2,97E-05 |
| ENST00000377221 | ENST00000377221 | Unknown | 1,8025 | 6,72E-07 | 2,97E-05 |
| SRXN1 | NM_080725 | Homo sapiens sulfiredoxin 1 homolog (S. cerevisiae) (SRXN1), mRNA [NM_080725] | 1,5355 | 6,75E-07 | 2,97E-05 |
| C13orf8 | NM_032436 | Homo sapiens chromosome 13 open reading frame 8 (C13orf8), mRNA [NM_032436] | 1,0950 | 6,81E-07 | 2,98E-05 |
| RGS10 | NM_001005339 | Homo sapiens regulator of G-protein signalling 10 (RGS10), transcript variant 1, mRNA [NM_001005339] | 0,9751 | 6,85E-07 | 3,00E-05 |
| GZMK | NM_002104 | Homo sapiens granzyme K (granzyme 3; tryptase II) (GZMK), mRNA [NM_002104] | 1,8758 | 6,91E-07 | 3,02E-05 |
| AK074614 | AK074614 | Homo sapiens cDNA FLJ90133 fis, clone HEMBB1000567. [AK074614] | 2,3778 | 7,06E-07 | 3,06E-05 |
| SLC7A1 | NM_003045 | Homo sapiens solute carrier family 7 (cationic amino acid transporter, y+ system), member 1 (SLC7A1), mRNA [NM_003045] | 1,0801 | 7,25E-07 | 3,14E-05 |
| THC2527772 | THC2527772 | HUMC4AA2 complement component C4A {Homo sapiens} (exp=-1; wgp=0; cg=0), partial (6%) [THC2527772] | 1,0980 | 7,28E-07 | 3,14E-05 |
| PRDX6 | NM_004905 | Homo sapiens peroxiredoxin 6 (PRDX6), mRNA [NM_004905] | 0,9681 | 7,56E-07 | 3,24E-05 |
| NCOA4 | NM_005437 | Homo sapiens nuclear receptor coactivator 4 (NCOA4), mRNA [NM_005437] | 1,3816 | 7,63E-07 | 3,27E-05 |
| RANBP10 | NM_020850 | Homo sapiens RAN binding protein 10 (RANBP10), mRNA [NM_020850] | 1,4840 | 8,09E-07 | 3,44E-05 |
| GSPT1 | NM_002094 | Homo sapiens G1 to S phase transition 1 (GSPT1), mRNA [NM_002094] | 1,5496 | 8,24E-07 | 3,50E-05 |
| PAGE2 | NM_207339 | Homo sapiens P antigen family, member 2 (prostate associated) (PAGE2), mRNA [NM_207339] | 1,6067 | 8,31E-07 | 3,52E-05 |
| UBXD1 | NM_025241 | Homo sapiens UBX domain containing 1 (UBXD1), mRNA [NM_025241] | 1,3515 | 8,36E-07 | 3,53E-05 |
| FIS1 | NM_016068 | Homo sapiens fission 1 (mitochondrial outer membrane) homolog (S. cerevisiae) (FIS1), mRNA [NM_016068] | 0,9501 | 8,39E-07 | 3,53E-05 |
| RNF26 | NM_032015 | Homo sapiens ring finger protein 26 (RNF26), mRNA [NM_032015] | 0,9740 | 8,51E-07 | 3,58E-05 |
| C4B | NM_001002029 | Homo sapiens complement component 4B (Childo blood group) (C4B), mRNA [NM_001002029] | 1,4673 | 8,66E-07 | 3,63E-05 |
| MXI1 | NM_130439 | Homo sapiens MAX interactor 1 (MXI1), transcript variant 2, mRNA [NM_130439] | 1,4181 | 8,96E-07 | 3,75E-05 |
| SDSL | NM_138432 | Homo sapiens serine dehydratase-like (SDSL), mRNA [NM_138432] | 1,1127 | 9,14E-07 | 3,81E-05 |
| CDH1 | NM_004360 | Homo sapiens cadherin 1, type 1, E-cadherin (epithelial) (CDH1), mRNA [NM_004360] | 1,5071 | 9,33E-07 | 3,88E-05 |
| LOC340508 | NR_002942 | Homo sapiens hypothetical protein LOC340508 (LOC340508) on chromosome 9 [NR_002942] | 0,7919 | 9,50E-07 | 3,93E-05 |
| CCR5 | NM_000579 | Homo sapiens chemokine (C-C motif) receptor 5 (CCR5), mRNA [NM_000579] | 1,3709 | 9,59E-07 | 3,96E-05 |
| AMMECR1 | NM_015365 | Homo sapiens Alport syndrome, mental retardation, midface hypoplasia and elliptocytosis chromosomal region, gene 1 (AMMECR1), transcript variant 1, mRNA [NM_015365] | 1,1017 | 9,65E-07 | 3,97E-05 |
| SLC37A4 | NM_001467 | Homo sapiens solute carrier family 37 (glycerol-6-phosphate transporter), member 4 (SLC37A4), mRNA [NM_001467] | 0,9677 | 1,02E-06 | 4,19E-05 |
| WDR34 | NM_052844 | Homo sapiens WD repeat domain 34 (WDR34), mRNA [NM_052844] | 1,2867 | 1,03E-06 | 4,19E-05 |
| A_23_P44053 | A_23_P44053 | Unknown | 1,7656 | 1,03E-06 | 4,21E-05 |
| HNRPAB | NM_004499 | Homo sapiens heterogeneous nuclear ribonucleoprotein A/B (HNRPAB), transcript variant 2, mRNA [NM_004499] | 1,1602 | 1,05E-06 | 4,28E-05 |
| DLEU2 | CR625878 | full-length cDNA clone CS0DJ009YK01 of T cells (Jurkat cell line) Cot 10-normalized of Homo sapiens (human). [CR625878] | 0,8301 | 1,10E-06 | 4,44E-05 |
| PC | NM_001040716 | Homo sapiens pyruvate carboxylase (PC), nuclear gene encoding mitochondrial protein, transcript variant 3, mRNA [NM_001040716] | 1,0272 | 1,14E-06 | 4,59E-05 |
| OPA1 | NM_130837 | Homo sapiens optic atrophy 1 (autosomal dominant) (OPA1), nuclear gene encoding mitochondrial protein, transcript variant 8, mRNA [NM_130837] | 1,2226 | 1,16E-06 | 4,67E-05 |
| TMOD1 | NM_003275 | Homo sapiens tropomodulin 1 (TMOD1), mRNA [NM_003275] | 2,0190 | 1,16E-06 | 4,67E-05 |
| MYBL2 | NM_002466 | Homo sapiens v-myb myeloblastosis viral oncogene homolog (avian)-like 2 (MYBL2), mRNA [NM_002466] | 1,4184 | 1,21E-06 | 4,84E-05 |
| RAD23A | NM_005053 | Homo sapiens RAD23 homolog A (S. cerevisiae) (RAD23A), mRNA [NM_005053] | 1,5687 | 1,21E-06 | 4,84E-05 |
| AJ399872 | AJ399872 | Homo sapiens partial mRNA for thyroid peroxidase-specific immunoglobulin kappa chain variable egion (IGKV gene), clone T2. [AJ399872] | 1,0820 | 1,26E-06 | 5,02E-05 |
| AW302758 | AW302758 | AW302758 xr55g08.x1 NCI_CGAP_Ov26 Homo sapiens cDNA clone IMAGE:2764094 3', mRNA sequence [AW302758] | 1,6744 | 1,34E-06 | 5,30E-05 |
| USP12 | ENST00000258451 | Ubiquitin carboxyl-terminal hydrolase 12 (EC 3.1.2.15) (Ubiquitin thioesterase 12) (Ubiquitin-specific-processing protease 12) (Deubiquitinating enzyme 12) (Ubiquitin-hydrolyzing enzyme 1). [Source:Uniprot/SWISSPROT;Acc:O75317] [ENST00000258451] | 1,2320 | 1,35E-06 | 5,32E-05 |
| RNF182 | NM_152737 | Homo sapiens ring finger protein 182 (RNF182), mRNA [NM_152737] | 1,9681 | 1,38E-06 | 5,42E-05 |
| HBD | NM_000519 | Homo sapiens hemoglobin, delta (HBD), mRNA [NM_000519] | 1,6099 | 1,39E-06 | 5,46E-05 |
| CKAP2L | NM_152515 | Homo sapiens cytoskeleton associated protein 2-like (CKAP2L), mRNA [NM_152515] | 1,1152 | 1,40E-06 | 5,50E-05 |
| CD612636 | CD612636 | CD612636 55102867H1 FLP Homo sapiens cDNA, mRNA sequence [CD612636] | 0,8952 | 1,42E-06 | 5,55E-05 |
| ELOVL1 | NM_022821 | Homo sapiens elongation of very long chain fatty acids (FEN1/Elo2, SUR4/Elo3, yeast)-like 1 (ELOVL1), mRNA [NM_022821] | 1,2960 | 1,42E-06 | 5,55E-05 |
| TESC | NM_017899 | Homo sapiens tescalcin (TESC), mRNA [NM_017899] | 0,9282 | 1,46E-06 | 5,69E-05 |
| CHID1 | NM_023947 | Homo sapiens chitinase domain containing 1 (CHID1), mRNA [NM_023947] | 0,9018 | 1,47E-06 | 5,71E-05 |
| NUS1 | NM_138459 | Homo sapiens nuclear undecaprenyl pyrophosphate synthase 1 homolog (S. cerevisiae) (NUS1), mRNA [NM_138459] | 1,0122 | 1,51E-06 | 5,87E-05 |
| NOLA1 | NM_018983 | Homo sapiens nucleolar protein family A, member 1 (H/ACA small nucleolar RNPs) (NOLA1), transcript variant 1, mRNA [NM_018983] | 0,7874 | 1,54E-06 | 5,96E-05 |
| JAZF1 | NM_175061 | Homo sapiens JAZF zinc finger 1 (JAZF1), mRNA [NM_175061] | 1,4068 | 1,55E-06 | 5,97E-05 |
| DERL3 | NM_198440 | Homo sapiens Der1-like domain family, member 3 (DERL3), transcript variant 1, mRNA [NM_198440] | 1,4448 | 1,59E-06 | 6,12E-05 |
| MPDU1 | NM_004870 | Homo sapiens mannose-P-dolichol utilization defect 1 (MPDU1), mRNA [NM_004870] | 0,8113 | 1,62E-06 | 6,20E-05 |
| STK11 | NM_000455 | Homo sapiens serine/threonine kinase 11 (STK11), mRNA [NM_000455] | 1,0840 | 1,67E-06 | 6,37E-05 |
| POLR1C | NM_004875 | Homo sapiens polymerase (RNA) I polypeptide C, 30kDa (POLR1C), transcript variant 2, mRNA [NM_004875] | 0,7834 | 1,71E-06 | 6,51E-05 |
| HES5 | NM_001010926 | Homo sapiens hairy and enhancer of split 5 (Drosophila) (HES5), mRNA [NM_001010926] | 1,2725 | 1,71E-06 | 6,52E-05 |
| FBXO30 | NM_032145 | Homo sapiens F-box protein 30 (FBXO30), mRNA [NM_032145] | 1,3182 | 1,72E-06 | 6,53E-05 |
| ODC1 | NM_002539 | Homo sapiens ornithine decarboxylase 1 (ODC1), mRNA [NM_002539] | 1,2850 | 1,73E-06 | 6,55E-05 |
| A_32_P108592 | A_32_P108592 | Unknown | 1,0775 | 1,74E-06 | 6,58E-05 |
| IDH2 | NM_002168 | Homo sapiens isocitrate dehydrogenase 2 (NADP+), mitochondrial (IDH2), mRNA [NM_002168] | 1,2781 | 1,75E-06 | 6,61E-05 |
| DPF3 | AK124946 | Homo sapiens cDNA FLJ42956 fis, clone BRSTN2009899. [AK124946] | 1,4516 | 1,76E-06 | 6,62E-05 |
| LOC642413 | XR_016155 | PREDICTED: Homo sapiens similar to Cathepsin L precursor (Major excreted protein) (MEP) (LOC642413), mRNA [XR_016155] | 1,2637 | 1,79E-06 | 6,69E-05 |
| A_24_P384604 | A_24_P384604 | Unknown | 1,3278 | 1,79E-06 | 6,69E-05 |
| CENPP | NM_001012267 | Homo sapiens centromere protein P (CENPP), mRNA [NM_001012267] | 0,8863 | 1,80E-06 | 6,71E-05 |
| C1orf128 | NM_020362 | Homo sapiens chromosome 1 open reading frame 128 (C1orf128), mRNA [NM_020362] | 1,4191 | 1,81E-06 | 6,74E-05 |
| CTA-246H3.1 | NM_001013618 | Homo sapiens similar to omega protein (LOC91353), mRNA [NM_001013618] | 1,8381 | 1,83E-06 | 6,81E-05 |
| FAM109B | NM_001002034 | Homo sapiens family with sequence similarity 109, member B (FAM109B), mRNA [NM_001002034] | 0,5957 | 1,90E-06 | 7,01E-05 |
| VWCE | NM_152718 | Homo sapiens von Willebrand factor C and EGF domains (VWCE), mRNA [NM_152718] | 1,9642 | 1,91E-06 | 7,07E-05 |
| SPBC24 | NM_182513 | Homo sapiens spindle pole body component 24 homolog (S. cerevisiae) (SPBC24), mRNA [NM_182513] | 1,0611 | 1,97E-06 | 7,26E-05 |
| FOXRED2 | NM_024955 | Homo sapiens FAD-dependent oxidoreductase domain containing 2 (FOXRED2), mRNA [NM_024955] | 0,6675 | 1,98E-06 | 7,27E-05 |
| PPP2R1B | NM_002716 | Homo sapiens protein phosphatase 2 (formerly 2A), regulatory subunit A (PR 65), beta isoform (PPP2R1B), transcript variant 1, mRNA [NM_002716] | 0,7776 | 2,01E-06 | 7,38E-05 |
| LCMT2 | NM_014793 | Homo sapiens leucine carboxyl methyltransferase 2 (LCMT2), mRNA [NM_014793] | 0,8546 | 2,03E-06 | 7,44E-05 |
| X01147 | X01147 | Human mRNA for immunoglobulin lambda variable region corresponding to NEW protein of V lambda subgroup I. [X01147] | 1,9985 | 2,08E-06 | 7,61E-05 |
| TOP1 | NM_003286 | Homo sapiens topoisomerase (DNA) I (TOP1), mRNA [NM_003286] | 0,9367 | 2,12E-06 | 7,71E-05 |
| TUBB | NM_178014 | Homo sapiens tubulin, beta (TUBB), mRNA [NM_178014] | 0,7745 | 2,13E-06 | 7,73E-05 |
| LHFPL2 | NM_005779 | Homo sapiens lipoma HMGIC fusion partner-like 2 (LHFPL2), mRNA [NM_005779] | 1,0798 | 2,16E-06 | 7,81E-05 |
| APOBEC3F | NM_145298 | Homo sapiens apolipoprotein B mRNA editing enzyme, catalytic polypeptide-like 3F (APOBEC3F), transcript variant 1, mRNA [NM_145298] | 0,8707 | 2,21E-06 | 7,95E-05 |
| THC2522223 | THC2522223 | AF192784 makorin 1 {Homo sapiens} (exp=-1; wgp=0; cg=0), partial (15%) [THC2522223] | 1,2077 | 2,25E-06 | 8,07E-05 |
| CD36 | NM_001001547 | Homo sapiens CD36 molecule (thrombospondin receptor) (CD36), transcript variant 2, mRNA [NM_001001547] | 1,3070 | 2,37E-06 | 8,46E-05 |
| KLHDC3 | NM_057161 | Homo sapiens kelch domain containing 3 (KLHDC3), mRNA [NM_057161] | 0,6885 | 2,38E-06 | 8,46E-05 |
| PBX1 | ENST00000328681 | Pre-B-cell leukemia transcription factor 1 (Homeobox protein PBX1) (Homeobox protein PRL). [Source:Uniprot/SWISSPROT;Acc:P40424] [ENST00000328681] | 1,3258 | 2,47E-06 | 8,77E-05 |
| C9orf125 | NM_032342 | Homo sapiens chromosome 9 open reading frame 125 (C9orf125), mRNA [NM_032342] | 1,3302 | 2,53E-06 | 8,97E-05 |
| IGJ | NM_144646 | Homo sapiens immunoglobulin J polypeptide, linker protein for immunoglobulin alpha and mu polypeptides (IGJ), mRNA [NM_144646] | 1,8164 | 2,61E-06 | 9,17E-05 |
| SHARPIN | NM_030974 | Homo sapiens SHANK-associated RH domain interactor (SHARPIN), mRNA [NM_030974] | 0,9700 | 2,62E-06 | 9,19E-05 |
| AJ009817 | AJ009817 | Homo sapiens mRNA for AMMECR1 protein, alternative exon 2. [AJ009817] | 1,4681 | 2,67E-06 | 9,35E-05 |
| LRRN5 | NM_201630 | Homo sapiens leucine rich repeat neuronal 5 (LRRN5), transcript variant 2, mRNA [NM_201630] | 1,6897 | 2,67E-06 | 9,35E-05 |
| BC035146 | BC035146 | Homo sapiens cDNA clone IMAGE:5264735. [BC035146] | 1,5636 | 2,74E-06 | 9,54E-05 |
| ENST00000307840 | ENST00000307840 | Homo sapiens partial mRNA for immunoglobulin kappa light chain variable region (IGKV gene), isolate B-CLL 302. [AJ697902] | 1,5997 | 2,74E-06 | 9,54E-05 |
| UBB | NM_018955 | Homo sapiens ubiquitin B (UBB), mRNA [NM_018955] | 0,9154 | 2,87E-06 | 9,91E-05 |
| HBLD2 | NM_030940 | Homo sapiens HESB like domain containing 2 (HBLD2), mRNA [NM_030940] | 0,8483 | 2,94E-06 | 1,01E-04 |
| ACO2 | NM_001098 | Homo sapiens aconitase 2, mitochondrial (ACO2), nuclear gene encoding mitochondrial protein, mRNA [NM_001098] | 0,7713 | 2,96E-06 | 1,02E-04 |
| GOSR2 | NM_004287 | Homo sapiens golgi SNAP receptor complex member 2 (GOSR2), transcript variant A, mRNA [NM_004287] | 0,7333 | 3,03E-06 | 1,04E-04 |
| PHOSPHO1 | NM_178500 | Homo sapiens phosphatase, orphan 1 (PHOSPHO1), mRNA [NM_178500] | 0,9451 | 3,22E-06 | 1,10E-04 |
| DNAJA4 | NM_018602 | Homo sapiens DnaJ (Hsp40) homolog, subfamily A, member 4 (DNAJA4), mRNA [NM_018602] | 1,4580 | 3,41E-06 | 1,15E-04 |
| FAHD1 | NM_031208 | Homo sapiens fumarylacetoacetate hydrolase domain containing 1 (FAHD1), transcript variant 2, mRNA [NM_031208] | 1,1411 | 3,42E-06 | 1,15E-04 |
| MPP1 | NM_002436 | Homo sapiens membrane protein, palmitoylated 1, 55kDa (MPP1), mRNA [NM_002436] | 1,1044 | 3,47E-06 | 1,16E-04 |
| THC2497143 | THC2497143 | Q6PIQ7_HUMAN (Q6PIQ7) IGLC1 protein, partial (98%) [THC2497143] | 0,9423 | 3,60E-06 | 1,20E-04 |
| FAM20B | NM_014864 | Homo sapiens family with sequence similarity 20, member B (FAM20B), mRNA [NM_014864] | 1,3572 | 3,66E-06 | 1,22E-04 |
| FAM82C | NM_018145 | Homo sapiens family with sequence similarity 82, member C (FAM82C), mRNA [NM_018145] | 0,9556 | 3,71E-06 | 1,23E-04 |
| MAN2A1 | NM_002372 | Homo sapiens mannosidase, alpha, class 2A, member 1 (MAN2A1), mRNA [NM_002372] | 0,7431 | 3,71E-06 | 1,23E-04 |
| ABCB10 | NM_012089 | Homo sapiens ATP-binding cassette, sub-family B (MDR/TAP), member 10 (ABCB10), nuclear gene encoding mitochondrial protein, mRNA [NM_012089] | 1,0225 | 3,77E-06 | 1,25E-04 |
| LPIN2 | NM_014646 | Homo sapiens lipin 2 (LPIN2), mRNA [NM_014646] | 0,8970 | 3,89E-06 | 1,28E-04 |
| GRPEL2 | NM_152407 | Homo sapiens GrpE-like 2, mitochondrial (E. coli) (GRPEL2), nuclear gene encoding mitochondrial protein, mRNA [NM_152407] | 0,7651 | 3,92E-06 | 1,29E-04 |
| HEBP1 | NM_015987 | Homo sapiens heme binding protein 1 (HEBP1), mRNA [NM_015987] | 1,1734 | 3,96E-06 | 1,30E-04 |
| PIM1 | NM_002648 | Homo sapiens pim-1 oncogene (PIM1), mRNA [NM_002648] | 1,6840 | 3,96E-06 | 1,30E-04 |
| GUK1 | ENST00000366716 | Guanylate kinase (EC 2.7.4.8) (GMP kinase). [Source:Uniprot/SWISSPROT;Acc:Q16774] [ENST00000366716] | 1,1166 | 4,01E-06 | 1,31E-04 |
| FBXO34 | NM_017943 | Homo sapiens F-box protein 34 (FBXO34), mRNA [NM_017943] | 1,1911 | 4,11E-06 | 1,34E-04 |
| CMAS | NM_018686 | Homo sapiens cytidine monophosphate N-acetylneuraminic acid synthetase (CMAS), mRNA [NM_018686] | 1,3302 | 4,12E-06 | 1,34E-04 |
| ACOT7 | NM_007274 | Homo sapiens acyl-CoA thioesterase 7 (ACOT7), transcript variant hBACHa, mRNA [NM_007274] | 1,2379 | 4,18E-06 | 1,36E-04 |
| AF063695 | AF063695 | Homo sapiens clone BCPBLL11 immunoglobulin lambda light chain variable region mRNA, partial cds. [AF063695] | 1,4795 | 4,19E-06 | 1,36E-04 |
| BTRC | NM_033637 | Homo sapiens beta-transducin repeat containing (BTRC), transcript variant 1, mRNA [NM_033637] | 1,2405 | 4,20E-06 | 1,36E-04 |
| DNAJC9 | NM_015190 | Homo sapiens DnaJ (Hsp40) homolog, subfamily C, member 9 (DNAJC9), mRNA [NM_015190] | 1,0198 | 4,27E-06 | 1,38E-04 |
| BQ017638 | BQ017638 | BQ017638 UI-H-DI0-auv-p-03-0-UI.s1 NCI_CGAP_DI0 Homo sapiens cDNA clone IMAGE:5875058 3', mRNA sequence [BQ017638] | 0,9357 | 4,41E-06 | 1,42E-04 |
| EIF4G1 | NM_182917 | Homo sapiens eukaryotic translation initiation factor 4 gamma, 1 (EIF4G1), transcript variant 1, mRNA [NM_182917] | 0,7835 | 4,46E-06 | 1,43E-04 |
| MRFAP1 | NM_033296 | Homo sapiens Mof4 family associated protein 1 (MRFAP1), mRNA [NM_033296] | 0,8152 | 4,56E-06 | 1,47E-04 |
| ADD2 | NM_017482 | Homo sapiens adducin 2 (beta) (ADD2), transcript variant beta-2, mRNA [NM_017482] | 0,7436 | 4,60E-06 | 1,47E-04 |
| MARCH2 | NM_016496 | Homo sapiens membrane-associated ring finger (C3HC4) 2 (MARCH2), transcript variant 1, mRNA [NM_016496] | 0,9743 | 4,60E-06 | 1,47E-04 |
| C7orf41 | NM_152793 | Homo sapiens chromosome 7 open reading frame 41 (C7orf41), mRNA [NM_152793] | 0,8132 | 4,63E-06 | 1,47E-04 |
| Y11328 | Y11328 | H.sapiens mRNA for immunoglobulin heavy chain, partial, clone VH3-7. [Y11328] | 1,6819 | 4,62E-06 | 1,47E-04 |
| HCCA2 | NM_053005 | Homo sapiens HCCA2 protein (HCCA2), mRNA [NM_053005] | 0,7711 | 4,67E-06 | 1,49E-04 |
| E2F4 | NM_001950 | Homo sapiens E2F transcription factor 4, p107/p130-binding (E2F4), mRNA [NM_001950] | 1,2242 | 4,81E-06 | 1,52E-04 |
| ABCF2 | NM_005692 | Homo sapiens ATP-binding cassette, sub-family F (GCN20), member 2 (ABCF2), nuclear gene encoding mitochondrial protein, transcript variant 2, mRNA [NM_005692] | 0,7835 | 4,99E-06 | 1,58E-04 |
| NEDD4L | NM_015277 | Homo sapiens neural precursor cell expressed, developmentally down-regulated 4-like (NEDD4L), mRNA [NM_015277] | 0,8604 | 5,00E-06 | 1,58E-04 |
| D83692 | D83692 | Human mRNA for immunoglobulin M (IgM), partial cds (VH3-N-D-N-JH4), clone H2-39E. [D83692] | 1,2398 | 5,24E-06 | 1,65E-04 |
| KCNN4 | NM_002250 | Homo sapiens potassium intermediate/small conductance calcium-activated channel, subfamily N, member 4 (KCNN4), mRNA [NM_002250] | 1,0391 | 5,34E-06 | 1,67E-04 |
| C17orf71 | NM_018149 | Homo sapiens chromosome 17 open reading frame 71 (C17orf71), mRNA [NM_018149] | 0,8650 | 5,35E-06 | 1,68E-04 |
| BC022362 | BC022362 | Homo sapiens cDNA clone MGC:23888 IMAGE:4704496, complete cds. [BC022362] | 0,9568 | 5,38E-06 | 1,68E-04 |
| DDX28 | NM_018380 | Homo sapiens DEAD (Asp-Glu-Ala-Asp) box polypeptide 28 (DDX28), nuclear gene encoding mitochondrial protein, mRNA [NM_018380] | 0,9732 | 5,38E-06 | 1,68E-04 |
| C6orf85 | BC022217 | Homo sapiens chromosome 6 open reading frame 85, mRNA (cDNA clone IMAGE:3846727), complete cds. [BC022217] | 1,8939 | 5,54E-06 | 1,71E-04 |
| MCAT | NM_173467 | Homo sapiens malonyl CoA:ACP acyltransferase (mitochondrial) (MCAT), nuclear gene encoding mitochondrial protein, transcript variant 1, mRNA [NM_173467] | 0,8350 | 5,53E-06 | 1,71E-04 |
| ZFYVE21 | NM_024071 | Homo sapiens zinc finger, FYVE domain containing 21 (ZFYVE21), mRNA [NM_024071] | 1,2330 | 5,51E-06 | 1,71E-04 |
| DQ100840 | DQ100840 | Homo sapiens isolate N1553H immunoglobulin heavy chain variable region (IGHV3-21) mRNA, IGHV3-21*01 allele, partial cds. [DQ100840] | 1,6994 | 5,79E-06 | 1,78E-04 |
| CLN6 | NM_017882 | Homo sapiens ceroid-lipofuscinosis, neuronal 6, late infantile, variant (CLN6), mRNA [NM_017882] | 0,8927 | 5,82E-06 | 1,79E-04 |
| PPT2 | NM_005155 | Homo sapiens palmitoyl-protein thioesterase 2 (PPT2), transcript variant 1, mRNA [NM_005155] | 1,3036 | 5,98E-06 | 1,84E-04 |
| MGC17403 | ENST00000314720 | TFS2-M domain-containing protein 1. [Source:Uniprot/SWISSPROT;Acc:Q8N8B7] [ENST00000314720] | 1,1662 | 6,07E-06 | 1,85E-04 |
| THC2671299 | THC2671299 | NM_102092 oxidoreductase/ zinc ion binding {Arabidopsis thaliana} (exp=-1; wgp=0; cg=0), partial (7%) [THC2671299] | 0,9804 | 6,08E-06 | 1,85E-04 |
| LOC91461 | NM_138370 | Homo sapiens hypothetical protein BC007901 (LOC91461), mRNA [NM_138370] | 1,1647 | 6,15E-06 | 1,87E-04 |
| TIGD6 | NM_030953 | Homo sapiens tigger transposable element derived 6 (TIGD6), mRNA [NM_030953] | 0,6550 | 6,29E-06 | 1,90E-04 |
| RNF121 | NM_018320 | Homo sapiens ring finger protein 121 (RNF121), transcript variant 1, mRNA [NM_018320] | 0,6795 | 6,41E-06 | 1,93E-04 |
| CTSB | NM_147780 | Homo sapiens cathepsin B (CTSB), transcript variant 2, mRNA [NM_147780] | 1,2999 | 6,43E-06 | 1,94E-04 |
| A_24_P204574 | A_24_P204574 | Unknown | 1,7921 | 6,61E-06 | 1,98E-04 |
| SLC35A4 | NM_080670 | Homo sapiens solute carrier family 35, member A4 (SLC35A4), mRNA [NM_080670] | 0,7355 | 6,68E-06 | 1,99E-04 |
| BCL2L11 | NM_138621 | Homo sapiens BCL2-like 11 (apoptosis facilitator) (BCL2L11), transcript variant 1, mRNA [NM_138621] | 1,1776 | 6,73E-06 | 2,00E-04 |
| PDXP | NM_020315 | Homo sapiens pyridoxal (pyridoxine, vitamin B6) phosphatase (PDXP), mRNA [NM_020315] | 0,8902 | 6,72E-06 | 2,00E-04 |
| CR605719 | CR605719 | full-length cDNA clone CS0DK002YG10 of HeLa cells Cot 25-normalized of Homo sapiens (human). [CR605719] | 0,8533 | 6,77E-06 | 2,01E-04 |
| C22orf25 | CR627452 | Homo sapiens mRNA; cDNA DKFZp686O2142 (from clone DKFZp686O2142). [CR627452] | 0,6818 | 6,84E-06 | 2,02E-04 |
| C9orf78 | NM_016520 | Homo sapiens chromosome 9 open reading frame 78 (C9orf78), mRNA [NM_016520] | 1,2203 | 6,84E-06 | 2,02E-04 |
| AA420998 | AA420998 | AA420998 zu08f10.s1 Soares_testis_NHT Homo sapiens cDNA clone IMAGE:731275 3' similar to contains element MER22 MER22 repetitive element ;, mRNA sequence [AA420998] | 1,0589 | 6,96E-06 | 2,05E-04 |
| PYCRL | NM_023078 | Homo sapiens pyrroline-5-carboxylate reductase-like (PYCRL), mRNA [NM_023078] | 0,7326 | 6,97E-06 | 2,05E-04 |
| C22orf13 | NM_031444 | Homo sapiens chromosome 22 open reading frame 13 (C22orf13), mRNA [NM_031444] | 1,0371 | 7,02E-06 | 2,05E-04 |
| EPN2 | NM_014964 | Homo sapiens epsin 2 (EPN2), transcript variant 2, mRNA [NM_014964] | 0,9966 | 7,02E-06 | 2,05E-04 |
| THC2541642 | THC2541642 | Unknown | 0,6086 | 7,02E-06 | 2,05E-04 |
| TUBA3 | NM_006009 | Homo sapiens tubulin, alpha 3 (TUBA3), mRNA [NM_006009] | 1,1008 | 7,07E-06 | 2,06E-04 |
| FUT1 | NM_000148 | Homo sapiens fucosyltransferase 1 (galactoside 2-alpha-L-fucosyltransferase, H blood group) (FUT1), mRNA [NM_000148] | 1,0320 | 7,22E-06 | 2,10E-04 |
| TMEM86B | NM_173804 | Homo sapiens transmembrane protein 86B (TMEM86B), mRNA [NM_173804] | 1,0842 | 7,41E-06 | 2,15E-04 |
| GNA12 | NM_007353 | Homo sapiens guanine nucleotide binding protein (G protein) alpha 12 (GNA12), mRNA [NM_007353] | 1,1633 | 7,61E-06 | 2,19E-04 |
| CDYL | NM_170752 | Homo sapiens chromodomain protein, Y-like (CDYL), transcript variant 3, mRNA [NM_170752] | 0,9155 | 7,64E-06 | 2,20E-04 |
| CENPO | AK027859 | Homo sapiens cDNA FLJ14953 fis, clone PLACE3000160. [AK027859] | 1,2597 | 7,70E-06 | 2,21E-04 |
| MAF1 | NM_032272 | Homo sapiens MAF1 homolog (S. cerevisiae) (MAF1), mRNA [NM_032272] | 0,9602 | 7,75E-06 | 2,22E-04 |
| KIF21A | NM_017641 | Homo sapiens kinesin family member 21A (KIF21A), mRNA [NM_017641] | 0,9961 | 7,80E-06 | 2,22E-04 |
| LMNA | NM_005572 | Homo sapiens lamin A/C (LMNA), transcript variant 2, mRNA [NM_005572] | 1,4799 | 7,80E-06 | 2,22E-04 |
| FHL2 | NM_001039492 | Homo sapiens four and a half LIM domains 2 (FHL2), transcript variant 5, mRNA [NM_001039492] | 1,7402 | 7,86E-06 | 2,23E-04 |
| MCM5 | NM_006739 | Homo sapiens MCM5 minichromosome maintenance deficient 5, cell division cycle 46 (S. cerevisiae) (MCM5), mRNA [NM_006739] | 0,7765 | 7,95E-06 | 2,25E-04 |
| RAP2A | NM_021033 | Homo sapiens RAP2A, member of RAS oncogene family (RAP2A), mRNA [NM_021033] | 0,8042 | 7,96E-06 | 2,25E-04 |
| ALG2 | NM_033087 | Homo sapiens asparagine-linked glycosylation 2 homolog (S. cerevisiae, alpha-1,3-mannosyltransferase) (ALG2), mRNA [NM_033087] | 0,6819 | 8,41E-06 | 2,36E-04 |
| EB386378 | EB386378 | nbj15e01.y1 Human optic nerve. Unnormalized (nbj) Homo sapiens cDNA clone nbj15e01 5', mRNA sequence [EB386378] | 0,8010 | 8,46E-06 | 2,37E-04 |
| CR601260 | CR601260 | full-length cDNA clone CS0DM001YA20 of Fetal liver of Homo sapiens (human). [CR601260] | 1,5731 | 8,57E-06 | 2,40E-04 |
| EDG1 | NM_001400 | Homo sapiens endothelial differentiation, sphingolipid G-protein-coupled receptor, 1 (EDG1), mRNA [NM_001400] | 1,4797 | 8,75E-06 | 2,44E-04 |
| KIFC1 | NM_002263 | Homo sapiens kinesin family member C1 (KIFC1), mRNA [NM_002263] | 0,9444 | 8,74E-06 | 2,44E-04 |
| A_24_P384119 | A_24_P384119 | Unknown | 1,4400 | 9,22E-06 | 2,57E-04 |
| FLJ20105 | NM_001009954 | Homo sapiens FLJ20105 protein (FLJ20105), transcript variant 2, mRNA [NM_001009954] | 0,6638 | 9,35E-06 | 2,60E-04 |
| SPN | NM_001030288 | Homo sapiens sialophorin (leukosialin, CD43) (SPN), transcript variant 1, mRNA [NM_001030288] | 1,0309 | 9,61E-06 | 2,66E-04 |
| TMEM56 | NM_152487 | Homo sapiens transmembrane protein 56 (TMEM56), mRNA [NM_152487] | 0,9788 | 9,68E-06 | 2,67E-04 |
| LOC388588 | ENST00000378266 | Homo sapiens, clone IMAGE:5162922, mRNA. [BC035379] | 1,8230 | 9,74E-06 | 2,68E-04 |
| AK095108 | AK095108 | Homo sapiens cDNA FLJ37789 fis, clone BRHIP3000081. [AK095108] | 0,5735 | 9,85E-06 | 2,71E-04 |
| ZYG11BL | NM_006336 | Homo sapiens zyg-11 homolog B (C. elegans)-like (ZYG11BL), mRNA [NM_006336] | 0,7868 | 1,01E-05 | 2,76E-04 |
| C20orf11 | NM_017896 | Homo sapiens chromosome 20 open reading frame 11 (C20orf11), mRNA [NM_017896] | 0,8810 | 1,02E-05 | 2,79E-04 |
| CAT | NM_001752 | Homo sapiens catalase (CAT), mRNA [NM_001752] | 0,8584 | 1,02E-05 | 2,80E-04 |
| SAMM50 | NM_015380 | Homo sapiens sorting and assembly machinery component 50 homolog (S. cerevisiae) (SAMM50), mRNA [NM_015380] | 0,8714 | 1,05E-05 | 2,87E-04 |
| KLHDC8B | NM_173546 | Homo sapiens kelch domain containing 8B (KLHDC8B), mRNA [NM_173546] | 0,6993 | 1,06E-05 | 2,88E-04 |
| SLC43A1 | NM_003627 | Homo sapiens solute carrier family 43, member 1 (SLC43A1), mRNA [NM_003627] | 0,9847 | 1,09E-05 | 2,96E-04 |
| PQLC1 | AK026031 | Homo sapiens cDNA: FLJ22378 fis, clone HRC07430. [AK026031] | 0,9850 | 1,14E-05 | 3,08E-04 |
| C18orf24 | NM_001039535 | Homo sapiens chromosome 18 open reading frame 24 (C18orf24), transcript variant 1, mRNA [NM_001039535] | 0,9741 | 1,16E-05 | 3,12E-04 |
| NUTF2 | NM_005796 | Homo sapiens nuclear transport factor 2 (NUTF2), mRNA [NM_005796] | 0,6778 | 1,19E-05 | 3,18E-04 |
| TERF2IP | NM_018975 | Homo sapiens telomeric repeat binding factor 2, interacting protein (TERF2IP), mRNA [NM_018975] | 0,9083 | 1,19E-05 | 3,18E-04 |
| TTLL12 | NM_015140 | Homo sapiens tubulin tyrosine ligase-like family, member 12 (TTLL12), mRNA [NM_015140] | 0,8278 | 1,19E-05 | 3,18E-04 |
| A_24_P799580 | A_24_P799580 | Unknown | 0,7405 | 1,20E-05 | 3,20E-04 |
| FBL | NM_001436 | Homo sapiens fibrillarin (FBL), mRNA [NM_001436] | 0,6213 | 1,20E-05 | 3,20E-04 |
| RAPGEF2 | AB002311 | Homo sapiens mRNA for KIAA0313 gene, partial cds. [AB002311] | 1,1885 | 1,21E-05 | 3,22E-04 |
| RANBP5 | NM_002271 | Homo sapiens RAN binding protein 5 (RANBP5), mRNA [NM_002271] | 0,6803 | 1,23E-05 | 3,27E-04 |
| ELOF1 | NM_032377 | Homo sapiens elongation factor 1 homolog (S. cerevisiae) (ELOF1), mRNA [NM_032377] | 0,8371 | 1,27E-05 | 3,36E-04 |
| FZD5 | NM_003468 | Homo sapiens frizzled homolog 5 (Drosophila) (FZD5), mRNA [NM_003468] | 0,8754 | 1,29E-05 | 3,42E-04 |
| PIGC | AL035301 | H.sapiens gene from PAC 106H8. [AL035301] | 2,0855 | 1,29E-05 | 3,42E-04 |
| FN3KRP | NM_024619 | Homo sapiens fructosamine-3-kinase-related protein (FN3KRP), mRNA [NM_024619] | 0,7593 | 1,33E-05 | 3,50E-04 |
| ZNF584 | NM_173548 | Homo sapiens zinc finger protein 584 (ZNF584), mRNA [NM_173548] | 0,6321 | 1,34E-05 | 3,52E-04 |
| THC2609092 | THC2609092 | ALU1_HUMAN (P39188) Alu subfamily J sequence contamination warning entry, partial (7%) [THC2609092] | 0,7262 | 1,39E-05 | 3,65E-04 |
| BACE2 | NM_012105 | Homo sapiens beta-site APP-cleaving enzyme 2 (BACE2), transcript variant a, mRNA [NM_012105] | 1,1000 | 1,40E-05 | 3,66E-04 |
| ENG | NM_000118 | Homo sapiens endoglin (Osler-Rendu-Weber syndrome 1) (ENG), mRNA [NM_000118] | 0,9903 | 1,40E-05 | 3,66E-04 |
| THC2707284 | THC2707284 | Q214U3_RHOPA (Q214U3) Penicillin-binding protein 1C precursor, partial (3%) [THC2707284] | 1,2429 | 1,40E-05 | 3,67E-04 |
| FAH | NM_000137 | Homo sapiens fumarylacetoacetate hydrolase (fumarylacetoacetase) (FAH), mRNA [NM_000137] | 0,8470 | 1,43E-05 | 3,74E-04 |
| RAB6B | NM_016577 | Homo sapiens RAB6B, member RAS oncogene family (RAB6B), mRNA [NM_016577] | 1,5114 | 1,46E-05 | 3,81E-04 |
| PFKM | NM_000289 | Homo sapiens phosphofructokinase, muscle (PFKM), mRNA [NM_000289] | 0,9611 | 1,48E-05 | 3,83E-04 |
| CKAP2 | NM_018204 | Homo sapiens cytoskeleton associated protein 2 (CKAP2), mRNA [NM_018204] | 1,0429 | 1,50E-05 | 3,86E-04 |
| DENND4A | AL833317 | Homo sapiens mRNA; cDNA DKFZp313L197 (from clone DKFZp313L197). [AL833317] | 0,6152 | 1,51E-05 | 3,88E-04 |
| IGKC | BC073764 | Homo sapiens immunoglobulin kappa constant, mRNA (cDNA clone MGC:88771 IMAGE:4576136), complete cds. [BC073764] | 1,3251 | 1,53E-05 | 3,93E-04 |
| SELM | NM_080430 | Homo sapiens selenoprotein M (SELM), mRNA [NM_080430] | 1,0748 | 1,55E-05 | 3,98E-04 |
| SLC25A1 | NM_005984 | Homo sapiens solute carrier family 25 (mitochondrial carrier; citrate transporter), member 1 (SLC25A1), mRNA [NM_005984] | 0,6864 | 1,58E-05 | 4,03E-04 |
| TLN2 | NM_015059 | Homo sapiens talin 2 (TLN2), mRNA [NM_015059] | 0,7667 | 1,58E-05 | 4,03E-04 |
| SACS | NM_014363 | Homo sapiens spastic ataxia of Charlevoix-Saguenay (sacsin) (SACS), mRNA [NM_014363] | 0,9256 | 1,58E-05 | 4,03E-04 |
| GPR137B | AL832142 | Homo sapiens mRNA; cDNA DKFZp686A22111 (from clone DKFZp686A22111). [AL832142] | 1,3310 | 1,59E-05 | 4,04E-04 |
| KREMEN1 | NM_001039570 | Homo sapiens kringle containing transmembrane protein 1 (KREMEN1), transcript variant 3, mRNA [NM_001039570] | 0,9856 | 1,59E-05 | 4,05E-04 |
| BU940040 | BU940040 | AGENCOURT_10576766 NIH_MGC_128 Homo sapiens cDNA clone IMAGE:6709025 5', mRNA sequence [BU940040] | 0,9608 | 1,60E-05 | 4,06E-04 |
| SPTB | NM_001024858 | Homo sapiens spectrin, beta, erythrocytic (includes spherocytosis, clinical type I) (SPTB), transcript variant 1, mRNA [NM_001024858] | 0,9204 | 1,62E-05 | 4,11E-04 |
| FEM1A | NM_018708 | Homo sapiens fem-1 homolog a (C. elegans) (FEM1A), mRNA [NM_018708] | 1,1207 | 1,63E-05 | 4,12E-04 |
| ITGB5 | NM_002213 | Homo sapiens integrin, beta 5 (ITGB5), mRNA [NM_002213] | 0,7826 | 1,64E-05 | 4,14E-04 |
| RAP2B | NM_002886 | Homo sapiens RAP2B, member of RAS oncogene family (RAP2B), mRNA [NM_002886] | 0,7055 | 1,69E-05 | 4,27E-04 |
| L38427 | L38427 | Homo sapiens Ig rearranged H-chain mRNA V region. [L38427] | 1,5140 | 1,70E-05 | 4,28E-04 |
| GMPPB | NM_021971 | Homo sapiens GDP-mannose pyrophosphorylase B (GMPPB), transcript variant 2, mRNA [NM_021971] | 0,6750 | 1,75E-05 | 4,40E-04 |
| MGC11102 | NM_032325 | Homo sapiens hypothetical protein MGC11102 (MGC11102), mRNA [NM_032325] | 0,8328 | 1,76E-05 | 4,40E-04 |
| ABHD14A | NM_015407 | Homo sapiens abhydrolase domain containing 14A (ABHD14A), mRNA [NM_015407] | 1,0740 | 1,76E-05 | 4,41E-04 |
| A_24_P234871 | A_24_P234871 | Unknown | 0,8314 | 1,80E-05 | 4,48E-04 |
| PVRL1 | NM_002855 | Homo sapiens poliovirus receptor-related 1 (herpesvirus entry mediator C; nectin) (PVRL1), transcript variant 1, mRNA [NM_002855] | 0,9474 | 1,83E-05 | 4,55E-04 |
| RUVBL2 | NM_006666 | Homo sapiens RuvB-like 2 (E. coli) (RUVBL2), mRNA [NM_006666] | 1,0590 | 1,84E-05 | 4,56E-04 |
| SPECC1 | NM_152904 | Homo sapiens sperm antigen with calponin homology and coiled-coil domains 1 (SPECC1), transcript variant NSP5beta3alpha, mRNA [NM_152904] | 1,0171 | 1,85E-05 | 4,58E-04 |
| SLC25A37 | AK093931 | Homo sapiens cDNA FLJ36612 fis, clone TRACH2016131, highly similar to Homo sapiens mitochondrial solute carrier mRNA. [AK093931] | 1,0792 | 1,86E-05 | 4,59E-04 |
| POLL | NM_013274 | Homo sapiens polymerase (DNA directed), lambda (POLL), mRNA [NM_013274] | 0,6687 | 1,86E-05 | 4,60E-04 |
| CR617018 | CR617018 | full-length cDNA clone CS0DG001YH13 of B cells (Ramos cell line) of Homo sapiens (human). [CR617018] | 0,8837 | 1,89E-05 | 4,65E-04 |
| ACY1 | NM_000666 | Homo sapiens aminoacylase 1 (ACY1), mRNA [NM_000666] | 0,8214 | 1,91E-05 | 4,69E-04 |
| FOXM1 | NM_202002 | Homo sapiens forkhead box M1 (FOXM1), transcript variant 1, mRNA [NM_202002] | 0,6680 | 1,91E-05 | 4,69E-04 |
| ZBTB3 | NM_024784 | Homo sapiens zinc finger and BTB domain containing 3 (ZBTB3), mRNA [NM_024784] | 1,1234 | 1,92E-05 | 4,69E-04 |
| MTMR12 | NM_001040446 | Homo sapiens myotubularin related protein 12 (MTMR12), mRNA [NM_001040446] | 0,9113 | 1,92E-05 | 4,70E-04 |
| SLC2A4RG | NM_020062 | Homo sapiens SLC2A4 regulator (SLC2A4RG), mRNA [NM_020062] | 0,6871 | 1,98E-05 | 4,81E-04 |
| METAP2 | NM_006838 | Homo sapiens methionyl aminopeptidase 2 (METAP2), mRNA [NM_006838] | 0,8950 | 2,02E-05 | 4,89E-04 |
| A_23_P159163 | A_23_P159163 | Unknown | 0,9922 | 2,04E-05 | 4,93E-04 |
| AP2B1 | NM_001030006 | Homo sapiens adaptor-related protein complex 2, beta 1 subunit (AP2B1), transcript variant 1, mRNA [NM_001030006] | 0,7744 | 2,05E-05 | 4,96E-04 |
| TSFM | NM_005726 | Homo sapiens Ts translation elongation factor, mitochondrial (TSFM), mRNA [NM_005726] | 0,7265 | 2,06E-05 | 4,98E-04 |
| POLR2I | NM_006233 | Homo sapiens polymerase (RNA) II (DNA directed) polypeptide I, 14.5kDa (POLR2I), mRNA [NM_006233] | 0,7358 | 2,10E-05 | 5,06E-04 |
| ARL4A | NM_005738 | Homo sapiens ADP-ribosylation factor-like 4A (ARL4A), transcript variant 1, mRNA [NM_005738] | 0,8819 | 2,13E-05 | 5,12E-04 |
| HPS6 | NM_024747 | Homo sapiens Hermansky-Pudlak syndrome 6 (HPS6), mRNA [NM_024747] | 1,3919 | 2,18E-05 | 5,23E-04 |
| FAM104A | NM_032837 | Homo sapiens family with sequence similarity 104, member A (FAM104A), mRNA [NM_032837] | 1,1070 | 2,21E-05 | 5,28E-04 |
| ICAM4 | NM_001544 | Homo sapiens intercellular adhesion molecule 4 (Landsteiner-Wiener blood group) (ICAM4), transcript variant 1, mRNA [NM_001544] | 0,9040 | 2,21E-05 | 5,29E-04 |
| PKLR | NM_000298 | Homo sapiens pyruvate kinase, liver and RBC (PKLR), nuclear gene encoding mitochondrial protein, transcript variant 1, mRNA [NM_000298] | 1,0305 | 2,22E-05 | 5,31E-04 |
| MOBKL2C | NM_145279 | Homo sapiens MOB1, Mps One Binder kinase activator-like 2C (yeast) (MOBKL2C), transcript variant 1, mRNA [NM_145279] | 0,8000 | 2,25E-05 | 5,37E-04 |
| ZNF175 | NM_007147 | Homo sapiens zinc finger protein 175 (ZNF175), mRNA [NM_007147] | 0,6637 | 2,27E-05 | 5,40E-04 |
| C6orf89 | NM_152734 | Homo sapiens chromosome 6 open reading frame 89 (C6orf89), mRNA [NM_152734] | 0,6487 | 2,28E-05 | 5,40E-04 |
| A_24_P325533 | A_24_P325533 | Unknown | 0,8897 | 2,28E-05 | 5,41E-04 |
| A_24_P281504 | A_24_P281504 | Unknown | 0,7726 | 2,29E-05 | 5,42E-04 |
| BNIP3L | NM_004331 | Homo sapiens BCL2/adenovirus E1B 19kDa interacting protein 3-like (BNIP3L), mRNA [NM_004331] | 1,2969 | 2,31E-05 | 5,46E-04 |
| PECI | NM_206836 | Homo sapiens peroxisomal D3,D2-enoyl-CoA isomerase (PECI), transcript variant 2, mRNA [NM_206836] | 0,8509 | 2,33E-05 | 5,50E-04 |
| PYCR2 | NM_013328 | Homo sapiens pyrroline-5-carboxylate reductase family, member 2 (PYCR2), mRNA [NM_013328] | 0,7015 | 2,33E-05 | 5,50E-04 |
| FAM122A | NM_138333 | Homo sapiens family with sequence similarity 122A (FAM122A), mRNA [NM_138333] | 0,8097 | 2,34E-05 | 5,51E-04 |
| CDC20 | NM_001255 | Homo sapiens cell division cycle 20 homolog (S. cerevisiae) (CDC20), mRNA [NM_001255] | 0,8885 | 2,35E-05 | 5,53E-04 |
| RNASE1 | NM_198232 | Homo sapiens ribonuclease, RNase A family, 1 (pancreatic) (RNASE1), transcript variant 3, mRNA [NM_198232] | 0,7501 | 2,36E-05 | 5,53E-04 |
| THC2689491 | THC2689491 | Unknown | 1,1411 | 2,36E-05 | 5,53E-04 |
| VDAC3 | NM_005662 | Homo sapiens voltage-dependent anion channel 3 (VDAC3), mRNA [NM_005662] | 0,6790 | 2,36E-05 | 5,53E-04 |
| MRPL37 | NM_016491 | Homo sapiens mitochondrial ribosomal protein L37 (MRPL37), nuclear gene encoding mitochondrial protein, mRNA [NM_016491] | 0,7343 | 2,39E-05 | 5,59E-04 |
| C14orf130 | NM_018108 | Homo sapiens chromosome 14 open reading frame 130 (C14orf130), transcript variant 1, mRNA [NM_018108] | 0,6955 | 2,40E-05 | 5,61E-04 |
| DAG1 | NM_004393 | Homo sapiens dystroglycan 1 (dystrophin-associated glycoprotein 1) (DAG1), mRNA [NM_004393] | 0,6434 | 2,42E-05 | 5,64E-04 |
| E2F2 | NM_004091 | Homo sapiens E2F transcription factor 2 (E2F2), mRNA [NM_004091] | 1,1980 | 2,42E-05 | 5,64E-04 |
| BG547557 | BG547557 | BG547557 602575410F1 NIH_MGC_77 Homo sapiens cDNA clone IMAGE:4703546 5', mRNA sequence [BG547557] | 1,3234 | 2,45E-05 | 5,69E-04 |
| RPN2 | NM_002951 | Homo sapiens ribophorin II (RPN2), mRNA [NM_002951] | 0,9185 | 2,45E-05 | 5,69E-04 |
| APEX2 | NM_014481 | Homo sapiens APEX nuclease (apurinic/apyrimidinic endonuclease) 2 (APEX2), nuclear gene encoding mitochondrial protein, mRNA [NM_014481] | 1,0401 | 2,49E-05 | 5,76E-04 |
| AKAP7 | NM_016377 | Homo sapiens A kinase (PRKA) anchor protein 7 (AKAP7), transcript variant gamma, mRNA [NM_016377] | 0,8173 | 2,50E-05 | 5,79E-04 |
| GCDH | NM_013976 | Homo sapiens glutaryl-Coenzyme A dehydrogenase (GCDH), nuclear gene encoding mitochondrial protein, transcript variant 2, mRNA [NM_013976] | 0,7946 | 2,52E-05 | 5,82E-04 |
| RNF14 | NM_004290 | Homo sapiens ring finger protein 14 (RNF14), transcript variant 1, mRNA [NM_004290] | 0,7741 | 2,55E-05 | 5,87E-04 |
| DAAM1 | NM_014992 | Homo sapiens dishevelled associated activator of morphogenesis 1 (DAAM1), mRNA [NM_014992] | 0,9019 | 2,56E-05 | 5,89E-04 |
| ALAD | NM_001003945 | Homo sapiens aminolevulinate, delta-, dehydratase (ALAD), transcript variant 1, mRNA [NM_001003945] | 1,2603 | 2,65E-05 | 6,05E-04 |
| SLC29A1 | NM_001078177 | Homo sapiens solute carrier family 29 (nucleoside transporters), member 1 (SLC29A1), nuclear gene encoding mitochondrial protein, transcript variant 1, mRNA [NM_001078177] | 1,1321 | 2,65E-05 | 6,06E-04 |
| TMED10 | NM_006827 | Homo sapiens transmembrane emp24-like trafficking protein 10 (yeast) (TMED10), mRNA [NM_006827] | 0,9524 | 2,67E-05 | 6,08E-04 |
| IFI6 | BC024289 | Homo sapiens interferon, alpha-inducible protein 6, mRNA (cDNA clone MGC:39273 IMAGE:5440834), complete cds. [BC024289] | 1,2113 | 2,72E-05 | 6,19E-04 |
| NME4 | NM_005009 | Homo sapiens non-metastatic cells 4, protein expressed in (NME4), mRNA [NM_005009] | 1,1441 | 2,72E-05 | 6,19E-04 |
| HTRA1 | NM_002775 | Homo sapiens HtrA serine peptidase 1 (HTRA1), mRNA [NM_002775] | 0,7639 | 2,75E-05 | 6,24E-04 |
| AK1 | NM_000476 | Homo sapiens adenylate kinase 1 (AK1), mRNA [NM_000476] | 1,4821 | 2,78E-05 | 6,29E-04 |
| MTMR2 | NM_201278 | Homo sapiens myotubularin related protein 2 (MTMR2), transcript variant 2, mRNA [NM_201278] | 0,6374 | 2,79E-05 | 6,31E-04 |
| TIMP3 | NM_000362 | Homo sapiens TIMP metallopeptidase inhibitor 3 (Sorsby fundus dystrophy, pseudoinflammatory) (TIMP3), mRNA [NM_000362] | 0,6417 | 2,81E-05 | 6,34E-04 |
| BC039479 | BC039479 | Homo sapiens, clone IMAGE:5534210, mRNA. [BC039479] | 0,5290 | 2,82E-05 | 6,36E-04 |
| SMC3 | NM_005445 | Homo sapiens structural maintenance of chromosomes 3 (SMC3), mRNA [NM_005445] | 0,5614 | 2,82E-05 | 6,36E-04 |
| EPRS | NM_004446 | Homo sapiens glutamyl-prolyl-tRNA synthetase (EPRS), mRNA [NM_004446] | 0,7297 | 2,87E-05 | 6,44E-04 |
| PPP2R4 | NM_178001 | Homo sapiens protein phosphatase 2A, regulatory subunit B' (PR 53) (PPP2R4), transcript variant 1, mRNA [NM_178001] | 0,8514 | 2,88E-05 | 6,47E-04 |
| MFSD5 | NM_032889 | Homo sapiens major facilitator superfamily domain containing 5 (MFSD5), mRNA [NM_032889] | 1,2575 | 2,89E-05 | 6,49E-04 |
| IGLL1 | NM_020070 | Homo sapiens immunoglobulin lambda-like polypeptide 1 (IGLL1), transcript variant 1, mRNA [NM_020070] | 1,6551 | 2,90E-05 | 6,49E-04 |
| THC2532155 | THC2532155 | Q8K2W0_MOUSE (Q8K2W0) Procollagen, type IX, alpha 2, partial (3%) [THC2532155] | 0,9438 | 2,96E-05 | 6,60E-04 |
| COMT | NM_000754 | Homo sapiens catechol-O-methyltransferase (COMT), transcript variant MB-COMT, mRNA [NM_000754] | 0,8194 | 2,96E-05 | 6,61E-04 |
| PIP5K1B | NM_003558 | Homo sapiens phosphatidylinositol-4-phosphate 5-kinase, type I, beta (PIP5K1B), transcript variant 2, mRNA [NM_003558] | 1,1326 | 2,99E-05 | 6,65E-04 |
| QSCN6L1 | NM_181701 | Homo sapiens quiescin Q6-like 1 (QSCN6L1), mRNA [NM_181701] | 0,8133 | 3,03E-05 | 6,71E-04 |
| PCAF | NM_003884 | Homo sapiens p300/CBP-associated factor (PCAF), mRNA [NM_003884] | 0,9057 | 3,05E-05 | 6,75E-04 |
| A_32_P19460 | A_32_P19460 | Unknown | 0,6833 | 3,06E-05 | 6,76E-04 |
| GOT1 | NM_002079 | Homo sapiens glutamic-oxaloacetic transaminase 1, soluble (aspartate aminotransferase 1) (GOT1), mRNA [NM_002079] | 0,5384 | 3,07E-05 | 6,76E-04 |
| AIFM2 | NM_032797 | Homo sapiens apoptosis-inducing factor, mitochondrion-associated, 2 (AIFM2), mRNA [NM_032797] | 0,8298 | 3,07E-05 | 6,77E-04 |
| A_24_P349869 | A_24_P349869 | Unknown | 1,0433 | 3,16E-05 | 6,91E-04 |
| FLJ30092 | AB014514 | Homo sapiens mRNA for KIAA0614 protein, partial cds. [AB014514] | 1,1761 | 3,16E-05 | 6,91E-04 |
| PDAP1 | NM_014891 | Homo sapiens PDGFA associated protein 1 (PDAP1), mRNA [NM_014891] | 1,3121 | 3,19E-05 | 6,97E-04 |
| FKBP8 | NM_012181 | Homo sapiens FK506 binding protein 8, 38kDa (FKBP8), mRNA [NM_012181] | 0,8517 | 3,20E-05 | 6,97E-04 |
| POLR3H | NM_001018052 | Homo sapiens polymerase (RNA) III (DNA directed) polypeptide H (22.9kD) (POLR3H), transcript variant 2, mRNA [NM_001018052] | 0,7591 | 3,21E-05 | 6,99E-04 |
| ZNF526 | NM_133444 | Homo sapiens zinc finger protein 526 (ZNF526), mRNA [NM_133444] | 0,9427 | 3,22E-05 | 7,00E-04 |
| ENST00000377226 | ENST00000377226 | Homo sapiens isolate D3-P-4-K-39 immunoglobulin light chain variable region mRNA, partial cds. [DQ841033] | 1,8316 | 3,24E-05 | 7,02E-04 |
| GCLM | ENST00000370238 | Glutamate--cysteine ligase regulatory subunit (EC 6.3.2.2) (Gamma- glutamylcysteine synthetase) (Gamma-ECS) (GCS light chain) (Glutamate--cysteine ligase modifier subunit). [Source:Uniprot/SWISSPROT;Acc:P48507] [ENST00000370238] | 1,3234 | 3,24E-05 | 7,02E-04 |
| YIF1A | NM_020470 | Homo sapiens Yip1 interacting factor homolog A (S. cerevisiae) (YIF1A), mRNA [NM_020470] | 0,8450 | 3,25E-05 | 7,02E-04 |
| LBH | NM_030915 | Homo sapiens limb bud and heart development homolog (mouse) (LBH), mRNA [NM_030915] | 0,8108 | 3,26E-05 | 7,04E-04 |
| FARSLA | NM_004461 | Homo sapiens phenylalanine-tRNA synthetase-like, alpha subunit (FARSLA), mRNA [NM_004461] | 0,5902 | 3,28E-05 | 7,07E-04 |
| CXorf6 | NM_005491 | Homo sapiens chromosome X open reading frame 6 (CXorf6), mRNA [NM_005491] | 0,7872 | 3,33E-05 | 7,15E-04 |
| A_23_P112957 | A_23_P112957 | Unknown | 1,2222 | 3,36E-05 | 7,21E-04 |
| GNPDA1 | NM_005471 | Homo sapiens glucosamine-6-phosphate deaminase 1 (GNPDA1), mRNA [NM_005471] | 0,6915 | 3,40E-05 | 7,27E-04 |
| YBX1 | NM_004559 | Homo sapiens Y box binding protein 1 (YBX1), mRNA [NM_004559] | 0,6024 | 3,41E-05 | 7,30E-04 |
| LOC283666 | BC048264 | Homo sapiens hypothetical protein LOC283666, mRNA (cDNA clone IMAGE:4415549), partial cds. [BC048264] | 1,3437 | 3,42E-05 | 7,30E-04 |
| ASF1A | NM_014034 | Homo sapiens ASF1 anti-silencing function 1 homolog A (S. cerevisiae) (ASF1A), mRNA [NM_014034] | 0,8230 | 3,43E-05 | 7,31E-04 |
| FAM127B | NM_001078172 | Homo sapiens family with sequence similarity 127, member B (FAM127B), mRNA [NM_001078172] | 0,6353 | 3,48E-05 | 7,40E-04 |
| DOLPP1 | NM_020438 | Homo sapiens dolichyl pyrophosphate phosphatase 1 (DOLPP1), mRNA [NM_020438] | 0,6643 | 3,49E-05 | 7,41E-04 |
| JAKMIP1 | NM_144720 | Homo sapiens janus kinase and microtubule interacting protein 1 (JAKMIP1), mRNA [NM_144720] | 1,0606 | 3,49E-05 | 7,41E-04 |
| PIGW | NM_178517 | Homo sapiens phosphatidylinositol glycan anchor biosynthesis, class W (PIGW), mRNA [NM_178517] | 0,6333 | 3,50E-05 | 7,42E-04 |
| A_23_P158868 | A_23_P158868 | Unknown | 1,5256 | 3,55E-05 | 7,51E-04 |
| ZNF23 | NM_145911 | Homo sapiens zinc finger protein 23 (KOX 16) (ZNF23), mRNA [NM_145911] | 0,8496 | 3,56E-05 | 7,54E-04 |
| TFRC | NM_003234 | Homo sapiens transferrin receptor (p90, CD71) (TFRC), mRNA [NM_003234] | 1,5062 | 3,60E-05 | 7,61E-04 |
| ZNF16 | NM_001029976 | Homo sapiens zinc finger protein 16 (ZNF16), transcript variant 2, mRNA [NM_001029976] | 0,5787 | 3,60E-05 | 7,61E-04 |
| ICMT | NM_012405 | Homo sapiens isoprenylcysteine carboxyl methyltransferase (ICMT), mRNA [NM_012405] | 1,0710 | 3,61E-05 | 7,63E-04 |
| AF076205 | AF076205 | Homo sapiens rheumatoid arthritis patient C355-6, immunoglobulin lambda light chain variable region (V4b) mRNA, partial cds. [AF076205] | 1,4211 | 3,64E-05 | 7,68E-04 |
| USP14 | NM_005151 | Homo sapiens ubiquitin specific peptidase 14 (tRNA-guanine transglycosylase) (USP14), transcript variant 1, mRNA [NM_005151] | 0,8390 | 3,81E-05 | 8,01E-04 |
| MTHFD1 | NM_005956 | Homo sapiens methylenetetrahydrofolate dehydrogenase (NADP+ dependent) 1, methenyltetrahydrofolate cyclohydrolase, formyltetrahydrofolate synthetase (MTHFD1), mRNA [NM_005956] | 0,6870 | 3,84E-05 | 8,05E-04 |
| RFX2 | NM_000635 | Homo sapiens regulatory factor X, 2 (influences HLA class II expression) (RFX2), transcript variant 1, mRNA [NM_000635] | 0,8772 | 3,86E-05 | 8,08E-04 |
| SLC25A42 | NM_178526 | Homo sapiens solute carrier family 25, member 42 (SLC25A42), mRNA [NM_178526] | 0,5930 | 3,91E-05 | 8,15E-04 |
| AP2A1 | NM_014203 | Homo sapiens adaptor-related protein complex 2, alpha 1 subunit (AP2A1), transcript variant 1, mRNA [NM_014203] | 0,9441 | 3,91E-05 | 8,15E-04 |
| KIAA1344 | NM_020784 | Homo sapiens KIAA1344 (KIAA1344), mRNA [NM_020784] | 0,6767 | 3,97E-05 | 8,25E-04 |
| PRKAR2B | NM_002736 | Homo sapiens protein kinase, cAMP-dependent, regulatory, type II, beta (PRKAR2B), mRNA [NM_002736] | 1,2076 | 4,00E-05 | 8,31E-04 |
| SOD1 | NM_000454 | Homo sapiens superoxide dismutase 1, soluble (amyotrophic lateral sclerosis 1 (adult)) (SOD1), mRNA [NM_000454] | 0,7041 | 4,15E-05 | 8,55E-04 |
| APEH | NM_001640 | Homo sapiens N-acylaminoacyl-peptide hydrolase (APEH), mRNA [NM_001640] | 0,8662 | 4,16E-05 | 8,58E-04 |
| FRZB | NM_001463 | Homo sapiens frizzled-related protein (FRZB), mRNA [NM_001463] | 0,8276 | 4,18E-05 | 8,61E-04 |
| UBXD3 | NM_152376 | Homo sapiens UBX domain containing 3 (UBXD3), mRNA [NM_152376] | 1,1462 | 4,22E-05 | 8,68E-04 |
| IMPDH2 | NM_000884 | Homo sapiens IMP (inosine monophosphate) dehydrogenase 2 (IMPDH2), mRNA [NM_000884] | 0,7579 | 4,23E-05 | 8,69E-04 |
| PPOX | NM_000309 | Homo sapiens protoporphyrinogen oxidase (PPOX), nuclear gene encoding mitochondrial protein, mRNA [NM_000309] | 1,0243 | 4,24E-05 | 8,70E-04 |
| A_32_P214565 | A_32_P214565 | Unknown | 0,8985 | 4,24E-05 | 8,70E-04 |
| MAZ | NM_001042539 | Homo sapiens MYC-associated zinc finger protein (purine-binding transcription factor) (MAZ), transcript variant 2, mRNA [NM_001042539] | 0,8530 | 4,27E-05 | 8,73E-04 |
| VTI1B | NM_006370 | Homo sapiens vesicle transport through interaction with t-SNAREs homolog 1B (yeast) (VTI1B), mRNA [NM_006370] | 0,8422 | 4,27E-05 | 8,73E-04 |
| WHSC1 | NM_133330 | Homo sapiens Wolf-Hirschhorn syndrome candidate 1 (WHSC1), transcript variant 1, mRNA [NM_133330] | 0,8472 | 4,27E-05 | 8,73E-04 |
| KIAA0406 | NM_014657 | Homo sapiens KIAA0406 (KIAA0406), mRNA [NM_014657] | 0,6145 | 4,41E-05 | 8,99E-04 |
| CD8B | NM_172102 | Homo sapiens CD8b molecule (CD8B), transcript variant 4, mRNA [NM_172102] | 0,9519 | 4,53E-05 | 9,22E-04 |
| GPAA1 | NM_003801 | Homo sapiens glycosylphosphatidylinositol anchor attachment protein 1 homolog (yeast) (GPAA1), mRNA [NM_003801] | 0,7841 | 4,56E-05 | 9,27E-04 |
| CDC25A | NM_001789 | Homo sapiens cell division cycle 25 homolog A (S. cerevisiae) (CDC25A), transcript variant 1, mRNA [NM_001789] | 0,6253 | 4,57E-05 | 9,28E-04 |
| A_24_P186354 | A_24_P186354 | Unknown | 0,8127 | 4,68E-05 | 9,47E-04 |
| FEN1 | NM_004111 | Homo sapiens flap structure-specific endonuclease 1 (FEN1), mRNA [NM_004111] | 1,1074 | 4,69E-05 | 9,47E-04 |
| C14orf32 | NM_144578 | Homo sapiens chromosome 14 open reading frame 32 (C14orf32), mRNA [NM_144578] | 0,7902 | 4,73E-05 | 9,53E-04 |
| CDCA4 | NM_017955 | Homo sapiens cell division cycle associated 4 (CDCA4), transcript variant 13, mRNA [NM_017955] | 0,8150 | 4,74E-05 | 9,55E-04 |
| GTF3C4 | NM_012204 | Homo sapiens general transcription factor IIIC, polypeptide 4, 90kDa (GTF3C4), mRNA [NM_012204] | 0,8344 | 4,76E-05 | 9,56E-04 |
| AY003763 | AY003763 | Homo sapiens isolate sy-3A/17-G9 immunoglobulin alpha heavy chain variable region mRNA, partial cds. [AY003763] | 1,6553 | 4,78E-05 | 9,60E-04 |
| LAGE3 | NM_006014 | Homo sapiens L antigen family, member 3 (LAGE3), mRNA [NM_006014] | 0,8436 | 4,82E-05 | 9,64E-04 |
| THRAP4 | NM_014815 | Homo sapiens thyroid hormone receptor associated protein 4 (THRAP4), transcript variant 1, mRNA [NM_014815] | 0,6214 | 4,93E-05 | 9,85E-04 |
| CAPN1 | NM_005186 | Homo sapiens calpain 1, (mu/I) large subunit (CAPN1), mRNA [NM_005186] | 0,9797 | 4,94E-05 | 9,85E-04 |
| RP11-529I10.4 | NM_015448 | Homo sapiens deleted in a mouse model of primary ciliary dyskinesia (RP11-529I10.4), mRNA [NM_015448] | 0,8119 | 4,97E-05 | 9,91E-04 |
| C5orf30 | NM_033211 | Homo sapiens chromosome 5 open reading frame 30 (C5orf30), mRNA [NM_033211] | 0,9369 | 5,00E-05 | 9,93E-04 |
| C9orf58 | NM_001002260 | Homo sapiens chromosome 9 open reading frame 58 (C9orf58), transcript variant 2, mRNA [NM_001002260] | 0,8895 | 5,03E-05 | 9,98E-04 |
| MGC27348 | BC026177 | Homo sapiens ribosomal protein S2 pseudogene, mRNA (cDNA clone MGC:27348 IMAGE:4671259), complete cds. [BC026177] | 0,7744 | 5,04E-05 | 9,99E-04 |
| SDF4 | NM_016176 | Homo sapiens stromal cell derived factor 4 (SDF4), mRNA [NM_016176] | 0,6411 | 5,06E-05 | 1,00E-03 |
| ITGB1 | NM_002211 | Homo sapiens integrin, beta 1 (fibronectin receptor, beta polypeptide, antigen CD29 includes MDF2, MSK12) (ITGB1), transcript variant 1A, mRNA [NM_002211] | 0,7150 | 5,08E-05 | 1,01E-03 |
| NAG | NM_015909 | Homo sapiens neuroblastoma-amplified protein (NAG), mRNA [NM_015909] | 1,0131 | 5,11E-05 | 1,01E-03 |
| FAM13A1 | NM_014883 | Homo sapiens family with sequence similarity 13, member A1 (FAM13A1), transcript variant 1, mRNA [NM_014883] | 0,7307 | 5,16E-05 | 1,02E-03 |
| PRDX4 | NM_006406 | Homo sapiens peroxiredoxin 4 (PRDX4), mRNA [NM_006406] | 0,8106 | 5,16E-05 | 1,02E-03 |
| NDUFS2 | NM_004550 | Homo sapiens NADH dehydrogenase (ubiquinone) Fe-S protein 2, 49kDa (NADH-coenzyme Q reductase) (NDUFS2), mRNA [NM_004550] | 0,7340 | 5,18E-05 | 1,02E-03 |
| LOC645000 | XR_016848 | PREDICTED: Homo sapiens similar to 40S ribosomal protein S3 (LOC645000), mRNA [XR_016848] | 0,8013 | 5,26E-05 | 1,03E-03 |
| ABCF1 | NM_001025091 | Homo sapiens ATP-binding cassette, sub-family F (GCN20), member 1 (ABCF1), transcript variant 1, mRNA [NM_001025091] | 0,8944 | 5,28E-05 | 1,04E-03 |
| TUBA6 | NM_032704 | Homo sapiens tubulin, alpha 6 (TUBA6), mRNA [NM_032704] | 0,7648 | 5,34E-05 | 1,04E-03 |
| ACOT1 | NM_001037161 | Homo sapiens acyl-CoA thioesterase 1 (ACOT1), mRNA [NM_001037161] | 0,7535 | 5,35E-05 | 1,05E-03 |
| VPS37C | NM_017966 | Homo sapiens vacuolar protein sorting 37 homolog C (S. cerevisiae) (VPS37C), mRNA [NM_017966] | 0,6855 | 5,36E-05 | 1,05E-03 |
| SELS | NM_018445 | Homo sapiens selenoprotein S (SELS), transcript variant 2, mRNA [NM_018445] | 0,6792 | 5,44E-05 | 1,06E-03 |
| SIGLECP3 | NR_002804 | Homo sapiens sialic acid binding Ig-like lectin, pseudogene 3 (SIGLECP3) on chromosome 19 [NR_002804] | 0,7332 | 5,44E-05 | 1,06E-03 |
| SAPS2 | XM_001129019 | PREDICTED: Homo sapiens SAPS domain family, member 2 (SAPS2), mRNA [XM_001129019] | 0,7115 | 5,50E-05 | 1,07E-03 |
| BOLA3 | NM_212552 | Homo sapiens bolA homolog 3 (E. coli) (BOLA3), transcript variant 1, mRNA [NM_212552] | 0,9476 | 5,51E-05 | 1,07E-03 |
| SLC22A4 | NM_003059 | Homo sapiens solute carrier family 22 (organic cation transporter), member 4 (SLC22A4), mRNA [NM_003059] | 0,9487 | 5,59E-05 | 1,08E-03 |
| TCEA1 | NM_006756 | Homo sapiens transcription elongation factor A (SII), 1 (TCEA1), transcript variant 1, mRNA [NM_006756] | 0,8034 | 5,71E-05 | 1,10E-03 |
| ENDOG | NM_004435 | Homo sapiens endonuclease G (ENDOG), nuclear gene encoding mitochondrial protein, mRNA [NM_004435] | 0,7689 | 5,73E-05 | 1,10E-03 |
| X92493 | X92493 | H.sapiens mRNA for STM-7 protein. [X92493] | 0,9353 | 5,77E-05 | 1,11E-03 |
| WBSCR16 | NM_030798 | Homo sapiens Williams-Beuren syndrome chromosome region 16 (WBSCR16), mRNA [NM_030798] | 0,8267 | 5,80E-05 | 1,11E-03 |
| AF343666 | AF343666 | Homo sapiens translocation associated fusion protein IRTA1/IGA1 (IRTA1/IGHA1) mRNA, complete cds. [AF343666] | 1,2924 | 5,95E-05 | 1,14E-03 |
| NUP188 | NM_015354 | Homo sapiens nucleoporin 188kDa (NUP188), mRNA [NM_015354] | 0,7369 | 6,00E-05 | 1,14E-03 |
| AK023559 | AK023559 | Homo sapiens cDNA FLJ13497 fis, clone PLACE1004518. [AK023559] | 0,6907 | 6,00E-05 | 1,14E-03 |
| BIRC5 | NM_001012271 | Homo sapiens baculoviral IAP repeat-containing 5 (survivin) (BIRC5), transcript variant 3, mRNA [NM_001012271] | 0,7943 | 6,03E-05 | 1,15E-03 |
| C1orf109 | NM_017850 | Homo sapiens chromosome 1 open reading frame 109 (C1orf109), mRNA [NM_017850] | 0,7916 | 6,10E-05 | 1,16E-03 |
| PLEKHF1 | NM_024310 | Homo sapiens pleckstrin homology domain containing, family F (with FYVE domain) member 1 (PLEKHF1), mRNA [NM_024310] | 0,7759 | 6,15E-05 | 1,17E-03 |
| RBX1 | NM_014248 | Homo sapiens ring-box 1 (RBX1), mRNA [NM_014248] | 0,8069 | 6,15E-05 | 1,17E-03 |
| EIF3S9 | NM_001037283 | Homo sapiens eukaryotic translation initiation factor 3, subunit 9 eta, 116kDa (EIF3S9), transcript variant 2, mRNA [NM_001037283] | 0,7044 | 6,25E-05 | 1,18E-03 |
| AK022030 | AK022030 | Homo sapiens cDNA FLJ11968 fis, clone HEMBB1001133. [AK022030] | 0,7414 | 6,31E-05 | 1,19E-03 |
| NDUFC2 | NM_004549 | Homo sapiens NADH dehydrogenase (ubiquinone) 1, subcomplex unknown, 2, 14.5kDa (NDUFC2), mRNA [NM_004549] | 0,6335 | 6,33E-05 | 1,20E-03 |
| ADIPOR1 | NM_015999 | Homo sapiens adiponectin receptor 1 (ADIPOR1), mRNA [NM_015999] | 1,2802 | 6,39E-05 | 1,21E-03 |
| BCL2 | NM_000633 | Homo sapiens B-cell CLL/lymphoma 2 (BCL2), nuclear gene encoding mitochondrial protein, transcript variant alpha, mRNA [NM_000633] | 1,0808 | 6,40E-05 | 1,21E-03 |
| PCNA | NM_002592 | Homo sapiens proliferating cell nuclear antigen (PCNA), transcript variant 1, mRNA [NM_002592] | 0,7527 | 6,40E-05 | 1,21E-03 |
| ACSBG1 | NM_015162 | Homo sapiens acyl-CoA synthetase bubblegum family member 1 (ACSBG1), mRNA [NM_015162] | 0,7413 | 6,52E-05 | 1,22E-03 |
| ENST00000355691 | ENST00000355691 | Copper-transporting ATPase 1 (EC 3.6.3.4) (Copper pump 1) (Menkes disease-associated protein). [Source:Uniprot/SWISSPROT;Acc:Q04656] [ENST00000355691] | 0,7891 | 6,51E-05 | 1,22E-03 |
| ZNF543 | NM_213598 | Homo sapiens zinc finger protein 543 (ZNF543), mRNA [NM_213598] | 0,5998 | 6,51E-05 | 1,22E-03 |
| AK026372 | AK026372 | Homo sapiens cDNA: FLJ22719 fis, clone HSI14307. [AK026372] | 0,9605 | 6,55E-05 | 1,23E-03 |
| DCK | NM_000788 | Homo sapiens deoxycytidine kinase (DCK), mRNA [NM_000788] | 1,0909 | 6,55E-05 | 1,23E-03 |
| PRPF19 | NM_014502 | Homo sapiens PRP19/PSO4 pre-mRNA processing factor 19 homolog (S. cerevisiae) (PRPF19), mRNA [NM_014502] | 0,9257 | 6,57E-05 | 1,23E-03 |
| THC2539698 | THC2539698 | Q59GN2_HUMAN (Q59GN2) Ribosomal protein L39 variant (Fragment), partial (77%) [THC2539698] | 0,5628 | 6,58E-05 | 1,23E-03 |
| CD248 | NM_020404 | Homo sapiens CD248 molecule, endosialin (CD248), mRNA [NM_020404] | 0,9437 | 6,75E-05 | 1,26E-03 |
| THC2685096 | THC2685096 | Q8HNY5_9NEOP (Q8HNY5) Cytochrome oxidase I (Fragment), partial (9%) [THC2685096] | 0,6961 | 6,77E-05 | 1,26E-03 |
| PRR15 | NM_175887 | Homo sapiens proline rich 15 (PRR15), mRNA [NM_175887] | 0,5262 | 6,79E-05 | 1,26E-03 |
| ATP1A1 | NM_000701 | Homo sapiens ATPase, Na+/K+ transporting, alpha 1 polypeptide (ATP1A1), transcript variant 1, mRNA [NM_000701] | 0,8175 | 6,83E-05 | 1,27E-03 |
| FXN | NM_181425 | Homo sapiens frataxin (FXN), nuclear gene encoding mitochondrial protein, transcript variant 2, mRNA [NM_181425] | 0,6669 | 6,85E-05 | 1,27E-03 |
| ZDHHC5 | NM_015457 | Homo sapiens zinc finger, DHHC-type containing 5 (ZDHHC5), mRNA [NM_015457] | 0,6941 | 6,88E-05 | 1,27E-03 |
| POP7 | NM_005837 | Homo sapiens processing of precursor 7, ribonuclease P subunit (S. cerevisiae) (POP7), mRNA [NM_005837] | 0,6539 | 6,88E-05 | 1,27E-03 |
| LRBA | NM_006726 | Homo sapiens LPS-responsive vesicle trafficking, beach and anchor containing (LRBA), mRNA [NM_006726] | 0,9079 | 6,91E-05 | 1,27E-03 |
| TMEM14B | NM_030969 | Homo sapiens transmembrane protein 14B (TMEM14B), mRNA [NM_030969] | 0,9781 | 6,93E-05 | 1,28E-03 |
| C9orf40 | NM_017998 | Homo sapiens chromosome 9 open reading frame 40 (C9orf40), mRNA [NM_017998] | 1,1461 | 7,01E-05 | 1,29E-03 |
| SDF2L1 | NM_022044 | Homo sapiens stromal cell-derived factor 2-like 1 (SDF2L1), mRNA [NM_022044] | 0,7365 | 7,01E-05 | 1,29E-03 |
| CR606637 | CR606637 | full-length cDNA clone CS0DI007YA21 of Placenta Cot 25-normalized of Homo sapiens (human). [CR606637] | 0,7281 | 7,11E-05 | 1,30E-03 |
| SUV39H1 | NM_003173 | Homo sapiens suppressor of variegation 3-9 homolog 1 (Drosophila) (SUV39H1), mRNA [NM_003173] | 0,6379 | 7,15E-05 | 1,31E-03 |
| TIMM44 | NM_006351 | Homo sapiens translocase of inner mitochondrial membrane 44 homolog (yeast) (TIMM44), mRNA [NM_006351] | 0,9710 | 7,28E-05 | 1,33E-03 |
| MST1 | NM_020998 | Homo sapiens macrophage stimulating 1 (hepatocyte growth factor-like) (MST1), mRNA [NM_020998] | 1,2277 | 7,37E-05 | 1,35E-03 |
| RGS16 | NM_002928 | Homo sapiens regulator of G-protein signalling 16 (RGS16), mRNA [NM_002928] | 1,2160 | 7,42E-05 | 1,35E-03 |
| TFR2 | NM_003227 | Homo sapiens transferrin receptor 2 (TFR2), mRNA [NM_003227] | 0,8489 | 7,43E-05 | 1,35E-03 |
| CGI-115 | NM_016052 | Homo sapiens CGI-115 protein (CGI-115), mRNA [NM_016052] | 0,5897 | 7,46E-05 | 1,36E-03 |
| ARL1 | NM_001177 | Homo sapiens ADP-ribosylation factor-like 1 (ARL1), mRNA [NM_001177] | 0,7176 | 7,48E-05 | 1,36E-03 |
| LOC391559 | XR_018345 | PREDICTED: Homo sapiens similar to vesicle transport through interaction with t-SNAREs 1B (LOC391559), mRNA [XR_018345] | 0,7356 | 7,63E-05 | 1,38E-03 |
| UROS | NM_000375 | Homo sapiens uroporphyrinogen III synthase (congenital erythropoietic porphyria) (UROS), mRNA [NM_000375] | 1,0950 | 7,66E-05 | 1,39E-03 |
| CDKN2C | NM_078626 | Homo sapiens cyclin-dependent kinase inhibitor 2C (p18, inhibits CDK4) (CDKN2C), transcript variant 2, mRNA [NM_078626] | 0,8629 | 7,78E-05 | 1,40E-03 |
| CXCL12 | NM_000609 | Homo sapiens chemokine (C-X-C motif) ligand 12 (stromal cell-derived factor 1) (CXCL12), transcript variant 2, mRNA [NM_000609] | 1,4631 | 7,87E-05 | 1,42E-03 |
| HTRA2 | NM_145074 | Homo sapiens HtrA serine peptidase 2 (HTRA2), nuclear gene encoding mitochondrial protein, transcript variant 2, mRNA [NM_145074] | 0,6888 | 7,88E-05 | 1,42E-03 |
| LIPA | NM_000235 | Homo sapiens lipase A, lysosomal acid, cholesterol esterase (Wolman disease) (LIPA), mRNA [NM_000235] | 0,9254 | 7,90E-05 | 1,42E-03 |
| AKR1C3 | NM_003739 | Homo sapiens aldo-keto reductase family 1, member C3 (3-alpha hydroxysteroid dehydrogenase, type II) (AKR1C3), mRNA [NM_003739] | 1,0985 | 7,94E-05 | 1,43E-03 |
| GAD1 | NM_013445 | Homo sapiens glutamate decarboxylase 1 (brain, 67kDa) (GAD1), transcript variant GAD25, mRNA [NM_013445] | 1,0617 | 7,96E-05 | 1,43E-03 |
| ENST00000379879 | ENST00000379879 | Immunglobulin heavy chain variable region (Fragment). [Source:Uniprot/SPTREMBL;Acc:Q0ZCG6] [ENST00000379879] | 1,1687 | 7,98E-05 | 1,43E-03 |
| MFAP1 | NM_005926 | Homo sapiens microfibrillar-associated protein 1 (MFAP1), mRNA [NM_005926] | 0,7084 | 8,02E-05 | 1,44E-03 |
| RBM13 | NM_032509 | Homo sapiens RNA binding motif protein 13 (RBM13), mRNA [NM_032509] | 0,6650 | 8,13E-05 | 1,45E-03 |
| AGPAT4 | NM_020133 | Homo sapiens 1-acylglycerol-3-phosphate O-acyltransferase 4 (lysophosphatidic acid acyltransferase, delta) (AGPAT4), mRNA [NM_020133] | 1,0671 | 8,29E-05 | 1,48E-03 |
| PLTP | NM_006227 | Homo sapiens phospholipid transfer protein (PLTP), transcript variant 1, mRNA [NM_006227] | 0,6938 | 8,32E-05 | 1,48E-03 |
| POLDIP2 | NM_015584 | Homo sapiens polymerase (DNA-directed), delta interacting protein 2 (POLDIP2), mRNA [NM_015584] | 0,7860 | 8,45E-05 | 1,50E-03 |
| TMEM85 | NM_016454 | Homo sapiens transmembrane protein 85 (TMEM85), mRNA [NM_016454] | 0,5757 | 8,46E-05 | 1,50E-03 |
| A_24_P814246 | A_24_P814246 | Unknown | 0,8288 | 8,54E-05 | 1,51E-03 |
| SHMT1 | NM_004169 | Homo sapiens serine hydroxymethyltransferase 1 (soluble) (SHMT1), transcript variant 1, mRNA [NM_004169] | 0,6504 | 8,54E-05 | 1,51E-03 |
| GPX1 | NM_201397 | Homo sapiens glutathione peroxidase 1 (GPX1), transcript variant 2, mRNA [NM_201397] | 0,7559 | 8,58E-05 | 1,51E-03 |
| SNX9 | NM_016224 | Homo sapiens sorting nexin 9 (SNX9), mRNA [NM_016224] | 0,9990 | 8,59E-05 | 1,52E-03 |
| MBP | NM_001025100 | Homo sapiens myelin basic protein (MBP), transcript variant 8, mRNA [NM_001025100] | 1,0312 | 8,62E-05 | 1,52E-03 |
| IFT122 | NM_018262 | Homo sapiens intraflagellar transport 122 homolog (Chlamydomonas) (IFT122), transcript variant 3, mRNA [NM_018262] | 0,6166 | 8,73E-05 | 1,53E-03 |
| MGC14327 | NM_053045 | Homo sapiens hypothetical protein MGC14327 (MGC14327), mRNA [NM_053045] | 0,8009 | 8,74E-05 | 1,53E-03 |
| TXNRD2 | NM_006440 | Homo sapiens thioredoxin reductase 2 (TXNRD2), nuclear gene encoding mitochondrial protein, mRNA [NM_006440] | 0,6401 | 8,80E-05 | 1,54E-03 |
| DOHH | NM_031304 | Homo sapiens deoxyhypusine hydroxylase/monooxygenase (DOHH), mRNA [NM_031304] | 0,9067 | 8,88E-05 | 1,55E-03 |
| AF086139 | AF086139 | Homo sapiens full length insert cDNA clone ZA91F08. [AF086139] | 0,9560 | 9,01E-05 | 1,57E-03 |
| TAC3 | NM_001006667 | Homo sapiens tachykinin 3 (neuromedin K, neurokinin beta) (TAC3), transcript variant 1, mRNA [NM_001006667] | 0,7403 | 9,16E-05 | 1,59E-03 |
| FAM129B | AF151783 | Homo sapiens MEG3 (MEG3) mRNA, complete cds. [AF151783] | 0,9839 | 9,17E-05 | 1,59E-03 |
| B4GALT7 | NM_007255 | Homo sapiens xylosylprotein beta 1,4-galactosyltransferase, polypeptide 7 (galactosyltransferase I) (B4GALT7), mRNA [NM_007255] | 0,6089 | 9,18E-05 | 1,59E-03 |
| SNX8 | NM_013321 | Homo sapiens sorting nexin 8 (SNX8), mRNA [NM_013321] | 0,9758 | 9,22E-05 | 1,60E-03 |
| A_24_P341408 | A_24_P341408 | Unknown | 0,7575 | 9,27E-05 | 1,60E-03 |
| MGC5139 | BC004815 | Homo sapiens hypothetical protein MGC5139, mRNA (cDNA clone IMAGE:3448346), complete cds. [BC004815] | 0,6983 | 9,39E-05 | 1,62E-03 |
| MAP2K3 | NM_145109 | Homo sapiens mitogen-activated protein kinase kinase 3 (MAP2K3), transcript variant B, mRNA [NM_145109] | 1,0641 | 9,46E-05 | 1,63E-03 |
| C1orf26 | NM_017673 | Homo sapiens chromosome 1 open reading frame 26 (C1orf26), mRNA [NM_017673] | 0,8178 | 9,58E-05 | 1,65E-03 |
| TUBB2A | NM_001069 | Homo sapiens tubulin, beta 2A (TUBB2A), mRNA [NM_001069] | 1,0592 | 9,65E-05 | 1,66E-03 |
| AK023159 | AK023159 | Homo sapiens cDNA FLJ13097 fis, clone NT2RP3002173. [AK023159] | 0,5230 | 9,76E-05 | 1,67E-03 |
| CTNNA1 | NM_001903 | Homo sapiens catenin (cadherin-associated protein), alpha 1, 102kDa (CTNNA1), mRNA [NM_001903] | 0,8284 | 9,84E-05 | 1,68E-03 |
| BC039021 | BC039021 | Homo sapiens cDNA clone IMAGE:6043059, partial cds. [BC039021] | 1,0312 | 9,98E-05 | 1,70E-03 |
| DCUN1D1 | AF292100 | Homo sapiens RP42 protein mRNA, complete cds. [AF292100] | 0,9007 | 1,02E-04 | 1,73E-03 |
| BG259864 | BG259864 | BG259864 602371819F1 NIH_MGC_93 Homo sapiens cDNA clone IMAGE:4479749 5', mRNA sequence [BG259864] | 1,0838 | 1,03E-04 | 1,74E-03 |
| MARVELD2 | NM_144724 | Homo sapiens MARVEL domain containing 2 (MARVELD2), transcript variant 2, mRNA [NM_144724] | 0,5669 | 1,03E-04 | 1,75E-03 |
| PCCB | NM_000532 | Homo sapiens propionyl Coenzyme A carboxylase, beta polypeptide (PCCB), mRNA [NM_000532] | 1,0505 | 1,03E-04 | 1,75E-03 |
| RPL4 | NM_000968 | Homo sapiens ribosomal protein L4 (RPL4), mRNA [NM_000968] | 0,6002 | 1,04E-04 | 1,75E-03 |
| AK123096 | AK123096 | Homo sapiens cDNA FLJ41101 fis, clone BLADE2004670. [AK123096] | 0,9570 | 1,04E-04 | 1,76E-03 |
| DEAF1 | NM_021008 | Homo sapiens deformed epidermal autoregulatory factor 1 (Drosophila) (DEAF1), mRNA [NM_021008] | 0,8533 | 1,04E-04 | 1,76E-03 |
| LMAN2 | NM_006816 | Homo sapiens lectin, mannose-binding 2 (LMAN2), mRNA [NM_006816] | 0,7631 | 1,05E-04 | 1,76E-03 |
| GRWD1 | NM_031485 | Homo sapiens glutamate-rich WD repeat containing 1 (GRWD1), mRNA [NM_031485] | 0,7132 | 1,06E-04 | 1,78E-03 |
| AGPAT3 | NM_020132 | Homo sapiens 1-acylglycerol-3-phosphate O-acyltransferase 3 (AGPAT3), transcript variant 1, mRNA [NM_020132] | 0,6934 | 1,07E-04 | 1,79E-03 |
| MKRN1 | AF117233 | Homo sapiens znf-xp protein mRNA, complete cds. [AF117233] | 1,1180 | 1,08E-04 | 1,80E-03 |
| C19orf48 | NM_199249 | Homo sapiens chromosome 19 open reading frame 48 (C19orf48), mRNA [NM_199249] | 0,6225 | 1,08E-04 | 1,80E-03 |
| CYB5A | NM_001914 | Homo sapiens cytochrome b5 type A (microsomal) (CYB5A), transcript variant 2, mRNA [NM_001914] | 0,8784 | 1,09E-04 | 1,82E-03 |
| BX105952 | BX105952 | BX105952 Soares placenta Nb2HP Homo sapiens cDNA clone IMAGp998D01212, mRNA sequence [BX105952] | 0,7131 | 1,10E-04 | 1,84E-03 |
| C10orf61 | NM_015631 | Homo sapiens chromosome 10 open reading frame 61 (C10orf61), transcript variant 2, mRNA [NM_015631] | 0,9639 | 1,10E-04 | 1,84E-03 |
| YIPF6 | NM_173834 | Homo sapiens Yip1 domain family, member 6 (YIPF6), mRNA [NM_173834] | 0,8862 | 1,10E-04 | 1,84E-03 |
| ARL2 | NM_001667 | Homo sapiens ADP-ribosylation factor-like 2 (ARL2), mRNA [NM_001667] | 0,6817 | 1,11E-04 | 1,85E-03 |
| LOC731076 | XR_015691 | PREDICTED: Homo sapiens hypothetical protein LOC731076 (LOC731076), mRNA [XR_015691] | 0,7838 | 1,12E-04 | 1,85E-03 |
| CYBRD1 | NM_024843 | Homo sapiens cytochrome b reductase 1 (CYBRD1), mRNA [NM_024843] | 0,8588 | 1,12E-04 | 1,86E-03 |
| AJ519285 | AJ519285 | Homo sapiens partial mRNA for IgM immunoglobulin heavy chain variable region (IGHV gene), clone ANBPM204. [AJ519285] | 1,1703 | 1,12E-04 | 1,86E-03 |
| FKBP1B | NM_054033 | Homo sapiens FK506 binding protein 1B, 12.6 kDa (FKBP1B), transcript variant 2, mRNA [NM_054033] | 0,9276 | 1,12E-04 | 1,86E-03 |
| DYRK3 | NM_001004023 | Homo sapiens dual-specificity tyrosine-(Y)-phosphorylation regulated kinase 3 (DYRK3), transcript variant 2, mRNA [NM_001004023] | 0,8069 | 1,13E-04 | 1,86E-03 |
| PPP1R8 | NM_138558 | Homo sapiens protein phosphatase 1, regulatory (inhibitor) subunit 8 (PPP1R8), transcript variant 2, mRNA [NM_138558] | 0,8405 | 1,13E-04 | 1,86E-03 |
| MBOAT5 | NM_005768 | Homo sapiens membrane bound O-acyltransferase domain containing 5 (MBOAT5), mRNA [NM_005768] | 0,9682 | 1,14E-04 | 1,89E-03 |
| PRPF8 | NM_006445 | Homo sapiens PRP8 pre-mRNA processing factor 8 homolog (S. cerevisiae) (PRPF8), mRNA [NM_006445] | 0,6677 | 1,14E-04 | 1,89E-03 |
| RMND5A | NM_022780 | Homo sapiens required for meiotic nuclear division 5 homolog A (S. cerevisiae) (RMND5A), mRNA [NM_022780] | 0,8077 | 1,17E-04 | 1,93E-03 |
| ENST00000380344 | ENST00000380344 | Beta-1,3-glucosyltransferase (EC 2.4.1.-) (Beta3Glc-T) (Beta-3- glycosyltransferase-like). [Source:Uniprot/SWISSPROT;Acc:Q6Y288] [ENST00000380344] | 0,7220 | 1,17E-04 | 1,93E-03 |
| GPR132 | NM_013345 | Homo sapiens G protein-coupled receptor 132 (GPR132), mRNA [NM_013345] | 1,2362 | 1,18E-04 | 1,94E-03 |
| ARPC1A | NM_006409 | Homo sapiens actin related protein 2/3 complex, subunit 1A, 41kDa (ARPC1A), mRNA [NM_006409] | 0,6706 | 1,18E-04 | 1,94E-03 |
| C20orf141 | BC021178 | Homo sapiens chromosome 20 open reading frame 141, mRNA (cDNA clone MGC:33216 IMAGE:5265299), complete cds. [BC021178] | 0,8503 | 1,19E-04 | 1,95E-03 |
| BC087732 | BC087732 | Homo sapiens cDNA clone IMAGE:6253289, **** WARNING: chimeric clone ****. [BC087732] | 0,5869 | 1,21E-04 | 1,99E-03 |
| RASIP1 | NM_017805 | Homo sapiens Ras interacting protein 1 (RASIP1), mRNA [NM_017805] | 0,9754 | 1,22E-04 | 1,99E-03 |
| ACOT2 | NM_006821 | Homo sapiens acyl-CoA thioesterase 2 (ACOT2), mRNA [NM_006821] | 0,7600 | 1,22E-04 | 1,99E-03 |
| ANKRD25 | NM_015493 | Homo sapiens ankyrin repeat domain 25 (ANKRD25), mRNA [NM_015493] | 0,9269 | 1,24E-04 | 2,02E-03 |
| TOP1P2 | NR_001283 | Homo sapiens topoisomerase (DNA) I pseudogene 2 (TOP1P2) on chromosome 22 [NR_001283] | 0,8577 | 1,26E-04 | 2,05E-03 |
| SLAMF7 | NM_021181 | Homo sapiens SLAM family member 7 (SLAMF7), mRNA [NM_021181] | 1,2991 | 1,27E-04 | 2,06E-03 |
| MCM6 | NM_005915 | Homo sapiens minichromosome maintenance deficient 6 homolog (S. cerevisiae) (MCM6), mRNA [NM_005915] | 0,6331 | 1,27E-04 | 2,06E-03 |
| KEAP1 | NM_203500 | Homo sapiens kelch-like ECH-associated protein 1 (KEAP1), transcript variant 1, mRNA [NM_203500] | 0,7632 | 1,28E-04 | 2,08E-03 |
| CRAT | NM_000755 | Homo sapiens carnitine acetyltransferase (CRAT), transcript variant 1, mRNA [NM_000755] | 0,6416 | 1,30E-04 | 2,09E-03 |
| ATG4A | NM_178271 | Homo sapiens ATG4 autophagy related 4 homolog A (S. cerevisiae) (ATG4A), transcript variant 3, mRNA [NM_178271] | 0,9298 | 1,30E-04 | 2,09E-03 |
| RIC8A | NM_021932 | Homo sapiens resistance to inhibitors of cholinesterase 8 homolog A (C. elegans) (RIC8A), mRNA [NM_021932] | 0,7537 | 1,30E-04 | 2,10E-03 |
| CCDC47 | NM_020198 | Homo sapiens coiled-coil domain containing 47 (CCDC47), mRNA [NM_020198] | 0,9221 | 1,31E-04 | 2,11E-03 |
| ALS2 | AK023024 | Homo sapiens cDNA FLJ12962 fis, clone NT2RP2005694, weakly similar to X-LINKED RETINITIS PIGMENTOSA GTPASE REGULATOR. [AK023024] | 0,5572 | 1,32E-04 | 2,12E-03 |
| OXNAD1 | NM_138381 | Homo sapiens oxidoreductase NAD-binding domain containing 1 (OXNAD1), mRNA [NM_138381] | 0,6475 | 1,32E-04 | 2,12E-03 |
| GSTT1 | NM_000853 | Homo sapiens glutathione S-transferase theta 1 (GSTT1), mRNA [NM_000853] | 0,9286 | 1,33E-04 | 2,13E-03 |
| CYBASC3 | NM_153611 | Homo sapiens cytochrome b, ascorbate dependent 3 (CYBASC3), mRNA [NM_153611] | 0,8193 | 1,33E-04 | 2,13E-03 |
| PTPLAD1 | NM_016395 | Homo sapiens protein tyrosine phosphatase-like A domain containing 1 (PTPLAD1), mRNA [NM_016395] | 0,7835 | 1,35E-04 | 2,17E-03 |
| C14orf1 | NM_007176 | Homo sapiens chromosome 14 open reading frame 1 (C14orf1), mRNA [NM_007176] | 0,6869 | 1,36E-04 | 2,18E-03 |
| SEC24C | NM_004922 | Homo sapiens SEC24 related gene family, member C (S. cerevisiae) (SEC24C), transcript variant 1, mRNA [NM_004922] | 0,6789 | 1,37E-04 | 2,19E-03 |
| A_32_P99804 | A_32_P99804 | Unknown | 0,7422 | 1,38E-04 | 2,20E-03 |
| AL117621 | AL117621 | Homo sapiens mRNA; cDNA DKFZp564M0264 (from clone DKFZp564M0264). [AL117621] | 0,7392 | 1,38E-04 | 2,20E-03 |
| RAC1 | NM_198829 | Homo sapiens ras-related C3 botulinum toxin substrate 1 (rho family, small GTP binding protein Rac1) (RAC1), transcript variant Rac1c, mRNA [NM_198829] | 0,8187 | 1,38E-04 | 2,20E-03 |
| RPA1 | NM_002945 | Homo sapiens replication protein A1, 70kDa (RPA1), mRNA [NM_002945] | 0,8201 | 1,38E-04 | 2,20E-03 |
| PDCD1 | NM_005018 | Homo sapiens programmed cell death 1 (PDCD1), mRNA [NM_005018] | 1,2652 | 1,41E-04 | 2,23E-03 |
| TMEM58 | NM_198149 | Homo sapiens transmembrane protein 58 (TMEM58), mRNA [NM_198149] | 1,1725 | 1,41E-04 | 2,23E-03 |
| SYTL4 | NM_080737 | Homo sapiens synaptotagmin-like 4 (granuphilin-a) (SYTL4), mRNA [NM_080737] | 0,6275 | 1,41E-04 | 2,24E-03 |
| TMEM48 | AK091439 | Homo sapiens cDNA FLJ34120 fis, clone FCBBF3009541. [AK091439] | 0,5603 | 1,42E-04 | 2,24E-03 |
| THC2649313 | THC2649313 | Unknown | 0,6639 | 1,42E-04 | 2,24E-03 |
| LOC343508 | XR_017446 | PREDICTED: Homo sapiens similar to aconitase 2 precursor (LOC343508), mRNA [XR_017446] | 0,7097 | 1,42E-04 | 2,25E-03 |
| THC2685373 | THC2685373 | Unknown | 0,7230 | 1,44E-04 | 2,27E-03 |
| TIMM23 | AF030162 | Homo sapiens inner mitochondrial membrane translocase Tim23 (TIM23) mRNA, nuclear gene encoding mitochondrial protein, complete cds. [AF030162] | 0,9875 | 1,44E-04 | 2,27E-03 |
| LEPROTL1 | NM_015344 | Homo sapiens leptin receptor overlapping transcript-like 1 (LEPROTL1), mRNA [NM_015344] | 0,9107 | 1,45E-04 | 2,27E-03 |
| AK024898 | AK024898 | Homo sapiens cDNA: FLJ21245 fis, clone COL01184. [AK024898] | 0,7020 | 1,46E-04 | 2,29E-03 |
| TUBB1 | NM_030773 | Homo sapiens tubulin, beta 1 (TUBB1), mRNA [NM_030773] | 0,6493 | 1,47E-04 | 2,31E-03 |
| SIAHBP1 | NM_014281 | Homo sapiens fuse-binding protein-interacting repressor (SIAHBP1), transcript variant 2, mRNA [NM_014281] | 0,5850 | 1,49E-04 | 2,32E-03 |
| C14orf169 | NM_024644 | Homo sapiens chromosome 14 open reading frame 169 (C14orf169), mRNA [NM_024644] | 0,9508 | 1,52E-04 | 2,37E-03 |
| DDHD2 | NM_015214 | Homo sapiens DDHD domain containing 2 (DDHD2), mRNA [NM_015214] | 0,4969 | 1,53E-04 | 2,38E-03 |
| A_24_P194954 | A_24_P194954 | Unknown | 0,6036 | 1,53E-04 | 2,38E-03 |
| OAT | NM_000274 | Homo sapiens ornithine aminotransferase (gyrate atrophy) (OAT), nuclear gene encoding mitochondrial protein, mRNA [NM_000274] | 0,8868 | 1,54E-04 | 2,39E-03 |
| MGC29891 | ENST00000368918 | GA repeat binding protein, beta 2 [Source:RefSeq_peptide;Acc:NP_653219] [ENST00000368918] | 0,5473 | 1,54E-04 | 2,40E-03 |
| AK127768 | AK127768 | Homo sapiens cDNA FLJ45869 fis, clone OCBBF3004908. [AK127768] | 2,2146 | 1,54E-04 | 2,40E-03 |
| MXD4 | NM_006454 | Homo sapiens MAX dimerization protein 4 (MXD4), mRNA [NM_006454] | 0,7638 | 1,56E-04 | 2,42E-03 |
| C11orf77 | NM_173811 | Homo sapiens chromosome 11 open reading frame 77 (C11orf77), mRNA [NM_173811] | 0,7033 | 1,57E-04 | 2,43E-03 |
| C9orf5 | NM_032012 | Homo sapiens chromosome 9 open reading frame 5 (C9orf5), mRNA [NM_032012] | 0,4731 | 1,57E-04 | 2,43E-03 |
| SPHK1 | NM_021972 | Homo sapiens sphingosine kinase 1 (SPHK1), transcript variant 1, mRNA [NM_021972] | 1,1434 | 1,57E-04 | 2,43E-03 |
| IQWD1 | NM_018442 | Homo sapiens IQ motif and WD repeats 1 (IQWD1), transcript variant 1, mRNA [NM_018442] | 0,8608 | 1,59E-04 | 2,46E-03 |
| CDC42BPA | NM_003607 | Homo sapiens CDC42 binding protein kinase alpha (DMPK-like) (CDC42BPA), transcript variant B, mRNA [NM_003607] | 0,6133 | 1,63E-04 | 2,51E-03 |
| ITSN1 | NM_001001132 | Homo sapiens intersectin 1 (SH3 domain protein) (ITSN1), transcript variant 2, mRNA [NM_001001132] | 0,8685 | 1,65E-04 | 2,54E-03 |
| KCTD5 | NM_018992 | Homo sapiens potassium channel tetramerisation domain containing 5 (KCTD5), mRNA [NM_018992] | 0,8155 | 1,66E-04 | 2,55E-03 |
| IL32 | NM_001012631 | Homo sapiens interleukin 32 (IL32), transcript variant 1, mRNA [NM_001012631] | 1,4346 | 1,68E-04 | 2,56E-03 |
| TFDP1 | NM_007111 | Homo sapiens transcription factor Dp-1 (TFDP1), mRNA [NM_007111] | 0,7393 | 1,68E-04 | 2,56E-03 |
| SAMD1 | NM_138352 | Homo sapiens sterile alpha motif domain containing 1 (SAMD1), mRNA [NM_138352] | 0,4890 | 1,69E-04 | 2,57E-03 |
| PDK2 | NM_002611 | Homo sapiens pyruvate dehydrogenase kinase, isozyme 2 (PDK2), mRNA [NM_002611] | 0,7587 | 1,69E-04 | 2,58E-03 |
| APLN | NM_017413 | Homo sapiens apelin, AGTRL1 ligand (APLN), mRNA [NM_017413] | 0,7483 | 1,69E-04 | 2,58E-03 |
| SHCBP1 | NM_024745 | Homo sapiens SHC SH2-domain binding protein 1 (SHCBP1), mRNA [NM_024745] | 0,9562 | 1,70E-04 | 2,58E-03 |
| CSDA | NM_003651 | Homo sapiens cold shock domain protein A (CSDA), mRNA [NM_003651] | 1,0737 | 1,70E-04 | 2,58E-03 |
| WDR32 | NM_024345 | Homo sapiens WD repeat domain 32 (WDR32), mRNA [NM_024345] | 2,5712 | 1,70E-04 | 2,58E-03 |
| AFG3L2 | NM_006796 | Homo sapiens AFG3 ATPase family gene 3-like 2 (yeast) (AFG3L2), nuclear gene encoding mitochondrial protein, mRNA [NM_006796] | 0,6734 | 1,73E-04 | 2,62E-03 |
| DKFZP686E2158 | NM_001048249 | Homo sapiens hypothetical protein LOC643155 (DKFZP686E2158), mRNA [NM_001048249] | 0,6416 | 1,74E-04 | 2,64E-03 |
| TMEM9B | NM_020644 | Homo sapiens TMEM9 domain family, member B (TMEM9B), mRNA [NM_020644] | 0,8337 | 1,76E-04 | 2,66E-03 |
| BC018095 | BC018095 | Homo sapiens cDNA clone IMAGE:4793786. [BC018095] | 1,4764 | 1,77E-04 | 2,67E-03 |
| KIAA1450 | AB040883 | Homo sapiens mRNA for KIAA1450 protein, partial cds. [AB040883] | 0,6871 | 1,77E-04 | 2,67E-03 |
| A_23_P28743 | A_23_P28743 | Unknown | 0,8769 | 1,78E-04 | 2,67E-03 |
| RPP38 | NM_183005 | Homo sapiens ribonuclease P/MRP 38kDa subunit (RPP38), transcript variant 1, mRNA [NM_183005] | 0,7633 | 1,79E-04 | 2,69E-03 |
| DGCR6 | NM_005675 | Homo sapiens DiGeorge syndrome critical region gene 6 (DGCR6), mRNA [NM_005675] | 0,5660 | 1,79E-04 | 2,69E-03 |
| DNASE2 | NM_001375 | Homo sapiens deoxyribonuclease II, lysosomal (DNASE2), mRNA [NM_001375] | 0,7562 | 1,80E-04 | 2,70E-03 |
| RBM12 | NM_006047 | Homo sapiens RNA binding motif protein 12 (RBM12), transcript variant 1, mRNA [NM_006047] | 0,8499 | 1,80E-04 | 2,70E-03 |
| CALM3 | NM_005184 | Homo sapiens calmodulin 3 (phosphorylase kinase, delta) (CALM3), mRNA [NM_005184] | 0,6051 | 1,81E-04 | 2,72E-03 |
| C22orf9 | NM_015264 | Homo sapiens chromosome 22 open reading frame 9 (C22orf9), transcript variant 1, mRNA [NM_015264] | 0,8690 | 1,82E-04 | 2,72E-03 |
| NGRN | NM_001033088 | Homo sapiens neugrin, neurite outgrowth associated (NGRN), transcript variant 2, mRNA [NM_001033088] | 0,9624 | 1,84E-04 | 2,76E-03 |
| LGR4 | NM_018490 | Homo sapiens leucine-rich repeat-containing G protein-coupled receptor 4 (LGR4), mRNA [NM_018490] | 0,7175 | 1,85E-04 | 2,76E-03 |
| KIF11 | NM_004523 | Homo sapiens kinesin family member 11 (KIF11), mRNA [NM_004523] | 0,7150 | 1,86E-04 | 2,77E-03 |
| HCCS | NM_005333 | Homo sapiens holocytochrome c synthase (cytochrome c heme-lyase) (HCCS), mRNA [NM_005333] | 0,7409 | 1,88E-04 | 2,79E-03 |
| RGC32 | NM_014059 | Homo sapiens response gene to complement 32 (RGC32), mRNA [NM_014059] | 1,0939 | 1,88E-04 | 2,80E-03 |
| ENST00000326261 | ENST00000326261 | IQ motif and Sec7 domain-containing protein 3. [Source:Uniprot/SWISSPROT;Acc:Q9UPP2] [ENST00000326261] | 0,5700 | 1,91E-04 | 2,82E-03 |
| ACAD9 | NM_014049 | Homo sapiens acyl-Coenzyme A dehydrogenase family, member 9 (ACAD9), mRNA [NM_014049] | 0,5087 | 1,91E-04 | 2,82E-03 |
| QARS | NM_005051 | Homo sapiens glutaminyl-tRNA synthetase (QARS), mRNA [NM_005051] | 0,7481 | 1,94E-04 | 2,86E-03 |
| ZBTB38 | BC072415 | Homo sapiens cDNA clone IMAGE:6168734. [BC072415] | 0,7247 | 1,94E-04 | 2,86E-03 |
| HSPBP1 | NM_012267 | Homo sapiens hsp70-interacting protein (HSPBP1), mRNA [NM_012267] | 0,7347 | 1,95E-04 | 2,87E-03 |
| RCCD1 | NM_033544 | Homo sapiens RCC1 domain containing 1 (RCCD1), transcript variant 1, mRNA [NM_033544] | 0,6900 | 1,95E-04 | 2,87E-03 |
| AA554330 | AA554330 | nl03d08.s1 NCI_CGAP_Co3 Homo sapiens cDNA clone IMAGE:1029231 3', mRNA sequence [AA554330] | 0,8152 | 1,96E-04 | 2,88E-03 |
| ATP1B1 | NM_001677 | Homo sapiens ATPase, Na+/K+ transporting, beta 1 polypeptide (ATP1B1), transcript variant 1, mRNA [NM_001677] | 0,8760 | 1,96E-04 | 2,88E-03 |
| PAIP1 | NM_006451 | Homo sapiens poly(A) binding protein interacting protein 1 (PAIP1), transcript variant 1, mRNA [NM_006451] | 0,8503 | 1,96E-04 | 2,88E-03 |
| TRAF3IP1 | BC059174 | Homo sapiens TNF receptor-associated factor 3 interacting protein 1, mRNA (cDNA clone MGC:54069 IMAGE:6191726), complete cds. [BC059174] | 0,6951 | 1,97E-04 | 2,89E-03 |
| KCNJ12 | NM_021012 | Homo sapiens potassium inwardly-rectifying channel, subfamily J, member 12 (KCNJ12), mRNA [NM_021012] | 0,8089 | 1,98E-04 | 2,89E-03 |
| CCDC124 | NM_138442 | Homo sapiens coiled-coil domain containing 124 (CCDC124), mRNA [NM_138442] | 0,4867 | 1,98E-04 | 2,90E-03 |
| LOC643992 | XR_018270 | PREDICTED: Homo sapiens hypothetical LOC643992 (LOC643992), mRNA [XR_018270] | 0,7306 | 1,99E-04 | 2,91E-03 |
| C16orf68 | NM_024109 | Homo sapiens chromosome 16 open reading frame 68 (C16orf68), mRNA [NM_024109] | 0,5157 | 2,02E-04 | 2,93E-03 |
| SF3B3 | D13642 | Homo sapiens KIAA0017 mRNA, complete cds. [D13642] | 0,4677 | 2,02E-04 | 2,93E-03 |
| HMG2L1 | NM_005487 | Homo sapiens high-mobility group protein 2-like 1 (HMG2L1), transcript variant 1, mRNA [NM_005487] | 0,7257 | 2,03E-04 | 2,95E-03 |
| GLG1 | NM_012201 | Homo sapiens golgi apparatus protein 1 (GLG1), mRNA [NM_012201] | 0,6235 | 2,03E-04 | 2,95E-03 |
| BRD3 | NM_007371 | Homo sapiens bromodomain containing 3 (BRD3), mRNA [NM_007371] | 1,1918 | 2,04E-04 | 2,96E-03 |
| PSMD12 | ENST00000356126 | 26S proteasome non-ATPase regulatory subunit 12 (26S proteasome regulatory subunit p55). [Source:Uniprot/SWISSPROT;Acc:O00232] [ENST00000356126] | 0,5418 | 2,05E-04 | 2,98E-03 |
| PRMT6 | NM_018137 | Homo sapiens protein arginine methyltransferase 6 (PRMT6), mRNA [NM_018137] | 1,0009 | 2,06E-04 | 2,98E-03 |
| TM7SF3 | NM_016551 | Homo sapiens transmembrane 7 superfamily member 3 (TM7SF3), mRNA [NM_016551] | 0,8845 | 2,06E-04 | 2,98E-03 |
| ZHX1 | NM_001017926 | Homo sapiens zinc fingers and homeoboxes 1 (ZHX1), transcript variant 1, mRNA [NM_001017926] | 0,7899 | 2,06E-04 | 2,98E-03 |
| PPAP2B | NM_003713 | Homo sapiens phosphatidic acid phosphatase type 2B (PPAP2B), transcript variant 1, mRNA [NM_003713] | 0,8606 | 2,08E-04 | 3,01E-03 |
| ALG3 | NM_005787 | Homo sapiens asparagine-linked glycosylation 3 homolog (S. cerevisiae, alpha-1,3-mannosyltransferase) (ALG3), mRNA [NM_005787] | 0,5885 | 2,10E-04 | 3,02E-03 |
| WBSCR18 | NM_032317 | Homo sapiens Williams Beuren syndrome chromosome region 18 (WBSCR18), mRNA [NM_032317] | 0,6803 | 2,10E-04 | 3,02E-03 |
| ZSCAN21 | NM_145914 | Homo sapiens zinc finger and SCAN domain containing 21 (ZSCAN21), mRNA [NM_145914] | 0,4952 | 2,10E-04 | 3,02E-03 |
| LRRC20 | NM_018205 | Homo sapiens leucine rich repeat containing 20 (LRRC20), transcript variant 3, mRNA [NM_018205] | 0,8987 | 2,11E-04 | 3,03E-03 |
| CD99 | NM_002414 | Homo sapiens CD99 molecule (CD99), mRNA [NM_002414] | 0,7871 | 2,11E-04 | 3,03E-03 |
| SH3GLB2 | NM_020145 | Homo sapiens SH3-domain GRB2-like endophilin B2 (SH3GLB2), mRNA [NM_020145] | 0,5920 | 2,11E-04 | 3,04E-03 |
| C20orf29 | NM_018347 | Homo sapiens chromosome 20 open reading frame 29 (C20orf29), mRNA [NM_018347] | 0,5026 | 2,13E-04 | 3,05E-03 |
| ZCD2 | NM_001008388 | Homo sapiens zinc finger, CDGSH-type domain 2 (ZCD2), mRNA [NM_001008388] | 1,2777 | 2,13E-04 | 3,05E-03 |
| APRIN | NM_015032 | Homo sapiens androgen-induced proliferation inhibitor (APRIN), mRNA [NM_015032] | 0,6772 | 2,13E-04 | 3,06E-03 |
| POLR3C | NM_006468 | Homo sapiens polymerase (RNA) III (DNA directed) polypeptide C (62kD) (POLR3C), mRNA [NM_006468] | 0,5637 | 2,18E-04 | 3,11E-03 |
| PDCL | NM_005388 | Homo sapiens phosducin-like (PDCL), mRNA [NM_005388] | 0,6749 | 2,18E-04 | 3,11E-03 |
| WDR13 | NM_017883 | Homo sapiens WD repeat domain 13 (WDR13), mRNA [NM_017883] | 0,5530 | 2,18E-04 | 3,11E-03 |
| ENST00000252134 | ENST00000252134 | Uncharacterized protein KIAA0819. [Source:Uniprot/SWISSPROT;Acc:O94909] [ENST00000252134] | 0,9727 | 2,18E-04 | 3,11E-03 |
| FGFRL1 | NM_001004356 | Homo sapiens fibroblast growth factor receptor-like 1 (FGFRL1), transcript variant 1, mRNA [NM_001004356] | 0,6086 | 2,18E-04 | 3,11E-03 |
| INTS5 | NM_030628 | Homo sapiens integrator complex subunit 5 (INTS5), mRNA [NM_030628] | 0,6221 | 2,19E-04 | 3,12E-03 |
| AAAS | NM_015665 | Homo sapiens achalasia, adrenocortical insufficiency, alacrimia (Allgrove, triple-A) (AAAS), mRNA [NM_015665] | 0,6314 | 2,20E-04 | 3,13E-03 |
| DECR2 | NM_020664 | Homo sapiens 2,4-dienoyl CoA reductase 2, peroxisomal (DECR2), mRNA [NM_020664] | 0,8772 | 2,20E-04 | 3,13E-03 |
| MOSPD1 | NM_019556 | Homo sapiens motile sperm domain containing 1 (MOSPD1), mRNA [NM_019556] | 0,8593 | 2,21E-04 | 3,13E-03 |
| UBE2V1 | NM_001032288 | Homo sapiens ubiquitin-conjugating enzyme E2 variant 1 (UBE2V1), transcript variant 4, mRNA [NM_001032288] | 0,7219 | 2,22E-04 | 3,14E-03 |
| TSEN54 | NM_207346 | Homo sapiens tRNA splicing endonuclease 54 homolog (S. cerevisiae) (TSEN54), mRNA [NM_207346] | 0,7747 | 2,22E-04 | 3,14E-03 |
| AP2S1 | NM_004069 | Homo sapiens adaptor-related protein complex 2, sigma 1 subunit (AP2S1), transcript variant AP17, mRNA [NM_004069] | 0,7749 | 2,22E-04 | 3,14E-03 |
| ST7 | NM_018412 | Homo sapiens suppression of tumorigenicity 7 (ST7), transcript variant a, mRNA [NM_018412] | 0,4710 | 2,23E-04 | 3,14E-03 |
| SIVA1 | NM_006427 | Homo sapiens SIVA1, apoptosis-inducing factor (SIVA1), transcript variant 1, mRNA [NM_006427] | 0,5281 | 2,23E-04 | 3,15E-03 |
| ZNF551 | NM_138347 | Homo sapiens zinc finger protein 551 (ZNF551), mRNA [NM_138347] | 0,5002 | 2,24E-04 | 3,15E-03 |
| TSPYL1 | NM_003309 | Homo sapiens TSPY-like 1 (TSPYL1), mRNA [NM_003309] | 1,0253 | 2,25E-04 | 3,16E-03 |
| C1orf164 | NM_018150 | Homo sapiens chromosome 1 open reading frame 164 (C1orf164), mRNA [NM_018150] | 0,5313 | 2,25E-04 | 3,17E-03 |
| ELAC2 | NM_018127 | Homo sapiens elaC homolog 2 (E. coli) (ELAC2), mRNA [NM_018127] | 0,7431 | 2,25E-04 | 3,17E-03 |
| RANGAP1 | NM_002883 | Homo sapiens Ran GTPase activating protein 1 (RANGAP1), mRNA [NM_002883] | 0,6528 | 2,27E-04 | 3,18E-03 |
| A_24_P552987 | A_24_P552987 | Unknown | 0,7341 | 2,29E-04 | 3,21E-03 |
| GOLGA4 | NM_002078 | Homo sapiens golgi autoantigen, golgin subfamily a, 4 (GOLGA4), mRNA [NM_002078] | 0,7622 | 2,30E-04 | 3,22E-03 |
| AP2M1 | NM_004068 | Homo sapiens adaptor-related protein complex 2, mu 1 subunit (AP2M1), transcript variant 1, mRNA [NM_004068] | 1,0804 | 2,31E-04 | 3,22E-03 |
| HDLBP | NM_005336 | Homo sapiens high density lipoprotein binding protein (vigilin) (HDLBP), mRNA [NM_005336] | 0,7303 | 2,31E-04 | 3,23E-03 |
| STOML2 | NM_013442 | Homo sapiens stomatin (EPB72)-like 2 (STOML2), mRNA [NM_013442] | 0,7175 | 2,33E-04 | 3,25E-03 |
| FUNDC2 | NM_023934 | Homo sapiens FUN14 domain containing 2 (FUNDC2), mRNA [NM_023934] | 0,9355 | 2,36E-04 | 3,28E-03 |
| VCP | NM_007126 | Homo sapiens valosin-containing protein (VCP), mRNA [NM_007126] | 0,4988 | 2,40E-04 | 3,33E-03 |
| KIAA0323 | NM_015299 | Homo sapiens KIAA0323 (KIAA0323), mRNA [NM_015299] | 0,9479 | 2,44E-04 | 3,39E-03 |
| C1orf77 | BC108721 | Homo sapiens chromosome 1 open reading frame 77, mRNA (cDNA clone MGC:131924 IMAGE:4450075), complete cds. [BC108721] | 0,5719 | 2,45E-04 | 3,39E-03 |
| ACADS | NM_000017 | Homo sapiens acyl-Coenzyme A dehydrogenase, C-2 to C-3 short chain (ACADS), nuclear gene encoding mitochondrial protein, mRNA [NM_000017] | 0,5736 | 2,46E-04 | 3,40E-03 |
| THC2636523 | THC2636523 | Unknown | 0,5463 | 2,51E-04 | 3,46E-03 |
| CLCN3 | NM_173872 | Homo sapiens chloride channel 3 (CLCN3), transcript variant e, mRNA [NM_173872] | 0,7207 | 2,52E-04 | 3,46E-03 |
| FLJ14981 | NM_032868 | Homo sapiens hypothetical protein FLJ14981 (FLJ14981), mRNA [NM_032868] | 0,9317 | 2,52E-04 | 3,46E-03 |
| ZNF696 | NM_030895 | Homo sapiens zinc finger protein 696 (ZNF696), mRNA [NM_030895] | 0,6158 | 2,57E-04 | 3,53E-03 |
| KIAA0355 | NM_014686 | Homo sapiens KIAA0355 (KIAA0355), mRNA [NM_014686] | 0,8762 | 2,58E-04 | 3,53E-03 |
| PRRT3 | NM_207351 | Homo sapiens proline-rich transmembrane protein 3 (PRRT3), mRNA [NM_207351] | 0,6109 | 2,59E-04 | 3,55E-03 |
| THC2672257 | THC2672257 | Unknown | 0,7089 | 2,60E-04 | 3,55E-03 |
| LANCL2 | NM_018697 | Homo sapiens LanC lantibiotic synthetase component C-like 2 (bacterial) (LANCL2), mRNA [NM_018697] | 0,8314 | 2,62E-04 | 3,57E-03 |
| WRN | NM_000553 | Homo sapiens Werner syndrome (WRN), mRNA [NM_000553] | 0,8127 | 2,62E-04 | 3,57E-03 |
| L3MBTL2 | NM_001003689 | Homo sapiens l(3)mbt-like 2 (Drosophila) (L3MBTL2), transcript variant 2, mRNA [NM_001003689] | 0,5908 | 2,65E-04 | 3,60E-03 |
| SMC1A | NM_006306 | Homo sapiens structural maintenance of chromosomes 1A (SMC1A), mRNA [NM_006306] | 0,9317 | 2,65E-04 | 3,60E-03 |
| LOC200810 | NM_001015050 | Homo sapiens similar to beta-1,4-mannosyltransferase; beta-1,4 mannosyltransferase (LOC200810), mRNA [NM_001015050] | 0,6784 | 2,67E-04 | 3,62E-03 |
| XPO4 | NM_022459 | Homo sapiens exportin 4 (XPO4), mRNA [NM_022459] | 0,5686 | 2,69E-04 | 3,64E-03 |
| KIAA1191 | NM_020444 | Homo sapiens KIAA1191 (KIAA1191), transcript variant 1, mRNA [NM_020444] | 0,8322 | 2,70E-04 | 3,67E-03 |
| THC2506002 | THC2506002 | Unknown | 0,6854 | 2,73E-04 | 3,70E-03 |
| CYB5R3 | NM_007326 | Homo sapiens cytochrome b5 reductase 3 (CYB5R3), transcript variant S, mRNA [NM_007326] | 0,5044 | 2,75E-04 | 3,72E-03 |
| ENO2 | NM_001975 | Homo sapiens enolase 2 (gamma, neuronal) (ENO2), mRNA [NM_001975] | 0,6176 | 2,76E-04 | 3,72E-03 |
| NYX | NM_022567 | Homo sapiens nyctalopin (NYX), mRNA [NM_022567] | 1,5966 | 2,76E-04 | 3,72E-03 |
| CIRH1A | NM_032830 | Homo sapiens cirrhosis, autosomal recessive 1A (cirhin) (CIRH1A), mRNA [NM_032830] | 0,6399 | 2,77E-04 | 3,73E-03 |
| ENST00000379895 | ENST00000379895 | Immunglobulin heavy chain variable region (Fragment). [Source:Uniprot/SPTREMBL;Acc:Q0ZCI9] [ENST00000379895] | 1,2604 | 2,78E-04 | 3,74E-03 |
| PLEKHH3 | NM_024927 | Homo sapiens pleckstrin homology domain containing, family H (with MyTH4 domain) member 3 (PLEKHH3), mRNA [NM_024927] | 1,2301 | 2,79E-04 | 3,75E-03 |
| SLC11A2 | NM_000617 | Homo sapiens solute carrier family 11 (proton-coupled divalent metal ion transporters), member 2 (SLC11A2), mRNA [NM_000617] | 0,8742 | 2,78E-04 | 3,75E-03 |
| TPX2 | NM_012112 | Homo sapiens TPX2, microtubule-associated, homolog (Xenopus laevis) (TPX2), mRNA [NM_012112] | 0,6163 | 2,80E-04 | 3,76E-03 |
| RNF5 | NM_006913 | Homo sapiens ring finger protein 5 (RNF5), mRNA [NM_006913] | 0,7314 | 2,81E-04 | 3,76E-03 |
| K-ALPHA-1 | NM_006082 | Homo sapiens alpha tubulin (K-ALPHA-1), mRNA [NM_006082] | 0,7284 | 2,82E-04 | 3,77E-03 |
| DKFZp779O175 | ENST00000330692 | CDNA FLJ27459 fis, clone TST05904. (Fragment). [Source:Uniprot/SPTREMBL;Acc:Q6ZNN0] [ENST00000330692] | 0,6529 | 2,82E-04 | 3,77E-03 |
| Z18824 | Z18824 | Homo sapiens rearranged Ig H-chain V-domain cDNA. [Z18824] | 1,1653 | 2,82E-04 | 3,78E-03 |
| C1orf142 | NM_053052 | Homo sapiens chromosome 1 open reading frame 142 (C1orf142), mRNA [NM_053052] | 0,6155 | 2,84E-04 | 3,79E-03 |
| RNH1 | NM_002939 | Homo sapiens ribonuclease/angiogenin inhibitor 1 (RNH1), transcript variant 1, mRNA [NM_002939] | 0,6919 | 2,89E-04 | 3,84E-03 |
| SEH1L | NM_031216 | Homo sapiens SEH1-like (S. cerevisiae) (SEH1L), transcript variant 2, mRNA [NM_031216] | 0,6693 | 2,90E-04 | 3,86E-03 |
| IL18BP | NM_173042 | Homo sapiens interleukin 18 binding protein (IL18BP), transcript variant A, mRNA [NM_173042] | 0,6619 | 2,93E-04 | 3,89E-03 |
| NCR3 | NM_147130 | Homo sapiens natural cytotoxicity triggering receptor 3 (NCR3), mRNA [NM_147130] | 0,9360 | 2,94E-04 | 3,90E-03 |
| DTYMK | NM_012145 | Homo sapiens deoxythymidylate kinase (thymidylate kinase) (DTYMK), mRNA [NM_012145] | 0,7277 | 2,95E-04 | 3,91E-03 |
| SPRYD4 | ENST00000338146 | SPRY domain-containing protein 4. [Source:Uniprot/SWISSPROT;Acc:Q8WW59] [ENST00000338146] | 0,5207 | 2,95E-04 | 3,91E-03 |
| NAPA | NM_003827 | Homo sapiens N-ethylmaleimide-sensitive factor attachment protein, alpha (NAPA), mRNA [NM_003827] | 0,6957 | 2,98E-04 | 3,95E-03 |
| THC2515746 | THC2515746 | Unknown | 0,6928 | 3,04E-04 | 4,01E-03 |
| PIK3R2 | NM_005027 | Homo sapiens phosphoinositide-3-kinase, regulatory subunit 2 (p85 beta) (PIK3R2), mRNA [NM_005027] | 0,4411 | 3,05E-04 | 4,03E-03 |
| ZNF653 | NM_138783 | Homo sapiens zinc finger protein 653 (ZNF653), mRNA [NM_138783] | 0,5025 | 3,06E-04 | 4,04E-03 |
| CCS | NM_005125 | Homo sapiens copper chaperone for superoxide dismutase (CCS), mRNA [NM_005125] | 0,8384 | 3,07E-04 | 4,04E-03 |
| ADD1 | NM_176801 | Homo sapiens adducin 1 (alpha) (ADD1), transcript variant 4, mRNA [NM_176801] | 0,5631 | 3,09E-04 | 4,06E-03 |
| NOLA2 | NM_017838 | Homo sapiens nucleolar protein family A, member 2 (H/ACA small nucleolar RNPs) (NOLA2), transcript variant 1, mRNA [NM_017838] | 0,6176 | 3,09E-04 | 4,06E-03 |
| C1orf93 | NM_152371 | Homo sapiens chromosome 1 open reading frame 93 (C1orf93), mRNA [NM_152371] | 0,7307 | 3,12E-04 | 4,08E-03 |
| C20orf4 | NM_015511 | Homo sapiens chromosome 20 open reading frame 4 (C20orf4), mRNA [NM_015511] | 0,6694 | 3,11E-04 | 4,08E-03 |
| THC2660636 | THC2660636 | Unknown | 0,7392 | 3,11E-04 | 4,08E-03 |
| MRPL46 | NM_022163 | Homo sapiens mitochondrial ribosomal protein L46 (MRPL46), nuclear gene encoding mitochondrial protein, mRNA [NM_022163] | 0,6386 | 3,14E-04 | 4,10E-03 |
| E2F7 | NM_203394 | Homo sapiens E2F transcription factor 7 (E2F7), mRNA [NM_203394] | 0,9166 | 3,15E-04 | 4,11E-03 |
| DGCR6L | NM_033257 | Homo sapiens DiGeorge syndrome critical region gene 6-like (DGCR6L), mRNA [NM_033257] | 0,7542 | 3,17E-04 | 4,13E-03 |
| C21orf45 | NM_018944 | Homo sapiens chromosome 21 open reading frame 45 (C21orf45), mRNA [NM_018944] | 0,5691 | 3,18E-04 | 4,14E-03 |
| HNRPUL1 | NM_007040 | Homo sapiens heterogeneous nuclear ribonucleoprotein U-like 1 (HNRPUL1), transcript variant 1, mRNA [NM_007040] | 0,4976 | 3,20E-04 | 4,16E-03 |
| ALDH5A1 | NM_170740 | Homo sapiens aldehyde dehydrogenase 5 family, member A1 (succinate-semialdehyde dehydrogenase) (ALDH5A1), nuclear gene encoding mitochondrial protein, transcript variant 1, mRNA [NM_170740] | 0,5584 | 3,21E-04 | 4,17E-03 |
| PSMB5 | NM_002797 | Homo sapiens proteasome (prosome, macropain) subunit, beta type, 5 (PSMB5), mRNA [NM_002797] | 0,7082 | 3,22E-04 | 4,18E-03 |
| FBXO21 | NM_033624 | Homo sapiens F-box protein 21 (FBXO21), transcript variant 1, mRNA [NM_033624] | 0,7433 | 3,26E-04 | 4,22E-03 |
| IMP3 | NM_018285 | Homo sapiens IMP3, U3 small nucleolar ribonucleoprotein, homolog (yeast) (IMP3), mRNA [NM_018285] | 0,6787 | 3,27E-04 | 4,23E-03 |
| BCL11B | NM_138576 | Homo sapiens B-cell CLL/lymphoma 11B (zinc finger protein) (BCL11B), transcript variant 1, mRNA [NM_138576] | 1,1601 | 3,31E-04 | 4,28E-03 |
| NP109842 | NP109842 | GB\|Z29609.1\|CAA82726.1 immunoglobulin heavy chain variable region (HC16-12) [NP109842] | 0,6869 | 3,31E-04 | 4,28E-03 |
| YOD1 | NM_018566 | Homo sapiens YOD1 OTU deubiquinating enzyme 1 homolog (S. cerevisiae) (YOD1), mRNA [NM_018566] | 0,9368 | 3,36E-04 | 4,34E-03 |
| CIT | NM_007174 | Homo sapiens citron (rho-interacting, serine/threonine kinase 21) (CIT), mRNA [NM_007174] | 0,8906 | 3,37E-04 | 4,34E-03 |
| THOC6 | NM_024339 | Homo sapiens THO complex 6 homolog (Drosophila) (THOC6), mRNA [NM_024339] | 0,4905 | 3,37E-04 | 4,34E-03 |
| TIMM22 | NM_013337 | Homo sapiens translocase of inner mitochondrial membrane 22 homolog (yeast) (TIMM22), mRNA [NM_013337] | 0,5752 | 3,37E-04 | 4,34E-03 |
| WBSCR22 | NM_017528 | Homo sapiens Williams Beuren syndrome chromosome region 22 (WBSCR22), mRNA [NM_017528] | 0,5793 | 3,39E-04 | 4,36E-03 |
| TUBB4 | NM_006087 | Homo sapiens tubulin, beta 4 (TUBB4), mRNA [NM_006087] | 0,9076 | 3,42E-04 | 4,39E-03 |
| TMEM64 | AK095472 | Homo sapiens cDNA FLJ38153 fis, clone DFNES1000083. [AK095472] | 0,6673 | 3,42E-04 | 4,39E-03 |
| DLG7 | NM_014750 | Homo sapiens discs, large homolog 7 (Drosophila) (DLG7), mRNA [NM_014750] | 0,7859 | 3,45E-04 | 4,42E-03 |
| FAHD2A | NM_016044 | Homo sapiens fumarylacetoacetate hydrolase domain containing 2A (FAHD2A), mRNA [NM_016044] | 0,6206 | 3,45E-04 | 4,42E-03 |
| BF965065 | BF965065 | 602268829F1 NIH_MGC_81 Homo sapiens cDNA clone IMAGE:4356966 5', mRNA sequence [BF965065] | 0,7444 | 3,46E-04 | 4,42E-03 |
| LDOC1L | NM_032287 | Homo sapiens leucine zipper, down-regulated in cancer 1-like (LDOC1L), mRNA [NM_032287] | 0,6853 | 3,49E-04 | 4,46E-03 |
| PPIA | NM_021130 | Homo sapiens peptidylprolyl isomerase A (cyclophilin A) (PPIA), mRNA [NM_021130] | 0,6375 | 3,50E-04 | 4,47E-03 |
| RFESD | NM_173362 | Homo sapiens Rieske (Fe-S) domain containing (RFESD), mRNA [NM_173362] | 1,1769 | 3,51E-04 | 4,48E-03 |
| ENST00000360102 | ENST00000360102 | Homo sapiens isolate D2-P-4-VH3-49 immunoglobulin heavy chain variable region mRNA, partial cds. [DQ840829] | 1,0354 | 3,57E-04 | 4,54E-03 |
| DPP3 | NM_130443 | Homo sapiens dipeptidyl-peptidase 3 (DPP3), transcript variant 2, mRNA [NM_130443] | 0,6553 | 3,58E-04 | 4,54E-03 |
| MCM4 | NM_005914 | Homo sapiens MCM4 minichromosome maintenance deficient 4 (S. cerevisiae) (MCM4), transcript variant 1, mRNA [NM_005914] | 0,5474 | 3,58E-04 | 4,55E-03 |
| PCK2 | NM_004563 | Homo sapiens phosphoenolpyruvate carboxykinase 2 (mitochondrial) (PCK2), nuclear gene encoding mitochondrial protein, transcript variant 1, mRNA [NM_004563] | 0,9040 | 3,60E-04 | 4,56E-03 |
| MAL | NM_002371 | Homo sapiens mal, T-cell differentiation protein (MAL), transcript variant a, mRNA [NM_002371] | 1,4064 | 3,61E-04 | 4,57E-03 |
| VCAM1 | NM_001078 | Homo sapiens vascular cell adhesion molecule 1 (VCAM1), transcript variant 1, mRNA [NM_001078] | 0,9627 | 3,61E-04 | 4,57E-03 |
| LOC652012 | XR_019252 | PREDICTED: Homo sapiens similar to 60S ribosomal protein L7a (Surfeit locus protein 3) (LOC652012), mRNA [XR_019252] | 0,5526 | 3,62E-04 | 4,58E-03 |
| TGS1 | NM_024831 | Homo sapiens trimethylguanosine synthase homolog (S. cerevisiae) (TGS1), mRNA [NM_024831] | 0,4252 | 3,63E-04 | 4,59E-03 |
| NAGPA | NM_016256 | Homo sapiens N-acetylglucosamine-1-phosphodiester alpha-N-acetylglucosaminidase (NAGPA), mRNA [NM_016256] | 0,6111 | 3,64E-04 | 4,59E-03 |
| WNK1 | NM_018979 | Homo sapiens WNK lysine deficient protein kinase 1 (WNK1), mRNA [NM_018979] | 0,9908 | 3,66E-04 | 4,61E-03 |
| FCER1A | NM_002001 | Homo sapiens Fc fragment of IgE, high affinity I, receptor for; alpha polypeptide (FCER1A), mRNA [NM_002001] | 1,3481 | 3,66E-04 | 4,62E-03 |
| CCNB1 | NM_031966 | Homo sapiens cyclin B1 (CCNB1), mRNA [NM_031966] | 0,7901 | 3,71E-04 | 4,67E-03 |
| PCGF5 | NM_032373 | Homo sapiens polycomb group ring finger 5 (PCGF5), mRNA [NM_032373] | 0,7363 | 3,73E-04 | 4,69E-03 |
| C6orf59 | AK026765 | Homo sapiens cDNA: FLJ23112 fis, clone LNG07874. [AK026765] | 1,2070 | 3,76E-04 | 4,72E-03 |
| BC007606 | BC007606 | Homo sapiens cDNA clone IMAGE:3351130, complete cds. [BC007606] | 0,6830 | 3,77E-04 | 4,74E-03 |
| DCN | NM_001920 | Homo sapiens decorin (DCN), transcript variant A1, mRNA [NM_001920] | 1,0309 | 3,80E-04 | 4,77E-03 |
| LOC388524 | NM_001005472 | Homo sapiens similar to Laminin receptor 1 (LOC388524), mRNA [NM_001005472] | 0,6450 | 3,80E-04 | 4,77E-03 |
| P4HB | NM_000918 | Homo sapiens procollagen-proline, 2-oxoglutarate 4-dioxygenase (proline 4-hydroxylase), beta polypeptide (P4HB), mRNA [NM_000918] | 1,1052 | 3,80E-04 | 4,77E-03 |
| COPE | NM_007263 | Homo sapiens coatomer protein complex, subunit epsilon (COPE), transcript variant 1, mRNA [NM_007263] | 0,4989 | 3,82E-04 | 4,78E-03 |
| IQSEC2 | NM_015075 | Homo sapiens IQ motif and Sec7 domain 2 (IQSEC2), mRNA [NM_015075] | 2,1137 | 3,83E-04 | 4,79E-03 |
| HN1L | NM_144570 | Homo sapiens hematological and neurological expressed 1-like (HN1L), mRNA [NM_144570] | 0,8458 | 3,84E-04 | 4,80E-03 |
| THC2487640 | THC2487640 | Unknown | 1,0748 | 3,85E-04 | 4,81E-03 |
| AK098422 | AK098422 | Homo sapiens cDNA FLJ25556 fis, clone JTH02629. [AK098422] | 0,7971 | 3,86E-04 | 4,82E-03 |
| A_23_P51966 | A_23_P51966 | Unknown | 0,5401 | 3,89E-04 | 4,85E-03 |
| CD2AP | NM_012120 | Homo sapiens CD2-associated protein (CD2AP), mRNA [NM_012120] | 0,3861 | 3,90E-04 | 4,86E-03 |
| POLD2 | NM_006230 | Homo sapiens polymerase (DNA directed), delta 2, regulatory subunit 50kDa (POLD2), mRNA [NM_006230] | 0,7469 | 3,92E-04 | 4,88E-03 |
| MRPL49 | NM_004927 | Homo sapiens mitochondrial ribosomal protein L49 (MRPL49), nuclear gene encoding mitochondrial protein, mRNA [NM_004927] | 0,7868 | 3,92E-04 | 4,88E-03 |
| BM479752 | BM479752 | AGENCOURT_6465050 NIH_MGC_92 Homo sapiens cDNA clone IMAGE:5577282 5', mRNA sequence [BM479752] | 0,6067 | 3,93E-04 | 4,89E-03 |
| AK098081 | AK098081 | Homo sapiens cDNA FLJ40762 fis, clone TRACH2002847. [AK098081] | 0,5707 | 3,96E-04 | 4,91E-03 |
| LOC643013 | XR_018155 | PREDICTED: Homo sapiens similar to laminin receptor 1 (ribosomal protein SA) (LOC643013), mRNA [XR_018155] | 0,6040 | 3,97E-04 | 4,91E-03 |
| DGCR8 | NM_022720 | Homo sapiens DiGeorge syndrome critical region gene 8 (DGCR8), mRNA [NM_022720] | 0,8331 | 3,98E-04 | 4,92E-03 |
| GRRP1 | NM_024869 | Homo sapiens glycine/arginine rich protein 1 (GRRP1), mRNA [NM_024869] | 0,8438 | 3,98E-04 | 4,92E-03 |
| NDUFV3 | NM_021075 | Homo sapiens NADH dehydrogenase (ubiquinone) flavoprotein 3, 10kDa (NDUFV3), nuclear gene encoding mitochondrial protein, transcript variant 1, mRNA [NM_021075] | 0,6600 | 4,03E-04 | 4,98E-03 |
| TSPAN4 | NM_001025237 | Homo sapiens tetraspanin 4 (TSPAN4), transcript variant 1, mRNA [NM_001025237] | 0,7629 | 4,06E-04 | 5,00E-03 |
| TMEM57 | NM_018202 | Homo sapiens transmembrane protein 57 (TMEM57), mRNA [NM_018202] | 1,0758 | 4,07E-04 | 5,01E-03 |
| CD8A | NM_001768 | Homo sapiens CD8a molecule (CD8A), transcript variant 1, mRNA [NM_001768] | 1,5803 | 4,09E-04 | 5,03E-03 |
| COG8 | NM_032382 | Homo sapiens component of oligomeric golgi complex 8 (COG8), mRNA [NM_032382] | 0,5130 | 4,09E-04 | 5,03E-03 |
| LOC494150 | BC014228 | Homo sapiens prohibitin pseudogene, mRNA (cDNA clone MGC:20874 IMAGE:4547239), complete cds. [BC014228] | 0,6341 | 4,10E-04 | 5,03E-03 |
| RRM1 | NM_001033 | Homo sapiens ribonucleotide reductase M1 polypeptide (RRM1), mRNA [NM_001033] | 0,6751 | 4,11E-04 | 5,05E-03 |
| TK1 | NM_003258 | Homo sapiens thymidine kinase 1, soluble (TK1), mRNA [NM_003258] | 0,7099 | 4,12E-04 | 5,06E-03 |
| PAFAH1B1 | NM_000430 | Homo sapiens platelet-activating factor acetylhydrolase, isoform Ib, alpha subunit 45kDa (PAFAH1B1), mRNA [NM_000430] | 0,9405 | 4,14E-04 | 5,08E-03 |
| EPPB9 | NM_015681 | Homo sapiens B9 protein (EPPB9), mRNA [NM_015681] | 0,7328 | 4,18E-04 | 5,12E-03 |
| EHD3 | NM_014600 | Homo sapiens EH-domain containing 3 (EHD3), mRNA [NM_014600] | 0,5717 | 4,19E-04 | 5,12E-03 |
| C18orf10 | NM_015476 | Homo sapiens chromosome 18 open reading frame 10 (C18orf10), mRNA [NM_015476] | 0,9181 | 4,20E-04 | 5,12E-03 |
| TLOC1 | NM_003262 | Homo sapiens translocation protein 1 (TLOC1), mRNA [NM_003262] | 0,9547 | 4,20E-04 | 5,12E-03 |
| MED9 | NM_018019 | Homo sapiens mediator of RNA polymerase II transcription, subunit 9 homolog (S. cerevisiae) (MED9), mRNA [NM_018019] | 0,6998 | 4,24E-04 | 5,16E-03 |
| IL2RB | NM_000878 | Homo sapiens interleukin 2 receptor, beta (IL2RB), mRNA [NM_000878] | 1,4101 | 4,25E-04 | 5,17E-03 |
| ATRN | NM_139322 | Homo sapiens attractin (ATRN), transcript variant 2, mRNA [NM_139322] | 0,8871 | 4,27E-04 | 5,19E-03 |
| NUP93 | NM_014669 | Homo sapiens nucleoporin 93kDa (NUP93), mRNA [NM_014669] | 0,8275 | 4,27E-04 | 5,19E-03 |
| RRM2 | NM_001034 | Homo sapiens ribonucleotide reductase M2 polypeptide (RRM2), mRNA [NM_001034] | 0,8757 | 4,27E-04 | 5,19E-03 |
| IGHA1 | AF067420 | Homo sapiens SNC73 protein (SNC73) mRNA, complete cds. [AF067420] | 1,3330 | 4,31E-04 | 5,22E-03 |
| PI4KII | NM_018425 | Homo sapiens phosphatidylinositol 4-kinase type II (PI4KII), mRNA [NM_018425] | 1,1790 | 4,32E-04 | 5,23E-03 |
| SND1 | NM_014390 | Homo sapiens staphylococcal nuclease and tudor domain containing 1 (SND1), mRNA [NM_014390] | 0,6171 | 4,33E-04 | 5,23E-03 |
| SLC43A3 | NM_199329 | Homo sapiens solute carrier family 43, member 3 (SLC43A3), mRNA [NM_199329] | 0,7291 | 4,33E-04 | 5,24E-03 |
| SLC39A3 | NM_213568 | Homo sapiens solute carrier family 39 (zinc transporter), member 3 (SLC39A3), transcript variant 2, mRNA [NM_213568] | 0,5777 | 4,35E-04 | 5,26E-03 |
| CRYL1 | NM_015974 | Homo sapiens crystallin, lambda 1 (CRYL1), mRNA [NM_015974] | 0,8485 | 4,38E-04 | 5,28E-03 |
| TUBB6 | NM_032525 | Homo sapiens tubulin, beta 6 (TUBB6), mRNA [NM_032525] | 0,6959 | 4,40E-04 | 5,30E-03 |
| CR617560 | CR617560 | full-length cDNA clone CS0DC013YG14 of Neuroblastoma Cot 25-normalized of Homo sapiens (human). [CR617560] | 0,6323 | 4,45E-04 | 5,35E-03 |
| UMPS | NM_000373 | Homo sapiens uridine monophosphate synthetase (orotate phosphoribosyl transferase and orotidine-5'-decarboxylase) (UMPS), mRNA [NM_000373] | 0,9169 | 4,46E-04 | 5,36E-03 |
| C20orf55 | NM_001042353 | Homo sapiens chromosome 20 open reading frame 55 (C20orf55), transcript variant 3, mRNA [NM_001042353] | 0,6104 | 4,49E-04 | 5,39E-03 |
| ZNF777 | NM_015694 | Homo sapiens zinc finger protein 777 (ZNF777), mRNA [NM_015694] | 0,6910 | 4,52E-04 | 5,42E-03 |
| WDR89 | NM_001008726 | Homo sapiens WD repeat domain 89 (WDR89), transcript variant 1, mRNA [NM_001008726] | 0,4770 | 4,54E-04 | 5,43E-03 |
| DEXI | NM_014015 | Homo sapiens dexamethasone-induced transcript (DEXI), mRNA [NM_014015] | 0,5877 | 4,61E-04 | 5,52E-03 |
| CLPB | NM_030813 | Homo sapiens ClpB caseinolytic peptidase B homolog (E. coli) (CLPB), mRNA [NM_030813] | 0,6532 | 4,69E-04 | 5,58E-03 |
| COL4A2 | NM_001846 | Homo sapiens collagen, type IV, alpha 2 (COL4A2), mRNA [NM_001846] | 0,6028 | 4,69E-04 | 5,58E-03 |
| CPVL | NM_019029 | Homo sapiens carboxypeptidase, vitellogenic-like (CPVL), transcript variant 2, mRNA [NM_019029] | 0,9453 | 4,69E-04 | 5,58E-03 |
| TIGD5 | NM_032862 | Homo sapiens tigger transposable element derived 5 (TIGD5), mRNA [NM_032862] | 0,5859 | 4,69E-04 | 5,58E-03 |
| CTNNAL1 | NM_003798 | Homo sapiens catenin (cadherin-associated protein), alpha-like 1 (CTNNAL1), mRNA [NM_003798] | 0,8235 | 4,71E-04 | 5,60E-03 |
| LOC442308 | XR_018043 | PREDICTED: Homo sapiens similar to tubulin, beta 5 (LOC442308), mRNA [XR_018043] | 0,9829 | 4,72E-04 | 5,60E-03 |
| THC2730631 | THC2730631 | ARL9_HUMAN (Q6T311) ADP-ribosylation factor-like protein 9, partial (39%) [THC2730631] | 0,7161 | 4,73E-04 | 5,62E-03 |
| GYPE | NM_002102 | Homo sapiens glycophorin E (GYPE), transcript variant 1, mRNA [NM_002102] | 1,3222 | 4,76E-04 | 5,64E-03 |
| NAT12 | BC048983 | Homo sapiens N-acetyltransferase 12, mRNA (cDNA clone IMAGE:5259876). [BC048983] | 0,5700 | 4,75E-04 | 5,64E-03 |
| PSKH1 | NM_006742 | Homo sapiens protein serine kinase H1 (PSKH1), mRNA [NM_006742] | 0,8583 | 4,78E-04 | 5,66E-03 |
| FBXO31 | AK026130 | Homo sapiens cDNA: FLJ22477 fis, clone HRC10815. [AK026130] | 0,7825 | 4,78E-04 | 5,66E-03 |
| ALG1 | NM_019109 | Homo sapiens asparagine-linked glycosylation 1 homolog (S. cerevisiae, beta-1,4-mannosyltransferase) (ALG1), mRNA [NM_019109] | 0,5971 | 4,81E-04 | 5,69E-03 |
| NOL9 | ENST00000377705 | Nucleolar protein 9. [Source:Uniprot/SWISSPROT;Acc:Q5SY16] [ENST00000377705] | 0,6491 | 4,83E-04 | 5,70E-03 |
| WSB2 | NM_018639 | Homo sapiens WD repeat and SOCS box-containing 2 (WSB2), mRNA [NM_018639] | 0,6351 | 4,83E-04 | 5,70E-03 |
| MCM7 | NM_182776 | Homo sapiens MCM7 minichromosome maintenance deficient 7 (S. cerevisiae) (MCM7), transcript variant 2, mRNA [NM_182776] | 0,7640 | 4,94E-04 | 5,83E-03 |
| CCNB2 | NM_004701 | Homo sapiens cyclin B2 (CCNB2), mRNA [NM_004701] | 0,6620 | 4,95E-04 | 5,83E-03 |
| BE893137 | BE893137 | 601437034F1 NIH_MGC_72 Homo sapiens cDNA clone IMAGE:3922112 5', mRNA sequence [BE893137] | 0,5162 | 4,96E-04 | 5,84E-03 |
| LOC158345 | XR_017130 | PREDICTED: Homo sapiens similar to ribosomal protein L4 (LOC158345), mRNA [XR_017130] | 0,7785 | 5,04E-04 | 5,92E-03 |
| ERGIC3 | NM_198398 | Homo sapiens ERGIC and golgi 3 (ERGIC3), transcript variant 1, mRNA [NM_198398] | 0,6934 | 5,07E-04 | 5,96E-03 |
| ASNA1 | NM_004317 | Homo sapiens arsA arsenite transporter, ATP-binding, homolog 1 (bacterial) (ASNA1), mRNA [NM_004317] | 0,8123 | 5,18E-04 | 6,07E-03 |
| HINT2 | NM_032593 | Homo sapiens histidine triad nucleotide binding protein 2 (HINT2), mRNA [NM_032593] | 0,8336 | 5,19E-04 | 6,08E-03 |
| C19orf52 | NM_138358 | Homo sapiens chromosome 19 open reading frame 52 (C19orf52), mRNA [NM_138358] | 0,5189 | 5,22E-04 | 6,11E-03 |
| DNAJC11 | NM_018198 | Homo sapiens DnaJ (Hsp40) homolog, subfamily C, member 11 (DNAJC11), mRNA [NM_018198] | 0,5536 | 5,24E-04 | 6,12E-03 |
| CIZ1 | NM_012127 | Homo sapiens CDKN1A interacting zinc finger protein 1 (CIZ1), mRNA [NM_012127] | 0,5059 | 5,25E-04 | 6,14E-03 |
| RNF10 | NM_014868 | Homo sapiens ring finger protein 10 (RNF10), mRNA [NM_014868] | 0,7252 | 5,28E-04 | 6,16E-03 |
| USP5 | NM_003481 | Homo sapiens ubiquitin specific peptidase 5 (isopeptidase T) (USP5), mRNA [NM_003481] | 0,7041 | 5,29E-04 | 6,17E-03 |
| SUPT16H | NM_007192 | Homo sapiens suppressor of Ty 16 homolog (S. cerevisiae) (SUPT16H), mRNA [NM_007192] | 0,6265 | 5,31E-04 | 6,18E-03 |
| MTCH2 | NM_014342 | Homo sapiens mitochondrial carrier homolog 2 (C. elegans) (MTCH2), nuclear gene encoding mitochondrial protein, mRNA [NM_014342] | 0,5554 | 5,37E-04 | 6,26E-03 |
| VANGL1 | NM_138959 | Homo sapiens vang-like 1 (van gogh, Drosophila) (VANGL1), mRNA [NM_138959] | 0,5203 | 5,39E-04 | 6,27E-03 |
| THC2527647 | THC2527647 | Q59GY2_HUMAN (Q59GY2) Ribosomal protein L4 variant (Fragment), partial (51%) [THC2527647] | 0,6128 | 5,43E-04 | 6,30E-03 |
| DNAJB2 | NM_001039550 | Homo sapiens DnaJ (Hsp40) homolog, subfamily B, member 2 (DNAJB2), transcript variant 1, mRNA [NM_001039550] | 0,8581 | 5,44E-04 | 6,31E-03 |
| THC2516487 | THC2516487 | Unknown | 0,8524 | 5,47E-04 | 6,34E-03 |
| RHBDD1 | NM_032276 | Homo sapiens rhomboid domain containing 1 (RHBDD1), mRNA [NM_032276] | 0,5217 | 5,49E-04 | 6,36E-03 |
| C20orf121 | NM_024331 | Homo sapiens chromosome 20 open reading frame 121 (C20orf121), transcript variant 1, mRNA [NM_024331] | 0,5562 | 5,51E-04 | 6,38E-03 |
| NIP7 | NM_016101 | Homo sapiens nuclear import 7 homolog (S. cerevisiae) (NIP7), mRNA [NM_016101] | 0,5560 | 5,52E-04 | 6,39E-03 |
| AK055981 | AK055981 | Homo sapiens cDNA FLJ31419 fis, clone NT2NE2000356. [AK055981] | 0,8045 | 5,54E-04 | 6,40E-03 |
| CCDC117 | NM_173510 | Homo sapiens coiled-coil domain containing 117 (CCDC117), mRNA [NM_173510] | 0,5061 | 5,55E-04 | 6,41E-03 |
| KIAA1303 | NM_020761 | Homo sapiens raptor (KIAA1303), mRNA [NM_020761] | 0,7792 | 5,56E-04 | 6,42E-03 |
| UNQ1887 | NM_139015 | Homo sapiens signal peptide peptidase 3 (SPPL3), mRNA [NM_139015] | 0,8906 | 5,56E-04 | 6,42E-03 |
| A_23_P72252 | A_23_P72252 | Unknown | 1,1093 | 5,58E-04 | 6,43E-03 |
| BC014395 | BC014395 | Homo sapiens, clone IMAGE:3029191, mRNA. [BC014395] | 0,9742 | 5,60E-04 | 6,44E-03 |
| GPR175 | NM_016372 | Homo sapiens G protein-coupled receptor 175 (GPR175), mRNA [NM_016372] | 0,5512 | 5,60E-04 | 6,44E-03 |
| GTPBP4 | ENST00000381391 | Nucleolar GTP-binding protein 1 (Chronic renal failure gene protein) (GTP-binding protein NGB). [Source:Uniprot/SWISSPROT;Acc:Q9BZE4] [ENST00000381391] | 0,7597 | 5,60E-04 | 6,44E-03 |
| PSMD1 | NM_002807 | Homo sapiens proteasome (prosome, macropain) 26S subunit, non-ATPase, 1 (PSMD1), mRNA [NM_002807] | 0,5939 | 5,60E-04 | 6,44E-03 |
| TRAP1 | NM_016292 | Homo sapiens TNF receptor-associated protein 1 (TRAP1), mRNA [NM_016292] | 0,5925 | 5,60E-04 | 6,44E-03 |
| PSMB6 | NM_002798 | Homo sapiens proteasome (prosome, macropain) subunit, beta type, 6 (PSMB6), mRNA [NM_002798] | 0,5132 | 5,63E-04 | 6,45E-03 |
| LY9 | NM_002348 | Homo sapiens lymphocyte antigen 9 (LY9), transcript variant 1, mRNA [NM_002348] | 0,7538 | 5,72E-04 | 6,54E-03 |
| KLF13 | NM_015995 | Homo sapiens Kruppel-like factor 13 (KLF13), mRNA [NM_015995] | 0,9243 | 5,77E-04 | 6,56E-03 |
| PDIK1L | NM_152835 | Homo sapiens PDLIM1 interacting kinase 1 like (PDIK1L), mRNA [NM_152835] | 0,5584 | 5,75E-04 | 6,56E-03 |
| TOE1 | BC009364 | Homo sapiens target of EGR1, member 1 (nuclear), mRNA (cDNA clone MGC:14971 IMAGE:4302712), complete cds. [BC009364] | 0,8846 | 5,75E-04 | 6,56E-03 |
| TRIM59 | NM_173084 | Homo sapiens tripartite motif-containing 59 (TRIM59), mRNA [NM_173084] | 0,9394 | 5,77E-04 | 6,56E-03 |
| TXN2 | NM_012473 | Homo sapiens thioredoxin 2 (TXN2), nuclear gene encoding mitochondrial protein, mRNA [NM_012473] | 0,5629 | 5,77E-04 | 6,56E-03 |
| CD99L2 | NM_031462 | Homo sapiens CD99 molecule-like 2 (CD99L2), transcript variant 1, mRNA [NM_031462] | 0,6873 | 5,79E-04 | 6,58E-03 |
| NUDT21 | NM_007006 | Homo sapiens nudix (nucleoside diphosphate linked moiety X)-type motif 21 (NUDT21), mRNA [NM_007006] | 0,6382 | 5,80E-04 | 6,59E-03 |
| TMEM111 | NM_018447 | Homo sapiens transmembrane protein 111 (TMEM111), mRNA [NM_018447] | 0,8588 | 5,83E-04 | 6,62E-03 |
| NUP133 | NM_018230 | Homo sapiens nucleoporin 133kDa (NUP133), mRNA [NM_018230] | 0,5917 | 5,84E-04 | 6,63E-03 |
| KHSRP | NM_003685 | Homo sapiens KH-type splicing regulatory protein (FUSE binding protein 2) (KHSRP), mRNA [NM_003685] | 0,7414 | 5,91E-04 | 6,69E-03 |
| EIF5A | NM_001970 | Homo sapiens eukaryotic translation initiation factor 5A (EIF5A), mRNA [NM_001970] | 0,8549 | 5,91E-04 | 6,70E-03 |
| HMGA1 | NM_145904 | Homo sapiens high mobility group AT-hook 1 (HMGA1), transcript variant 6, mRNA [NM_145904] | 0,7294 | 5,93E-04 | 6,71E-03 |
| KIAA1147 | BC012493 | Homo sapiens KIAA1147, mRNA (cDNA clone IMAGE:4470021), complete cds. [BC012493] | 0,6244 | 5,94E-04 | 6,72E-03 |
| RMND5B | AK094065 | Homo sapiens cDNA FLJ36746 fis, clone UTERU2016757. [AK094065] | 0,7542 | 5,94E-04 | 6,72E-03 |
| CD59 | NM_203330 | Homo sapiens CD59 molecule, complement regulatory protein (CD59), transcript variant 1, mRNA [NM_203330] | 0,8574 | 5,96E-04 | 6,74E-03 |
| RAD51C | NM_002876 | Homo sapiens RAD51 homolog C (S. cerevisiae) (RAD51C), transcript variant 2, mRNA [NM_002876] | 0,6407 | 5,98E-04 | 6,75E-03 |
| CCDC94 | NM_018074 | Homo sapiens coiled-coil domain containing 94 (CCDC94), mRNA [NM_018074] | 0,7181 | 6,01E-04 | 6,79E-03 |
| FLJ12331 | NM_024986 | Homo sapiens hypothetical protein FLJ12331 (FLJ12331), mRNA [NM_024986] | 0,9424 | 6,02E-04 | 6,79E-03 |
| PTPN11 | NM_002834 | Homo sapiens protein tyrosine phosphatase, non-receptor type 11 (Noonan syndrome 1) (PTPN11), mRNA [NM_002834] | 0,5976 | 6,07E-04 | 6,84E-03 |
| CXCR3 | NM_001504 | Homo sapiens chemokine (C-X-C motif) receptor 3 (CXCR3), mRNA [NM_001504] | 1,5335 | 6,09E-04 | 6,85E-03 |
| LGR6 | NM_001017403 | Homo sapiens leucine-rich repeat-containing G protein-coupled receptor 6 (LGR6), transcript variant 1, mRNA [NM_001017403] | 0,7757 | 6,09E-04 | 6,85E-03 |
| TMEM138 | NM_016464 | Homo sapiens transmembrane protein 138 (TMEM138), mRNA [NM_016464] | 0,6023 | 6,10E-04 | 6,85E-03 |
| ATP5A1 | NM_001001937 | Homo sapiens ATP synthase, H+ transporting, mitochondrial F1 complex, alpha subunit 1, cardiac muscle (ATP5A1), nuclear gene encoding mitochondrial protein, transcript variant 1, mRNA [NM_001001937] | 0,5579 | 6,11E-04 | 6,85E-03 |
| FNBP1L | NM_001024948 | Homo sapiens formin binding protein 1-like (FNBP1L), transcript variant 1, mRNA [NM_001024948] | 0,7056 | 6,11E-04 | 6,85E-03 |
| TUBB2C | NM_006088 | Homo sapiens tubulin, beta 2C (TUBB2C), mRNA [NM_006088] | 0,6041 | 6,11E-04 | 6,85E-03 |
| ZNF289 | NM_032389 | Homo sapiens zinc finger protein 289, ID1 regulated (ZNF289), mRNA [NM_032389] | 0,5981 | 6,11E-04 | 6,85E-03 |
| PRKRIR | NM_004705 | Homo sapiens protein-kinase, interferon-inducible double stranded RNA dependent inhibitor, repressor of (P58 repressor) (PRKRIR), mRNA [NM_004705] | 0,5992 | 6,12E-04 | 6,85E-03 |
| SLC25A15 | ENST00000379534 | Mitochondrial ornithine transporter 1 (Solute carrier family 25 member 15). [Source:Uniprot/SWISSPROT;Acc:Q9Y619] [ENST00000379534] | 0,5523 | 6,20E-04 | 6,92E-03 |
| SNX12 | ENST00000374274 | Sorting nexin-12. [Source:Uniprot/SWISSPROT;Acc:Q9UMY4] [ENST00000374274] | 0,6023 | 6,20E-04 | 6,92E-03 |
| AKT1S1 | NM_032375 | Homo sapiens AKT1 substrate 1 (proline-rich) (AKT1S1), mRNA [NM_032375] | 0,5598 | 6,21E-04 | 6,93E-03 |
| NELL2 | NM_006159 | Homo sapiens NEL-like 2 (chicken) (NELL2), mRNA [NM_006159] | 1,1530 | 6,22E-04 | 6,94E-03 |
| HSPC142 | NM_001033549 | Homo sapiens HSPC142 protein (HSPC142), transcript variant 1, mRNA [NM_001033549] | 0,5477 | 6,23E-04 | 6,95E-03 |
| GSG2 | AK056691 | Homo sapiens cDNA FLJ32129 fis, clone PEBLM2000213, weakly similar to Mus musculus genes for integrin aM290, hapsin. [AK056691] | 0,8170 | 6,37E-04 | 7,09E-03 |
| EXOD1 | AB040937 | Homo sapiens mRNA for KIAA1504 protein, partial cds. [AB040937] | 0,5903 | 6,40E-04 | 7,12E-03 |
| UHMK1 | ENST00000282169 | Serine/threonine-protein kinase Kist (EC 2.7.11.1) (Kinase interacting with stathmin) (U2AF homology motif kinase 1). [Source:Uniprot/SWISSPROT;Acc:Q8TAS1] [ENST00000282169] | 0,7756 | 6,42E-04 | 7,13E-03 |
| FLJ22222 | NM_024648 | Homo sapiens hypothetical protein FLJ22222 (FLJ22222), transcript variant 1, mRNA [NM_024648] | 0,7593 | 6,43E-04 | 7,14E-03 |
| RG9MTD1 | NM_017819 | Homo sapiens RNA (guanine-9-) methyltransferase domain containing 1 (RG9MTD1), mRNA [NM_017819] | 0,6609 | 6,47E-04 | 7,17E-03 |
| OLFML2B | NM_015441 | Homo sapiens olfactomedin-like 2B (OLFML2B), mRNA [NM_015441] | 0,6150 | 6,48E-04 | 7,18E-03 |
| FH | NM_000143 | Homo sapiens fumarate hydratase (FH), nuclear gene encoding mitochondrial protein, mRNA [NM_000143] | 0,5460 | 6,49E-04 | 7,19E-03 |
| BTG2 | NM_006763 | Homo sapiens BTG family, member 2 (BTG2), mRNA [NM_006763] | 0,7452 | 6,50E-04 | 7,20E-03 |
| PLOD3 | NM_001084 | Homo sapiens procollagen-lysine, 2-oxoglutarate 5-dioxygenase 3 (PLOD3), mRNA [NM_001084] | 0,6636 | 6,51E-04 | 7,20E-03 |
| CD40 | NM_001250 | Homo sapiens CD40 molecule, TNF receptor superfamily member 5 (CD40), transcript variant 1, mRNA [NM_001250] | 0,5477 | 6,57E-04 | 7,26E-03 |
| EIF3S1 | NM_003758 | Homo sapiens eukaryotic translation initiation factor 3, subunit 1 alpha, 35kDa (EIF3S1), mRNA [NM_003758] | 0,5820 | 6,58E-04 | 7,27E-03 |
| SH3BGRL2 | NM_031469 | Homo sapiens SH3 domain binding glutamic acid-rich protein like 2 (SH3BGRL2), mRNA [NM_031469] | 0,5033 | 6,62E-04 | 7,30E-03 |
| PIR | NM_003662 | Homo sapiens pirin (iron-binding nuclear protein) (PIR), transcript variant 1, mRNA [NM_003662] | 0,9249 | 6,68E-04 | 7,35E-03 |
| A_32_P169353 | A_32_P169353 | Unknown | 0,8020 | 6,69E-04 | 7,36E-03 |
| DCXR | NM_016286 | Homo sapiens dicarbonyl/L-xylulose reductase (DCXR), mRNA [NM_016286] | 0,6505 | 6,72E-04 | 7,37E-03 |
| EMID1 | NM_133455 | Homo sapiens EMI domain containing 1 (EMID1), mRNA [NM_133455] | 0,6638 | 6,72E-04 | 7,37E-03 |
| ENST00000379877 | ENST00000379877 | Full-length cDNA clone CS0DL004YM19 of B cells (Ramos cell line) of Homo sapiens (human) (Fragment). [Source:Uniprot/SPTREMBL;Acc:Q86SX2] [ENST00000379877] | 0,6369 | 6,72E-04 | 7,37E-03 |
| PLK1 | NM_005030 | Homo sapiens polo-like kinase 1 (Drosophila) (PLK1), mRNA [NM_005030] | 0,6978 | 6,72E-04 | 7,37E-03 |
| WDR45 | NM_007075 | Homo sapiens WD repeat domain 45 (WDR45), transcript variant 1, mRNA [NM_007075] | 0,6791 | 6,73E-04 | 7,38E-03 |
| HEXA | S76980 | HEXA {HEXA4bpDeltaA mutation, exon 11} [human, Tay-Sachs disease patient, mRNA Partial Mutant, 78 nt]. [S76980] | 0,6495 | 6,75E-04 | 7,39E-03 |
| C1orf166 | NM_024544 | Homo sapiens chromosome 1 open reading frame 166 (C1orf166), mRNA [NM_024544] | 0,7847 | 6,76E-04 | 7,39E-03 |
| CR611332 | CR611332 | full-length cDNA clone CS0DF014YA22 of Fetal brain of Homo sapiens (human). [CR611332] | 0,5489 | 6,76E-04 | 7,39E-03 |
| ENST00000354689 | ENST00000354689 | Homo sapiens immunoglobulin heavy chain mRNA, partial cds. [AY505570] | 0,5898 | 6,79E-04 | 7,41E-03 |
| TRAM2 | NM_012288 | Homo sapiens translocation associated membrane protein 2 (TRAM2), mRNA [NM_012288] | 0,4710 | 6,84E-04 | 7,47E-03 |
| SMCR7L | NM_019008 | Homo sapiens Smith-Magenis syndrome chromosome region, candidate 7-like (SMCR7L), mRNA [NM_019008] | 0,8996 | 6,87E-04 | 7,49E-03 |
| NUDT4 | NM_199040 | Homo sapiens nudix (nucleoside diphosphate linked moiety X)-type motif 4 (NUDT4), transcript variant 2, mRNA [NM_199040] | 0,9254 | 6,93E-04 | 7,56E-03 |
| BAD | NM_004322 | Homo sapiens BCL2-antagonist of cell death (BAD), transcript variant 1, mRNA [NM_004322] | 0,4995 | 6,97E-04 | 7,58E-03 |
| KLF3 | NM_016531 | Homo sapiens Kruppel-like factor 3 (basic) (KLF3), mRNA [NM_016531] | 0,8364 | 6,97E-04 | 7,58E-03 |
| TMEM9 | NM_016456 | Homo sapiens transmembrane protein 9 (TMEM9), mRNA [NM_016456] | 0,7601 | 6,97E-04 | 7,58E-03 |
| TMEPAI | NM_020182 | Homo sapiens transmembrane, prostate androgen induced RNA (TMEPAI), transcript variant 1, mRNA [NM_020182] | 1,0470 | 6,97E-04 | 7,58E-03 |
| ZNF264 | NM_003417 | Homo sapiens zinc finger protein 264 (ZNF264), mRNA [NM_003417] | 0,5745 | 6,98E-04 | 7,59E-03 |
| CTDSPL | NM_001008392 | Homo sapiens CTD (carboxy-terminal domain, RNA polymerase II, polypeptide A) small phosphatase-like (CTDSPL), transcript variant 1, mRNA [NM_001008392] | 0,4437 | 7,01E-04 | 7,61E-03 |
| PACSIN1 | NM_020804 | Homo sapiens protein kinase C and casein kinase substrate in neurons 1 (PACSIN1), mRNA [NM_020804] | 0,8709 | 7,01E-04 | 7,61E-03 |
| DIAPH3 | NM_001042517 | Homo sapiens diaphanous homolog 3 (Drosophila) (DIAPH3), transcript variant 1, mRNA [NM_001042517] | 0,7098 | 7,04E-04 | 7,63E-03 |
| TCEB3 | ENST00000374536 | Transcription elongation factor B polypeptide 3 (RNA polymerase II transcription factor SIII subunit A1) (SIII p110) (Elongin-A) (EloA) (Elongin 110 kDa subunit). [Source:Uniprot/SWISSPROT;Acc:Q14241] [ENST00000374536] | 0,5966 | 7,04E-04 | 7,63E-03 |
| C19orf43 | NM_024038 | Homo sapiens chromosome 19 open reading frame 43 (C19orf43), mRNA [NM_024038] | 0,3897 | 7,05E-04 | 7,63E-03 |
| PRPS1L1 | NM_175886 | Homo sapiens phosphoribosyl pyrophosphate synthetase 1-like 1 (PRPS1L1), mRNA [NM_175886] | 0,6602 | 7,19E-04 | 7,76E-03 |
| MGC4677 | BC010491 | Homo sapiens hypothetical protein MGC4677, mRNA (cDNA clone MGC:17431 IMAGE:2984883), complete cds. [BC010491] | 0,5599 | 7,20E-04 | 7,77E-03 |
| APOL3 | NM_145641 | Homo sapiens apolipoprotein L, 3 (APOL3), transcript variant beta/a, mRNA [NM_145641] | 0,7213 | 7,32E-04 | 7,87E-03 |
| PITPNA | NM_006224 | Homo sapiens phosphatidylinositol transfer protein, alpha (PITPNA), mRNA [NM_006224] | 0,6210 | 7,32E-04 | 7,87E-03 |
| LYCAT | NM_182551 | Homo sapiens lysocardiolipin acyltransferase (LYCAT), transcript variant 1, mRNA [NM_182551] | 0,8076 | 7,33E-04 | 7,88E-03 |
| MBNL2 | NM_144778 | Homo sapiens muscleblind-like 2 (Drosophila) (MBNL2), transcript variant 1, mRNA [NM_144778] | 0,7029 | 7,36E-04 | 7,90E-03 |
| MGC13057 | NM_001042519 | Homo sapiens hypothetical protein MGC13057 (MGC13057), transcript variant 1, mRNA [NM_001042519] | 0,8644 | 7,39E-04 | 7,93E-03 |
| NF2 | NM_181831 | Homo sapiens neurofibromin 2 (bilateral acoustic neuroma) (NF2), transcript variant 13, mRNA [NM_181831] | 0,5364 | 7,40E-04 | 7,93E-03 |
| CCNDBP1 | NM_037370 | Homo sapiens cyclin D-type binding-protein 1 (CCNDBP1), transcript variant 2, mRNA [NM_037370] | 0,8070 | 7,49E-04 | 8,02E-03 |
| HLA-DQA2 | NM_020056 | Homo sapiens major histocompatibility complex, class II, DQ alpha 2 (HLA-DQA2), mRNA [NM_020056] | 1,2638 | 7,50E-04 | 8,02E-03 |
| PEPD | NM_000285 | Homo sapiens peptidase D (PEPD), mRNA [NM_000285] | 0,6916 | 7,55E-04 | 8,05E-03 |
| GMEB1 | ENST00000373816 | Glucocorticoid modulatory element-binding protein 1 (GMEB-1) (Parvovirus initiation factor p96) (PIF p96) (DNA-binding protein p96PIF). [Source:Uniprot/SWISSPROT;Acc:Q9Y692] [ENST00000373816] | 0,6717 | 7,56E-04 | 8,06E-03 |
| SLC25A11 | NM_003562 | Homo sapiens solute carrier family 25 (mitochondrial carrier; oxoglutarate carrier), member 11 (SLC25A11), mRNA [NM_003562] | 0,5980 | 7,61E-04 | 8,11E-03 |
| ZNF268 | X78926 | H.sapiens HZF3 mRNA for zinc finger protein. [X78926] | 0,7002 | 7,67E-04 | 8,16E-03 |
| THAP11 | NM_020457 | Homo sapiens THAP domain containing 11 (THAP11), mRNA [NM_020457] | 0,7736 | 7,69E-04 | 8,17E-03 |
| BM455859 | BM455859 | AGENCOURT_6409185 NIH_MGC_85 Homo sapiens cDNA clone IMAGE:5498310 5', mRNA sequence [BM455859] | 0,5258 | 7,72E-04 | 8,19E-03 |
| GEMIN4 | NM_015721 | Homo sapiens gem (nuclear organelle) associated protein 4 (GEMIN4), mRNA [NM_015721] | 0,8324 | 7,74E-04 | 8,22E-03 |
| CCDC92 | NM_025140 | Homo sapiens coiled-coil domain containing 92 (CCDC92), mRNA [NM_025140] | 0,5855 | 7,78E-04 | 8,25E-03 |
| FOXO3A | NM_001455 | Homo sapiens forkhead box O3A (FOXO3A), transcript variant 1, mRNA [NM_001455] | 1,2041 | 7,80E-04 | 8,27E-03 |
| USP31 | AB033029 | Homo sapiens mRNA for KIAA1203 protein, partial cds. [AB033029] | 0,5139 | 7,83E-04 | 8,29E-03 |
| TMEM39A | NM_018266 | Homo sapiens transmembrane protein 39A (TMEM39A), mRNA [NM_018266] | 0,6862 | 7,85E-04 | 8,30E-03 |
| THC2500271 | THC2500271 | Unknown | 0,3787 | 7,86E-04 | 8,30E-03 |
| AK095583 | AK095583 | Homo sapiens cDNA FLJ38264 fis, clone FCBBF3001657. [AK095583] | 0,7555 | 7,96E-04 | 8,39E-03 |
| DTL | NM_016448 | Homo sapiens denticleless homolog (Drosophila) (DTL), mRNA [NM_016448] | 0,5821 | 7,98E-04 | 8,41E-03 |
| YWHAG | NM_012479 | Homo sapiens tyrosine 3-monooxygenase/tryptophan 5-monooxygenase activation protein, gamma polypeptide (YWHAG), mRNA [NM_012479] | 0,7062 | 7,99E-04 | 8,42E-03 |
| NAP1L5 | NM_153757 | Homo sapiens nucleosome assembly protein 1-like 5 (NAP1L5), mRNA [NM_153757] | 0,7086 | 8,02E-04 | 8,45E-03 |
| DUSP14 | NM_007026 | Homo sapiens dual specificity phosphatase 14 (DUSP14), mRNA [NM_007026] | 0,4641 | 8,06E-04 | 8,48E-03 |
| COASY | NM_025233 | Homo sapiens Coenzyme A synthase (COASY), transcript variant 1, mRNA [NM_025233] | 0,7059 | 8,08E-04 | 8,49E-03 |
| BU732811 | BU732811 | BU732811 UI-E-CQ1-afz-j-16-0-UI.s1 UI-E-CQ1 Homo sapiens cDNA clone UI-E-CQ1-afz-j-16-0-UI 3', mRNA sequence [BU732811] | 0,6415 | 8,09E-04 | 8,50E-03 |
| SLC12A7 | NM_006598 | Homo sapiens solute carrier family 12 (potassium/chloride transporters), member 7 (SLC12A7), mRNA [NM_006598] | 0,5593 | 8,12E-04 | 8,52E-03 |
| CBX7 | NM_175709 | Homo sapiens chromobox homolog 7 (CBX7), mRNA [NM_175709] | 0,7404 | 8,14E-04 | 8,53E-03 |
| PAICS | NM_001079525 | Homo sapiens phosphoribosylaminoimidazole carboxylase, phosphoribosylaminoimidazole succinocarboxamide synthetase (PAICS), transcript variant 1, mRNA [NM_001079525] | 0,6634 | 8,14E-04 | 8,53E-03 |
| SEPP1 | NM_005410 | Homo sapiens selenoprotein P, plasma, 1 (SEPP1), mRNA [NM_005410] | 1,0643 | 8,21E-04 | 8,59E-03 |
| ARVCF | NM_001670 | Homo sapiens armadillo repeat gene deletes in velocardiofacial syndrome (ARVCF), mRNA [NM_001670] | 0,5132 | 8,22E-04 | 8,59E-03 |
| ZNF134 | NM_003435 | Homo sapiens zinc finger protein 134 (ZNF134), mRNA [NM_003435] | 0,6144 | 8,22E-04 | 8,59E-03 |
| POLE3 | NM_017443 | Homo sapiens polymerase (DNA directed), epsilon 3 (p17 subunit) (POLE3), mRNA [NM_017443] | 0,7215 | 8,24E-04 | 8,61E-03 |
| C2orf24 | NM_015680 | Homo sapiens chromosome 2 open reading frame 24 (C2orf24), mRNA [NM_015680] | 0,7943 | 8,25E-04 | 8,62E-03 |
| TRIP6 | NM_003302 | Homo sapiens thyroid hormone receptor interactor 6 (TRIP6), mRNA [NM_003302] | 0,6507 | 8,31E-04 | 8,66E-03 |
| FZR1 | NM_016263 | Homo sapiens fizzy/cell division cycle 20 related 1 (Drosophila) (FZR1), mRNA [NM_016263] | 0,7182 | 8,35E-04 | 8,71E-03 |
| ZBTB32 | NM_014383 | Homo sapiens zinc finger and BTB domain containing 32 (ZBTB32), mRNA [NM_014383] | 0,8339 | 8,36E-04 | 8,71E-03 |
| APOM | NM_019101 | Homo sapiens apolipoprotein M (APOM), mRNA [NM_019101] | 0,4996 | 8,38E-04 | 8,73E-03 |
| MRPS2 | NM_016034 | Homo sapiens mitochondrial ribosomal protein S2 (MRPS2), nuclear gene encoding mitochondrial protein, mRNA [NM_016034] | 0,6246 | 8,38E-04 | 8,73E-03 |
| AK125299 | AK125299 | Homo sapiens cDNA FLJ43309 fis, clone NT2RI2004618, highly similar to Cytosolic acyl coenzyme A thioester hydrolase (EC 3.1.2.2). [AK125299] | 0,4341 | 8,43E-04 | 8,76E-03 |
| SLC39A8 | NM_022154 | Homo sapiens solute carrier family 39 (zinc transporter), member 8 (SLC39A8), mRNA [NM_022154] | 0,7913 | 8,44E-04 | 8,76E-03 |
| TUSC1 | NM_001004125 | Homo sapiens tumor suppressor candidate 1 (TUSC1), mRNA [NM_001004125] | 0,5673 | 8,44E-04 | 8,76E-03 |
| CCDC86 | NM_024098 | Homo sapiens coiled-coil domain containing 86 (CCDC86), mRNA [NM_024098] | 0,6276 | 8,48E-04 | 8,80E-03 |
| AK095167 | AK095167 | Homo sapiens cDNA FLJ37848 fis, clone BRSSN2013544. [AK095167] | 0,7473 | 8,52E-04 | 8,82E-03 |
| ARL2BP | NM_012106 | Homo sapiens ADP-ribosylation factor-like 2 binding protein (ARL2BP), mRNA [NM_012106] | 0,8189 | 8,54E-04 | 8,83E-03 |
| NMNAT3 | NM_178177 | Homo sapiens nicotinamide nucleotide adenylyltransferase 3 (NMNAT3), mRNA [NM_178177] | 0,8763 | 8,53E-04 | 8,83E-03 |
| PET112L | NM_004564 | Homo sapiens PET112-like (yeast) (PET112L), mRNA [NM_004564] | 0,6426 | 8,54E-04 | 8,83E-03 |
| FAM33A | NM_182620 | Homo sapiens family with sequence similarity 33, member A (FAM33A), mRNA [NM_182620] | 0,5785 | 8,57E-04 | 8,85E-03 |
| SSBP3 | NM_001009955 | Homo sapiens single stranded DNA binding protein 3 (SSBP3), transcript variant 3, mRNA [NM_001009955] | 0,5997 | 8,57E-04 | 8,85E-03 |
| ZDHHC14 | NM_153746 | Homo sapiens zinc finger, DHHC-type containing 14 (ZDHHC14), transcript variant 2, mRNA [NM_153746] | 0,7051 | 8,59E-04 | 8,86E-03 |
| SEPHS1 | NM_012247 | Homo sapiens selenophosphate synthetase 1 (SEPHS1), mRNA [NM_012247] | 0,5873 | 8,59E-04 | 8,86E-03 |
| SLBP | NM_006527 | Homo sapiens stem-loop (histone) binding protein (SLBP), mRNA [NM_006527] | 0,6140 | 8,66E-04 | 8,91E-03 |
| AP3D1 | NM_003938 | Homo sapiens adaptor-related protein complex 3, delta 1 subunit (AP3D1), transcript variant 2, mRNA [NM_003938] | 0,7600 | 8,70E-04 | 8,94E-03 |
| RNMTL1 | NM_018146 | Homo sapiens RNA methyltransferase like 1 (RNMTL1), mRNA [NM_018146] | 0,5252 | 8,70E-04 | 8,94E-03 |
| TH1L | NM_198976 | Homo sapiens TH1-like (Drosophila) (TH1L), transcript variant 1, mRNA [NM_198976] | 0,5118 | 8,73E-04 | 8,97E-03 |
| FAM100A | NM_145253 | Homo sapiens family with sequence similarity 100, member A (FAM100A), mRNA [NM_145253] | 0,8131 | 8,78E-04 | 9,01E-03 |
| MGC15523 | NM_138570 | Homo sapiens hypothetical protein MGC15523 (MGC15523), transcript variant 2, mRNA [NM_138570] | 0,4989 | 8,78E-04 | 9,01E-03 |
| BC013077 | BC013077 | Homo sapiens, clone IMAGE:3459334, mRNA. [BC013077] | 0,3581 | 8,79E-04 | 9,01E-03 |
| ZNF364 | ENST00000369291 | Zinc finger protein 364 (Rabring 7) (RING finger protein 115). [Source:Uniprot/SWISSPROT;Acc:Q9Y4L5] [ENST00000369291] | 0,5634 | 8,80E-04 | 9,01E-03 |
| KCNH2 | NM_000238 | Homo sapiens potassium voltage-gated channel, subfamily H (eag-related), member 2 (KCNH2), transcript variant 1, mRNA [NM_000238] | 1,0677 | 8,80E-04 | 9,01E-03 |
| EEF2K | NM_013302 | Homo sapiens eukaryotic elongation factor-2 kinase (EEF2K), mRNA [NM_013302] | 0,5731 | 8,84E-04 | 9,04E-03 |
| AK093617 | AK093617 | Homo sapiens cDNA FLJ36298 fis, clone THYMU2004344. [AK093617] | 1,0613 | 8,86E-04 | 9,06E-03 |
| C22orf28 | NM_014306 | Homo sapiens chromosome 22 open reading frame 28 (C22orf28), mRNA [NM_014306] | 0,6355 | 8,90E-04 | 9,08E-03 |
| EI24 | NM_004879 | Homo sapiens etoposide induced 2.4 mRNA (EI24), transcript variant 1, mRNA [NM_004879] | 0,6889 | 8,91E-04 | 9,08E-03 |
| PES1 | NM_014303 | Homo sapiens pescadillo homolog 1, containing BRCT domain (zebrafish) (PES1), mRNA [NM_014303] | 0,6744 | 8,93E-04 | 9,09E-03 |
| FANCC | NM_000136 | Homo sapiens Fanconi anemia, complementation group C (FANCC), mRNA [NM_000136] | 0,5986 | 8,95E-04 | 9,10E-03 |
| ATP7B | NM_000053 | Homo sapiens ATPase, Cu++ transporting, beta polypeptide (ATP7B), transcript variant 1, mRNA [NM_000053] | 0,8842 | 8,96E-04 | 9,11E-03 |
| RBM15B | NM_013286 | Homo sapiens RNA binding motif protein 15B (RBM15B), mRNA [NM_013286] | 0,7811 | 8,97E-04 | 9,11E-03 |
| RNPS1 | NM_006711 | Homo sapiens RNA binding protein S1, serine-rich domain (RNPS1), transcript variant 1, mRNA [NM_006711] | 0,8475 | 9,02E-04 | 9,16E-03 |
| STAU1 | NM_017453 | Homo sapiens staufen, RNA binding protein, homolog 1 (Drosophila) (STAU1), transcript variant T3, mRNA [NM_017453] | 0,6937 | 9,11E-04 | 9,23E-03 |
| KIAA0133 | NM_014777 | Homo sapiens KIAA0133 (KIAA0133), mRNA [NM_014777] | 0,5676 | 9,12E-04 | 9,25E-03 |
| ZSCAN5 | NM_024303 | Homo sapiens zinc finger and SCAN domain containing 5 (ZSCAN5), mRNA [NM_024303] | 0,6316 | 9,19E-04 | 9,30E-03 |
| RPS3 | NM_001005 | Homo sapiens ribosomal protein S3 (RPS3), mRNA [NM_001005] | 0,5686 | 9,20E-04 | 9,31E-03 |
| ZNF689 | NM_138447 | Homo sapiens zinc finger protein 689 (ZNF689), mRNA [NM_138447] | 0,7869 | 9,31E-04 | 9,39E-03 |
| SRM | NM_003132 | Homo sapiens spermidine synthase (SRM), mRNA [NM_003132] | 0,6437 | 9,39E-04 | 9,47E-03 |
| CHAC2 | NM_001008708 | Homo sapiens ChaC, cation transport regulator homolog 2 (E. coli) (CHAC2), mRNA [NM_001008708] | 0,9831 | 9,40E-04 | 9,47E-03 |
| FTSJ2 | NM_013393 | Homo sapiens FtsJ homolog 2 (E. coli) (FTSJ2), mRNA [NM_013393] | 0,5476 | 9,44E-04 | 9,51E-03 |
| SRD5A2L | NM_024592 | Homo sapiens steroid 5 alpha-reductase 2-like (SRD5A2L), mRNA [NM_024592] | 0,7474 | 9,44E-04 | 9,51E-03 |
| PSMC4 | NM_006503 | Homo sapiens proteasome (prosome, macropain) 26S subunit, ATPase, 4 (PSMC4), transcript variant 1, mRNA [NM_006503] | 0,4501 | 9,52E-04 | 9,57E-03 |
| ENST00000358618 | ENST00000358618 | PREDICTED: Homo sapiens similar to ribosomal protein S2 (LOC729842), mRNA [XM_001134158] | 0,7714 | 9,64E-04 | 9,66E-03 |
| ZAK | NM_133646 | Homo sapiens sterile alpha motif and leucine zipper containing kinase AZK (ZAK), transcript variant 2, mRNA [NM_133646] | 0,6391 | 9,66E-04 | 9,67E-03 |
| AK124299 | AK124299 | Homo sapiens cDNA FLJ42306 fis, clone TRACH2001646. [AK124299] | 0,6946 | 9,67E-04 | 9,68E-03 |
| CKAP5 | NM_001008938 | Homo sapiens cytoskeleton associated protein 5 (CKAP5), transcript variant 1, mRNA [NM_001008938] | 0,5474 | 9,69E-04 | 9,68E-03 |
| EIF5A2 | NM_020390 | Homo sapiens eukaryotic translation initiation factor 5A2 (EIF5A2), mRNA [NM_020390] | 0,6029 | 9,69E-04 | 9,68E-03 |
| KIAA1815 | NM_024896 | Homo sapiens KIAA1815 (KIAA1815), mRNA [NM_024896] | 0,4483 | 9,68E-04 | 9,68E-03 |
| PHOSPHO2 | NM_001008489 | Homo sapiens phosphatase, orphan 2 (PHOSPHO2), mRNA [NM_001008489] | 0,5611 | 9,69E-04 | 9,68E-03 |
| KIAA0652 | NM_014741 | Homo sapiens KIAA0652 (KIAA0652), mRNA [NM_014741] | 0,7662 | 9,78E-04 | 9,75E-03 |
| QDPR | NM_000320 | Homo sapiens quinoid dihydropteridine reductase (QDPR), mRNA [NM_000320] | 0,5387 | 9,86E-04 | 9,82E-03 |
| ACAA2 | NM_006111 | Homo sapiens acetyl-Coenzyme A acyltransferase 2 (mitochondrial 3-oxoacyl-Coenzyme A thiolase) (ACAA2), nuclear gene encoding mitochondrial protein, mRNA [NM_006111] | 0,6055 | 9,95E-04 | 9,90E-03 |
| CCT2 | NM_006431 | Homo sapiens chaperonin containing TCP1, subunit 2 (beta) (CCT2), mRNA [NM_006431] | 0,6540 | 9,95E-04 | 9,90E-03 |
| EGFL8 | NM_030652 | Homo sapiens EGF-like-domain, multiple 8 (EGFL8), mRNA [NM_030652] | 0,9961 | 1,00E-03 | 9,93E-03 |
| AK095707 | AK095707 | Homo sapiens cDNA FLJ38388 fis, clone FEBRA2004485. [AK095707] | 0,4166 | 1,00E-03 | 9,97E-03 |
| MIS12 | NM_024039 | Homo sapiens MIS12, MIND kinetochore complex component, homolog (yeast) (MIS12), mRNA [NM_024039] | 0,5696 | 1,01E-03 | 9,99E-03 |
| PPME1 | NM_016147 | Homo sapiens protein phosphatase methylesterase 1 (PPME1), mRNA [NM_016147] | 0,6240 | 1,01E-03 | 9,99E-03 |

**Supplementary Table 1: C)** Genes under-expressed in BM resident cells from patients with metastatic NB as compared to patients with localized NB (Bonferroni’s adjusted P value was not significant).

| **Gene name** | **Systematic name** | **Description** | **Log fold Change** | **P value** |
| --- | --- | --- | --- | --- |
| CXCL12 | NM_199168 | Homo sapiens chemokine (C-X-C motif) ligand 12 (stromal cell-derived factor 1) (CXCL12), transcript variant 1, mRNA [NM_199168] | 1,4122 | 7,48E-06 |
| PF4 | NM_002619 | Homo sapiens platelet factor 4 (chemokine (C-X-C motif) ligand 4) (PF4), mRNA [NM_002619] | 2,4356 | 7,33E-05 |
| W60781 | W60781 | W60781 zd26f05.r1 Soares_fetal_heart_NbHH19W Homo sapiens cDNA clone IMAGE:341793 5' similar to gb:J02874 FATTY ACID-BINDING PROTEIN, ADIPOCYTE (HUMAN);, mRNA sequence [W60781] | 2,0030 | 9,12E-05 |
| AK126431 | AK126431 | Homo sapiens cDNA FLJ44467 fis, clone UTERU2025891. [AK126431] | 1,0559 | 1,28E-04 |
| TF | NM_001063 | Homo sapiens transferrin (TF), mRNA [NM_001063] | 1,5288 | 1,34E-04 |
| PPBP | NM_002704 | Homo sapiens pro-platelet basic protein (chemokine (C-X-C motif) ligand 7) (PPBP), mRNA [NM_002704] | 2,1980 | 4,48E-04 |
| GNAZ | NM_002073 | Homo sapiens guanine nucleotide binding protein (G protein), alpha z polypeptide (GNAZ), mRNA [NM_002073] | 1,5263 | 5,13E-04 |
| CLEC1B | NM_016509 | Homo sapiens C-type lectin domain family 1, member B (CLEC1B), mRNA [NM_016509] | 0,9878 | 9,48E-04 |
| ENST00000382909 | ENST00000382909 | Zinc finger MYM-type protein 5 (Zinc finger protein 237) (Zinc finger protein 198-like 1). [Source:Uniprot/SWISSPROT;Acc:Q9UJ78] [ENST00000382909] | 0,6141 | 1,10E-03 |
| TIMD4 | NM_138379 | Homo sapiens T-cell immunoglobulin and mucin domain containing 4 (TIMD4), mRNA [NM_138379] | 1,0594 | 1,31E-03 |
| TUBA8 | NM_018943 | Homo sapiens tubulin, alpha 8 (TUBA8), mRNA [NM_018943] | 1,1205 | 1,32E-03 |
| SEPP1 | NM_005410 | Homo sapiens selenoprotein P, plasma, 1 (SEPP1), mRNA [NM_005410] | 1,0435 | 1,52E-03 |
| EPN2 | NM_014964 | Homo sapiens epsin 2 (EPN2), transcript variant 2, mRNA [NM_014964] | 0,7545 | 1,72E-03 |
| GP1BA | J02940 | Human platelet glycoprotein Ib alpha chain mRNA, complete cds. [J02940] | 1,1056 | 2,10E-03 |
| CMTM5 | NM_001037288 | Homo sapiens CKLF-like MARVEL transmembrane domain containing 5 (CMTM5), transcript variant 3, mRNA [NM_001037288] | 1,2181 | 2,32E-03 |
| C6orf25 | ENST00000375806 | G6b protein precursor. [Source:Uniprot/SWISSPROT;Acc:O95866] [ENST00000375806] | 0,6601 | 4,30E-03 |
| CLDN5 | NM_003277 | Homo sapiens claudin 5 (transmembrane protein deleted in velocardiofacial syndrome) (CLDN5), mRNA [NM_003277] | 0,9518 | 4,30E-03 |
| ZNF182 | NM_006962 | Homo sapiens zinc finger protein 182 (ZNF182), transcript variant 1, mRNA [NM_006962] | 0,9789 | 4,38E-03 |
| CMTM7 | AL832450 | Homo sapiens mRNA; cDNA DKFZp434I2129 (from clone DKFZp434I2129). [AL832450] | 1,0914 | 4,44E-03 |
| CROP | NM_016424 | Homo sapiens cisplatin resistance-associated overexpressed protein (CROP), transcript variant 1, mRNA [NM_016424] | 0,7433 | 4,60E-03 |
| THC2682560 | THC2682560 | Unknown | 0,6419 | 5,89E-03 |
| THC2539273 | THC2539273 | Unknown | 0,7592 | 5,94E-03 |
| CIRBP | AK128423 | Homo sapiens cDNA FLJ46566 fis, clone THYMU3040829, moderately similar to Cold-inducible RNA-binding protein. [AK128423] | 0,6734 | 5,94E-03 |
| DIDO1 | NM_080797 | Homo sapiens death inducer-obliterator 1 (DIDO1), transcript variant 3, mRNA [NM_080797] | 0,7377 | 6,14E-03 |
| SFRS1 | NM_001078166 | Homo sapiens splicing factor, arginine/serine-rich 1 (splicing factor 2, alternate splicing factor) (SFRS1), transcript variant 2, mRNA [NM_001078166] | 0,5961 | 6,97E-03 |
| GIT2 | NM_139201 | Homo sapiens G protein-coupled receptor kinase interactor 2 (GIT2), transcript variant 4, mRNA [NM_139201] | 0,7021 | 7,39E-03 |
| RBM5 | NM_005778 | Homo sapiens RNA binding motif protein 5 (RBM5), mRNA [NM_005778] | 0,7252 | 8,07E-03 |
| TAF1 | ENST00000373790 | Transcription initiation factor TFIID subunit 1 (EC 2.7.11.1) (Transcription initiation factor TFIID 250 kDa subunit) (TAF(II)250) (TAFII-250) (TAFII250) (TBP-associated factor 250 kDa) (p250) (Cell cycle gene 1 protein).... | 0,6428 | 9,28E-03 |
| IGF2 | NM_000612 | Homo sapiens insulin-like growth factor 2 (somatomedin A) (IGF2), transcript variant 1, mRNA [NM_000612] | 1,4915 | 9,60E-03 |

**Supplementary Table 1: D)** Genes significantly (Bonferroni’s adjusted P value < 0.01) under-expressed in BM resident cells from patients with either metastatic or localized NB as compared to healthy children.

| **Gene name** | **Systematic name** | **Description** | **Log fold change** | **P value** | **Adjusted P value** |
| --- | --- | --- | --- | --- | --- |
| TNS1 | NM_022648 | Homo sapiens tensin 1 (TNS1), mRNA [NM_022648] | -3,0348 | 2,64E-16 | 2,47E-12 |
| ENST00000374390 | ENST00000374390 | cellular modulator of immune recognition isoform 7 [Source:RefSeq_peptide;Acc:NP_001002266] [ENST00000374390] | -2,4471 | 6,16E-16 | 2,80E-12 |
| SPTB | NM_000347 | Homo sapiens spectrin, beta, erythrocytic (includes spherocytosis, clinical type I) (SPTB), transcript variant 2, mRNA [NM_000347] | -2,6641 | 7,49E-16 | 2,80E-12 |
| THC2613527 | THC2613527 | Unknown | -2,0309 | 1,10E-15 | 3,42E-12 |
| SLC6A10P | NR_003083 | Homo sapiens solute carrier family 6 (neurotransmitter transporter, creatine), member 10 (pseudogene) (SLC6A10P) on chromosome 16 [NR_003083] | -2,3794 | 1,58E-15 | 4,23E-12 |
| SLC4A1 | NM_000342 | Homo sapiens solute carrier family 4, anion exchanger, member 1 (erythrocyte membrane protein band 3, Diego blood group) (SLC4A1), mRNA [NM_000342] | -2,9000 | 6,01E-15 | 1,25E-11 |
| BX537432 | BX537432 | Homo sapiens mRNA; cDNA DKFZp686N09198 (from clone DKFZp686N09198); complete cds. [BX537432] | -3,1793 | 1,29E-14 | 1,95E-11 |
| FAM46C | NM_017709 | Homo sapiens family with sequence similarity 46, member C (FAM46C), mRNA [NM_017709] | -2,3703 | 1,35E-14 | 1,95E-11 |
| PDZK1IP1 | NM_005764 | Homo sapiens PDZK1 interacting protein 1 (PDZK1IP1), mRNA [NM_005764] | -3,3778 | 1,46E-14 | 1,95E-11 |
| PLEK2 | NM_016445 | Homo sapiens pleckstrin 2 (PLEK2), mRNA [NM_016445] | -2,7179 | 1,38E-14 | 1,95E-11 |
| SLC6A8 | NM_005629 | Homo sapiens solute carrier family 6 (neurotransmitter transporter, creatine), member 8 (SLC6A8), mRNA [NM_005629] | -2,1894 | 3,09E-14 | 3,85E-11 |
| TSPAN5 | AK055659 | Homo sapiens cDNA FLJ31097 fis, clone IMR321000210. [AK055659] | -3,5672 | 4,77E-14 | 5,58E-11 |
| GPR146 | NM_138445 | Homo sapiens G protein-coupled receptor 146 (GPR146), mRNA [NM_138445] | -1,8312 | 6,70E-14 | 7,38E-11 |
| A_32_P78285 | A_32_P78285 | Unknown | -2,6162 | 7,81E-14 | 8,13E-11 |
| ENST00000371189 | ENST00000371189 | Nuclear factor 1 A-type (Nuclear factor 1/A) (NF1-A) (NFI-A) (NF-I/A) (CCAAT-box-binding transcription factor) (CTF) (TGGCA-binding protein). [Source:Uniprot/SWISSPROT;Acc:Q12857] [ENST00000371189] | -1,8408 | 1,06E-13 | 9,83E-11 |
| SLC14A1 | ENST00000321925 | Urea transporter, erythrocyte. [Source:Uniprot/SWISSPROT;Acc:Q13336] [ENST00000321925] | -3,0106 | 1,10E-13 | 9,83E-11 |
| TMCC2 | NM_014858 | Homo sapiens transmembrane and coiled-coil domain family 2 (TMCC2), mRNA [NM_014858] | -4,3570 | 1,60E-13 | 1,36E-10 |
| PAQR9 | NM_198504 | Homo sapiens progestin and adipoQ receptor family member IX (PAQR9), mRNA [NM_198504] | -2,4999 | 1,89E-13 | 1,54E-10 |
| FECH | NM_001012515 | Homo sapiens ferrochelatase (protoporphyria) (FECH), nuclear gene encoding mitochondrial protein, transcript variant 1, mRNA [NM_001012515] | -3,3515 | 2,23E-13 | 1,74E-10 |
| TRAK2 | NM_015049 | Homo sapiens trafficking protein, kinesin binding 2 (TRAK2), mRNA [NM_015049] | -2,0387 | 2,79E-13 | 2,09E-10 |
| SLC25A39 | NM_016016 | Homo sapiens solute carrier family 25, member 39 (SLC25A39), mRNA [NM_016016] | -2,9547 | 3,75E-13 | 2,70E-10 |
| SNCA | NM_007308 | Homo sapiens synuclein, alpha (non A4 component of amyloid precursor) (SNCA), transcript variant NACP112, mRNA [NM_007308] | -4,0324 | 4,78E-13 | 3,18E-10 |
| TBCEL | BC020501 | Homo sapiens leucine rich repeat containing 35, mRNA (cDNA clone IMAGE:3913004). [BC020501] | -1,7503 | 4,93E-13 | 3,18E-10 |
| THC2669092 | THC2669092 | Unknown | -3,6104 | 4,89E-13 | 3,18E-10 |
| AQP1 | NM_198098 | Homo sapiens aquaporin 1 (Colton blood group) (AQP1), mRNA [NM_198098] | -3,7765 | 7,45E-13 | 4,10E-10 |
| FLJ41603 | NM_001001669 | Homo sapiens FLJ41603 protein (FLJ41603), mRNA [NM_001001669] | -1,8714 | 7,15E-13 | 4,10E-10 |
| ACSL6 | NM_001009185 | Homo sapiens acyl-CoA synthetase long-chain family member 6 (ACSL6), transcript variant 2, mRNA [NM_001009185] | -2,7526 | 8,95E-13 | 4,78E-10 |
| HAGH | NM_001040427 | Homo sapiens hydroxyacylglutathione hydrolase (HAGH), transcript variant 2, mRNA [NM_001040427] | -1,9912 | 1,04E-12 | 5,42E-10 |
| MICAL2 | NM_014632 | Homo sapiens microtubule associated monoxygenase, calponin and LIM domain containing 2 (MICAL2), mRNA [NM_014632] | -2,7862 | 1,24E-12 | 6,30E-10 |
| THC2654231 | THC2654231 | ALU5_HUMAN (P39192) Alu subfamily SC sequence contamination warning entry, partial (8%) [THC2654231] | -1,9036 | 1,34E-12 | 6,60E-10 |
| GMPR | NM_006877 | Homo sapiens guanosine monophosphate reductase (GMPR), mRNA [NM_006877] | -2,5618 | 1,56E-12 | 7,50E-10 |
| KRT1 | NM_006121 | Homo sapiens keratin 1 (epidermolytic hyperkeratosis) (KRT1), mRNA [NM_006121] | -4,2709 | 1,67E-12 | 7,60E-10 |
| WDR40A | NM_015397 | Homo sapiens WD repeat domain 40A (WDR40A), mRNA [NM_015397] | -2,3478 | 1,64E-12 | 7,60E-10 |
| THC2656519 | THC2656519 | Unknown | -1,6794 | 1,76E-12 | 7,83E-10 |
| THC2588392 | THC2588392 | 1ABW_A Chain A, Deoxy Rhb1.1 (Recombinant Hemoglobin). {synthetic construct} (exp=-1; wgp=-1; cg=-1), partial (18%) [THC2588392] | -4,2552 | 1,97E-12 | 8,59E-10 |
| GYPB | NM_002100 | Homo sapiens glycophorin B (MNS blood group) (GYPB), mRNA [NM_002100] | -2,9260 | 2,14E-12 | 9,09E-10 |
| THC2633438 | THC2633438 | ALU5_HUMAN (P39192) Alu subfamily SC sequence contamination warning entry, partial (9%) [THC2633438] | -2,0461 | 2,79E-12 | 1,16E-09 |
| C5orf4 | NM_032385 | Homo sapiens chromosome 5 open reading frame 4 (C5orf4), transcript variant 2, mRNA [NM_032385] | -3,0174 | 3,47E-12 | 1,41E-09 |
| RHCE | NM_020485 | Homo sapiens Rh blood group, CcEe antigens (RHCE), transcript variant 1, mRNA [NM_020485] | -3,1877 | 3,77E-12 | 1,50E-09 |
| MICALCL | NM_032867 | Homo sapiens MICAL C-terminal like (MICALCL), mRNA [NM_032867] | -1,5256 | 4,05E-12 | 1,58E-09 |
| NFIX | NM_002501 | Homo sapiens nuclear factor I/X (CCAAT-binding transcription factor) (NFIX), mRNA [NM_002501] | -2,6503 | 5,57E-12 | 2,12E-09 |
| SLC1A5 | NM_005628 | Homo sapiens solute carrier family 1 (neutral amino acid transporter), member 5 (SLC1A5), mRNA [NM_005628] | -1,6491 | 8,81E-12 | 3,17E-09 |
| HEMGN | NM_018437 | Homo sapiens hemogen (HEMGN), transcript variant 1, mRNA [NM_018437] | -3,1085 | 9,50E-12 | 3,36E-09 |
| LOC644462 | XM_930312 | PREDICTED: Homo sapiens similar to amyotrophic lateral sclerosis 2 (juvenile) chromosome region, candidate 2 (LOC644462), mRNA [XM_930312] | -2,0788 | 1,00E-11 | 3,48E-09 |
| XPO7 | NM_015024 | Homo sapiens exportin 7 (XPO7), mRNA [NM_015024] | -1,6526 | 1,03E-11 | 3,50E-09 |
| EPB42 | NM_000119 | Homo sapiens erythrocyte membrane protein band 4.2 (EPB42), mRNA [NM_000119] | -3,4259 | 1,06E-11 | 3,54E-09 |
| KLF1 | NM_006563 | Homo sapiens Kruppel-like factor 1 (erythroid) (KLF1), mRNA [NM_006563] | -1,8487 | 1,13E-11 | 3,72E-09 |
| ALS2CR2 | NM_018571 | Homo sapiens amyotrophic lateral sclerosis 2 (juvenile) chromosome region, candidate 2 (ALS2CR2), mRNA [NM_018571] | -2,7829 | 1,18E-11 | 3,75E-09 |
| SLC2A1 | NM_006516 | Homo sapiens solute carrier family 2 (facilitated glucose transporter), member 1 (SLC2A1), mRNA [NM_006516] | -2,8539 | 1,18E-11 | 3,75E-09 |
| DPM2 | NM_003863 | Homo sapiens dolichyl-phosphate mannosyltransferase polypeptide 2, regulatory subunit (DPM2), mRNA [NM_003863] | -1,5026 | 1,28E-11 | 4,01E-09 |
| KIF26A | BC009415 | Homo sapiens kinesin family member 26A, mRNA (cDNA clone IMAGE:3502885), complete cds. [BC009415] | -1,8588 | 1,44E-11 | 4,33E-09 |
| PRDX2 | NM_181738 | Homo sapiens peroxiredoxin 2 (PRDX2), nuclear gene encoding mitochondrial protein, transcript variant 3, mRNA [NM_181738] | -2,0444 | 1,46E-11 | 4,33E-09 |
| BPGM | NM_199186 | Homo sapiens 2,3-bisphosphoglycerate mutase (BPGM), transcript variant 2, mRNA [NM_199186] | -3,5390 | 1,61E-11 | 4,70E-09 |
| BSG | NM_001728 | Homo sapiens basigin (Ok blood group) (BSG), transcript variant 1, mRNA [NM_001728] | -1,6889 | 1,68E-11 | 4,83E-09 |
| C20orf108 | NM_080821 | Homo sapiens chromosome 20 open reading frame 108 (C20orf108), mRNA [NM_080821] | -2,2301 | 1,90E-11 | 5,38E-09 |
| WDR23 | NM_025230 | Homo sapiens WD repeat domain 23 (WDR23), transcript variant 1, mRNA [NM_025230] | -1,6890 | 2,29E-11 | 6,40E-09 |
| A_24_P401150 | A_24_P401150 | Unknown | -2,1904 | 2,69E-11 | 7,40E-09 |
| ITLN1 | NM_017625 | Homo sapiens intelectin 1 (galactofuranose binding) (ITLN1), mRNA [NM_017625] | -2,2737 | 2,91E-11 | 7,88E-09 |
| THC2688497 | THC2688497 | Q59GX2_HUMAN (Q59GX2) Solute carrier family 2 (Facilitated glucose transporter), member 1 variant (Fragment), partial (6%) [THC2564899] | -2,3589 | 3,71E-11 | 9,92E-09 |
| FBXO7 | NM_012179 | Homo sapiens F-box protein 7 (FBXO7), transcript variant 1, mRNA [NM_012179] | -1,9229 | 4,36E-11 | 1,12E-08 |
| THC2478531 | THC2478531 | Unknown | -3,9338 | 5,75E-11 | 1,45E-08 |
| AQP3 | NM_004925 | Homo sapiens aquaporin 3 (Gill blood group) (AQP3), mRNA [NM_004925] | -2,3171 | 6,00E-11 | 1,48E-08 |
| GCLC | NM_001498 | Homo sapiens glutamate-cysteine ligase, catalytic subunit (GCLC), mRNA [NM_001498] | -2,2270 | 6,62E-11 | 1,61E-08 |
| PHLPPL | NM_015020 | Homo sapiens PH domain and leucine rich repeat protein phosphatase-like (PHLPPL), mRNA [NM_015020] | -1,4675 | 6,69E-11 | 1,61E-08 |
| FZD5 | NM_003468 | Homo sapiens frizzled homolog 5 (Drosophila) (FZD5), mRNA [NM_003468] | -1,6550 | 6,80E-11 | 1,61E-08 |
| ART4 | NM_021071 | Homo sapiens ADP-ribosyltransferase 4 (Dombrock blood group) (ART4), mRNA [NM_021071] | -2,0740 | 7,38E-11 | 1,73E-08 |
| ANKH | ENST00000382327 | Progressive ankylosis protein homolog (ANK). [Source:Uniprot/SWISSPROT;Acc:Q9HCJ1] [ENST00000382327] | -1,2062 | 7,60E-11 | 1,74E-08 |
| HBQ1 | NM_005331 | Homo sapiens hemoglobin, theta 1 (HBQ1), mRNA [NM_005331] | -4,0430 | 7,53E-11 | 1,74E-08 |
| A_24_P147849 | A_24_P147849 | Unknown | -5,0742 | 8,78E-11 | 1,92E-08 |
| KEL | NM_000420 | Homo sapiens Kell blood group, metallo-endopeptidase (KEL), mRNA [NM_000420] | -1,6176 | 8,81E-11 | 1,92E-08 |
| SELENBP1 | NM_003944 | Homo sapiens selenium binding protein 1 (SELENBP1), mRNA [NM_003944] | -3,9777 | 8,82E-11 | 1,92E-08 |
| ERMAP | NM_001017922 | Homo sapiens erythroblast membrane-associated protein (Scianna blood group) (ERMAP), transcript variant 1, mRNA [NM_001017922] | -1,7573 | 9,13E-11 | 1,96E-08 |
| OSBP2 | NM_030758 | Homo sapiens oxysterol binding protein 2 (OSBP2), transcript variant 1, mRNA [NM_030758] | -2,4754 | 1,00E-10 | 2,14E-08 |
| TNXB | NM_032470 | Homo sapiens tenascin XB (TNXB), transcript variant XB-S, mRNA [NM_032470] | -2,4572 | 1,10E-10 | 2,28E-08 |
| EFNB3 | NM_001406 | Homo sapiens ephrin-B3 (EFNB3), mRNA [NM_001406] | -4,1116 | 1,13E-10 | 2,31E-08 |
| ABCG2 | NM_004827 | Homo sapiens ATP-binding cassette, sub-family G (WHITE), member 2 (ABCG2), mRNA [NM_004827] | -1,7097 | 1,20E-10 | 2,41E-08 |
| C16orf35 | NM_001039476 | Homo sapiens chromosome 16 open reading frame 35 (C16orf35), transcript variant 2, mRNA [NM_001039476] | -2,0697 | 1,29E-10 | 2,57E-08 |
| RILP | NM_031430 | Homo sapiens Rab interacting lysosomal protein (RILP), mRNA [NM_031430] | -2,0923 | 1,32E-10 | 2,61E-08 |
| TTC25 | NM_031421 | Homo sapiens tetratricopeptide repeat domain 25 (TTC25), mRNA [NM_031421] | -2,5012 | 1,37E-10 | 2,64E-08 |
| AY358510 | AY358510 | Homo sapiens clone DNA57836 GLPG464 (UNQ464) mRNA, complete cds. [AY358510] | -1,3668 | 1,53E-10 | 2,90E-08 |
| XK | NM_021083 | Homo sapiens X-linked Kx blood group (McLeod syndrome) (XK), mRNA [NM_021083] | -2,5265 | 1,71E-10 | 3,21E-08 |
| ANK1 | NM_000037 | Homo sapiens ankyrin 1, erythrocytic (ANK1), transcript variant 3, mRNA [NM_000037] | -2,6934 | 1,86E-10 | 3,41E-08 |
| EIF2AK1 | NM_014413 | Homo sapiens eukaryotic translation initiation factor 2-alpha kinase 1 (EIF2AK1), mRNA [NM_014413] | -1,7276 | 1,99E-10 | 3,61E-08 |
| TRIM10 | NM_052828 | Homo sapiens tripartite motif-containing 10 (TRIM10), transcript variant 2, mRNA [NM_052828] | -3,3856 | 2,03E-10 | 3,66E-08 |
| HMBS | NM_000190 | Homo sapiens hydroxymethylbilane synthase (HMBS), transcript variant 1, mRNA [NM_000190] | -2,5494 | 2,27E-10 | 4,05E-08 |
| A_23_P84791 | A_23_P84791 | Unknown | -1,9364 | 2,33E-10 | 4,09E-08 |
| HBA2 | NM_000517 | Homo sapiens hemoglobin, alpha 2 (HBA2), mRNA [NM_000517] | -4,4727 | 2,34E-10 | 4,09E-08 |
| GATA1 | NM_002049 | Homo sapiens GATA binding protein 1 (globin transcription factor 1) (GATA1), mRNA [NM_002049] | -1,5493 | 2,52E-10 | 4,38E-08 |
| NT5M | NM_020201 | Homo sapiens 5',3'-nucleotidase, mitochondrial (NT5M), nuclear gene encoding mitochondrial protein, mRNA [NM_020201] | -1,2022 | 2,58E-10 | 4,44E-08 |
| ATP1B2 | NM_001678 | Homo sapiens ATPase, Na+/K+ transporting, beta 2 polypeptide (ATP1B2), mRNA [NM_001678] | -1,9364 | 3,06E-10 | 5,21E-08 |
| CTSE | NM_001910 | Homo sapiens cathepsin E (CTSE), transcript variant 1, mRNA [NM_001910] | -2,0257 | 3,13E-10 | 5,23E-08 |
| TGM2 | NM_198951 | Homo sapiens transglutaminase 2 (C polypeptide, protein-glutamine-gamma-glutamyltransferase) (TGM2), transcript variant 2, mRNA [NM_198951] | -1,8334 | 3,11E-10 | 5,23E-08 |
| IL8 | X77737 | H.sapiens mRNA for red cell anion exchanger (EPB3, AE1, Band 3) 3' non-coding region. [X77737] | -3,3396 | 3,32E-10 | 5,50E-08 |
| GYPC | NM_002101 | Homo sapiens glycophorin C (Gerbich blood group) (GYPC), transcript variant 1, mRNA [NM_002101] | -2,0214 | 3,41E-10 | 5,61E-08 |
| DHRS13 | NM_144683 | Homo sapiens dehydrogenase/reductase (SDR family) member 13 (DHRS13), mRNA [NM_144683] | -1,8026 | 3,74E-10 | 6,04E-08 |
| RBM38 | NM_017495 | Homo sapiens RNA binding motif protein 38 (RBM38), transcript variant 1, mRNA [NM_017495] | -1,8301 | 3,96E-10 | 6,27E-08 |
| ADD2 | NM_017488 | Homo sapiens adducin 2 (beta) (ADD2), transcript variant beta-4, mRNA [NM_017488] | -1,5176 | 4,07E-10 | 6,35E-08 |
| PRR5 | NM_015366 | Homo sapiens proline rich 5 (renal) (PRR5), transcript variant 2, mRNA [NM_015366] | -1,6077 | 4,16E-10 | 6,43E-08 |
| RHAG | NM_000324 | Homo sapiens Rh-associated glycoprotein (RHAG), mRNA [NM_000324] | -2,4954 | 4,34E-10 | 6,61E-08 |
| SEC14L4 | NM_174977 | Homo sapiens SEC14-like 4 (S. cerevisiae) (SEC14L4), mRNA [NM_174977] | -2,4731 | 4,41E-10 | 6,66E-08 |
| BCL2L1 | NM_138578 | Homo sapiens BCL2-like 1 (BCL2L1), nuclear gene encoding mitochondrial protein, transcript variant 1, mRNA [NM_138578] | -1,9970 | 4,92E-10 | 7,37E-08 |
| ALAS2 | NM_000032 | Homo sapiens aminolevulinate, delta-, synthase 2 (sideroblastic/hypochromic anemia) (ALAS2), nuclear gene encoding mitochondrial protein, transcript variant 1, mRNA [NM_000032] | -3,8863 | 5,64E-10 | 8,25E-08 |
| RAB3IL1 | NM_013401 | Homo sapiens RAB3A interacting protein (rabin3)-like 1 (RAB3IL1), mRNA [NM_013401] | -1,2555 | 5,64E-10 | 8,25E-08 |
| ENDOD1 | ENST00000278505 | Endonuclease domain-containing 1 protein precursor (EC 3.1.30.-). [Source:Uniprot/SWISSPROT;Acc:O94919] [ENST00000278505] | -1,6094 | 7,85E-10 | 1,11E-07 |
| LOC442239 | XR_018980 | PREDICTED: Homo sapiens similar to Peroxiredoxin-2 (Thioredoxin peroxidase 1) (Thioredoxin-dependent peroxide reductase 1) (Thiol-specific antioxidant protein) (TSA) (PRP) (Natural killer cell-enhancing factor B) (NKEF-B) (LOC442239), mRNA [XR_018980] | -1,8826 | 8,20E-10 | 1,13E-07 |
| A_32_P93894 | A_32_P93894 | Unknown | -0,8331 | 9,01E-10 | 1,22E-07 |
| RNF14 | NM_004290 | Homo sapiens ring finger protein 14 (RNF14), transcript variant 1, mRNA [NM_004290] | -1,2926 | 9,42E-10 | 1,27E-07 |
| CDC34 | NM_004359 | Homo sapiens cell division cycle 34 homolog (S. cerevisiae) (CDC34), mRNA [NM_004359] | -1,3598 | 9,69E-10 | 1,30E-07 |
| A_32_P208713 | A_32_P208713 | Unknown | -2,7389 | 9,82E-10 | 1,30E-07 |
| ANKRD9 | NM_152326 | Homo sapiens ankyrin repeat domain 9 (ANKRD9), mRNA [NM_152326] | -2,0648 | 1,01E-09 | 1,30E-07 |
| STEAP3 | NM_182915 | Homo sapiens STEAP family member 3 (STEAP3), transcript variant 1, mRNA [NM_182915] | -1,5365 | 1,01E-09 | 1,30E-07 |
| A_24_P25020 | A_24_P25020 | Unknown | -1,7396 | 1,10E-09 | 1,38E-07 |
| CCRL2 | NM_003965 | Homo sapiens chemokine (C-C motif) receptor-like 2 (CCRL2), mRNA [NM_003965] | -1,7734 | 1,17E-09 | 1,46E-07 |
| KCNH2 | NM_172056 | Homo sapiens potassium voltage-gated channel, subfamily H (eag-related), member 2 (KCNH2), transcript variant 2, mRNA [NM_172056] | -1,3909 | 1,22E-09 | 1,51E-07 |
| TSPAN17 | NM_012171 | Homo sapiens tetraspanin 17 (TSPAN17), transcript variant 1, mRNA [NM_012171] | -1,2067 | 1,25E-09 | 1,53E-07 |
| CD242823 | CD242823 | AGENCOURT_14126724 NIH_MGC_179 Homo sapiens cDNA clone IMAGE:30385216 5', mRNA sequence [CD242823] | -1,3847 | 1,46E-09 | 1,76E-07 |
| PIGQ | NM_004204 | Homo sapiens phosphatidylinositol glycan anchor biosynthesis, class Q (PIGQ), transcript variant 2, mRNA [NM_004204] | -1,7696 | 1,51E-09 | 1,81E-07 |
| CA2 | NM_000067 | Homo sapiens carbonic anhydrase II (CA2), mRNA [NM_000067] | -2,8886 | 1,81E-09 | 2,10E-07 |
| GYPA | NM_002099 | Homo sapiens glycophorin A (MNS blood group) (GYPA), mRNA [NM_002099] | -2,7430 | 1,82E-09 | 2,10E-07 |
| GAS2L1 | NM_152237 | Homo sapiens growth arrest-specific 2 like 1 (GAS2L1), transcript variant 3, mRNA [NM_152237] | -2,0814 | 2,29E-09 | 2,59E-07 |
| RNF123 | NM_022064 | Homo sapiens ring finger protein 123 (RNF123), mRNA [NM_022064] | -1,5835 | 2,36E-09 | 2,65E-07 |
| ALAD | NM_001003945 | Homo sapiens aminolevulinate, delta-, dehydratase (ALAD), transcript variant 1, mRNA [NM_001003945] | -1,8258 | 2,39E-09 | 2,66E-07 |
| GLRX5 | NM_016417 | Homo sapiens glutaredoxin 5 homolog (S. cerevisiae) (GLRX5), mRNA [NM_016417] | -2,0470 | 2,47E-09 | 2,71E-07 |
| ABCB10 | NM_012089 | Homo sapiens ATP-binding cassette, sub-family B (MDR/TAP), member 10 (ABCB10), nuclear gene encoding mitochondrial protein, mRNA [NM_012089] | -1,8082 | 2,51E-09 | 2,74E-07 |
| RCL1 | NM_005772 | Homo sapiens RNA terminal phosphate cyclase-like 1 (RCL1), mRNA [NM_005772] | -1,2480 | 2,57E-09 | 2,77E-07 |
| NAP1L4 | NM_005969 | Homo sapiens nucleosome assembly protein 1-like 4 (NAP1L4), mRNA [NM_005969] | -1,0522 | 2,70E-09 | 2,88E-07 |
| NCOA4 | NM_005437 | Homo sapiens nuclear receptor coactivator 4 (NCOA4), mRNA [NM_005437] | -1,4669 | 3,02E-09 | 3,19E-07 |
| BLVRB | NM_000713 | Homo sapiens biliverdin reductase B (flavin reductase (NADPH)) (BLVRB), mRNA [NM_000713] | -1,9841 | 3,16E-09 | 3,27E-07 |
| ARHGAP23 | XM_290799 | PREDICTED: Homo sapiens Rho GTPase activating protein 23, transcript variant 1 (ARHGAP23), mRNA [XM_290799] | -1,8228 | 3,32E-09 | 3,42E-07 |
| ENST00000370857 | ENST00000370857 | Muscleblind-like X-linked protein (Muscleblind-like protein 3) (Cys3His CCG1-required protein) (Protein HCHCR). [Source:Uniprot/SWISSPROT;Acc:Q9NUK0] [ENST00000370857] | -2,0593 | 3,41E-09 | 3,49E-07 |
| ANKRD41 | NM_152363 | Homo sapiens ankyrin repeat domain 41 (ANKRD41), mRNA [NM_152363] | -1,4495 | 3,44E-09 | 3,50E-07 |
| SFRS2B | NM_032102 | Homo sapiens splicing factor, arginine/serine-rich 2B (SFRS2B), mRNA [NM_032102] | -1,7494 | 3,79E-09 | 3,80E-07 |
| CPOX | NM_000097 | Homo sapiens coproporphyrinogen oxidase (CPOX), mRNA [NM_000097] | -1,7583 | 4,15E-09 | 4,11E-07 |
| MOBKL1A | NM_173468 | Homo sapiens MOB1, Mps One Binder kinase activator-like 1A (yeast) (MOBKL1A), mRNA [NM_173468] | -1,7965 | 4,22E-09 | 4,16E-07 |
| C1orf198 | NM_032800 | Homo sapiens chromosome 1 open reading frame 198 (C1orf198), mRNA [NM_032800] | -1,3785 | 5,33E-09 | 5,14E-07 |
| YPEL4 | NM_145008 | Homo sapiens yippee-like 4 (Drosophila) (YPEL4), mRNA [NM_145008] | -3,0645 | 5,99E-09 | 5,60E-07 |
| LOC253012 | NM_001039372 | Homo sapiens hypothetical protein LOC253012 (LOC253012), transcript variant 1, mRNA [NM_001039372] | -2,0778 | 6,03E-09 | 5,61E-07 |
| OPTN | NM_001008211 | Homo sapiens optineurin (OPTN), transcript variant 1, mRNA [NM_001008211] | -2,0379 | 6,23E-09 | 5,72E-07 |
| ST6GALNAC4 | NM_175039 | Homo sapiens ST6 (alpha-N-acetyl-neuraminyl-2,3-beta-galactosyl-1,3)-N-acetylgalactosaminide alpha-2,6-sialyltransferase 4 (ST6GALNAC4), transcript variant 1, mRNA [NM_175039] | -1,2939 | 6,21E-09 | 5,72E-07 |
| TUBB | NM_178014 | Homo sapiens tubulin, beta (TUBB), mRNA [NM_178014] | -0,9701 | 6,52E-09 | 5,95E-07 |
| CHST2 | NM_004267 | Homo sapiens carbohydrate (N-acetylglucosamine-6-O) sulfotransferase 2 (CHST2), mRNA [NM_004267] | -1,8225 | 6,60E-09 | 6,00E-07 |
| DPF3 | AK124946 | Homo sapiens cDNA FLJ42956 fis, clone BRSTN2009899. [AK124946] | -1,4629 | 6,76E-09 | 6,11E-07 |
| NFIA | NM_005595 | Homo sapiens nuclear factor I/A (NFIA), mRNA [NM_005595] | -1,8257 | 6,87E-09 | 6,18E-07 |
| AADACL1 | NM_020792 | Homo sapiens arylacetamide deacetylase-like 1 (AADACL1), mRNA [NM_020792] | -1,7104 | 7,30E-09 | 6,46E-07 |
| PPOX | NM_000309 | Homo sapiens protoporphyrinogen oxidase (PPOX), nuclear gene encoding mitochondrial protein, mRNA [NM_000309] | -1,5155 | 7,31E-09 | 6,46E-07 |
| DDB1 | NM_001923 | Homo sapiens damage-specific DNA binding protein 1, 127kDa (DDB1), mRNA [NM_001923] | -1,1646 | 7,45E-09 | 6,51E-07 |
| HNRPAB | NM_004499 | Homo sapiens heterogeneous nuclear ribonucleoprotein A/B (HNRPAB), transcript variant 2, mRNA [NM_004499] | -1,1109 | 7,47E-09 | 6,51E-07 |
| LOC283177 | AK095081 | Homo sapiens cDNA FLJ37762 fis, clone BRHIP2024347, weakly similar to GALECTIN-3. [AK095081] | -2,7864 | 7,48E-09 | 6,51E-07 |
| RNF182 | NM_152737 | Homo sapiens ring finger protein 182 (RNF182), mRNA [NM_152737] | -1,9656 | 7,69E-09 | 6,64E-07 |
| TAL1 | NM_003189 | Homo sapiens T-cell acute lymphocytic leukemia 1 (TAL1), mRNA [NM_003189] | -1,4807 | 7,87E-09 | 6,76E-07 |
| CDC25A | NM_001789 | Homo sapiens cell division cycle 25 homolog A (S. cerevisiae) (CDC25A), transcript variant 1, mRNA [NM_001789] | -1,1735 | 8,05E-09 | 6,88E-07 |
| HBD | NM_000519 | Homo sapiens hemoglobin, delta (HBD), mRNA [NM_000519] | -2,9721 | 8,34E-09 | 7,06E-07 |
| RNF187 | BC012758 | Homo sapiens ring finger protein 187, mRNA (cDNA clone IMAGE:3633225), partial cds. [BC012758] | -1,0627 | 8,71E-09 | 7,31E-07 |
| TUBG1 | NM_001070 | Homo sapiens tubulin, gamma 1 (TUBG1), mRNA [NM_001070] | -1,4751 | 9,18E-09 | 7,64E-07 |
| SPTA1 | NM_003126 | Homo sapiens spectrin, alpha, erythrocytic 1 (elliptocytosis 2) (SPTA1), mRNA [NM_003126] | -2,5707 | 9,42E-09 | 7,80E-07 |
| APEH | NM_001640 | Homo sapiens N-acylaminoacyl-peptide hydrolase (APEH), mRNA [NM_001640] | -1,1600 | 9,67E-09 | 7,98E-07 |
| SLC25A38 | NM_017875 | Homo sapiens solute carrier family 25, member 38 (SLC25A38), mRNA [NM_017875] | -1,1866 | 1,06E-08 | 8,60E-07 |
| JAZF1 | NM_175061 | Homo sapiens JAZF zinc finger 1 (JAZF1), mRNA [NM_175061] | -1,3279 | 1,12E-08 | 9,06E-07 |
| THC2663297 | THC2663297 | Q5VWT3_HUMAN (Q5VWT3) Complement component (3b/4b) receptor 1-like, partial (27%) [THC2663297] | -1,7917 | 1,22E-08 | 9,84E-07 |
| HBM | NM_001003938 | Homo sapiens hemoglobin, mu (HBM), mRNA [NM_001003938] | -3,6998 | 1,24E-08 | 9,95E-07 |
| C20orf175 | NM_080829 | Homo sapiens chromosome 20 open reading frame 175 (C20orf175), mRNA [NM_080829] | -1,4622 | 1,28E-08 | 1,02E-06 |
| C13orf8 | NM_032436 | Homo sapiens chromosome 13 open reading frame 8 (C13orf8), mRNA [NM_032436] | -0,9825 | 1,36E-08 | 1,07E-06 |
| THC2663668 | THC2663668 | Unknown | -0,9160 | 1,35E-08 | 1,07E-06 |
| ENST00000383048 | ENST00000383048 | Ig gamma-1 chain C region. [Source:Uniprot/SWISSPROT;Acc:P01857] [ENST00000383048] | -1,5925 | 1,37E-08 | 1,07E-06 |
| FLJ36208 | NM_176677 | Homo sapiens hypothetical protein FLJ36208 (FLJ36208), mRNA [NM_176677] | -1,0663 | 1,40E-08 | 1,09E-06 |
| A_24_P7330 | A_24_P7330 | Unknown | -1,7819 | 1,45E-08 | 1,12E-06 |
| TMEM15 | NM_014908 | Homo sapiens transmembrane protein 15 (TMEM15), mRNA [NM_014908] | -0,8692 | 1,45E-08 | 1,12E-06 |
| THC2530075 | THC2530075 | Unknown | -0,9813 | 1,49E-08 | 1,14E-06 |
| CA1 | NM_001738 | Homo sapiens carbonic anhydrase I (CA1), mRNA [NM_001738] | -2,9505 | 1,55E-08 | 1,18E-06 |
| MYBL2 | NM_002466 | Homo sapiens v-myb myeloblastosis viral oncogene homolog (avian)-like 2 (MYBL2), mRNA [NM_002466] | -1,3497 | 1,56E-08 | 1,18E-06 |
| UROD | NM_000374 | Homo sapiens uroporphyrinogen decarboxylase (UROD), mRNA [NM_000374] | -1,8193 | 1,87E-08 | 1,39E-06 |
| BC031344 | BC031344 | Homo sapiens, Similar to makorin, ring finger protein, 1, clone IMAGE:5556543, mRNA. [BC031344] | -1,5762 | 1,94E-08 | 1,44E-06 |
| SLC22A16 | NM_033125 | Homo sapiens solute carrier family 22 (organic cation transporter), member 16 (SLC22A16), mRNA [NM_033125] | -1,6623 | 1,96E-08 | 1,45E-06 |
| TMPRSS9 | AK131261 | Homo sapiens cDNA FLJ16193 fis, clone BRTHA2018011, weakly similar to EPITHIN (EC 3.4.21.-). [AK131261] | -1,2788 | 2,01E-08 | 1,48E-06 |
| UBE2O | NM_022066 | Homo sapiens ubiquitin-conjugating enzyme E2O (UBE2O), mRNA [NM_022066] | -1,7551 | 2,03E-08 | 1,48E-06 |
| CXCL12 | NM_199168 | Homo sapiens chemokine (C-X-C motif) ligand 12 (stromal cell-derived factor 1) (CXCL12), transcript variant 1, mRNA [NM_199168] | -2,0945 | 2,07E-08 | 1,49E-06 |
| GFI1B | NM_004188 | Homo sapiens growth factor independent 1B (potential regulator of CDKN1A, translocated in CML) (GFI1B), mRNA [NM_004188] | -1,9783 | 2,08E-08 | 1,49E-06 |
| SNX22 | NM_024798 | Homo sapiens sorting nexin 22 (SNX22), mRNA [NM_024798] | -1,5546 | 2,08E-08 | 1,49E-06 |
| ERAF | NM_016633 | Homo sapiens erythroid associated factor (ERAF), mRNA [NM_016633] | -2,5394 | 2,09E-08 | 1,50E-06 |
| MYL4 | NM_002476 | Homo sapiens myosin, light chain 4, alkali; atrial, embryonic (MYL4), transcript variant 2, mRNA [NM_002476] | -2,2854 | 2,17E-08 | 1,54E-06 |
| MCM2 | NM_004526 | Homo sapiens MCM2 minichromosome maintenance deficient 2, mitotin (S. cerevisiae) (MCM2), mRNA [NM_004526] | -1,2511 | 2,24E-08 | 1,59E-06 |
| TMEM111 | ENST00000383810 | Transmembrane protein 111. [Source:Uniprot/SWISSPROT;Acc:Q9P0I2] [ENST00000383810] | -1,4404 | 2,34E-08 | 1,66E-06 |
| MKRN1 | NM_013446 | Homo sapiens makorin, ring finger protein, 1 (MKRN1), mRNA [NM_013446] | -1,6200 | 2,45E-08 | 1,72E-06 |
| SLC7A5 | NM_003486 | Homo sapiens solute carrier family 7 (cationic amino acid transporter, y+ system), member 5 (SLC7A5), mRNA [NM_003486] | -2,1415 | 2,44E-08 | 1,72E-06 |
| SPBC24 | NM_182513 | Homo sapiens spindle pole body component 24 homolog (S. cerevisiae) (SPBC24), mRNA [NM_182513] | -1,0187 | 2,71E-08 | 1,88E-06 |
| TMEM23 | NM_147156 | Homo sapiens transmembrane protein 23 (TMEM23), mRNA [NM_147156] | -0,9057 | 2,76E-08 | 1,91E-06 |
| THC2522223 | THC2522223 | AF192784 makorin 1 {Homo sapiens} (exp=-1; wgp=0; cg=0), partial (15%) [THC2522223] | -1,0816 | 2,95E-08 | 2,02E-06 |
| FAM83D | NM_030919 | Homo sapiens family with sequence similarity 83, member D (FAM83D), mRNA [NM_030919] | -1,2869 | 3,02E-08 | 2,06E-06 |
| CKAP2L | NM_152515 | Homo sapiens cytoskeleton associated protein 2-like (CKAP2L), mRNA [NM_152515] | -1,1358 | 3,06E-08 | 2,09E-06 |
| GSPT1 | NM_002094 | Homo sapiens G1 to S phase transition 1 (GSPT1), mRNA [NM_002094] | -1,3810 | 3,13E-08 | 2,12E-06 |
| BC000986 | BC000986 | Homo sapiens cDNA clone IMAGE:3446313, complete cds. [BC000986] | -1,3531 | 3,25E-08 | 2,20E-06 |
| PBX1 | ENST00000328681 | Pre-B-cell leukemia transcription factor 1 (Homeobox protein PBX1) (Homeobox protein PRL). [Source:Uniprot/SWISSPROT;Acc:P40424] [ENST00000328681] | -1,2653 | 3,45E-08 | 2,32E-06 |
| AK125361 | AK125361 | Homo sapiens cDNA FLJ43371 fis, clone NTONG2005969. [AK125361] | -1,6564 | 3,47E-08 | 2,32E-06 |
| ZNF416 | NM_017879 | Homo sapiens zinc finger protein 416 (ZNF416), mRNA [NM_017879] | -0,8801 | 3,50E-08 | 2,33E-06 |
| ASCC2 | NM_032204 | Homo sapiens activating signal cointegrator 1 complex subunit 2 (ASCC2), mRNA [NM_032204] | -1,0672 | 3,55E-08 | 2,35E-06 |
| GDPD5 | NM_030792 | Homo sapiens glycerophosphodiester phosphodiesterase domain containing 5 (GDPD5), mRNA [NM_030792] | -1,4112 | 3,55E-08 | 2,35E-06 |
| IFRD2 | NM_006764 | Homo sapiens interferon-related developmental regulator 2 (IFRD2), mRNA [NM_006764] | -1,0361 | 4,03E-08 | 2,60E-06 |
| CCNE1 | NM_001238 | Homo sapiens cyclin E1 (CCNE1), transcript variant 1, mRNA [NM_001238] | -1,1488 | 4,20E-08 | 2,70E-06 |
| BMP2K | NM_017593 | Homo sapiens BMP2 inducible kinase (BMP2K), transcript variant 2, mRNA [NM_017593] | -0,9224 | 4,49E-08 | 2,87E-06 |
| AF332145 | AF332145 | Homo sapiens anti-pneumococcal antibody NAD light chain variable region mRNA, partial cds. [AF332145] | -1,6991 | 4,71E-08 | 2,97E-06 |
| WNK1 | AB002342 | Human mRNA for KIAA0344 gene, partial cds. [AB002342] | -1,0757 | 4,79E-08 | 3,01E-06 |
| MINPP1 | NM_004897 | Homo sapiens multiple inositol polyphosphate histidine phosphatase, 1 (MINPP1), mRNA [NM_004897] | -1,3472 | 4,98E-08 | 3,10E-06 |
| C1orf128 | NM_020362 | Homo sapiens chromosome 1 open reading frame 128 (C1orf128), mRNA [NM_020362] | -1,3225 | 5,08E-08 | 3,12E-06 |
| CDC27 | NM_001256 | Homo sapiens cell division cycle 27 homolog (S. cerevisiae) (CDC27), mRNA [NM_001256] | -1,2100 | 5,05E-08 | 3,12E-06 |
| MYH10 | NM_005964 | Homo sapiens myosin, heavy chain 10, non-muscle (MYH10), mRNA [NM_005964] | -0,9257 | 5,07E-08 | 3,12E-06 |
| VWCE | NM_152718 | Homo sapiens von Willebrand factor C and EGF domains (VWCE), mRNA [NM_152718] | -1,9481 | 5,05E-08 | 3,12E-06 |
| IGKV1-5 | BC034142 | Homo sapiens immunoglobulin kappa variable 1-5, mRNA (cDNA clone MGC:32715 IMAGE:4694346), complete cds. [BC034142] | -1,8340 | 5,14E-08 | 3,13E-06 |
| VKORC1 | NM_206824 | Homo sapiens vitamin K epoxide reductase complex, subunit 1 (VKORC1), transcript variant 2, mRNA [NM_206824] | -1,3016 | 5,43E-08 | 3,30E-06 |
| IGHA1 | AK128476 | Homo sapiens cDNA FLJ46621 fis, clone TLUNG2001445, highly similar to Ig alpha-1 chain C region. [AK128476] | -3,4968 | 5,66E-08 | 3,43E-06 |
| PRPS1 | NM_002764 | Homo sapiens phosphoribosyl pyrophosphate synthetase 1 (PRPS1), mRNA [NM_002764] | -1,5248 | 5,74E-08 | 3,47E-06 |
| CMAS | NM_018686 | Homo sapiens cytidine monophosphate N-acetylneuraminic acid synthetase (CMAS), mRNA [NM_018686] | -1,3751 | 5,89E-08 | 3,54E-06 |
| THC2527772 | THC2527772 | HUMC4AA2 complement component C4A {Homo sapiens} (exp=-1; wgp=0; cg=0), partial (6%) [THC2527772] | -0,9416 | 6,05E-08 | 3,63E-06 |
| CHID1 | NM_023947 | Homo sapiens chitinase domain containing 1 (CHID1), mRNA [NM_023947] | -1,0550 | 6,64E-08 | 3,96E-06 |
| FBL | NM_001436 | Homo sapiens fibrillarin (FBL), mRNA [NM_001436] | -0,9334 | 6,86E-08 | 4,08E-06 |
| OPA1 | NM_130837 | Homo sapiens optic atrophy 1 (autosomal dominant) (OPA1), nuclear gene encoding mitochondrial protein, transcript variant 8, mRNA [NM_130837] | -1,0971 | 7,00E-08 | 4,12E-06 |
| NEDD4L | NM_015277 | Homo sapiens neural precursor cell expressed, developmentally down-regulated 4-like (NEDD4L), mRNA [NM_015277] | -1,0348 | 7,09E-08 | 4,15E-06 |
| FAM117A | NM_030802 | Homo sapiens family with sequence similarity 117, member A (FAM117A), mRNA [NM_030802] | -1,7972 | 7,42E-08 | 4,33E-06 |
| MARCH3 | NM_178450 | Homo sapiens membrane-associated ring finger (C3HC4) 3 (MARCH3), mRNA [NM_178450] | -1,4536 | 8,15E-08 | 4,71E-06 |
| BC035146 | BC035146 | Homo sapiens cDNA clone IMAGE:5264735. [BC035146] | -1,6266 | 8,39E-08 | 4,83E-06 |
| ABCB6 | NM_005689 | Homo sapiens ATP-binding cassette, sub-family B (MDR/TAP), member 6 (ABCB6), nuclear gene encoding mitochondrial protein, mRNA [NM_005689] | -2,0008 | 9,02E-08 | 5,13E-06 |
| GUK1 | NM_000858 | Homo sapiens guanylate kinase 1 (GUK1), mRNA [NM_000858] | -1,1886 | 9,19E-08 | 5,21E-06 |
| FAHD1 | NM_031208 | Homo sapiens fumarylacetoacetate hydrolase domain containing 1 (FAHD1), transcript variant 2, mRNA [NM_031208] | -0,9934 | 9,35E-08 | 5,29E-06 |
| BNIP3L | NM_004331 | Homo sapiens BCL2/adenovirus E1B 19kDa interacting protein 3-like (BNIP3L), mRNA [NM_004331] | -1,8557 | 9,75E-08 | 5,48E-06 |
| SMOX | NM_175839 | Homo sapiens spermine oxidase (SMOX), transcript variant 1, mRNA [NM_175839] | -1,9755 | 9,96E-08 | 5,58E-06 |
| SDC1 | NM_001006946 | Homo sapiens syndecan 1 (SDC1), transcript variant 1, mRNA [NM_001006946] | -1,5637 | 1,01E-07 | 5,62E-06 |
| HES5 | NM_001010926 | Homo sapiens hairy and enhancer of split 5 (Drosophila) (HES5), mRNA [NM_001010926] | -1,1773 | 1,01E-07 | 5,63E-06 |
| C4B | NM_001002029 | Homo sapiens complement component 4B (Childo blood group) (C4B), mRNA [NM_001002029] | -1,2518 | 1,04E-07 | 5,76E-06 |
| AW302758 | AW302758 | AW302758 xr55g08.x1 NCI_CGAP_Ov26 Homo sapiens cDNA clone IMAGE:2764094 3', mRNA sequence [AW302758] | -1,4578 | 1,10E-07 | 6,07E-06 |
| EIF4G1 | NM_182917 | Homo sapiens eukaryotic translation initiation factor 4 gamma, 1 (EIF4G1), transcript variant 1, mRNA [NM_182917] | -0,7709 | 1,12E-07 | 6,13E-06 |
| A_24_P298179 | A_24_P298179 | Unknown | -1,2346 | 1,17E-07 | 6,42E-06 |
| WDR34 | NM_052844 | Homo sapiens WD repeat domain 34 (WDR34), mRNA [NM_052844] | -1,3246 | 1,18E-07 | 6,43E-06 |
| DLC1 | NM_182643 | Homo sapiens deleted in liver cancer 1 (DLC1), transcript variant 1, mRNA [NM_182643] | -1,5225 | 1,22E-07 | 6,62E-06 |
| FEM1A | NM_018708 | Homo sapiens fem-1 homolog a (C. elegans) (FEM1A), mRNA [NM_018708] | -1,0611 | 1,24E-07 | 6,71E-06 |
| CR601260 | CR601260 | full-length cDNA clone CS0DM001YA20 of Fetal liver of Homo sapiens (human). [CR601260] | -1,5678 | 1,26E-07 | 6,77E-06 |
| FZD1 | NM_003505 | Homo sapiens frizzled homolog 1 (Drosophila) (FZD1), mRNA [NM_003505] | -0,8737 | 1,32E-07 | 7,08E-06 |
| CENPO | AK027859 | Homo sapiens cDNA FLJ14953 fis, clone PLACE3000160. [AK027859] | -1,1727 | 1,35E-07 | 7,17E-06 |
| DNAJA4 | NM_018602 | Homo sapiens DnaJ (Hsp40) homolog, subfamily A, member 4 (DNAJA4), mRNA [NM_018602] | -1,7256 | 1,36E-07 | 7,19E-06 |
| BC032451 | BC032451 | Homo sapiens cDNA clone MGC:40426 IMAGE:5178085, complete cds. [BC032451] | -1,9086 | 1,39E-07 | 7,35E-06 |
| ENST00000283657 | ENST00000283657 | V kappa 4=immunoglobulin light chain variable region {complementarity determining regions} [human, CD5+ tonsillar B cells, mRNA PartialMutant, 303 nt]. [S62210] | -2,0907 | 1,40E-07 | 7,37E-06 |
| TMEM86B | NM_173804 | Homo sapiens transmembrane protein 86B (TMEM86B), mRNA [NM_173804] | -1,1199 | 1,44E-07 | 7,57E-06 |
| EPOR | NM_000121 | Homo sapiens erythropoietin receptor (EPOR), mRNA [NM_000121] | -1,7574 | 1,46E-07 | 7,63E-06 |
| CD709370 | CD709370 | CD709370 EST25897 human nasopharynx Homo sapiens cDNA, mRNA sequence [CD709370] | -1,4276 | 1,56E-07 | 8,06E-06 |
| S76132 | S76132 | Ig V lambda II=IgG rheumatoid factor [human, hybridoma AEE111F, mRNA Partial, 315 nt]. [S76132] | -2,2601 | 1,57E-07 | 8,07E-06 |
| PC | NM_001040716 | Homo sapiens pyruvate carboxylase (PC), nuclear gene encoding mitochondrial protein, transcript variant 3, mRNA [NM_001040716] | -1,0166 | 1,58E-07 | 8,10E-06 |
| SLC7A1 | NM_003045 | Homo sapiens solute carrier family 7 (cationic amino acid transporter, y+ system), member 1 (SLC7A1), mRNA [NM_003045] | -0,9735 | 1,63E-07 | 8,30E-06 |
| C6orf85 | BC022217 | Homo sapiens chromosome 6 open reading frame 85, mRNA (cDNA clone IMAGE:3846727), complete cds. [BC022217] | -1,9493 | 1,71E-07 | 8,69E-06 |
| THC2618446 | THC2618446 | Q213Y3_RHOPA (Q213Y3) Single-strand binding protein, partial (9%) [THC2618446] | -1,0396 | 1,81E-07 | 9,11E-06 |
| RANBP10 | NM_020850 | Homo sapiens RAN binding protein 10 (RANBP10), mRNA [NM_020850] | -1,4843 | 1,83E-07 | 9,19E-06 |
| PCAF | NM_003884 | Homo sapiens p300/CBP-associated factor (PCAF), mRNA [NM_003884] | -1,4756 | 1,85E-07 | 9,25E-06 |
| C22orf13 | NM_031444 | Homo sapiens chromosome 22 open reading frame 13 (C22orf13), mRNA [NM_031444] | -1,3149 | 1,93E-07 | 9,62E-06 |
| PIGC | AL035301 | H.sapiens gene from PAC 106H8. [AL035301] | -1,9297 | 1,98E-07 | 9,78E-06 |
| A_24_P203886 | A_24_P203886 | Unknown | -1,1870 | 2,32E-07 | 1,13E-05 |
| SLC38A5 | NM_033518 | Homo sapiens solute carrier family 38, member 5 (SLC38A5), mRNA [NM_033518] | -1,3012 | 2,32E-07 | 1,13E-05 |
| RPUSD2 | NM_152260 | Homo sapiens RNA pseudouridylate synthase domain containing 2 (RPUSD2), mRNA [NM_152260] | -1,2456 | 2,34E-07 | 1,14E-05 |
| TOP1 | NM_003286 | Homo sapiens topoisomerase (DNA) I (TOP1), mRNA [NM_003286] | -0,8769 | 2,56E-07 | 1,23E-05 |
| UBADC1 | NM_016172 | Homo sapiens ubiquitin associated domain containing 1 (UBADC1), mRNA [NM_016172] | -1,3828 | 2,56E-07 | 1,23E-05 |
| IDH2 | NM_002168 | Homo sapiens isocitrate dehydrogenase 2 (NADP+), mitochondrial (IDH2), mRNA [NM_002168] | -1,1548 | 2,61E-07 | 1,25E-05 |
| CSDA | NM_003651 | Homo sapiens cold shock domain protein A (CSDA), mRNA [NM_003651] | -1,3964 | 2,64E-07 | 1,25E-05 |
| CYC1 | NM_001916 | Homo sapiens cytochrome c-1 (CYC1), mRNA [NM_001916] | -0,8739 | 2,64E-07 | 1,25E-05 |
| USP12 | ENST00000258451 | Ubiquitin carboxyl-terminal hydrolase 12 (EC 3.1.2.15) (Ubiquitin thioesterase 12) (Ubiquitin-specific-processing protease 12) (Deubiquitinating enzyme 12) (Ubiquitin-hydrolyzing enzyme 1). [Source:Uniprot/SWISSPROT;Acc:O75317] [ENST00000258451] | -1,3660 | 2,63E-07 | 1,25E-05 |
| BTRC | NM_033637 | Homo sapiens beta-transducin repeat containing (BTRC), transcript variant 1, mRNA [NM_033637] | -1,1312 | 2,74E-07 | 1,29E-05 |
| FBXO30 | NM_032145 | Homo sapiens F-box protein 30 (FBXO30), mRNA [NM_032145] | -1,3096 | 2,84E-07 | 1,33E-05 |
| EPN2 | NM_014964 | Homo sapiens epsin 2 (EPN2), transcript variant 2, mRNA [NM_014964] | -1,3102 | 2,92E-07 | 1,36E-05 |
| RNF26 | NM_032015 | Homo sapiens ring finger protein 26 (RNF26), mRNA [NM_032015] | -0,8786 | 3,06E-07 | 1,42E-05 |
| AW804491 | AW804491 | AW804491 QV0-UM0093-170400-191-d05 UM0093 Homo sapiens cDNA, mRNA sequence [AW804491] | -0,9390 | 3,10E-07 | 1,44E-05 |
| HBA1 | NM_000558 | Homo sapiens hemoglobin, alpha 1 (HBA1), mRNA [NM_000558] | -2,4343 | 3,27E-07 | 1,51E-05 |
| AMMECR1 | NM_015365 | Homo sapiens Alport syndrome, mental retardation, midface hypoplasia and elliptocytosis chromosomal region, gene 1 (AMMECR1), transcript variant 1, mRNA [NM_015365] | -0,9741 | 3,49E-07 | 1,60E-05 |
| C18orf10 | NM_015476 | Homo sapiens chromosome 18 open reading frame 10 (C18orf10), mRNA [NM_015476] | -0,9839 | 3,62E-07 | 1,66E-05 |
| NP | NM_000270 | Homo sapiens nucleoside phosphorylase (NP), mRNA [NM_000270] | -1,3538 | 3,63E-07 | 1,66E-05 |
| FGFR3 | NM_000142 | Homo sapiens fibroblast growth factor receptor 3 (achondroplasia, thanatophoric dwarfism) (FGFR3), transcript variant 1, mRNA [NM_000142] | -0,8840 | 3,72E-07 | 1,69E-05 |
| PGRMC2 | NM_006320 | Homo sapiens progesterone receptor membrane component 2 (PGRMC2), mRNA [NM_006320] | -1,3176 | 3,82E-07 | 1,72E-05 |
| FRMD4A | AK001072 | Homo sapiens cDNA FLJ10210 fis, clone HEMBA1006344, weakly similar to RADIXIN. [AK001072] | -2,1310 | 3,86E-07 | 1,73E-05 |
| ENST00000312946 | ENST00000312946 | AY320849 immunoglobulin kappa chain variable region {Homo sapiens} (exp=-1; wgp=0; cg=0), complete [THC2568849] | -1,2798 | 3,98E-07 | 1,78E-05 |
| CR603982 | CR603982 | full-length cDNA clone CS0DF021YL03 of Fetal brain of Homo sapiens (human). [CR603982] | -1,2120 | 4,03E-07 | 1,80E-05 |
| CDYL | NM_170752 | Homo sapiens chromodomain protein, Y-like (CDYL), transcript variant 3, mRNA [NM_170752] | -0,9121 | 4,13E-07 | 1,84E-05 |
| MGC17403 | ENST00000314720 | TFS2-M domain-containing protein 1. [Source:Uniprot/SWISSPROT;Acc:Q8N8B7] [ENST00000314720] | -1,1096 | 4,21E-07 | 1,86E-05 |
| CD36 | S67044 | CD36=collagen type I/thrombospondin receptor {one exon} [human, mRNA Partial, 369 nt]. [S67044] | -1,2859 | 4,30E-07 | 1,90E-05 |
| CTNNA1 | NM_001903 | Homo sapiens catenin (cadherin-associated protein), alpha 1, 102kDa (CTNNA1), mRNA [NM_001903] | -0,9093 | 4,44E-07 | 1,95E-05 |
| ENST00000295410 | ENST00000295410 | Ig kappa chain V-I region HK101 precursor (Fragment). [Source:Uniprot/SWISSPROT;Acc:P01601] [ENST00000377228] | -1,8218 | 4,55E-07 | 1,99E-05 |
| SLC43A1 | NM_003627 | Homo sapiens solute carrier family 43, member 1 (SLC43A1), mRNA [NM_003627] | -0,9870 | 4,58E-07 | 2,00E-05 |
| TFDP1 | NM_007111 | Homo sapiens transcription factor Dp-1 (TFDP1), mRNA [NM_007111] | -1,0427 | 4,60E-07 | 2,00E-05 |
| ANKRD25 | NM_015493 | Homo sapiens ankyrin repeat domain 25 (ANKRD25), mRNA [NM_015493] | -1,8591 | 5,01E-07 | 2,17E-05 |
| CTSL | NM_001912 | Homo sapiens cathepsin L (CTSL), transcript variant 1, mRNA [NM_001912] | -2,0284 | 5,25E-07 | 2,26E-05 |
| FAM104A | NM_032837 | Homo sapiens family with sequence similarity 104, member A (FAM104A), mRNA [NM_032837] | -1,6919 | 5,47E-07 | 2,35E-05 |
| CTB-1048E9.5 | NM_001013694 | Homo sapiens similar to SRR1-like protein (LOC402055), mRNA [NM_001013694] | -1,1218 | 5,60E-07 | 2,40E-05 |
| PVRL1 | NM_002855 | Homo sapiens poliovirus receptor-related 1 (herpesvirus entry mediator C; nectin) (PVRL1), transcript variant 1, mRNA [NM_002855] | -1,0276 | 5,75E-07 | 2,45E-05 |
| RNF5 | NM_006913 | Homo sapiens ring finger protein 5 (RNF5), mRNA [NM_006913] | -0,9643 | 5,74E-07 | 2,45E-05 |
| FLJ30092 | AB014514 | Homo sapiens mRNA for KIAA0614 protein, partial cds. [AB014514] | -1,1322 | 6,06E-07 | 2,56E-05 |
| IGHG1 | BC092518 | Homo sapiens immunoglobulin heavy constant gamma 1 (G1m marker), mRNA (cDNA clone MGC:105004 IMAGE:3056327), complete cds. [BC092518] | -1,6928 | 6,38E-07 | 2,69E-05 |
| ACOT7 | NM_007274 | Homo sapiens acyl-CoA thioesterase 7 (ACOT7), transcript variant hBACHa, mRNA [NM_007274] | -1,0548 | 6,46E-07 | 2,71E-05 |
| MXI1 | NM_130439 | Homo sapiens MAX interactor 1 (MXI1), transcript variant 2, mRNA [NM_130439] | -1,4071 | 6,47E-07 | 2,71E-05 |
| RAB6B | NM_016577 | Homo sapiens RAB6B, member RAS oncogene family (RAB6B), mRNA [NM_016577] | -1,4296 | 6,56E-07 | 2,74E-05 |
| PPP2R1B | NM_002716 | Homo sapiens protein phosphatase 2 (formerly 2A), regulatory subunit A (PR 65), beta isoform (PPP2R1B), transcript variant 1, mRNA [NM_002716] | -0,7064 | 6,60E-07 | 2,74E-05 |
| GNPDA1 | NM_005471 | Homo sapiens glucosamine-6-phosphate deaminase 1 (GNPDA1), mRNA [NM_005471] | -0,6090 | 6,62E-07 | 2,75E-05 |
| LPIN2 | NM_014646 | Homo sapiens lipin 2 (LPIN2), mRNA [NM_014646] | -0,9247 | 6,76E-07 | 2,80E-05 |
| POLR3H | NM_001018051 | Homo sapiens polymerase (RNA) III (DNA directed) polypeptide H (22.9kD) (POLR3H), transcript variant 4, mRNA [NM_001018051] | -1,1107 | 6,80E-07 | 2,80E-05 |
| LOC388588 | ENST00000378266 | Homo sapiens, clone IMAGE:5162922, mRNA. [BC035379] | -1,7247 | 7,05E-07 | 2,90E-05 |
| TUBG2 | NM_016437 | Homo sapiens tubulin, gamma 2 (TUBG2), mRNA [NM_016437] | -1,3352 | 7,24E-07 | 2,97E-05 |
| DAAM1 | NM_014992 | Homo sapiens dishevelled associated activator of morphogenesis 1 (DAAM1), mRNA [NM_014992] | -0,9683 | 7,27E-07 | 2,98E-05 |
| AY998685 | AY998685 | Homo sapiens isolate 13K immunoglobulin kappa light chain variable region (IGKV4) mRNA, IGKV4-1*01 allele, partial cds. [AY998685] | -1,2057 | 7,58E-07 | 3,09E-05 |
| ENST00000379913 | ENST00000379913 | Ig gamma-4 chain C region. [Source:Uniprot/SWISSPROT;Acc:P01861] [ENST00000379913] | -3,3650 | 7,57E-07 | 3,09E-05 |
| DYRK3 | NM_001004023 | Homo sapiens dual-specificity tyrosine-(Y)-phosphorylation regulated kinase 3 (DYRK3), transcript variant 2, mRNA [NM_001004023] | -1,4125 | 7,69E-07 | 3,12E-05 |
| LCMT2 | NM_014793 | Homo sapiens leucine carboxyl methyltransferase 2 (LCMT2), mRNA [NM_014793] | -0,7883 | 7,73E-07 | 3,13E-05 |
| UBXD1 | NM_025241 | Homo sapiens UBX domain containing 1 (UBXD1), mRNA [NM_025241] | -1,3557 | 8,06E-07 | 3,26E-05 |
| LNX2 | NM_153371 | Homo sapiens ligand of numb-protein X 2 (LNX2), mRNA [NM_153371] | -0,9388 | 8,12E-07 | 3,28E-05 |
| TXNDC5 | NM_022085 | Homo sapiens thioredoxin domain containing 5 (TXNDC5), transcript variant 2, mRNA [NM_022085] | -1,4657 | 8,14E-07 | 3,28E-05 |
| AKAP7 | NM_016377 | Homo sapiens A kinase (PRKA) anchor protein 7 (AKAP7), transcript variant gamma, mRNA [NM_016377] | -0,7988 | 8,19E-07 | 3,28E-05 |
| CDCA4 | NM_017955 | Homo sapiens cell division cycle associated 4 (CDCA4), transcript variant 13, mRNA [NM_017955] | -0,7238 | 8,17E-07 | 3,28E-05 |
| C22orf25 | NM_152906 | Homo sapiens chromosome 22 open reading frame 25 (C22orf25), mRNA [NM_152906] | -1,2943 | 8,65E-07 | 3,44E-05 |
| BC030813 | BC030813 | Homo sapiens cDNA clone MGC:22645 IMAGE:4700961, complete cds. [BC030813] | -1,9843 | 8,78E-07 | 3,48E-05 |
| NUS1 | NM_138459 | Homo sapiens nuclear undecaprenyl pyrophosphate synthase 1 homolog (S. cerevisiae) (NUS1), mRNA [NM_138459] | -0,8635 | 8,79E-07 | 3,48E-05 |
| AF471454 | AF471454 | Homo sapiens clone 68-46a Ig heavy chain variable region, VH3 family mRNA, partial cds. [AF471454] | -1,7100 | 9,19E-07 | 3,62E-05 |
| MGST3 | NM_004528 | Homo sapiens microsomal glutathione S-transferase 3 (MGST3), mRNA [NM_004528] | -1,6132 | 9,40E-07 | 3,70E-05 |
| FIS1 | NM_016068 | Homo sapiens fission 1 (mitochondrial outer membrane) homolog (S. cerevisiae) (FIS1), mRNA [NM_016068] | -0,8320 | 9,76E-07 | 3,82E-05 |
| A_24_P341126 | A_24_P341126 | Unknown | -1,7429 | 9,80E-07 | 3,82E-05 |
| KLF3 | ENST00000381956 | Krueppel-like factor 3 (Basic krueppel-like factor) (CACCC-box-binding protein BKLF) (TEF-2). [Source:Uniprot/SWISSPROT;Acc:P57682] [ENST00000381956] | -1,6525 | 9,93E-07 | 3,86E-05 |
| A_24_P755069 | A_24_P755069 | Unknown | -1,7827 | 1,04E-06 | 4,00E-05 |
| ASF1A | NM_014034 | Homo sapiens ASF1 anti-silencing function 1 homolog A (S. cerevisiae) (ASF1A), mRNA [NM_014034] | -0,8512 | 1,08E-06 | 4,13E-05 |
| VCAM1 | NM_001078 | Homo sapiens vascular cell adhesion molecule 1 (VCAM1), transcript variant 1, mRNA [NM_001078] | -1,1891 | 1,08E-06 | 4,13E-05 |
| FEN1 | NM_004111 | Homo sapiens flap structure-specific endonuclease 1 (FEN1), mRNA [NM_004111] | -1,1852 | 1,09E-06 | 4,18E-05 |
| APOBEC3F | NM_145298 | Homo sapiens apolipoprotein B mRNA editing enzyme, catalytic polypeptide-like 3F (APOBEC3F), transcript variant 1, mRNA [NM_145298] | -0,6739 | 1,11E-06 | 4,24E-05 |
| PKLR | NM_000298 | Homo sapiens pyruvate kinase, liver and RBC (PKLR), nuclear gene encoding mitochondrial protein, transcript variant 1, mRNA [NM_000298] | -0,9472 | 1,16E-06 | 4,40E-05 |
| AF267875 | AF267875 | Homo sapiens amyloid lambda 6 light chain variable region SAR mRNA, partial cds. [AF267875] | -1,1886 | 1,20E-06 | 4,53E-05 |
| GPR137B | AL832142 | Homo sapiens mRNA; cDNA DKFZp686A22111 (from clone DKFZp686A22111). [AL832142] | -1,1118 | 1,25E-06 | 4,72E-05 |
| ZNF23 | NM_145911 | Homo sapiens zinc finger protein 23 (KOX 16) (ZNF23), mRNA [NM_145911] | -0,7824 | 1,26E-06 | 4,74E-05 |
| ENST00000359488 | ENST00000359488 | Ig kappa chain V-I region Walker precursor. [Source:Uniprot/SWISSPROT;Acc:P04431] [ENST00000359488] | -1,8716 | 1,27E-06 | 4,76E-05 |
| LOC91461 | NM_138370 | Homo sapiens hypothetical protein BC007901 (LOC91461), mRNA [NM_138370] | -1,0097 | 1,29E-06 | 4,84E-05 |
| AK095108 | AK095108 | Homo sapiens cDNA FLJ37789 fis, clone BRHIP3000081. [AK095108] | -0,4966 | 1,31E-06 | 4,89E-05 |
| AY172962 | AY172962 | Homo sapiens anti-rabies SOJB immunoglobulin lambda light chain mRNA, complete cds. [AY172962] | -1,9991 | 1,34E-06 | 4,97E-05 |
| PGM2L1 | NM_173582 | Homo sapiens phosphoglucomutase 2-like 1 (PGM2L1), mRNA [NM_173582] | -1,0535 | 1,35E-06 | 5,01E-05 |
| SLC25A37 | AF495725 | Homo sapiens FP15737 mRNA, complete cds. [AF495725] | -2,2330 | 1,37E-06 | 5,08E-05 |
| TUBA3 | NM_006009 | Homo sapiens tubulin, alpha 3 (TUBA3), mRNA [NM_006009] | -1,0512 | 1,40E-06 | 5,19E-05 |
| FAM82C | NM_018145 | Homo sapiens family with sequence similarity 82, member C (FAM82C), mRNA [NM_018145] | -1,1430 | 1,41E-06 | 5,19E-05 |
| MCAT | NM_173467 | Homo sapiens malonyl CoA:ACP acyltransferase (mitochondrial) (MCAT), nuclear gene encoding mitochondrial protein, transcript variant 1, mRNA [NM_173467] | -0,7509 | 1,41E-06 | 5,20E-05 |
| IL15RA | NM_172200 | Homo sapiens interleukin 15 receptor, alpha (IL15RA), transcript variant 2, mRNA [NM_172200] | -1,0394 | 1,41E-06 | 5,20E-05 |
| A_32_P108592 | A_32_P108592 | Unknown | -0,8540 | 1,44E-06 | 5,27E-05 |
| ENST00000331195 | ENST00000331195 | V1-16 protein (Fragment). [Source:Uniprot/SPTREMBL;Acc:Q5NV81] [ENST00000331195] | -1,8052 | 1,44E-06 | 5,28E-05 |
| ODC1 | NM_002539 | Homo sapiens ornithine decarboxylase 1 (ODC1), mRNA [NM_002539] | -1,2671 | 1,48E-06 | 5,39E-05 |
| FREQ | NM_014286 | Homo sapiens frequenin homolog (Drosophila) (FREQ), mRNA [NM_014286] | -1,3504 | 1,53E-06 | 5,56E-05 |
| KIFC1 | NM_002263 | Homo sapiens kinesin family member C1 (KIFC1), mRNA [NM_002263] | -1,0389 | 1,53E-06 | 5,56E-05 |
| RAP2A | NM_021033 | Homo sapiens RAP2A, member of RAS oncogene family (RAP2A), mRNA [NM_021033] | -0,7223 | 1,59E-06 | 5,77E-05 |
| C3orf39 | NM_032806 | Homo sapiens chromosome 3 open reading frame 39 (C3orf39), mRNA [NM_032806] | -1,5599 | 1,71E-06 | 6,13E-05 |
| A_24_P325533 | A_24_P325533 | Unknown | -0,8002 | 1,75E-06 | 6,27E-05 |
| ENST00000327926 | ENST00000327926 | Homo sapiens isolate donor Z clone Z1K immunoglobulin kappa light chain variable region mRNA, partial cds. [AF103555] | -1,2072 | 1,82E-06 | 6,53E-05 |
| RGS16 | NM_002928 | Homo sapiens regulator of G-protein signalling 16 (RGS16), mRNA [NM_002928] | -1,3262 | 1,85E-06 | 6,60E-05 |
| A_24_P384604 | A_24_P384604 | Unknown | -1,1346 | 1,89E-06 | 6,70E-05 |
| BC039021 | BC039021 | Homo sapiens cDNA clone IMAGE:6043059, partial cds. [BC039021] | -0,9562 | 1,89E-06 | 6,70E-05 |
| TUBA6 | NM_032704 | Homo sapiens tubulin, alpha 6 (TUBA6), mRNA [NM_032704] | -1,2243 | 1,93E-06 | 6,85E-05 |
| IGKC | BC095489 | Homo sapiens immunoglobulin kappa constant, mRNA (cDNA clone MGC:111575 IMAGE:30328747), complete cds. [BC095489] | -2,0351 | 1,94E-06 | 6,85E-05 |
| PARVB | NM_001003828 | Homo sapiens parvin, beta (PARVB), transcript variant 1, mRNA [NM_001003828] | -0,8964 | 1,96E-06 | 6,91E-05 |
| AK1 | NM_000476 | Homo sapiens adenylate kinase 1 (AK1), mRNA [NM_000476] | -1,4463 | 2,03E-06 | 7,14E-05 |
| LOC134357 | XR_018355 | PREDICTED: Homo sapiens similar to aconitase 2 precursor (LOC134357), mRNA [XR_018355] | -0,9504 | 2,16E-06 | 7,57E-05 |
| A_24_P281504 | A_24_P281504 | Unknown | -0,7549 | 2,19E-06 | 7,64E-05 |
| ZYG11BL | NM_006336 | Homo sapiens zyg-11 homolog B (C. elegans)-like (ZYG11BL), mRNA [NM_006336] | -1,0488 | 2,19E-06 | 7,64E-05 |
| MBP | NM_001025100 | Homo sapiens myelin basic protein (MBP), transcript variant 8, mRNA [NM_001025100] | -1,0477 | 2,20E-06 | 7,67E-05 |
| WBSCR16 | NM_030798 | Homo sapiens Williams-Beuren syndrome chromosome region 16 (WBSCR16), mRNA [NM_030798] | -0,7585 | 2,24E-06 | 7,79E-05 |
| CDC20 | NM_001255 | Homo sapiens cell division cycle 20 homolog (S. cerevisiae) (CDC20), mRNA [NM_001255] | -0,9446 | 2,32E-06 | 8,02E-05 |
| MTMR12 | NM_001040446 | Homo sapiens myotubularin related protein 12 (MTMR12), mRNA [NM_001040446] | -0,8659 | 2,32E-06 | 8,02E-05 |
| PIP5K1B | NM_003558 | Homo sapiens phosphatidylinositol-4-phosphate 5-kinase, type I, beta (PIP5K1B), transcript variant 2, mRNA [NM_003558] | -1,1184 | 2,33E-06 | 8,04E-05 |
| PIM1 | NM_002648 | Homo sapiens pim-1 oncogene (PIM1), mRNA [NM_002648] | -1,6406 | 2,38E-06 | 8,17E-05 |
| KLHDC8B | NM_173546 | Homo sapiens kelch domain containing 8B (KLHDC8B), mRNA [NM_173546] | -0,8000 | 2,41E-06 | 8,27E-05 |
| ENST00000360102 | ENST00000360102 | Homo sapiens clone CD-27-VH immunoglobulin heavy chain variable region mRNA, partial cds. [AY944711] | -1,8500 | 2,53E-06 | 8,63E-05 |
| CCS | NM_005125 | Homo sapiens copper chaperone for superoxide dismutase (CCS), mRNA [NM_005125] | -1,0156 | 2,54E-06 | 8,65E-05 |
| CCR5 | NM_000579 | Homo sapiens chemokine (C-C motif) receptor 5 (CCR5), mRNA [NM_000579] | -1,0152 | 2,71E-06 | 9,16E-05 |
| AK024898 | AK024898 | Homo sapiens cDNA: FLJ21245 fis, clone COL01184. [AK024898] | -0,7658 | 2,89E-06 | 9,72E-05 |
| LMAN2 | NM_006816 | Homo sapiens lectin, mannose-binding 2 (LMAN2), mRNA [NM_006816] | -0,8196 | 2,93E-06 | 9,82E-05 |
| RAD23A | NM_005053 | Homo sapiens RAD23 homolog A (S. cerevisiae) (RAD23A), mRNA [NM_005053] | -1,3308 | 2,92E-06 | 9,82E-05 |
| FBXO34 | NM_017943 | Homo sapiens F-box protein 34 (FBXO34), mRNA [NM_017943] | -1,1950 | 2,93E-06 | 9,82E-05 |
| UROS | NM_000375 | Homo sapiens uroporphyrinogen III synthase (congenital erythropoietic porphyria) (UROS), mRNA [NM_000375] | -1,0616 | 2,97E-06 | 9,94E-05 |
| BACE2 | NM_012105 | Homo sapiens beta-site APP-cleaving enzyme 2 (BACE2), transcript variant a, mRNA [NM_012105] | -1,0508 | 3,05E-06 | 1,01E-04 |
| ZBTB3 | NM_024784 | Homo sapiens zinc finger and BTB domain containing 3 (ZBTB3), mRNA [NM_024784] | -1,0345 | 3,14E-06 | 1,04E-04 |
| ACO2 | NM_001098 | Homo sapiens aconitase 2, mitochondrial (ACO2), nuclear gene encoding mitochondrial protein, mRNA [NM_001098] | -0,8739 | 3,19E-06 | 1,05E-04 |
| A_24_P341408 | A_24_P341408 | Unknown | -0,7896 | 3,25E-06 | 1,07E-04 |
| UBB | NM_018955 | Homo sapiens ubiquitin B (UBB), mRNA [NM_018955] | -0,8508 | 3,27E-06 | 1,08E-04 |
| C18orf24 | NM_001039535 | Homo sapiens chromosome 18 open reading frame 24 (C18orf24), transcript variant 1, mRNA [NM_001039535] | -0,8438 | 3,34E-06 | 1,10E-04 |
| GPR132 | NM_013345 | Homo sapiens G protein-coupled receptor 132 (GPR132), mRNA [NM_013345] | -1,1860 | 3,35E-06 | 1,10E-04 |
| SEPP1 | NM_005410 | Homo sapiens selenoprotein P, plasma, 1 (SEPP1), mRNA [NM_005410] | -1,4634 | 3,35E-06 | 1,10E-04 |
| X92493 | X92493 | H.sapiens mRNA for STM-7 protein. [X92493] | -0,9851 | 3,39E-06 | 1,11E-04 |
| AY062331 | AY062331 | Homo sapiens clone 105/707 immunoglobulin light chain kappa variable region mRNA, partial cds. [AY062331] | -1,1969 | 3,47E-06 | 1,13E-04 |
| AK026372 | AK026372 | Homo sapiens cDNA: FLJ22719 fis, clone HSI14307. [AK026372] | -0,8259 | 3,52E-06 | 1,14E-04 |
| LHFPL2 | NM_005779 | Homo sapiens lipoma HMGIC fusion partner-like 2 (LHFPL2), mRNA [NM_005779] | -1,0080 | 3,56E-06 | 1,15E-04 |
| GCDH | NM_013976 | Homo sapiens glutaryl-Coenzyme A dehydrogenase (GCDH), nuclear gene encoding mitochondrial protein, transcript variant 2, mRNA [NM_013976] | -0,7198 | 3,57E-06 | 1,16E-04 |
| PRPF19 | NM_014502 | Homo sapiens PRP19/PSO4 pre-mRNA processing factor 19 homolog (S. cerevisiae) (PRPF19), mRNA [NM_014502] | -0,8896 | 3,59E-06 | 1,16E-04 |
| PRKAR2B | NM_002736 | Homo sapiens protein kinase, cAMP-dependent, regulatory, type II, beta (PRKAR2B), mRNA [NM_002736] | -1,1536 | 3,61E-06 | 1,16E-04 |
| ZNF526 | NM_133444 | Homo sapiens zinc finger protein 526 (ZNF526), mRNA [NM_133444] | -0,8453 | 3,78E-06 | 1,21E-04 |
| IFT122 | NM_018262 | Homo sapiens intraflagellar transport 122 homolog (Chlamydomonas) (IFT122), transcript variant 3, mRNA [NM_018262] | -0,5861 | 3,79E-06 | 1,21E-04 |
| HPS6 | NM_024747 | Homo sapiens Hermansky-Pudlak syndrome 6 (HPS6), mRNA [NM_024747] | -1,5128 | 3,82E-06 | 1,22E-04 |
| SLC37A4 | NM_001467 | Homo sapiens solute carrier family 37 (glycerol-6-phosphate transporter), member 4 (SLC37A4), mRNA [NM_001467] | -0,8976 | 3,83E-06 | 1,22E-04 |
| LMNA | NM_005572 | Homo sapiens lamin A/C (LMNA), transcript variant 2, mRNA [NM_005572] | -1,5170 | 3,91E-06 | 1,24E-04 |
| NUDT4 | NM_199040 | Homo sapiens nudix (nucleoside diphosphate linked moiety X)-type motif 4 (NUDT4), transcript variant 2, mRNA [NM_199040] | -1,3340 | 3,91E-06 | 1,24E-04 |
| QSCN6L1 | NM_181701 | Homo sapiens quiescin Q6-like 1 (QSCN6L1), mRNA [NM_181701] | -0,8392 | 3,92E-06 | 1,24E-04 |
| PROP1 | NM_006261 | Homo sapiens prophet of Pit1, paired-like homeodomain transcription factor (PROP1), mRNA [NM_006261] | -1,4685 | 3,96E-06 | 1,25E-04 |
| ENST00000322032 | ENST00000322032 | Ig alpha-2 chain C region. [Source:Uniprot/SWISSPROT;Acc:P01877] [ENST00000322032] | -3,1402 | 4,08E-06 | 1,28E-04 |
| PSMF1 | NM_006814 | Homo sapiens proteasome (prosome, macropain) inhibitor subunit 1 (PI31) (PSMF1), transcript variant 1, mRNA [NM_006814] | -1,2587 | 4,08E-06 | 1,28E-04 |
| E2F4 | NM_001950 | Homo sapiens E2F transcription factor 4, p107/p130-binding (E2F4), mRNA [NM_001950] | -1,2621 | 4,09E-06 | 1,28E-04 |
| RGS10 | NM_001005339 | Homo sapiens regulator of G-protein signalling 10 (RGS10), transcript variant 1, mRNA [NM_001005339] | -1,0505 | 4,30E-06 | 1,34E-04 |
| METAP2 | NM_006838 | Homo sapiens methionyl aminopeptidase 2 (METAP2), mRNA [NM_006838] | -0,8993 | 4,31E-06 | 1,34E-04 |
| FHL2 | NM_001039492 | Homo sapiens four and a half LIM domains 2 (FHL2), transcript variant 5, mRNA [NM_001039492] | -1,6918 | 4,66E-06 | 1,45E-04 |
| RPIA | NM_144563 | Homo sapiens ribose 5-phosphate isomerase A (ribose 5-phosphate epimerase) (RPIA), mRNA [NM_144563] | -1,2934 | 4,76E-06 | 1,47E-04 |
| RNF121 | NM_018320 | Homo sapiens ring finger protein 121 (RNF121), transcript variant 1, mRNA [NM_018320] | -0,5128 | 4,80E-06 | 1,48E-04 |
| X01147 | X01147 | Human mRNA for immunoglobulin lambda variable region corresponding to NEW protein of V lambda subgroup I. [X01147] | -1,6493 | 4,80E-06 | 1,48E-04 |
| C9orf5 | NM_032012 | Homo sapiens chromosome 9 open reading frame 5 (C9orf5), mRNA [NM_032012] | -1,1033 | 4,95E-06 | 1,53E-04 |
| ICAM4 | NM_001544 | Homo sapiens intercellular adhesion molecule 4 (Landsteiner-Wiener blood group) (ICAM4), transcript variant 1, mRNA [NM_001544] | -0,8435 | 5,00E-06 | 1,54E-04 |
| ALG2 | NM_033087 | Homo sapiens asparagine-linked glycosylation 2 homolog (S. cerevisiae, alpha-1,3-mannosyltransferase) (ALG2), mRNA [NM_033087] | -0,6102 | 5,14E-06 | 1,58E-04 |
| CDKN2C | NM_078626 | Homo sapiens cyclin-dependent kinase inhibitor 2C (p18, inhibits CDK4) (CDKN2C), transcript variant 2, mRNA [NM_078626] | -0,9901 | 5,23E-06 | 1,60E-04 |
| CDH1 | NM_004360 | Homo sapiens cadherin 1, type 1, E-cadherin (epithelial) (CDH1), mRNA [NM_004360] | -1,2910 | 5,46E-06 | 1,65E-04 |
| FUT1 | NM_000148 | Homo sapiens fucosyltransferase 1 (galactoside 2-alpha-L-fucosyltransferase, H blood group) (FUT1), mRNA [NM_000148] | -1,0918 | 5,46E-06 | 1,65E-04 |
| TMEM48 | AK091439 | Homo sapiens cDNA FLJ34120 fis, clone FCBBF3009541. [AK091439] | -0,7672 | 5,72E-06 | 1,72E-04 |
| BRD3 | NM_007371 | Homo sapiens bromodomain containing 3 (BRD3), mRNA [NM_007371] | -1,4448 | 5,74E-06 | 1,73E-04 |
| SDSL | NM_138432 | Homo sapiens serine dehydratase-like (SDSL), mRNA [NM_138432] | -0,8064 | 5,79E-06 | 1,74E-04 |
| GNA12 | NM_007353 | Homo sapiens guanine nucleotide binding protein (G protein) alpha 12 (GNA12), mRNA [NM_007353] | -1,0243 | 5,83E-06 | 1,75E-04 |
| MGC11102 | NM_032325 | Homo sapiens hypothetical protein MGC11102 (MGC11102), mRNA [NM_032325] | -0,7255 | 5,96E-06 | 1,78E-04 |
| HEXA | NM_000520 | Homo sapiens hexosaminidase A (alpha polypeptide) (HEXA), mRNA [NM_000520] | -0,8502 | 6,05E-06 | 1,80E-04 |
| C1orf26 | NM_017673 | Homo sapiens chromosome 1 open reading frame 26 (C1orf26), mRNA [NM_017673] | -0,7772 | 6,09E-06 | 1,81E-04 |
| CLTC | NM_004859 | Homo sapiens clathrin, heavy chain (Hc) (CLTC), mRNA [NM_004859] | -1,2217 | 6,17E-06 | 1,83E-04 |
| PIP5K2A | ENST00000376573 | Phosphatidylinositol-4-phosphate 5-kinase type-2 alpha (EC 2.7.1.68) (Phosphatidylinositol-4-phosphate 5-kinase type II alpha) (1- phosphatidylinositol-4-phosphate 5-kinase 2-alpha) (PtdIns(4)P-5- kinase isoform 2-alpha) (PIP5KII-alpha)... | -1,3787 | 6,18E-06 | 1,83E-04 |
| C10orf12 | AK025166 | Homo sapiens cDNA: FLJ21513 fis, clone COL05778. [AK025166] | -1,3206 | 6,21E-06 | 1,84E-04 |
| COMT | NM_000754 | Homo sapiens catechol-O-methyltransferase (COMT), transcript variant MB-COMT, mRNA [NM_000754] | -0,7944 | 6,30E-06 | 1,85E-04 |
| AIFM2 | NM_032797 | Homo sapiens apoptosis-inducing factor, mitochondrion-associated, 2 (AIFM2), mRNA [NM_032797] | -0,7152 | 6,55E-06 | 1,91E-04 |
| LOC642413 | XR_016155 | PREDICTED: Homo sapiens similar to Cathepsin L precursor (Major excreted protein) (MEP) (LOC642413), mRNA [XR_016155] | -0,9867 | 6,54E-06 | 1,91E-04 |
| FN3KRP | NM_024619 | Homo sapiens fructosamine-3-kinase-related protein (FN3KRP), mRNA [NM_024619] | -0,7646 | 6,84E-06 | 1,98E-04 |
| RAP2B | NM_002886 | Homo sapiens RAP2B, member of RAS oncogene family (RAP2B), mRNA [NM_002886] | -0,5674 | 6,97E-06 | 2,01E-04 |
| VCP | NM_007126 | Homo sapiens valosin-containing protein (VCP), mRNA [NM_007126] | -0,6940 | 7,17E-06 | 2,07E-04 |
| EB386378 | EB386378 | nbj15e01.y1 Human optic nerve. Unnormalized (nbj) Homo sapiens cDNA clone nbj15e01 5', mRNA sequence [EB386378] | -0,6121 | 7,29E-06 | 2,10E-04 |
| C17orf71 | NM_018149 | Homo sapiens chromosome 17 open reading frame 71 (C17orf71), mRNA [NM_018149] | -0,8348 | 7,32E-06 | 2,10E-04 |
| CTA-246H3.1 | NM_001013618 | Homo sapiens similar to omega protein (LOC91353), mRNA [NM_001013618] | -1,6057 | 7,46E-06 | 2,12E-04 |
| C20orf121 | NM_024331 | Homo sapiens chromosome 20 open reading frame 121 (C20orf121), transcript variant 1, mRNA [NM_024331] | -1,2496 | 7,55E-06 | 2,15E-04 |
| FAM122A | NM_138333 | Homo sapiens family with sequence similarity 122A (FAM122A), mRNA [NM_138333] | -0,7963 | 7,60E-06 | 2,15E-04 |
| CR617018 | CR617018 | full-length cDNA clone CS0DG001YH13 of B cells (Ramos cell line) of Homo sapiens (human). [CR617018] | -0,8252 | 7,74E-06 | 2,18E-04 |
| TCEA1 | NM_006756 | Homo sapiens transcription elongation factor A (SII), 1 (TCEA1), transcript variant 1, mRNA [NM_006756] | -0,7917 | 7,85E-06 | 2,21E-04 |
| IFI6 | BC024289 | Homo sapiens interferon, alpha-inducible protein 6, mRNA (cDNA clone MGC:39273 IMAGE:5440834), complete cds. [BC024289] | -3,0151 | 7,91E-06 | 2,22E-04 |
| AJ009817 | AJ009817 | Homo sapiens mRNA for AMMECR1 protein, alternative exon 2. [AJ009817] | -1,0961 | 7,96E-06 | 2,23E-04 |
| AJ399872 | AJ399872 | Homo sapiens partial mRNA for thyroid peroxidase-specific immunoglobulin kappa chain variable egion (IGKV gene), clone T2. [AJ399872] | -0,7866 | 8,38E-06 | 2,33E-04 |
| LOC652254 | XR_019518 | PREDICTED: Homo sapiens similar to 60S ribosomal protein L8 (LOC652254), mRNA [XR_019518] | -1,1401 | 8,41E-06 | 2,33E-04 |
| FARSLA | NM_004461 | Homo sapiens phenylalanine-tRNA synthetase-like, alpha subunit (FARSLA), mRNA [NM_004461] | -0,5511 | 8,48E-06 | 2,35E-04 |
| POLR1C | NM_004875 | Homo sapiens polymerase (RNA) I polypeptide C, 30kDa (POLR1C), transcript variant 2, mRNA [NM_004875] | -0,7323 | 8,59E-06 | 2,37E-04 |
| ZFYVE21 | NM_024071 | Homo sapiens zinc finger, FYVE domain containing 21 (ZFYVE21), mRNA [NM_024071] | -1,4240 | 8,58E-06 | 2,37E-04 |
| BC012876 | BC012876 | Homo sapiens cDNA clone MGC:17259 IMAGE:4149333, complete cds. [BC012876] | -2,0322 | 8,66E-06 | 2,38E-04 |
| VDAC3 | NM_005662 | Homo sapiens voltage-dependent anion channel 3 (VDAC3), mRNA [NM_005662] | -0,6137 | 8,75E-06 | 2,40E-04 |
| ICMT | NM_012405 | Homo sapiens isoprenylcysteine carboxyl methyltransferase (ICMT), mRNA [NM_012405] | -0,8928 | 9,36E-06 | 2,55E-04 |
| TMED10 | NM_006827 | Homo sapiens transmembrane emp24-like trafficking protein 10 (yeast) (TMED10), mRNA [NM_006827] | -0,8981 | 9,35E-06 | 2,55E-04 |
| DCK | NM_000788 | Homo sapiens deoxycytidine kinase (DCK), mRNA [NM_000788] | -1,1868 | 9,43E-06 | 2,56E-04 |
| HCCA2 | NM_053005 | Homo sapiens HCCA2 protein (HCCA2), mRNA [NM_053005] | -0,6901 | 9,47E-06 | 2,57E-04 |
| TTLL12 | NM_015140 | Homo sapiens tubulin tyrosine ligase-like family, member 12 (TTLL12), mRNA [NM_015140] | -0,9382 | 9,48E-06 | 2,57E-04 |
| X57802 | X57802 | Human rearranged immunoglobulin lambda light chain mRNA. [X57802] | -2,1480 | 9,59E-06 | 2,60E-04 |
| KLHDC3 | NM_057161 | Homo sapiens kelch domain containing 3 (KLHDC3), mRNA [NM_057161] | -0,6874 | 9,63E-06 | 2,61E-04 |
| HDLBP | NM_005336 | Homo sapiens high density lipoprotein binding protein (vigilin) (HDLBP), mRNA [NM_005336] | -0,7212 | 9,66E-06 | 2,61E-04 |
| AGPAT3 | NM_020132 | Homo sapiens 1-acylglycerol-3-phosphate O-acyltransferase 3 (AGPAT3), transcript variant 1, mRNA [NM_020132] | -1,0531 | 9,85E-06 | 2,65E-04 |
| APOBEC3C | NM_014508 | Homo sapiens apolipoprotein B mRNA editing enzyme, catalytic polypeptide-like 3C (APOBEC3C), mRNA [NM_014508] | -0,7083 | 9,94E-06 | 2,67E-04 |
| ANKRD33 | NM_182608 | Homo sapiens ankyrin repeat domain 33 (ANKRD33), mRNA [NM_182608] | -1,4257 | 1,00E-05 | 2,69E-04 |
| RASIP1 | NM_017805 | Homo sapiens Ras interacting protein 1 (RASIP1), mRNA [NM_017805] | -1,0308 | 1,00E-05 | 2,69E-04 |
| L38427 | L38427 | Homo sapiens Ig rearranged H-chain mRNA V region. [L38427] | -1,4000 | 1,01E-05 | 2,70E-04 |
| PLEKHH3 | NM_024927 | Homo sapiens pleckstrin homology domain containing, family H (with MyTH4 domain) member 3 (PLEKHH3), mRNA [NM_024927] | -1,1792 | 1,01E-05 | 2,70E-04 |
| BC022362 | BC022362 | Homo sapiens cDNA clone MGC:23888 IMAGE:4704496, complete cds. [BC022362] | -0,7430 | 1,06E-05 | 2,81E-04 |
| D83692 | D83692 | Human mRNA for immunoglobulin M (IgM), partial cds (VH3-N-D-N-JH4), clone H2-39E. [D83692] | -0,9594 | 1,06E-05 | 2,81E-04 |
| SLC2A4RG | NM_020062 | Homo sapiens SLC2A4 regulator (SLC2A4RG), mRNA [NM_020062] | -0,8911 | 1,06E-05 | 2,81E-04 |
| A_23_P435390 | A_23_P435390 | Unknown | -2,0916 | 1,06E-05 | 2,81E-04 |
| C9orf40 | NM_017998 | Homo sapiens chromosome 9 open reading frame 40 (C9orf40), mRNA [NM_017998] | -1,2447 | 1,06E-05 | 2,81E-04 |
| MOBKL2C | NM_145279 | Homo sapiens MOB1, Mps One Binder kinase activator-like 2C (yeast) (MOBKL2C), transcript variant 1, mRNA [NM_145279] | -0,7734 | 1,07E-05 | 2,81E-04 |
| LOC442308 | XR_018043 | PREDICTED: Homo sapiens similar to tubulin, beta 5 (LOC442308), mRNA [XR_018043] | -0,9589 | 1,07E-05 | 2,82E-04 |
| CALM3 | NM_005184 | Homo sapiens calmodulin 3 (phosphorylase kinase, delta) (CALM3), mRNA [NM_005184] | -0,7466 | 1,09E-05 | 2,85E-04 |
| DOHH | NM_031304 | Homo sapiens deoxyhypusine hydroxylase/monooxygenase (DOHH), mRNA [NM_031304] | -0,8944 | 1,09E-05 | 2,85E-04 |
| HK1 | NM_033500 | Homo sapiens hexokinase 1 (HK1), nuclear gene encoding mitochondrial protein, transcript variant 5, mRNA [NM_033500] | -1,1505 | 1,09E-05 | 2,85E-04 |
| GYPE | NM_002102 | Homo sapiens glycophorin E (GYPE), transcript variant 1, mRNA [NM_002102] | -1,3091 | 1,10E-05 | 2,87E-04 |
| AK074614 | AK074614 | Homo sapiens cDNA FLJ90133 fis, clone HEMBB1000567. [AK074614] | -2,0007 | 1,15E-05 | 2,97E-04 |
| HTRA2 | NM_145074 | Homo sapiens HtrA serine peptidase 2 (HTRA2), nuclear gene encoding mitochondrial protein, transcript variant 2, mRNA [NM_145074] | -0,7177 | 1,14E-05 | 2,97E-04 |
| IQWD1 | NM_018442 | Homo sapiens IQ motif and WD repeats 1 (IQWD1), transcript variant 1, mRNA [NM_018442] | -0,7834 | 1,17E-05 | 3,03E-04 |
| MST1 | NM_020998 | Homo sapiens macrophage stimulating 1 (hepatocyte growth factor-like) (MST1), mRNA [NM_020998] | -1,1405 | 1,17E-05 | 3,03E-04 |
| NDUFS2 | NM_004550 | Homo sapiens NADH dehydrogenase (ubiquinone) Fe-S protein 2, 49kDa (NADH-coenzyme Q reductase) (NDUFS2), mRNA [NM_004550] | -0,6566 | 1,19E-05 | 3,06E-04 |
| AJ319669 | AJ319669 | Homo sapiens germline mRNA for immunoglobulin lambda-2 chain constant region, Daudi cell line. [AJ319669] | -1,4378 | 1,20E-05 | 3,08E-04 |
| SRXN1 | NM_080725 | Homo sapiens sulfiredoxin 1 homolog (S. cerevisiae) (SRXN1), mRNA [NM_080725] | -1,3276 | 1,21E-05 | 3,11E-04 |
| C6orf59 | AK026765 | Homo sapiens cDNA: FLJ23112 fis, clone LNG07874. [AK026765] | -1,1261 | 1,22E-05 | 3,13E-04 |
| EPB41 | NM_004437 | Homo sapiens erythrocyte membrane protein band 4.1 (elliptocytosis 1, RH-linked) (EPB41), transcript variant 3, mRNA [NM_004437] | -1,0513 | 1,22E-05 | 3,13E-04 |
| CAPN1 | NM_005186 | Homo sapiens calpain 1, (mu/I) large subunit (CAPN1), mRNA [NM_005186] | -0,8808 | 1,24E-05 | 3,15E-04 |
| DERL3 | NM_198440 | Homo sapiens Der1-like domain family, member 3 (DERL3), transcript variant 1, mRNA [NM_198440] | -1,1057 | 1,24E-05 | 3,15E-04 |
| DNAJB2 | NM_006736 | Homo sapiens DnaJ (Hsp40) homolog, subfamily B, member 2 (DNAJB2), transcript variant 2, mRNA [NM_006736] | -1,0491 | 1,24E-05 | 3,15E-04 |
| PPT2 | NM_005155 | Homo sapiens palmitoyl-protein thioesterase 2 (PPT2), transcript variant 1, mRNA [NM_005155] | -1,0928 | 1,23E-05 | 3,15E-04 |
| SLC30A1 | ENST00000367001 | Zinc transporter 1 (ZnT-1) (Solute carrier family 30 member 1). [Source:Uniprot/SWISSPROT;Acc:Q9Y6M5] [ENST00000367001] | -1,3561 | 1,25E-05 | 3,17E-04 |
| M87790 | M87790 | Human (hybridoma H210) anti-hepatitis A immunoglobulin lambda chain variable region, constant region, complementarity-determining regions mRNA, complete cds. [M87790] | -1,6280 | 1,27E-05 | 3,21E-04 |
| DCUN1D1 | AF292100 | Homo sapiens RP42 protein mRNA, complete cds. [AF292100] | -1,1346 | 1,30E-05 | 3,28E-04 |
| LRRN5 | NM_201630 | Homo sapiens leucine rich repeat neuronal 5 (LRRN5), transcript variant 2, mRNA [NM_201630] | -1,5218 | 1,31E-05 | 3,28E-04 |
| ARL2 | NM_001667 | Homo sapiens ADP-ribosylation factor-like 2 (ARL2), mRNA [NM_001667] | -0,6466 | 1,33E-05 | 3,33E-04 |
| ZNF264 | NM_003417 | Homo sapiens zinc finger protein 264 (ZNF264), mRNA [NM_003417] | -0,6347 | 1,40E-05 | 3,48E-04 |
| CLCN3 | NM_173872 | Homo sapiens chloride channel 3 (CLCN3), transcript variant e, mRNA [NM_173872] | -0,7071 | 1,41E-05 | 3,49E-04 |
| SEC24C | NM_004922 | Homo sapiens SEC24 related gene family, member C (S. cerevisiae) (SEC24C), transcript variant 1, mRNA [NM_004922] | -0,5656 | 1,41E-05 | 3,50E-04 |
| FZR1 | NM_016263 | Homo sapiens fizzy/cell division cycle 20 related 1 (Drosophila) (FZR1), mRNA [NM_016263] | -0,8092 | 1,43E-05 | 3,53E-04 |
| CR605719 | CR605719 | full-length cDNA clone CS0DK002YG10 of HeLa cells Cot 25-normalized of Homo sapiens (human). [CR605719] | -0,8501 | 1,45E-05 | 3,57E-04 |
| ST7 | NM_018412 | Homo sapiens suppression of tumorigenicity 7 (ST7), transcript variant a, mRNA [NM_018412] | -0,7655 | 1,47E-05 | 3,63E-04 |
| LOC645000 | XR_016848 | PREDICTED: Homo sapiens similar to 40S ribosomal protein S3 (LOC645000), mRNA [XR_016848] | -0,7339 | 1,48E-05 | 3,64E-04 |
| USP14 | NM_005151 | Homo sapiens ubiquitin specific peptidase 14 (tRNA-guanine transglycosylase) (USP14), transcript variant 1, mRNA [NM_005151] | -0,8134 | 1,50E-05 | 3,67E-04 |
| GRRP1 | NM_024869 | Homo sapiens glycine/arginine rich protein 1 (GRRP1), mRNA [NM_024869] | -0,7848 | 1,56E-05 | 3,80E-04 |
| ZDHHC5 | NM_015457 | Homo sapiens zinc finger, DHHC-type containing 5 (ZDHHC5), mRNA [NM_015457] | -0,6515 | 1,56E-05 | 3,80E-04 |
| C22orf9 | NM_015264 | Homo sapiens chromosome 22 open reading frame 9 (C22orf9), transcript variant 1, mRNA [NM_015264] | -0,7818 | 1,56E-05 | 3,80E-04 |
| LOC643992 | XR_018270 | PREDICTED: Homo sapiens hypothetical LOC643992 (LOC643992), mRNA [XR_018270] | -0,7929 | 1,57E-05 | 3,82E-04 |
| A_23_P44053 | A_23_P44053 | Unknown | -1,4845 | 1,58E-05 | 3,83E-04 |
| SACS | NM_014363 | Homo sapiens spastic ataxia of Charlevoix-Saguenay (sacsin) (SACS), mRNA [NM_014363] | -1,0106 | 1,59E-05 | 3,86E-04 |
| BCL2L11 | NM_138621 | Homo sapiens BCL2-like 11 (apoptosis facilitator) (BCL2L11), transcript variant 1, mRNA [NM_138621] | -1,1945 | 1,60E-05 | 3,88E-04 |
| FOXM1 | NM_202002 | Homo sapiens forkhead box M1 (FOXM1), transcript variant 1, mRNA [NM_202002] | -0,6280 | 1,68E-05 | 4,02E-04 |
| ENST00000259219 | ENST00000259219 | Homo sapiens clone 63a12 anti-tetanus toxoid immunoglobulin light chain variable region (IGL@) mRNA, partial cds. [AY867113] | -1,3723 | 1,70E-05 | 4,07E-04 |
| Y11328 | Y11328 | H.sapiens mRNA for immunoglobulin heavy chain, partial, clone VH3-7. [Y11328] | -1,4719 | 1,71E-05 | 4,08E-04 |
| A_24_P490109 | A_24_P490109 | Unknown | -1,5535 | 1,72E-05 | 4,08E-04 |
| ENST00000295339 | ENST00000295339 | Ig kappa chain V-III region VH precursor (Fragment). [Source:Uniprot/SWISSPROT;Acc:P04434] [ENST00000295339] | -1,4578 | 1,72E-05 | 4,08E-04 |
| SHARPIN | NM_030974 | Homo sapiens SHANK-associated RH domain interactor (SHARPIN), mRNA [NM_030974] | -0,8638 | 1,72E-05 | 4,08E-04 |
| WHSC1 | NM_133330 | Homo sapiens Wolf-Hirschhorn syndrome candidate 1 (WHSC1), transcript variant 1, mRNA [NM_133330] | -0,8634 | 1,72E-05 | 4,08E-04 |
| ABCF2 | NM_005692 | Homo sapiens ATP-binding cassette, sub-family F (GCN20), member 2 (ABCF2), nuclear gene encoding mitochondrial protein, transcript variant 2, mRNA [NM_005692] | -0,6074 | 1,73E-05 | 4,08E-04 |
| AK023159 | AK023159 | Homo sapiens cDNA FLJ13097 fis, clone NT2RP3002173. [AK023159] | -0,4710 | 1,74E-05 | 4,10E-04 |
| DOLPP1 | NM_020438 | Homo sapiens dolichyl pyrophosphate phosphatase 1 (DOLPP1), mRNA [NM_020438] | -0,6227 | 1,74E-05 | 4,10E-04 |
| GZMK | NM_002104 | Homo sapiens granzyme K (granzyme 3; tryptase II) (GZMK), mRNA [NM_002104] | -1,4395 | 1,75E-05 | 4,12E-04 |
| C22orf28 | NM_014306 | Homo sapiens chromosome 22 open reading frame 28 (C22orf28), mRNA [NM_014306] | -0,6409 | 1,76E-05 | 4,13E-04 |
| TLOC1 | NM_003262 | Homo sapiens translocation protein 1 (TLOC1), mRNA [NM_003262] | -1,1391 | 1,79E-05 | 4,20E-04 |
| RCCD1 | NM_033544 | Homo sapiens RCC1 domain containing 1 (RCCD1), transcript variant 1, mRNA [NM_033544] | -0,7247 | 1,85E-05 | 4,33E-04 |
| IGJ | NM_144646 | Homo sapiens immunoglobulin J polypeptide, linker protein for immunoglobulin alpha and mu polypeptides (IGJ), mRNA [NM_144646] | -1,5554 | 1,89E-05 | 4,40E-04 |
| ENST00000331696 | ENST00000331696 | HUMIGKPB Ig kappa chain {Homo sapiens} (exp=-1; wgp=0; cg=0), complete [THC2557512] | -1,4738 | 1,90E-05 | 4,42E-04 |
| NME4 | NM_005009 | Homo sapiens non-metastatic cells 4, protein expressed in (NME4), mRNA [NM_005009] | -0,9672 | 1,91E-05 | 4,45E-04 |
| FLJ20105 | NM_001009954 | Homo sapiens FLJ20105 protein (FLJ20105), transcript variant 2, mRNA [NM_001009954] | -0,6449 | 1,93E-05 | 4,48E-04 |
| UBXD3 | NM_152376 | Homo sapiens UBX domain containing 3 (UBXD3), mRNA [NM_152376] | -0,9593 | 1,93E-05 | 4,48E-04 |
| C9orf125 | NM_032342 | Homo sapiens chromosome 9 open reading frame 125 (C9orf125), mRNA [NM_032342] | -0,9995 | 2,02E-05 | 4,66E-04 |
| AGPAT4 | NM_020133 | Homo sapiens 1-acylglycerol-3-phosphate O-acyltransferase 4 (lysophosphatidic acid acyltransferase, delta) (AGPAT4), mRNA [NM_020133] | -1,0200 | 2,04E-05 | 4,70E-04 |
| MAP2K3 | NM_145109 | Homo sapiens mitogen-activated protein kinase kinase 3 (MAP2K3), transcript variant B, mRNA [NM_145109] | -1,0742 | 2,07E-05 | 4,75E-04 |
| PDXP | NM_020315 | Homo sapiens pyridoxal (pyridoxine, vitamin B6) phosphatase (PDXP), mRNA [NM_020315] | -0,7433 | 2,09E-05 | 4,78E-04 |
| AF076205 | AF076205 | Homo sapiens rheumatoid arthritis patient C355-6, immunoglobulin lambda light chain variable region (V4b) mRNA, partial cds. [AF076205] | -1,2383 | 2,11E-05 | 4,82E-04 |
| KIAA1344 | NM_020784 | Homo sapiens KIAA1344 (KIAA1344), mRNA [NM_020784] | -0,5743 | 2,11E-05 | 4,82E-04 |
| SHMT1 | NM_004169 | Homo sapiens serine hydroxymethyltransferase 1 (soluble) (SHMT1), transcript variant 1, mRNA [NM_004169] | -0,5584 | 2,11E-05 | 4,82E-04 |
| A_23_P28743 | A_23_P28743 | Unknown | -0,7664 | 2,14E-05 | 4,87E-04 |
| ELOF1 | NM_032377 | Homo sapiens elongation factor 1 homolog (S. cerevisiae) (ELOF1), mRNA [NM_032377] | -0,6400 | 2,13E-05 | 4,87E-04 |
| GCLM | ENST00000370238 | Glutamate--cysteine ligase regulatory subunit (EC 6.3.2.2) (Gamma- glutamylcysteine synthetase) (Gamma-ECS) (GCS light chain) (Glutamate--cysteine ligase modifier subunit). [Source:Uniprot/SWISSPROT;Acc:P48507] [ENST00000370238] | -1,3673 | 2,14E-05 | 4,87E-04 |
| TMEPAI | NM_020182 | Homo sapiens transmembrane, prostate androgen induced RNA (TMEPAI), transcript variant 1, mRNA [NM_020182] | -0,9975 | 2,14E-05 | 4,87E-04 |
| AL522622 | AL522622 | AL522622 AL522622 Homo sapiens NEUROBLASTOMA COT 10-NORMALIZED Homo sapiens cDNA clone CS0DB009YE01 3-PRIME, mRNA sequence [AL522622] | -1,1741 | 2,15E-05 | 4,87E-04 |
| CKAP2 | NM_018204 | Homo sapiens cytoskeleton associated protein 2 (CKAP2), mRNA [NM_018204] | -1,1818 | 2,15E-05 | 4,88E-04 |
| ENST00000355691 | ENST00000355691 | Copper-transporting ATPase 1 (EC 3.6.3.4) (Copper pump 1) (Menkes disease-associated protein). [Source:Uniprot/SWISSPROT;Acc:Q04656] [ENST00000355691] | -0,6396 | 2,16E-05 | 4,88E-04 |
| KCNN4 | NM_002250 | Homo sapiens potassium intermediate/small conductance calcium-activated channel, subfamily N, member 4 (KCNN4), mRNA [NM_002250] | -0,9201 | 2,17E-05 | 4,90E-04 |
| UCP2 | NM_003355 | Homo sapiens uncoupling protein 2 (mitochondrial, proton carrier) (UCP2), nuclear gene encoding mitochondrial protein, mRNA [NM_003355] | -1,3667 | 2,20E-05 | 4,96E-04 |
| BAG1 | NM_004323 | Homo sapiens BCL2-associated athanogene (BAG1), mRNA [NM_004323] | -1,3072 | 2,22E-05 | 4,99E-04 |
| THC2487640 | THC2487640 | Unknown | -1,0106 | 2,22E-05 | 4,99E-04 |
| SHCBP1 | NM_024745 | Homo sapiens SHC SH2-domain binding protein 1 (SHCBP1), mRNA [NM_024745] | -0,9009 | 2,22E-05 | 5,00E-04 |
| MBOAT5 | NM_005768 | Homo sapiens membrane bound O-acyltransferase domain containing 5 (MBOAT5), mRNA [NM_005768] | -0,7802 | 2,24E-05 | 5,02E-04 |
| WDR13 | NM_017883 | Homo sapiens WD repeat domain 13 (WDR13), mRNA [NM_017883] | -0,5803 | 2,28E-05 | 5,09E-04 |
| FAM20B | NM_014864 | Homo sapiens family with sequence similarity 20, member B (FAM20B), mRNA [NM_014864] | -1,3746 | 2,28E-05 | 5,10E-04 |
| EIF3S9 | NM_001037283 | Homo sapiens eukaryotic translation initiation factor 3, subunit 9 eta, 116kDa (EIF3S9), transcript variant 2, mRNA [NM_001037283] | -0,7887 | 2,33E-05 | 5,18E-04 |
| C9orf78 | NM_016520 | Homo sapiens chromosome 9 open reading frame 78 (C9orf78), mRNA [NM_016520] | -0,9563 | 2,33E-05 | 5,19E-04 |
| LRBA | NM_006726 | Homo sapiens LPS-responsive vesicle trafficking, beach and anchor containing (LRBA), mRNA [NM_006726] | -0,8029 | 2,34E-05 | 5,20E-04 |
| C9orf114 | NM_016390 | Homo sapiens chromosome 9 open reading frame 114 (C9orf114), mRNA [NM_016390] | -0,7928 | 2,37E-05 | 5,23E-04 |
| IGH@ | AK130614 | Homo sapiens cDNA FLJ27104 fis, clone SPL04981, highly similar to Ig gamma-2 chain C region. [AK130614] | -3,4070 | 2,44E-05 | 5,37E-04 |
| DECR2 | NM_020664 | Homo sapiens 2,4-dienoyl CoA reductase 2, peroxisomal (DECR2), mRNA [NM_020664] | -0,9994 | 2,47E-05 | 5,42E-04 |
| MPDU1 | NM_004870 | Homo sapiens mannose-P-dolichol utilization defect 1 (MPDU1), mRNA [NM_004870] | -0,8920 | 2,47E-05 | 5,42E-04 |
| PRDX6 | NM_004905 | Homo sapiens peroxiredoxin 6 (PRDX6), mRNA [NM_004905] | -0,9612 | 2,47E-05 | 5,42E-04 |
| TERF2IP | NM_018975 | Homo sapiens telomeric repeat binding factor 2, interacting protein (TERF2IP), mRNA [NM_018975] | -0,9931 | 2,48E-05 | 5,43E-04 |
| ADIPOR1 | NM_015999 | Homo sapiens adiponectin receptor 1 (ADIPOR1), mRNA [NM_015999] | -1,2637 | 2,52E-05 | 5,50E-04 |
| PYCRL | NM_023078 | Homo sapiens pyrroline-5-carboxylate reductase-like (PYCRL), mRNA [NM_023078] | -1,0208 | 2,59E-05 | 5,64E-04 |
| THC2685096 | THC2685096 | Q8HNY5_9NEOP (Q8HNY5) Cytochrome oxidase I (Fragment), partial (9%) [THC2685096] | -0,5708 | 2,59E-05 | 5,65E-04 |
| THC2609092 | THC2609092 | ALU1_HUMAN (P39188) Alu subfamily J sequence contamination warning entry, partial (7%) [THC2609092] | -0,5732 | 2,60E-05 | 5,65E-04 |
| MARCH2 | NM_016496 | Homo sapiens membrane-associated ring finger (C3HC4) 2 (MARCH2), transcript variant 1, mRNA [NM_016496] | -0,9191 | 2,65E-05 | 5,75E-04 |
| E2F7 | NM_203394 | Homo sapiens E2F transcription factor 7 (E2F7), mRNA [NM_203394] | -0,9438 | 2,65E-05 | 5,75E-04 |
| MOSPD1 | NM_019556 | Homo sapiens motile sperm domain containing 1 (MOSPD1), mRNA [NM_019556] | -0,8630 | 2,68E-05 | 5,81E-04 |
| GRPEL2 | NM_152407 | Homo sapiens GrpE-like 2, mitochondrial (E. coli) (GRPEL2), nuclear gene encoding mitochondrial protein, mRNA [NM_152407] | -0,7667 | 2,86E-05 | 6,15E-04 |
| A_24_P384119 | A_24_P384119 | Unknown | -1,2392 | 2,92E-05 | 6,26E-04 |
| C1orf109 | NM_017850 | Homo sapiens chromosome 1 open reading frame 109 (C1orf109), mRNA [NM_017850] | -0,8042 | 2,94E-05 | 6,30E-04 |
| ABHD14A | NM_015407 | Homo sapiens abhydrolase domain containing 14A (ABHD14A), mRNA [NM_015407] | -0,8472 | 2,98E-05 | 6,38E-04 |
| CR606637 | CR606637 | full-length cDNA clone CS0DI007YA21 of Placenta Cot 25-normalized of Homo sapiens (human). [CR606637] | -0,6690 | 3,02E-05 | 6,44E-04 |
| BQ017638 | BQ017638 | BQ017638 UI-H-DI0-auv-p-03-0-UI.s1 NCI_CGAP_DI0 Homo sapiens cDNA clone IMAGE:5875058 3', mRNA sequence [BQ017638] | -0,8416 | 3,03E-05 | 6,44E-04 |
| TUBB4 | NM_006087 | Homo sapiens tubulin, beta 4 (TUBB4), mRNA [NM_006087] | -0,8009 | 3,09E-05 | 6,55E-04 |
| TF | NM_001063 | Homo sapiens transferrin (TF), mRNA [NM_001063] | -1,7752 | 3,11E-05 | 6,59E-04 |
| TUBB6 | NM_032525 | Homo sapiens tubulin, beta 6 (TUBB6), mRNA [NM_032525] | -0,6946 | 3,11E-05 | 6,59E-04 |
| THC2649313 | THC2649313 | Unknown | -0,6628 | 3,12E-05 | 6,60E-04 |
| KIAA1450 | AK057981 | Homo sapiens cDNA FLJ25252 fis, clone STM03814. [AK057981] | -0,5355 | 3,14E-05 | 6,63E-04 |
| PLEKHF1 | NM_024310 | Homo sapiens pleckstrin homology domain containing, family F (with FYVE domain) member 1 (PLEKHF1), mRNA [NM_024310] | -1,1688 | 3,17E-05 | 6,67E-04 |
| ZSCAN21 | NM_145914 | Homo sapiens zinc finger and SCAN domain containing 21 (ZSCAN21), mRNA [NM_145914] | -0,4227 | 3,16E-05 | 6,67E-04 |
| ACSBG1 | NM_015162 | Homo sapiens acyl-CoA synthetase bubblegum family member 1 (ACSBG1), mRNA [NM_015162] | -0,5820 | 3,22E-05 | 6,77E-04 |
| A_32_P214565 | A_32_P214565 | Unknown | -0,8318 | 3,23E-05 | 6,79E-04 |
| A_24_P349869 | A_24_P349869 | Unknown | -0,9277 | 3,25E-05 | 6,81E-04 |
| CAT | NM_001752 | Homo sapiens catalase (CAT), mRNA [NM_001752] | -1,0308 | 3,27E-05 | 6,85E-04 |
| STK11 | NM_000455 | Homo sapiens serine/threonine kinase 11 (STK11), mRNA [NM_000455] | -0,8713 | 3,29E-05 | 6,88E-04 |
| ENST00000252134 | ENST00000252134 | Uncharacterized protein KIAA0819. [Source:Uniprot/SWISSPROT;Acc:O94909] [ENST00000252134] | -0,9188 | 3,30E-05 | 6,88E-04 |
| RFESD | NM_173362 | Homo sapiens Rieske (Fe-S) domain containing (RFESD), mRNA [NM_173362] | -1,3005 | 3,34E-05 | 6,96E-04 |
| SLC22A4 | NM_003059 | Homo sapiens solute carrier family 22 (organic cation transporter), member 4 (SLC22A4), mRNA [NM_003059] | -0,9680 | 3,40E-05 | 7,04E-04 |
| C14orf169 | NM_024644 | Homo sapiens chromosome 14 open reading frame 169 (C14orf169), mRNA [NM_024644] | -0,9627 | 3,40E-05 | 7,05E-04 |
| BM479752 | BM479752 | AGENCOURT_6465050 NIH_MGC_92 Homo sapiens cDNA clone IMAGE:5577282 5', mRNA sequence [BM479752] | -0,5308 | 3,51E-05 | 7,24E-04 |
| DIAPH3 | NM_001042517 | Homo sapiens diaphanous homolog 3 (Drosophila) (DIAPH3), transcript variant 1, mRNA [NM_001042517] | -0,7039 | 3,51E-05 | 7,24E-04 |
| FKBP1B | NM_054033 | Homo sapiens FK506 binding protein 1B, 12.6 kDa (FKBP1B), transcript variant 2, mRNA [NM_054033] | -0,8094 | 3,59E-05 | 7,39E-04 |
| PPAP2B | NM_003713 | Homo sapiens phosphatidic acid phosphatase type 2B (PPAP2B), transcript variant 1, mRNA [NM_003713] | -0,9132 | 3,59E-05 | 7,39E-04 |
| RFX2 | NM_000635 | Homo sapiens regulatory factor X, 2 (influences HLA class II expression) (RFX2), transcript variant 1, mRNA [NM_000635] | -0,7209 | 3,60E-05 | 7,39E-04 |
| THC2506002 | THC2506002 | Unknown | -0,5876 | 3,63E-05 | 7,45E-04 |
| C5orf30 | NM_033211 | Homo sapiens chromosome 5 open reading frame 30 (C5orf30), mRNA [NM_033211] | -1,0517 | 3,70E-05 | 7,57E-04 |
| KREMEN1 | NM_001039570 | Homo sapiens kringle containing transmembrane protein 1 (KREMEN1), transcript variant 3, mRNA [NM_001039570] | -1,0447 | 3,73E-05 | 7,63E-04 |
| SOD1 | NM_000454 | Homo sapiens superoxide dismutase 1, soluble (amyotrophic lateral sclerosis 1 (adult)) (SOD1), mRNA [NM_000454] | -0,7211 | 3,75E-05 | 7,65E-04 |
| OAT | NM_000274 | Homo sapiens ornithine aminotransferase (gyrate atrophy) (OAT), nuclear gene encoding mitochondrial protein, mRNA [NM_000274] | -0,9175 | 3,77E-05 | 7,69E-04 |
| TIMM44 | NM_006351 | Homo sapiens translocase of inner mitochondrial membrane 44 homolog (yeast) (TIMM44), mRNA [NM_006351] | -0,7716 | 3,88E-05 | 7,89E-04 |
| MCM5 | NM_006739 | Homo sapiens MCM5 minichromosome maintenance deficient 5, cell division cycle 46 (S. cerevisiae) (MCM5), mRNA [NM_006739] | -0,6925 | 3,93E-05 | 7,98E-04 |
| A_24_P814246 | A_24_P814246 | Unknown | -0,7379 | 3,99E-05 | 8,05E-04 |
| AFG3L2 | NM_006796 | Homo sapiens AFG3 ATPase family gene 3-like 2 (yeast) (AFG3L2), nuclear gene encoding mitochondrial protein, mRNA [NM_006796] | -0,6737 | 3,99E-05 | 8,05E-04 |
| AL117621 | AL117621 | Homo sapiens mRNA; cDNA DKFZp564M0264 (from clone DKFZp564M0264). [AL117621] | -0,8212 | 3,98E-05 | 8,05E-04 |
| SAMM50 | NM_015380 | Homo sapiens sorting and assembly machinery component 50 homolog (S. cerevisiae) (SAMM50), mRNA [NM_015380] | -0,8454 | 4,02E-05 | 8,10E-04 |
| A_24_P194954 | A_24_P194954 | Unknown | -0,5899 | 4,09E-05 | 8,23E-04 |
| THC2672257 | THC2672257 | Unknown | -0,5936 | 4,09E-05 | 8,23E-04 |
| DQ100840 | DQ100840 | Homo sapiens isolate N1553H immunoglobulin heavy chain variable region (IGHV3-21) mRNA, IGHV3-21*01 allele, partial cds. [DQ100840] | -1,3565 | 4,11E-05 | 8,25E-04 |
| IGLL1 | NM_020070 | Homo sapiens immunoglobulin lambda-like polypeptide 1 (IGLL1), transcript variant 1, mRNA [NM_020070] | -1,4578 | 4,20E-05 | 8,41E-04 |
| RNF10 | NM_014868 | Homo sapiens ring finger protein 10 (RNF10), mRNA [NM_014868] | -0,9213 | 4,25E-05 | 8,51E-04 |
| AP2B1 | NM_001030006 | Homo sapiens adaptor-related protein complex 2, beta 1 subunit (AP2B1), transcript variant 1, mRNA [NM_001030006] | -0,7783 | 4,39E-05 | 8,75E-04 |
| RPP38 | NM_183005 | Homo sapiens ribonuclease P/MRP 38kDa subunit (RPP38), transcript variant 1, mRNA [NM_183005] | -0,7332 | 4,40E-05 | 8,75E-04 |
| FNBP1L | NM_001024948 | Homo sapiens formin binding protein 1-like (FNBP1L), transcript variant 1, mRNA [NM_001024948] | -0,6938 | 4,41E-05 | 8,76E-04 |
| C1orf164 | NM_018150 | Homo sapiens chromosome 1 open reading frame 164 (C1orf164), mRNA [NM_018150] | -0,6050 | 4,42E-05 | 8,79E-04 |
| RP11-529I10.4 | NM_015448 | Homo sapiens deleted in a mouse model of primary ciliary dyskinesia (RP11-529I10.4), mRNA [NM_015448] | -0,7236 | 4,46E-05 | 8,85E-04 |
| TH1L | NM_198976 | Homo sapiens TH1-like (Drosophila) (TH1L), transcript variant 1, mRNA [NM_198976] | -0,5779 | 4,49E-05 | 8,89E-04 |
| ITSN1 | NM_001001132 | Homo sapiens intersectin 1 (SH3 domain protein) (ITSN1), transcript variant 2, mRNA [NM_001001132] | -0,7427 | 4,51E-05 | 8,93E-04 |
| MGC5139 | BC004815 | Homo sapiens hypothetical protein MGC5139, mRNA (cDNA clone IMAGE:3448346), complete cds. [BC004815] | -0,6709 | 4,54E-05 | 8,98E-04 |
| C8orf55 | NM_016647 | Homo sapiens chromosome 8 open reading frame 55 (C8orf55), mRNA [NM_016647] | -0,6846 | 4,68E-05 | 9,25E-04 |
| PFKM | NM_000289 | Homo sapiens phosphofructokinase, muscle (PFKM), mRNA [NM_000289] | -1,0008 | 4,73E-05 | 9,34E-04 |
| CHAC2 | NM_001008708 | Homo sapiens ChaC, cation transport regulator homolog 2 (E. coli) (CHAC2), mRNA [NM_001008708] | -0,9665 | 4,85E-05 | 9,54E-04 |
| LRRC20 | NM_018205 | Homo sapiens leucine rich repeat containing 20 (LRRC20), transcript variant 3, mRNA [NM_018205] | -0,8468 | 4,87E-05 | 9,56E-04 |
| POLR2I | NM_006233 | Homo sapiens polymerase (RNA) II (DNA directed) polypeptide I, 14.5kDa (POLR2I), mRNA [NM_006233] | -0,6857 | 4,91E-05 | 9,64E-04 |
| CDC42BPA | NM_003607 | Homo sapiens CDC42 binding protein kinase alpha (DMPK-like) (CDC42BPA), transcript variant B, mRNA [NM_003607] | -0,5035 | 4,93E-05 | 9,65E-04 |
| BG259864 | BG259864 | BG259864 602371819F1 NIH_MGC_93 Homo sapiens cDNA clone IMAGE:4479749 5', mRNA sequence [BG259864] | -1,3001 | 4,94E-05 | 9,68E-04 |
| A_24_P204574 | A_24_P204574 | Unknown | -1,4527 | 4,95E-05 | 9,68E-04 |
| SUV39H1 | NM_003173 | Homo sapiens suppressor of variegation 3-9 homolog 1 (Drosophila) (SUV39H1), mRNA [NM_003173] | -0,6097 | 4,97E-05 | 9,71E-04 |
| RPN2 | NM_002951 | Homo sapiens ribophorin II (RPN2), mRNA [NM_002951] | -0,7441 | 5,03E-05 | 9,81E-04 |
| THC2707284 | THC2707284 | Q214U3_RHOPA (Q214U3) Penicillin-binding protein 1C precursor, partial (3%) [THC2707284] | -1,0307 | 5,06E-05 | 9,85E-04 |
| PET112L | NM_004564 | Homo sapiens PET112-like (yeast) (PET112L), mRNA [NM_004564] | -0,6545 | 5,08E-05 | 9,87E-04 |
| SIGLECP3 | NR_002804 | Homo sapiens sialic acid binding Ig-like lectin, pseudogene 3 (SIGLECP3) on chromosome 19 [NR_002804] | -0,5832 | 5,10E-05 | 9,89E-04 |
| TMEM64 | AK095472 | Homo sapiens cDNA FLJ38153 fis, clone DFNES1000083. [AK095472] | -0,6106 | 5,10E-05 | 9,89E-04 |
| APRIN | NM_015032 | Homo sapiens androgen-induced proliferation inhibitor (APRIN), mRNA [NM_015032] | -0,6019 | 5,26E-05 | 1,02E-03 |
| ZNF543 | NM_213598 | Homo sapiens zinc finger protein 543 (ZNF543), mRNA [NM_213598] | -0,4853 | 5,27E-05 | 1,02E-03 |
| KIAA1191 | NM_020444 | Homo sapiens KIAA1191 (KIAA1191), transcript variant 1, mRNA [NM_020444] | -1,0629 | 5,29E-05 | 1,02E-03 |
| ENST00000377233 | ENST00000377233 | Human clone 120Pa immunoglobulin light chain variable region (VkJ) mRNA, partial cds. [U21012] | -0,9823 | 5,47E-05 | 1,05E-03 |
| SYTL4 | NM_080737 | Homo sapiens synaptotagmin-like 4 (granuphilin-a) (SYTL4), mRNA [NM_080737] | -0,5483 | 5,47E-05 | 1,05E-03 |
| YIF1A | NM_020470 | Homo sapiens Yip1 interacting factor homolog A (S. cerevisiae) (YIF1A), mRNA [NM_020470] | -0,7882 | 5,47E-05 | 1,05E-03 |
| YOD1 | NM_018566 | Homo sapiens YOD1 OTU deubiquinating enzyme 1 homolog (S. cerevisiae) (YOD1), mRNA [NM_018566] | -1,4643 | 5,47E-05 | 1,05E-03 |
| FAM13A1 | NM_014883 | Homo sapiens family with sequence similarity 13, member A1 (FAM13A1), transcript variant 1, mRNA [NM_014883] | -0,6207 | 5,61E-05 | 1,07E-03 |
| CLDN5 | NM_003277 | Homo sapiens claudin 5 (transmembrane protein deleted in velocardiofacial syndrome) (CLDN5), mRNA [NM_003277] | -1,2508 | 5,67E-05 | 1,07E-03 |
| RAPGEF2 | AB002311 | Homo sapiens mRNA for KIAA0313 gene, partial cds. [AB002311] | -1,1206 | 5,68E-05 | 1,07E-03 |
| DPP3 | NM_130443 | Homo sapiens dipeptidyl-peptidase 3 (DPP3), transcript variant 2, mRNA [NM_130443] | -0,6428 | 5,77E-05 | 1,09E-03 |
| AY003763 | AY003763 | Homo sapiens isolate sy-3A/17-G9 immunoglobulin alpha heavy chain variable region mRNA, partial cds. [AY003763] | -1,5012 | 5,86E-05 | 1,10E-03 |
| FXN | NM_181425 | Homo sapiens frataxin (FXN), nuclear gene encoding mitochondrial protein, transcript variant 2, mRNA [NM_181425] | -0,5836 | 5,93E-05 | 1,11E-03 |
| LRRC8A | NM_019594 | Homo sapiens leucine rich repeat containing 8 family, member A (LRRC8A), mRNA [NM_019594] | -0,9864 | 5,96E-05 | 1,12E-03 |
| DAB2 | NM_001343 | Homo sapiens disabled homolog 2, mitogen-responsive phosphoprotein (Drosophila) (DAB2), mRNA [NM_001343] | -0,5991 | 6,00E-05 | 1,13E-03 |
| PRPF8 | NM_006445 | Homo sapiens PRP8 pre-mRNA processing factor 8 homolog (S. cerevisiae) (PRPF8), mRNA [NM_006445] | -0,7013 | 6,06E-05 | 1,14E-03 |
| RRM2 | NM_001034 | Homo sapiens ribonucleotide reductase M2 polypeptide (RRM2), mRNA [NM_001034] | -0,8960 | 6,08E-05 | 1,14E-03 |
| MFSD5 | NM_032889 | Homo sapiens major facilitator superfamily domain containing 5 (MFSD5), mRNA [NM_032889] | -1,2017 | 6,09E-05 | 1,14E-03 |
| ENST00000377221 | ENST00000377221 | Unknown | -1,4574 | 6,10E-05 | 1,14E-03 |
| ZNF696 | NM_030895 | Homo sapiens zinc finger protein 696 (ZNF696), mRNA [NM_030895] | -0,5418 | 6,12E-05 | 1,14E-03 |
| TRIM59 | NM_173084 | Homo sapiens tripartite motif-containing 59 (TRIM59), mRNA [NM_173084] | -1,0099 | 6,16E-05 | 1,15E-03 |
| FUNDC2 | NM_023934 | Homo sapiens FUN14 domain containing 2 (FUNDC2), mRNA [NM_023934] | -0,7862 | 6,19E-05 | 1,15E-03 |
| RSC1A1 | NM_006511 | Homo sapiens regulatory solute carrier protein, family 1, member 1 (RSC1A1), mRNA [NM_006511] | -0,6470 | 6,19E-05 | 1,15E-03 |
| ELOVL1 | NM_022821 | Homo sapiens elongation of very long chain fatty acids (FEN1/Elo2, SUR4/Elo3, yeast)-like 1 (ELOVL1), mRNA [NM_022821] | -1,0188 | 6,24E-05 | 1,16E-03 |
| HSPC142 | NM_001033549 | Homo sapiens HSPC142 protein (HSPC142), transcript variant 1, mRNA [NM_001033549] | -0,5436 | 6,24E-05 | 1,16E-03 |
| BCL2 | NM_000633 | Homo sapiens B-cell CLL/lymphoma 2 (BCL2), nuclear gene encoding mitochondrial protein, transcript variant alpha, mRNA [NM_000633] | -0,9312 | 6,33E-05 | 1,17E-03 |
| NAG | NM_015909 | Homo sapiens neuroblastoma-amplified protein (NAG), mRNA [NM_015909] | -0,8516 | 6,35E-05 | 1,18E-03 |
| BC014395 | BC014395 | Homo sapiens, clone IMAGE:3029191, mRNA. [BC014395] | -0,9643 | 6,46E-05 | 1,19E-03 |
| JAKMIP1 | NM_144720 | Homo sapiens janus kinase and microtubule interacting protein 1 (JAKMIP1), mRNA [NM_144720] | -0,7970 | 6,60E-05 | 1,22E-03 |
| PRMT6 | NM_018137 | Homo sapiens protein arginine methyltransferase 6 (PRMT6), mRNA [NM_018137] | -0,9863 | 6,62E-05 | 1,22E-03 |
| ENST00000377226 | ENST00000377226 | Homo sapiens isolate D3-P-4-K-39 immunoglobulin light chain variable region mRNA, partial cds. [DQ841033] | -1,5711 | 6,71E-05 | 1,23E-03 |
| SPECC1 | NM_152904 | Homo sapiens sperm antigen with calponin homology and coiled-coil domains 1 (SPECC1), transcript variant NSP5beta3alpha, mRNA [NM_152904] | -0,9869 | 6,71E-05 | 1,23E-03 |
| RBX1 | NM_014248 | Homo sapiens ring-box 1 (RBX1), mRNA [NM_014248] | -0,9553 | 6,78E-05 | 1,24E-03 |
| APEX2 | NM_014481 | Homo sapiens APEX nuclease (apurinic/apyrimidinic endonuclease) 2 (APEX2), nuclear gene encoding mitochondrial protein, mRNA [NM_014481] | -0,9606 | 6,94E-05 | 1,27E-03 |
| FLJ20489 | BC026344 | Homo sapiens cDNA clone MGC:26667 IMAGE:4798578, complete cds. [BC026344] | -1,1288 | 6,96E-05 | 1,27E-03 |
| TRAF3IP1 | BC059174 | Homo sapiens TNF receptor-associated factor 3 interacting protein 1, mRNA (cDNA clone MGC:54069 IMAGE:6191726), complete cds. [BC059174] | -0,6332 | 6,97E-05 | 1,27E-03 |
| ATP1B1 | NM_001677 | Homo sapiens ATPase, Na+/K+ transporting, beta 1 polypeptide (ATP1B1), transcript variant 1, mRNA [NM_001677] | -0,8010 | 6,97E-05 | 1,27E-03 |
| SLC25A21 | NM_030631 | Homo sapiens solute carrier family 25 (mitochondrial oxodicarboxylate carrier), member 21 (SLC25A21), mRNA [NM_030631] | -0,6759 | 6,98E-05 | 1,27E-03 |
| HEBP1 | NM_015987 | Homo sapiens heme binding protein 1 (HEBP1), mRNA [NM_015987] | -1,2682 | 7,13E-05 | 1,29E-03 |
| PSKH1 | NM_006742 | Homo sapiens protein serine kinase H1 (PSKH1), mRNA [NM_006742] | -0,7762 | 7,16E-05 | 1,30E-03 |
| RANBP5 | NM_002271 | Homo sapiens RAN binding protein 5 (RANBP5), mRNA [NM_002271] | -0,6657 | 7,21E-05 | 1,30E-03 |
| PAFAH1B1 | NM_000430 | Homo sapiens platelet-activating factor acetylhydrolase, isoform Ib, alpha subunit 45kDa (PAFAH1B1), mRNA [NM_000430] | -1,2071 | 7,38E-05 | 1,33E-03 |
| EDG1 | NM_001400 | Homo sapiens endothelial differentiation, sphingolipid G-protein-coupled receptor, 1 (EDG1), mRNA [NM_001400] | -1,1021 | 7,41E-05 | 1,34E-03 |
| A_24_P552987 | A_24_P552987 | Unknown | -0,7651 | 7,51E-05 | 1,35E-03 |
| AF086139 | AF086139 | Homo sapiens full length insert cDNA clone ZA91F08. [AF086139] | -0,9759 | 7,52E-05 | 1,35E-03 |
| TUBB1 | NM_030773 | Homo sapiens tubulin, beta 1 (TUBB1), mRNA [NM_030773] | -0,7130 | 7,54E-05 | 1,35E-03 |
| ATG4A | NM_178271 | Homo sapiens ATG4 autophagy related 4 homolog A (S. cerevisiae) (ATG4A), transcript variant 3, mRNA [NM_178271] | -0,8574 | 7,58E-05 | 1,36E-03 |
| ACHE | NM_000665 | Homo sapiens acetylcholinesterase (Yt blood group) (ACHE), transcript variant E4-E6, mRNA [NM_000665] | -1,0670 | 7,59E-05 | 1,36E-03 |
| KIAA0406 | NM_014657 | Homo sapiens KIAA0406 (KIAA0406), mRNA [NM_014657] | -0,4607 | 7,79E-05 | 1,39E-03 |
| GRWD1 | NM_031485 | Homo sapiens glutamate-rich WD repeat containing 1 (GRWD1), mRNA [NM_031485] | -0,6756 | 7,88E-05 | 1,41E-03 |
| C10orf61 | NM_015631 | Homo sapiens chromosome 10 open reading frame 61 (C10orf61), transcript variant 2, mRNA [NM_015631] | -0,9236 | 7,97E-05 | 1,42E-03 |
| LGR4 | NM_018490 | Homo sapiens leucine-rich repeat-containing G protein-coupled receptor 4 (LGR4), mRNA [NM_018490] | -0,6027 | 7,97E-05 | 1,42E-03 |
| BC040991 | BC040991 | Homo sapiens cDNA clone IMAGE:4817695. [BC040991] | -0,6936 | 8,23E-05 | 1,45E-03 |
| A_32_P19460 | A_32_P19460 | Unknown | -0,5152 | 8,34E-05 | 1,47E-03 |
| CTSB | NM_147780 | Homo sapiens cathepsin B (CTSB), transcript variant 2, mRNA [NM_147780] | -1,3607 | 8,38E-05 | 1,48E-03 |
| IGLV6-57 | BC023973 | Homo sapiens immunoglobulin lambda variable 6-57, mRNA (cDNA clone MGC:34845 IMAGE:5223747), complete cds. [BC023973] | -1,5345 | 8,41E-05 | 1,48E-03 |
| EGFL8 | NM_030652 | Homo sapiens EGF-like-domain, multiple 8 (EGFL8), mRNA [NM_030652] | -1,0744 | 8,62E-05 | 1,51E-03 |
| PLK1 | NM_005030 | Homo sapiens polo-like kinase 1 (Drosophila) (PLK1), mRNA [NM_005030] | -0,6235 | 8,77E-05 | 1,53E-03 |
| CUL4A | NM_001008895 | Homo sapiens cullin 4A (CUL4A), transcript variant 1, mRNA [NM_001008895] | -0,8327 | 8,78E-05 | 1,53E-03 |
| AF289562 | AF289562 | Homo sapiens clone pp6337 unknown mRNA. [AF289562] | -1,5084 | 9,08E-05 | 1,58E-03 |
| C11orf77 | NM_173811 | Homo sapiens chromosome 11 open reading frame 77 (C11orf77), mRNA [NM_173811] | -0,5722 | 9,14E-05 | 1,59E-03 |
| KIAA0133 | NM_014777 | Homo sapiens KIAA0133 (KIAA0133), mRNA [NM_014777] | -0,5661 | 9,18E-05 | 1,59E-03 |
| WDR32 | NM_024345 | Homo sapiens WD repeat domain 32 (WDR32), mRNA [NM_024345] | -2,4503 | 9,22E-05 | 1,60E-03 |
| GEMIN4 | NM_015721 | Homo sapiens gem (nuclear organelle) associated protein 4 (GEMIN4), mRNA [NM_015721] | -0,9086 | 9,24E-05 | 1,60E-03 |
| THC2685373 | THC2685373 | Unknown | -0,5714 | 9,31E-05 | 1,61E-03 |
| ALDH5A1 | NM_170740 | Homo sapiens aldehyde dehydrogenase 5 family, member A1 (succinate-semialdehyde dehydrogenase) (ALDH5A1), nuclear gene encoding mitochondrial protein, transcript variant 1, mRNA [NM_170740] | -0,7988 | 9,44E-05 | 1,63E-03 |
| SPSB1 | NM_025106 | Homo sapiens splA/ryanodine receptor domain and SOCS box containing 1 (SPSB1), mRNA [NM_025106] | -0,8344 | 9,56E-05 | 1,65E-03 |
| FBXO18 | NM_178150 | Homo sapiens F-box protein, helicase, 18 (FBXO18), transcript variant 2, mRNA [NM_178150] | -0,5965 | 9,62E-05 | 1,66E-03 |
| UBE2V1 | NM_001032288 | Homo sapiens ubiquitin-conjugating enzyme E2 variant 1 (UBE2V1), transcript variant 4, mRNA [NM_001032288] | -0,7546 | 9,63E-05 | 1,66E-03 |
| CENPP | NM_001012267 | Homo sapiens centromere protein P (CENPP), mRNA [NM_001012267] | -0,8739 | 9,67E-05 | 1,66E-03 |
| THC2636523 | THC2636523 | Unknown | -0,4936 | 9,67E-05 | 1,66E-03 |
| CLN6 | NM_017882 | Homo sapiens ceroid-lipofuscinosis, neuronal 6, late infantile, variant (CLN6), mRNA [NM_017882] | -0,6452 | 9,78E-05 | 1,68E-03 |
| RPS3 | NM_001005 | Homo sapiens ribosomal protein S3 (RPS3), mRNA [NM_001005] | -0,6691 | 9,99E-05 | 1,71E-03 |
| DKFZP761M1511 | AK096661 | Homo sapiens cDNA FLJ39342 fis, clone OCBBF2018873. [AK096661] | -1,0765 | 1,03E-04 | 1,76E-03 |
| ZCD2 | NM_001008388 | Homo sapiens zinc finger, CDGSH-type domain 2 (ZCD2), mRNA [NM_001008388] | -1,2174 | 1,04E-04 | 1,77E-03 |
| CYBASC3 | NM_153611 | Homo sapiens cytochrome b, ascorbate dependent 3 (CYBASC3), mRNA [NM_153611] | -0,7687 | 1,06E-04 | 1,80E-03 |
| NGRN | NM_001033088 | Homo sapiens neugrin, neurite outgrowth associated (NGRN), transcript variant 2, mRNA [NM_001033088] | -0,7690 | 1,07E-04 | 1,81E-03 |
| LANCL2 | NM_018697 | Homo sapiens LanC lantibiotic synthetase component C-like 2 (bacterial) (LANCL2), mRNA [NM_018697] | -0,6379 | 1,07E-04 | 1,81E-03 |
| VPS41 | BX648347 | Homo sapiens mRNA; cDNA DKFZp686I08170 (from clone DKFZp686I08170). [BX648347] | -0,6382 | 1,08E-04 | 1,83E-03 |
| SH3GLB2 | NM_020145 | Homo sapiens SH3-domain GRB2-like endophilin B2 (SH3GLB2), mRNA [NM_020145] | -0,4707 | 1,11E-04 | 1,86E-03 |
| NF2 | NM_181832 | Homo sapiens neurofibromin 2 (bilateral acoustic neuroma) (NF2), transcript variant 8, mRNA [NM_181832] | -1,0495 | 1,11E-04 | 1,86E-03 |
| C6orf89 | NM_152734 | Homo sapiens chromosome 6 open reading frame 89 (C6orf89), mRNA [NM_152734] | -0,9591 | 1,11E-04 | 1,87E-03 |
| PECI | NM_206836 | Homo sapiens peroxisomal D3,D2-enoyl-CoA isomerase (PECI), transcript variant 2, mRNA [NM_206836] | -0,8849 | 1,12E-04 | 1,87E-03 |
| PRRT3 | NM_207351 | Homo sapiens proline-rich transmembrane protein 3 (PRRT3), mRNA [NM_207351] | -0,5943 | 1,12E-04 | 1,88E-03 |
| TMEM56 | NM_152487 | Homo sapiens transmembrane protein 56 (TMEM56), mRNA [NM_152487] | -0,7598 | 1,13E-04 | 1,88E-03 |
| BC087732 | BC087732 | Homo sapiens cDNA clone IMAGE:6253289, **** WARNING: chimeric clone ****. [BC087732] | -0,7078 | 1,13E-04 | 1,88E-03 |
| YIPF6 | NM_173834 | Homo sapiens Yip1 domain family, member 6 (YIPF6), mRNA [NM_173834] | -1,0081 | 1,13E-04 | 1,88E-03 |
| ABCF1 | NM_001025091 | Homo sapiens ATP-binding cassette, sub-family F (GCN20), member 1 (ABCF1), transcript variant 1, mRNA [NM_001025091] | -0,7218 | 1,13E-04 | 1,89E-03 |
| LOC283666 | BC048264 | Homo sapiens hypothetical protein LOC283666, mRNA (cDNA clone IMAGE:4415549), partial cds. [BC048264] | -1,0889 | 1,14E-04 | 1,89E-03 |
| VTI1B | NM_006370 | Homo sapiens vesicle transport through interaction with t-SNAREs homolog 1B (yeast) (VTI1B), mRNA [NM_006370] | -0,7354 | 1,18E-04 | 1,96E-03 |
| HBLD2 | NM_030940 | Homo sapiens HESB like domain containing 2 (HBLD2), mRNA [NM_030940] | -1,0021 | 1,20E-04 | 1,98E-03 |
| LTBP1 | NM_206943 | Homo sapiens latent transforming growth factor beta binding protein 1 (LTBP1), transcript variant 1, mRNA [NM_206943] | -0,7483 | 1,20E-04 | 1,98E-03 |
| AK090416 | AK090416 | Homo sapiens mRNA for FLJ00318 protein. [AK090416] | -0,5515 | 1,21E-04 | 1,99E-03 |
| MRPL37 | NM_016491 | Homo sapiens mitochondrial ribosomal protein L37 (MRPL37), nuclear gene encoding mitochondrial protein, mRNA [NM_016491] | -0,6743 | 1,21E-04 | 1,99E-03 |
| FTSJ2 | NM_013393 | Homo sapiens FtsJ homolog 2 (E. coli) (FTSJ2), mRNA [NM_013393] | -0,6915 | 1,22E-04 | 2,01E-03 |
| ACAD9 | NM_014049 | Homo sapiens acyl-Coenzyme A dehydrogenase family, member 9 (ACAD9), mRNA [NM_014049] | -0,4606 | 1,23E-04 | 2,03E-03 |
| CCDC117 | NM_173510 | Homo sapiens coiled-coil domain containing 117 (CCDC117), mRNA [NM_173510] | -0,5807 | 1,26E-04 | 2,06E-03 |
| WIPI2 | NM_015610 | Homo sapiens WD repeat domain, phosphoinositide interacting 2 (WIPI2), transcript variant 1, mRNA [NM_015610] | -0,7366 | 1,27E-04 | 2,07E-03 |
| DKFZp779O175 | ENST00000330692 | CDNA FLJ27459 fis, clone TST05904. (Fragment). [Source:Uniprot/SPTREMBL;Acc:Q6ZNN0] [ENST00000330692] | -0,5997 | 1,28E-04 | 2,08E-03 |
| INTS5 | NM_030628 | Homo sapiens integrator complex subunit 5 (INTS5), mRNA [NM_030628] | -0,4695 | 1,30E-04 | 2,12E-03 |
| C20orf55 | NM_001042353 | Homo sapiens chromosome 20 open reading frame 55 (C20orf55), transcript variant 3, mRNA [NM_001042353] | -0,7181 | 1,31E-04 | 2,12E-03 |
| HCCS | NM_005333 | Homo sapiens holocytochrome c synthase (cytochrome c heme-lyase) (HCCS), mRNA [NM_005333] | -0,8400 | 1,31E-04 | 2,13E-03 |
| AF063695 | AF063695 | Homo sapiens clone BCPBLL11 immunoglobulin lambda light chain variable region mRNA, partial cds. [AF063695] | -1,0750 | 1,32E-04 | 2,14E-03 |
| RUVBL2 | NM_006666 | Homo sapiens RuvB-like 2 (E. coli) (RUVBL2), mRNA [NM_006666] | -0,8112 | 1,34E-04 | 2,17E-03 |
| BF965065 | BF965065 | 602268829F1 NIH_MGC_81 Homo sapiens cDNA clone IMAGE:4356966 5', mRNA sequence [BF965065] | -0,6272 | 1,35E-04 | 2,18E-03 |
| BM455859 | BM455859 | AGENCOURT_6409185 NIH_MGC_85 Homo sapiens cDNA clone IMAGE:5498310 5', mRNA sequence [BM455859] | -0,4865 | 1,35E-04 | 2,18E-03 |
| ZHX1 | NM_001017926 | Homo sapiens zinc fingers and homeoboxes 1 (ZHX1), transcript variant 1, mRNA [NM_001017926] | -0,7194 | 1,35E-04 | 2,18E-03 |
| CCDC47 | NM_020198 | Homo sapiens coiled-coil domain containing 47 (CCDC47), mRNA [NM_020198] | -1,0207 | 1,36E-04 | 2,20E-03 |
| HSPBP1 | NM_012267 | Homo sapiens hsp70-interacting protein (HSPBP1), mRNA [NM_012267] | -0,5900 | 1,36E-04 | 2,20E-03 |
| CYBRD1 | NM_024843 | Homo sapiens cytochrome b reductase 1 (CYBRD1), mRNA [NM_024843] | -0,7790 | 1,38E-04 | 2,22E-03 |
| STOML2 | NM_013442 | Homo sapiens stomatin (EPB72)-like 2 (STOML2), mRNA [NM_013442] | -0,6042 | 1,41E-04 | 2,26E-03 |
| TMEM14B | NM_030969 | Homo sapiens transmembrane protein 14B (TMEM14B), mRNA [NM_030969] | -1,0037 | 1,42E-04 | 2,28E-03 |
| ENST00000379879 | ENST00000379879 | Immunglobulin heavy chain variable region (Fragment). [Source:Uniprot/SPTREMBL;Acc:Q0ZCG6] [ENST00000379879] | -1,0347 | 1,43E-04 | 2,28E-03 |
| RABGAP1L | NM_014857 | Homo sapiens RAB GTPase activating protein 1-like (RABGAP1L), transcript variant 1, mRNA [NM_014857] | -0,4708 | 1,44E-04 | 2,30E-03 |
| ARPC1A | NM_006409 | Homo sapiens actin related protein 2/3 complex, subunit 1A, 41kDa (ARPC1A), mRNA [NM_006409] | -0,6946 | 1,46E-04 | 2,33E-03 |
| GOSR2 | NM_004287 | Homo sapiens golgi SNAP receptor complex member 2 (GOSR2), transcript variant A, mRNA [NM_004287] | -0,4726 | 1,46E-04 | 2,34E-03 |
| PDK2 | NM_002611 | Homo sapiens pyruvate dehydrogenase kinase, isozyme 2 (PDK2), mRNA [NM_002611] | -0,5585 | 1,47E-04 | 2,35E-03 |
| CYB5A | NM_001914 | Homo sapiens cytochrome b5 type A (microsomal) (CYB5A), transcript variant 2, mRNA [NM_001914] | -0,8775 | 1,49E-04 | 2,37E-03 |
| MXRA8 | NM_032348 | Homo sapiens matrix-remodelling associated 8 (MXRA8), mRNA [NM_032348] | -0,8291 | 1,52E-04 | 2,41E-03 |
| FBXO21 | NM_033624 | Homo sapiens F-box protein 21 (FBXO21), transcript variant 1, mRNA [NM_033624] | -0,7731 | 1,53E-04 | 2,42E-03 |
| COASY | NM_025233 | Homo sapiens Coenzyme A synthase (COASY), transcript variant 1, mRNA [NM_025233] | -0,8633 | 1,53E-04 | 2,42E-03 |
| LDOC1L | NM_032287 | Homo sapiens leucine zipper, down-regulated in cancer 1-like (LDOC1L), mRNA [NM_032287] | -0,8045 | 1,55E-04 | 2,45E-03 |
| LOC391559 | XR_018345 | PREDICTED: Homo sapiens similar to vesicle transport through interaction with t-SNAREs 1B (LOC391559), mRNA [XR_018345] | -0,6173 | 1,59E-04 | 2,50E-03 |
| TOP1P2 | NR_001283 | Homo sapiens topoisomerase (DNA) I pseudogene 2 (TOP1P2) on chromosome 22 [NR_001283] | -0,8490 | 1,60E-04 | 2,52E-03 |
| KIF11 | NM_004523 | Homo sapiens kinesin family member 11 (KIF11), mRNA [NM_004523] | -0,7819 | 1,62E-04 | 2,54E-03 |
| AK127768 | AK127768 | Homo sapiens cDNA FLJ45869 fis, clone OCBBF3004908. [AK127768] | -2,0857 | 1,65E-04 | 2,58E-03 |
| RHD | NM_016124 | Homo sapiens Rh blood group, D antigen (RHD), mRNA [NM_016124] | -1,8435 | 1,65E-04 | 2,58E-03 |
| AP2M1 | NM_004068 | Homo sapiens adaptor-related protein complex 2, mu 1 subunit (AP2M1), transcript variant 1, mRNA [NM_004068] | -0,9093 | 1,66E-04 | 2,59E-03 |
| W60781 | W60781 | W60781 zd26f05.r1 Soares_fetal_heart_NbHH19W Homo sapiens cDNA clone IMAGE:341793 5' similar to gb:J02874 FATTY ACID-BINDING PROTEIN, ADIPOCYTE (HUMAN);, mRNA sequence [W60781] | -1,8338 | 1,67E-04 | 2,61E-03 |
| SMC3 | NM_005445 | Homo sapiens structural maintenance of chromosomes 3 (SMC3), mRNA [NM_005445] | -0,6843 | 1,68E-04 | 2,61E-03 |
| DNAJC7 | NM_003315 | Homo sapiens DnaJ (Hsp40) homolog, subfamily C, member 7 (DNAJC7), mRNA [NM_003315] | -0,8509 | 1,68E-04 | 2,61E-03 |
| TUSC1 | NM_001004125 | Homo sapiens tumor suppressor candidate 1 (TUSC1), mRNA [NM_001004125] | -0,5386 | 1,68E-04 | 2,62E-03 |
| HIP2 | NM_005339 | Homo sapiens huntingtin interacting protein 2 (HIP2), mRNA [NM_005339] | -0,6049 | 1,69E-04 | 2,63E-03 |
| KIAA0355 | NM_014686 | Homo sapiens KIAA0355 (KIAA0355), mRNA [NM_014686] | -0,7800 | 1,70E-04 | 2,64E-03 |
| C19orf48 | NM_199249 | Homo sapiens chromosome 19 open reading frame 48 (C19orf48), mRNA [NM_199249] | -0,5581 | 1,71E-04 | 2,65E-03 |
| CR617560 | CR617560 | full-length cDNA clone CS0DC013YG14 of Neuroblastoma Cot 25-normalized of Homo sapiens (human). [CR617560] | -0,5218 | 1,72E-04 | 2,66E-03 |
| COL6A1 | NM_001848 | Homo sapiens collagen, type VI, alpha 1 (COL6A1), mRNA [NM_001848] | -0,7592 | 1,72E-04 | 2,66E-03 |
| FAM129B | AF151783 | Homo sapiens MEG3 (MEG3) mRNA, complete cds. [AF151783] | -0,8340 | 1,73E-04 | 2,66E-03 |
| DNAJC9 | NM_015190 | Homo sapiens DnaJ (Hsp40) homolog, subfamily C, member 9 (DNAJC9), mRNA [NM_015190] | -1,1924 | 1,74E-04 | 2,67E-03 |
| NUP93 | NM_014669 | Homo sapiens nucleoporin 93kDa (NUP93), mRNA [NM_014669] | -0,7532 | 1,74E-04 | 2,67E-03 |
| SEPHS1 | NM_012247 | Homo sapiens selenophosphate synthetase 1 (SEPHS1), mRNA [NM_012247] | -0,5281 | 1,74E-04 | 2,68E-03 |
| C14orf1 | NM_007176 | Homo sapiens chromosome 14 open reading frame 1 (C14orf1), mRNA [NM_007176] | -0,5330 | 1,75E-04 | 2,69E-03 |
| MAN2A1 | NM_002372 | Homo sapiens mannosidase, alpha, class 2A, member 1 (MAN2A1), mRNA [NM_002372] | -0,5411 | 1,76E-04 | 2,70E-03 |
| TTC4 | NM_004623 | Homo sapiens tetratricopeptide repeat domain 4 (TTC4), mRNA [NM_004623] | -0,4917 | 1,77E-04 | 2,72E-03 |
| KIAA1542 | NM_020901 | Homo sapiens CTD-binding SR-like protein rA9 (KIAA1542), mRNA [NM_020901] | -0,9734 | 1,78E-04 | 2,72E-03 |
| LOC731076 | XR_015691 | PREDICTED: Homo sapiens hypothetical protein LOC731076 (LOC731076), mRNA [XR_015691] | -0,7891 | 1,79E-04 | 2,73E-03 |
| ELAC2 | NM_018127 | Homo sapiens elaC homolog 2 (E. coli) (ELAC2), mRNA [NM_018127] | -0,7920 | 1,81E-04 | 2,76E-03 |
| GOLGA4 | NM_002078 | Homo sapiens golgi autoantigen, golgin subfamily a, 4 (GOLGA4), mRNA [NM_002078] | -0,7397 | 1,83E-04 | 2,78E-03 |
| SLC25A11 | NM_003562 | Homo sapiens solute carrier family 25 (mitochondrial carrier; oxoglutarate carrier), member 11 (SLC25A11), mRNA [NM_003562] | -0,5731 | 1,83E-04 | 2,78E-03 |
| TIMM23 | NM_006327 | Homo sapiens translocase of inner mitochondrial membrane 23 homolog (yeast) (TIMM23), mRNA [NM_006327] | -1,0133 | 1,88E-04 | 2,85E-03 |
| HMG2L1 | NM_005487 | Homo sapiens high-mobility group protein 2-like 1 (HMG2L1), transcript variant 1, mRNA [NM_005487] | -0,6755 | 1,92E-04 | 2,90E-03 |
| LAGE3 | NM_006014 | Homo sapiens L antigen family, member 3 (LAGE3), mRNA [NM_006014] | -0,7904 | 1,93E-04 | 2,91E-03 |
| ZNF584 | NM_173548 | Homo sapiens zinc finger protein 584 (ZNF584), mRNA [NM_173548] | -0,4940 | 1,93E-04 | 2,92E-03 |
| MTCH2 | NM_014342 | Homo sapiens mitochondrial carrier homolog 2 (C. elegans) (MTCH2), nuclear gene encoding mitochondrial protein, mRNA [NM_014342] | -0,4651 | 1,95E-04 | 2,94E-03 |
| TXNRD2 | NM_006440 | Homo sapiens thioredoxin reductase 2 (TXNRD2), nuclear gene encoding mitochondrial protein, mRNA [NM_006440] | -0,4468 | 1,96E-04 | 2,94E-03 |
| ASXL2 | ENST00000336112 | additional sex combs like 2 [Source:RefSeq_peptide;Acc:NP_060733] [ENST00000336112] | -0,6724 | 1,96E-04 | 2,95E-03 |
| THC2730631 | THC2730631 | ARL9_HUMAN (Q6T311) ADP-ribosylation factor-like protein 9, partial (39%) [THC2730631] | -0,7686 | 1,99E-04 | 2,99E-03 |
| AFF1 | NM_005935 | Homo sapiens AF4/FMR2 family, member 1 (AFF1), mRNA [NM_005935] | -0,5724 | 2,00E-04 | 3,00E-03 |
| RPA1 | NM_002945 | Homo sapiens replication protein A1, 70kDa (RPA1), mRNA [NM_002945] | -0,9083 | 2,02E-04 | 3,02E-03 |
| ARHGAP19 | NM_032900 | Homo sapiens Rho GTPase activating protein 19 (ARHGAP19), mRNA [NM_032900] | -0,6830 | 2,03E-04 | 3,03E-03 |
| DLG7 | NM_014750 | Homo sapiens discs, large homolog 7 (Drosophila) (DLG7), mRNA [NM_014750] | -0,9981 | 2,03E-04 | 3,03E-03 |
| C21orf45 | NM_018944 | Homo sapiens chromosome 21 open reading frame 45 (C21orf45), mRNA [NM_018944] | -0,5777 | 2,04E-04 | 3,04E-03 |
| X57818 | X57818 | Human rearranged immunoglobulin lambda light chain mRNA. [X57818] | -1,2098 | 2,04E-04 | 3,04E-03 |
| CIT | NM_007174 | Homo sapiens citron (rho-interacting, serine/threonine kinase 21) (CIT), mRNA [NM_007174] | -1,0828 | 2,04E-04 | 3,04E-03 |
| PCCB | NM_000532 | Homo sapiens propionyl Coenzyme A carboxylase, beta polypeptide (PCCB), mRNA [NM_000532] | -0,9063 | 2,04E-04 | 3,04E-03 |
| RNH1 | NM_002939 | Homo sapiens ribonuclease/angiogenin inhibitor 1 (RNH1), transcript variant 1, mRNA [NM_002939] | -0,6386 | 2,06E-04 | 3,07E-03 |
| ACY1 | NM_000666 | Homo sapiens aminoacylase 1 (ACY1), mRNA [NM_000666] | -0,6398 | 2,07E-04 | 3,08E-03 |
| KLF13 | NM_015995 | Homo sapiens Kruppel-like factor 13 (KLF13), mRNA [NM_015995] | -1,0139 | 2,08E-04 | 3,09E-03 |
| PSMD12 | ENST00000356126 | 26S proteasome non-ATPase regulatory subunit 12 (26S proteasome regulatory subunit p55). [Source:Uniprot/SWISSPROT;Acc:O00232] [ENST00000356126] | -0,4739 | 2,09E-04 | 3,10E-03 |
| TMEM85 | NM_016454 | Homo sapiens transmembrane protein 85 (TMEM85), mRNA [NM_016454] | -0,6399 | 2,10E-04 | 3,11E-03 |
| CENPF | NM_016343 | Homo sapiens centromere protein F, 350/400ka (mitosin) (CENPF), mRNA [NM_016343] | -0,6312 | 2,12E-04 | 3,13E-03 |
| POLDIP2 | NM_015584 | Homo sapiens polymerase (DNA-directed), delta interacting protein 2 (POLDIP2), mRNA [NM_015584] | -0,7212 | 2,14E-04 | 3,15E-03 |
| TMEM57 | NM_018202 | Homo sapiens transmembrane protein 57 (TMEM57), mRNA [NM_018202] | -0,9295 | 2,13E-04 | 3,15E-03 |
| WBSCR18 | NM_032317 | Homo sapiens Williams Beuren syndrome chromosome region 18 (WBSCR18), mRNA [NM_032317] | -0,5392 | 2,14E-04 | 3,15E-03 |
| ENDOG | NM_004435 | Homo sapiens endonuclease G (ENDOG), nuclear gene encoding mitochondrial protein, mRNA [NM_004435] | -0,6547 | 2,18E-04 | 3,21E-03 |
| CTDSPL | NM_001008392 | Homo sapiens CTD (carboxy-terminal domain, RNA polymerase II, polypeptide A) small phosphatase-like (CTDSPL), transcript variant 1, mRNA [NM_001008392] | -0,4758 | 2,18E-04 | 3,21E-03 |
| C20orf29 | NM_018347 | Homo sapiens chromosome 20 open reading frame 29 (C20orf29), mRNA [NM_018347] | -0,4729 | 2,21E-04 | 3,25E-03 |
| IMP3 | NM_018285 | Homo sapiens IMP3, U3 small nucleolar ribonucleoprotein, homolog (yeast) (IMP3), mRNA [NM_018285] | -0,7593 | 2,23E-04 | 3,27E-03 |
| MAF1 | NM_032272 | Homo sapiens MAF1 homolog (S. cerevisiae) (MAF1), mRNA [NM_032272] | -0,7519 | 2,28E-04 | 3,34E-03 |
| DKFZP686E2158 | NM_001048249 | Homo sapiens hypothetical protein LOC643155 (DKFZP686E2158), mRNA [NM_001048249] | -0,6487 | 2,30E-04 | 3,36E-03 |
| ITGB3 | NM_000212 | Homo sapiens integrin, beta 3 (platelet glycoprotein IIIa, antigen CD61) (ITGB3), mRNA [NM_000212] | -1,0614 | 2,31E-04 | 3,36E-03 |
| RPL4 | NM_000968 | Homo sapiens ribosomal protein L4 (RPL4), mRNA [NM_000968] | -0,5451 | 2,31E-04 | 3,37E-03 |
| A_24_P508946 | A_24_P508946 | Unknown | -0,7906 | 2,35E-04 | 3,42E-03 |
| ENST00000307840 | ENST00000307840 | Homo sapiens partial mRNA for immunoglobulin kappa light chain variable region (IGKV gene), isolate B-CLL 302. [AJ697902] | -1,1923 | 2,35E-04 | 3,42E-03 |
| PPP1R8 | NM_138558 | Homo sapiens protein phosphatase 1, regulatory (inhibitor) subunit 8 (PPP1R8), transcript variant 2, mRNA [NM_138558] | -1,0677 | 2,36E-04 | 3,43E-03 |
| DGCR8 | NM_022720 | Homo sapiens DiGeorge syndrome critical region gene 8 (DGCR8), mRNA [NM_022720] | -0,7173 | 2,36E-04 | 3,43E-03 |
| A_23_P158868 | A_23_P158868 | Unknown | -1,2904 | 2,37E-04 | 3,44E-03 |
| RHBDD1 | BC062636 | Homo sapiens rhomboid domain containing 1, mRNA (cDNA clone IMAGE:5493862), partial cds. [BC062636] | -0,8985 | 2,37E-04 | 3,44E-03 |
| A_24_P186354 | A_24_P186354 | Unknown | -0,6542 | 2,38E-04 | 3,44E-03 |
| TM7SF3 | NM_016551 | Homo sapiens transmembrane 7 superfamily member 3 (TM7SF3), mRNA [NM_016551] | -0,8938 | 2,39E-04 | 3,46E-03 |
| PDAP1 | NM_014891 | Homo sapiens PDGFA associated protein 1 (PDAP1), mRNA [NM_014891] | -1,2413 | 2,41E-04 | 3,48E-03 |
| THC2515746 | THC2515746 | Unknown | -0,5738 | 2,48E-04 | 3,57E-03 |
| KATNAL1 | NM_032116 | Homo sapiens katanin p60 subunit A-like 1 (KATNAL1), transcript variant 1, mRNA [NM_032116] | -0,8619 | 2,49E-04 | 3,58E-03 |
| SMC1A | NM_006306 | Homo sapiens structural maintenance of chromosomes 1A (SMC1A), mRNA [NM_006306] | -0,8063 | 2,50E-04 | 3,58E-03 |
| MRFAP1 | NM_033296 | Homo sapiens Mof4 family associated protein 1 (MRFAP1), mRNA [NM_033296] | -0,8598 | 2,52E-04 | 3,61E-03 |
| C20orf11 | NM_017896 | Homo sapiens chromosome 20 open reading frame 11 (C20orf11), mRNA [NM_017896] | -0,9368 | 2,52E-04 | 3,61E-03 |
| FAM33A | NM_182620 | Homo sapiens family with sequence similarity 33, member A (FAM33A), mRNA [NM_182620] | -0,4949 | 2,53E-04 | 3,62E-03 |
| TCEB3 | ENST00000374536 | Transcription elongation factor B polypeptide 3 (RNA polymerase II transcription factor SIII subunit A1) (SIII p110) (Elongin-A) (EloA) (Elongin 110 kDa subunit). [Source:Uniprot/SWISSPROT;Acc:Q14241] [ENST00000374536] | -0,4885 | 2,53E-04 | 3,62E-03 |
| TFRC | NM_003234 | Homo sapiens transferrin receptor (p90, CD71) (TFRC), mRNA [NM_003234] | -1,3383 | 2,57E-04 | 3,66E-03 |
| CIRH1A | NM_032830 | Homo sapiens cirrhosis, autosomal recessive 1A (cirhin) (CIRH1A), mRNA [NM_032830] | -0,6610 | 2,58E-04 | 3,67E-03 |
| SMCR7L | NM_019008 | Homo sapiens Smith-Magenis syndrome chromosome region, candidate 7-like (SMCR7L), mRNA [NM_019008] | -0,5655 | 2,59E-04 | 3,68E-03 |
| AK055981 | AK055981 | Homo sapiens cDNA FLJ31419 fis, clone NT2NE2000356. [AK055981] | -0,6755 | 2,59E-04 | 3,68E-03 |
| TIGD5 | NM_032862 | Homo sapiens tigger transposable element derived 5 (TIGD5), mRNA [NM_032862] | -0,5200 | 2,61E-04 | 3,70E-03 |
| AP2A1 | NM_014203 | Homo sapiens adaptor-related protein complex 2, alpha 1 subunit (AP2A1), transcript variant 1, mRNA [NM_014203] | -0,7420 | 2,62E-04 | 3,71E-03 |
| PIR | NM_003662 | Homo sapiens pirin (iron-binding nuclear protein) (PIR), transcript variant 1, mRNA [NM_003662] | -0,8633 | 2,64E-04 | 3,74E-03 |
| AK023559 | AK023559 | Homo sapiens cDNA FLJ13497 fis, clone PLACE1004518. [AK023559] | -0,5343 | 2,66E-04 | 3,75E-03 |
| MGC27348 | BC026177 | Homo sapiens ribosomal protein S2 pseudogene, mRNA (cDNA clone MGC:27348 IMAGE:4671259), complete cds. [BC026177] | -0,6798 | 2,69E-04 | 3,78E-03 |
| ALDH18A1 | NM_002860 | Homo sapiens aldehyde dehydrogenase 18 family, member A1 (ALDH18A1), nuclear gene encoding mitochondrial protein, transcript variant 1, mRNA [NM_002860] | -0,7227 | 2,73E-04 | 3,84E-03 |
| ECH1 | NM_001398 | Homo sapiens enoyl Coenzyme A hydratase 1, peroxisomal (ECH1), mRNA [NM_001398] | -0,6154 | 2,77E-04 | 3,90E-03 |
| SPN | NM_001030288 | Homo sapiens sialophorin (leukosialin, CD43) (SPN), transcript variant 1, mRNA [NM_001030288] | -0,8069 | 2,86E-04 | 4,01E-03 |
| RBM13 | NM_032509 | Homo sapiens RNA binding motif protein 13 (RBM13), mRNA [NM_032509] | -0,7119 | 2,87E-04 | 4,02E-03 |
| MSTP9 | NR_002729 | Homo sapiens macrophage stimulating, pseudogene 9 (MSTP9) on chromosome 1 [NR_002729] | -0,8776 | 2,88E-04 | 4,04E-03 |
| GSG2 | AK056691 | Homo sapiens cDNA FLJ32129 fis, clone PEBLM2000213, weakly similar to Mus musculus genes for integrin aM290, hapsin. [AK056691] | -0,8899 | 2,91E-04 | 4,07E-03 |
| TUBB2A | NM_001069 | Homo sapiens tubulin, beta 2A (TUBB2A), mRNA [NM_001069] | -0,9080 | 2,91E-04 | 4,07E-03 |
| AJ519285 | AJ519285 | Homo sapiens partial mRNA for IgM immunoglobulin heavy chain variable region (IGHV gene), clone ANBPM204. [AJ519285] | -0,9904 | 2,92E-04 | 4,08E-03 |
| THC2660636 | THC2660636 | Unknown | -0,5842 | 2,97E-04 | 4,14E-03 |
| RMND5A | AL050139 | Homo sapiens mRNA; cDNA DKFZp586M141 (from clone DKFZp586M141). [AL050139] | -0,8216 | 2,99E-04 | 4,16E-03 |
| AA554330 | AA554330 | nl03d08.s1 NCI_CGAP_Co3 Homo sapiens cDNA clone IMAGE:1029231 3', mRNA sequence [AA554330] | -0,6576 | 3,01E-04 | 4,18E-03 |
| ATRN | NM_139322 | Homo sapiens attractin (ATRN), transcript variant 2, mRNA [NM_139322] | -0,6781 | 3,03E-04 | 4,20E-03 |
| THC2551769 | THC2551769 | AA411302 zv24g06.r1 Soares_NhHMPu_S1 Homo sapiens cDNA clone IMAGE:754618 5', mRNA sequence [AA411302] | -0,8453 | 3,03E-04 | 4,20E-03 |
| THC2664480 | THC2664480 | ALU1_HUMAN (P39188) Alu subfamily J sequence contamination warning entry, partial (6%) [THC2664480] | -0,4626 | 3,04E-04 | 4,22E-03 |
| PF4 | NM_002619 | Homo sapiens platelet factor 4 (chemokine (C-X-C motif) ligand 4) (PF4), mRNA [NM_002619] | -1,9901 | 3,05E-04 | 4,23E-03 |
| E2F2 | NM_004091 | Homo sapiens E2F transcription factor 2 (E2F2), mRNA [NM_004091] | -1,5993 | 3,05E-04 | 4,23E-03 |
| ENST00000358618 | ENST00000358618 | PREDICTED: Homo sapiens similar to ribosomal protein S2 (LOC729842), mRNA [XM_001134158] | -0,8032 | 3,06E-04 | 4,24E-03 |
| HDAC4 | NM_006037 | Homo sapiens histone deacetylase 4 (HDAC4), mRNA [NM_006037] | -0,9151 | 3,06E-04 | 4,24E-03 |
| EPC2 | NM_015630 | Homo sapiens enhancer of polycomb homolog 2 (Drosophila) (EPC2), mRNA [NM_015630] | -0,8767 | 3,07E-04 | 4,24E-03 |
| AK095583 | AK095583 | Homo sapiens cDNA FLJ38264 fis, clone FCBBF3001657. [AK095583] | -0,6332 | 3,08E-04 | 4,26E-03 |
| C17orf76 | NM_207387 | Homo sapiens chromosome 17 open reading frame 76 (C17orf76), mRNA [NM_207387] | -0,8770 | 3,09E-04 | 4,26E-03 |
| RBM15B | NM_013286 | Homo sapiens RNA binding motif protein 15B (RBM15B), mRNA [NM_013286] | -0,7090 | 3,09E-04 | 4,26E-03 |
| VPS37C | NM_017966 | Homo sapiens vacuolar protein sorting 37 homolog C (S. cerevisiae) (VPS37C), mRNA [NM_017966] | -0,7964 | 3,15E-04 | 4,33E-03 |
| GPAA1 | NM_003801 | Homo sapiens glycosylphosphatidylinositol anchor attachment protein 1 homolog (yeast) (GPAA1), mRNA [NM_003801] | -0,6347 | 3,16E-04 | 4,34E-03 |
| CKAP5 | NM_001008938 | Homo sapiens cytoskeleton associated protein 5 (CKAP5), transcript variant 1, mRNA [NM_001008938] | -0,9162 | 3,21E-04 | 4,38E-03 |
| FOXRED2 | NM_024955 | Homo sapiens FAD-dependent oxidoreductase domain containing 2 (FOXRED2), mRNA [NM_024955] | -0,4799 | 3,23E-04 | 4,40E-03 |
| SFT2D3 | NM_032740 | Homo sapiens SFT2 domain containing 3 (SFT2D3), mRNA [NM_032740] | -0,5212 | 3,23E-04 | 4,40E-03 |
| UBL4A | NM_014235 | Homo sapiens ubiquitin-like 4A (UBL4A), mRNA [NM_014235] | -0,9334 | 3,26E-04 | 4,44E-03 |
| C6orf129 | NM_138493 | Homo sapiens chromosome 6 open reading frame 129 (C6orf129), mRNA [NM_138493] | -0,6096 | 3,31E-04 | 4,49E-03 |
| MRPL49 | NM_004927 | Homo sapiens mitochondrial ribosomal protein L49 (MRPL49), nuclear gene encoding mitochondrial protein, mRNA [NM_004927] | -0,6624 | 3,31E-04 | 4,49E-03 |
| DTYMK | NM_012145 | Homo sapiens deoxythymidylate kinase (thymidylate kinase) (DTYMK), mRNA [NM_012145] | -0,6663 | 3,34E-04 | 4,51E-03 |
| HDGF | NM_004494 | Homo sapiens hepatoma-derived growth factor (high-mobility group protein 1-like) (HDGF), mRNA [NM_004494] | -0,7474 | 3,35E-04 | 4,51E-03 |
| TIMD4 | NM_138379 | Homo sapiens T-cell immunoglobulin and mucin domain containing 4 (TIMD4), mRNA [NM_138379] | -1,2067 | 3,39E-04 | 4,56E-03 |
| AF086448 | AF086448 | Homo sapiens full length insert cDNA clone ZD82B02. [AF086448] | -0,9491 | 3,43E-04 | 4,61E-03 |
| KIAA1155 | ENST00000244221 | Homo sapiens cDNA FLJ37016 fis, clone BRACE2010632. [AK094335] | -0,4147 | 3,49E-04 | 4,67E-03 |
| KIAA1815 | NM_024896 | Homo sapiens KIAA1815 (KIAA1815), mRNA [NM_024896] | -0,3880 | 3,51E-04 | 4,70E-03 |
| ZBTB38 | BC072415 | Homo sapiens cDNA clone IMAGE:6168734. [BC072415] | -0,6025 | 3,52E-04 | 4,71E-03 |
| ARL1 | NM_001177 | Homo sapiens ADP-ribosylation factor-like 1 (ARL1), mRNA [NM_001177] | -0,6488 | 3,54E-04 | 4,73E-03 |
| MGC11257 | BC025971 | Homo sapiens hypothetical protein MGC11257, mRNA (cDNA clone IMAGE:4907364), partial cds. [BC025971] | -0,6285 | 3,58E-04 | 4,76E-03 |
| GMEB1 | ENST00000373816 | Glucocorticoid modulatory element-binding protein 1 (GMEB-1) (Parvovirus initiation factor p96) (PIF p96) (DNA-binding protein p96PIF). [Source:Uniprot/SWISSPROT;Acc:Q9Y692] [ENST00000373816] | -0,7542 | 3,59E-04 | 4,77E-03 |
| THC2532155 | THC2532155 | Q8K2W0_MOUSE (Q8K2W0) Procollagen, type IX, alpha 2, partial (3%) [THC2532155] | -0,8972 | 3,64E-04 | 4,83E-03 |
| TMEM39A | NM_018266 | Homo sapiens transmembrane protein 39A (TMEM39A), mRNA [NM_018266] | -0,6147 | 3,67E-04 | 4,86E-03 |
| LEPREL1 | NM_018192 | Homo sapiens leprecan-like 1 (LEPREL1), mRNA [NM_018192] | -0,6928 | 3,69E-04 | 4,88E-03 |
| STCH | NM_006948 | Homo sapiens stress 70 protein chaperone, microsome-associated, 60kDa (STCH), mRNA [NM_006948] | -0,7384 | 3,74E-04 | 4,93E-03 |
| E2F8 | NM_024680 | Homo sapiens E2F transcription factor 8 (E2F8), mRNA [NM_024680] | -0,8151 | 3,77E-04 | 4,97E-03 |
| AK098422 | AK098422 | Homo sapiens cDNA FLJ25556 fis, clone JTH02629. [AK098422] | -0,7291 | 3,77E-04 | 4,97E-03 |
| PSMC3 | NM_002804 | Homo sapiens proteasome (prosome, macropain) 26S subunit, ATPase, 3 (PSMC3), mRNA [NM_002804] | -0,6778 | 3,77E-04 | 4,97E-03 |
| SESN3 | NM_144665 | Homo sapiens sestrin 3 (SESN3), mRNA [NM_144665] | -0,8527 | 3,80E-04 | 4,99E-03 |
| PDIK1L | NM_152835 | Homo sapiens PDLIM1 interacting kinase 1 like (PDIK1L), mRNA [NM_152835] | -0,5895 | 3,82E-04 | 5,01E-03 |
| RAD51C | NM_002876 | Homo sapiens RAD51 homolog C (S. cerevisiae) (RAD51C), transcript variant 2, mRNA [NM_002876] | -0,6565 | 3,82E-04 | 5,01E-03 |
| TFIP11 | NM_001008697 | Homo sapiens tuftelin interacting protein 11 (TFIP11), transcript variant 1, mRNA [NM_001008697] | -0,6231 | 3,89E-04 | 5,09E-03 |
| MRPL46 | NM_022163 | Homo sapiens mitochondrial ribosomal protein L46 (MRPL46), nuclear gene encoding mitochondrial protein, mRNA [NM_022163] | -0,6194 | 3,90E-04 | 5,10E-03 |
| TAF5L | NM_014409 | Homo sapiens TAF5-like RNA polymerase II, p300/CBP-associated factor (PCAF)-associated factor, 65kDa (TAF5L), transcript variant 1, mRNA [NM_014409] | -0,7777 | 3,90E-04 | 5,10E-03 |
| NOLA2 | NM_017838 | Homo sapiens nucleolar protein family A, member 2 (H/ACA small nucleolar RNPs) (NOLA2), transcript variant 1, mRNA [NM_017838] | -0,5364 | 3,92E-04 | 5,12E-03 |
| MIS12 | NM_024039 | Homo sapiens MIS12, MIND kinetochore complex component, homolog (yeast) (MIS12), mRNA [NM_024039] | -0,4931 | 3,93E-04 | 5,13E-03 |
| BOLA3 | NM_212552 | Homo sapiens bolA homolog 3 (E. coli) (BOLA3), transcript variant 1, mRNA [NM_212552] | -0,9999 | 3,96E-04 | 5,16E-03 |
| KHSRP | NM_003685 | Homo sapiens KH-type splicing regulatory protein (FUSE binding protein 2) (KHSRP), mRNA [NM_003685] | -0,8609 | 3,98E-04 | 5,18E-03 |
| ARL4A | NM_005738 | Homo sapiens ADP-ribosylation factor-like 4A (ARL4A), transcript variant 1, mRNA [NM_005738] | -0,6864 | 3,98E-04 | 5,18E-03 |
| C16orf58 | NM_022744 | Homo sapiens chromosome 16 open reading frame 58 (C16orf58), mRNA [NM_022744] | -0,4349 | 3,98E-04 | 5,18E-03 |
| KIF22 | NM_007317 | Homo sapiens kinesin family member 22 (KIF22), mRNA [NM_007317] | -0,4804 | 3,99E-04 | 5,18E-03 |
| TSPYL1 | NM_003309 | Homo sapiens TSPY-like 1 (TSPYL1), mRNA [NM_003309] | -0,8353 | 4,02E-04 | 5,21E-03 |
| HN1L | NM_144570 | Homo sapiens hematological and neurological expressed 1-like (HN1L), mRNA [NM_144570] | -0,9089 | 4,07E-04 | 5,27E-03 |
| A_24_P161853 | A_24_P161853 | Unknown | -1,1030 | 4,08E-04 | 5,27E-03 |
| MED9 | NM_018019 | Homo sapiens mediator of RNA polymerase II transcription, subunit 9 homolog (S. cerevisiae) (MED9), mRNA [NM_018019] | -0,7989 | 4,10E-04 | 5,29E-03 |
| TMEM9B | NM_020644 | Homo sapiens TMEM9 domain family, member B (TMEM9B), mRNA [NM_020644] | -0,8844 | 4,11E-04 | 5,30E-03 |
| GTF3C4 | ENST00000372146 | General transcription factor 3C polypeptide 4 (EC 2.3.1.48) (Transcription factor IIIC subunit delta) (TF3C-delta) (TFIIIC 90 kDa subunit) (TFIIIC 90). [Source:Uniprot/SWISSPROT;Acc:Q9UKN8] [ENST00000372146] | -0,7682 | 4,11E-04 | 5,30E-03 |
| NAT12 | BC048983 | Homo sapiens N-acetyltransferase 12, mRNA (cDNA clone IMAGE:5259876). [BC048983] | -0,4563 | 4,13E-04 | 5,31E-03 |
| PCGF5 | BC007377 | Homo sapiens polycomb group ring finger 5, mRNA (cDNA clone IMAGE:3640258), complete cds. [BC007377] | -0,8635 | 4,15E-04 | 5,33E-03 |
| FOXO3A | NM_001455 | Homo sapiens forkhead box O3A (FOXO3A), transcript variant 1, mRNA [NM_001455] | -1,0372 | 4,32E-04 | 5,54E-03 |
| FAM100A | NM_145253 | Homo sapiens family with sequence similarity 100, member A (FAM100A), mRNA [NM_145253] | -0,6803 | 4,36E-04 | 5,58E-03 |
| LEPROTL1 | NM_015344 | Homo sapiens leptin receptor overlapping transcript-like 1 (LEPROTL1), mRNA [NM_015344] | -0,9458 | 4,37E-04 | 5,58E-03 |
| LOC152663 | XM_087499 | PREDICTED: Homo sapiens similar to 60S ribosomal protein L7a (LOC152663), mRNA [XM_087499] | -0,5270 | 4,40E-04 | 5,62E-03 |
| SLC29A1 | NM_001078177 | Homo sapiens solute carrier family 29 (nucleoside transporters), member 1 (SLC29A1), nuclear gene encoding mitochondrial protein, transcript variant 1, mRNA [NM_001078177] | -1,1394 | 4,40E-04 | 5,62E-03 |
| ENG | NM_000118 | Homo sapiens endoglin (Osler-Rendu-Weber syndrome 1) (ENG), mRNA [NM_000118] | -0,6602 | 4,40E-04 | 5,62E-03 |
| MFAP1 | NM_005926 | Homo sapiens microfibrillar-associated protein 1 (MFAP1), mRNA [NM_005926] | -0,8699 | 4,41E-04 | 5,62E-03 |
| PSMD2 | NM_002808 | Homo sapiens proteasome (prosome, macropain) 26S subunit, non-ATPase, 2 (PSMD2), mRNA [NM_002808] | -0,7679 | 4,43E-04 | 5,64E-03 |
| PSMC4 | NM_006503 | Homo sapiens proteasome (prosome, macropain) 26S subunit, ATPase, 4 (PSMC4), transcript variant 1, mRNA [NM_006503] | -0,3951 | 4,44E-04 | 5,65E-03 |
| MGC12760 | BC006312 | Homo sapiens hypothetical protein MGC12760, mRNA (cDNA clone MGC:12760 IMAGE:4111573), complete cds. [BC006312] | -1,0519 | 4,47E-04 | 5,68E-03 |
| SLC12A7 | NM_006598 | Homo sapiens solute carrier family 12 (potassium/chloride transporters), member 7 (SLC12A7), mRNA [NM_006598] | -1,0694 | 4,48E-04 | 5,68E-03 |
| RAB2 | AL137321 | Homo sapiens mRNA; cDNA DKFZp434P231 (from clone DKFZp434P231). [AL137321] | -0,5929 | 4,52E-04 | 5,72E-03 |
| IQSEC2 | NM_015075 | Homo sapiens IQ motif and Sec7 domain 2 (IQSEC2), mRNA [NM_015075] | -1,9208 | 4,59E-04 | 5,80E-03 |
[truncated: 100,531 more chars]
