# Supplementary material for: Altered erythropoiesis and decreased number of erythrocytes in children with neuroblastoma
Source: Oncotarget. 2017 May 30;8(32):53194–209. doi: 10.18632/oncotarget.18285 (PMC5581103; doi:10.18632/oncotarget.18285)
Supplement: Supplementary file 3 [file oncotarget-08-53194-s003.docx]

**Supplementary Table 2: Functional annotation clustering of genes down-modulated in resident BM cells from NB patients as compared to healthy children**.

| **Annotation cluster 1** | | **Enrichment score: 6.27** | **Count** | **P_value** | **Benjamini** |
| --- | --- | --- | --- | --- | --- |
|  | BIOCARTA | Hemoglobin's Chaperone | 10 | 1.3E-11 | 1.6E-9 |
|  | UP_KEYWORDS | Heme biosynthesis | 9 | 3.4E-11 | 3.0E-9 |
|  | GOTERM_BP_DIRECT | heme biosynthetic process | 10 | 1.4E-10 | 2.5E-7 |
|  | UP_KEYWORDS | Porphyrin biosynthesis | 7 | 1.0E-9 | 7.3E-8 |
|  | GOTERM_BP_DIRECT | protoporphyrinogen IX biosynthetic process | 7 | 5.8E-9 | 5.1E-6 |
|  | GOTERM_BP_DIRECT | porphyrin-containing compound biosynthetic process | 6 | 7.2E-8 | 4.3E-5 |
|  | GOTERM_BP_DIRECT | response to methylmercury | 6 | 4.0E-6 | 1.8E-3 |
|  | KEGG_PATHWAY | Porphyrin and chlorophyll metabolism | 9 | 7.5E-6 | 1.6E-3 |
|  | GOTERM_BP_DIRECT | response to arsenic-containing substance | 4 | 2.1E-3 | 2.7E-1 |
|  | GOTERM_BP_DIRECT | tetrapyrrole biosynthetic process | 3 | 2.5E-3 | 2.8E-1 |
|  | GOTERM_BP_DIRECT | response to iron ion | 4 | 7.6E-3 | 5.5E-1 |
|  | GOTERM_BP_DIRECT | cellular response to arsenic-containing substance | 3 | 2.0E-2 | 7.3E-1 |
|  | |  |  |  |  |
| **Annotation Cluster 2** | | **Enrichment Score: 3.34** | **Count** | **P_Value** | **Benjamini** |
|  | GOTERM_CC_DIRECT | hemoglobin complex | 5 | 6.6E-5 | 1.1E-2 |
|  | UP_KEYWORDS | Oxygen transport | 5 | 7.1E-5 | 2.8E-3 |
|  | INTERPRO | Haemoglobin, alpha | 4 | 7.6E-5 | 3.2E-2 |
|  | UP_SEQ_FEATURE | metal ion-binding site:Iron (heme distal ligand) | 5 | 7.8E-5 | 4.5E-2 |
|  | INTERPRO | Globin-like | 5 | 9.6E-5 | 2.7E-2 |
|  | INTERPRO | Globin | 5 | 9.6E-5 | 2.7E-2 |
|  | INTERPRO | Globin, structural domain | 5 | 1.3E-4 | 2.8E-2 |
|  | GOTERM_MF_DIRECT | oxygen transporter activity | 5 | 1.6E-4 | 5.0E-2 |
|  | UP_SEQ_FEATURE | metal ion-binding site:Iron (heme proximal ligand) | 5 | 1.9E-4 | 7.2E-2 |
|  | GOTERM_BP_DIRECT | oxygen transport | 5 | 2.0E-4 | 4.3E-2 |
|  | UP_KEYWORDS | Iron | 17 | 3.6E-4 | 9.9E-3 |
|  | INTERPRO | Haemoglobin, pi | 3 | 1.2E-3 | 1.5E-1 |
|  | GOTERM_MF_DIRECT | heme binding | 10 | 2.3E-3 | 3.1E-1 |
|  | UP_KEYWORDS | Heme | 8 | 1.1E-2 | 1.3E-1 |
|  | GOTERM_MF_DIRECT | oxygen binding | 5 | 1.7E-2 | 7.0E-1 |
|  | GOTERM_MF_DIRECT | iron ion binding | 7 | 1.0E-1 | 8.6E-1 |
|  | |  |  |  |  |
| **Annotation Cluster 3** | | **Enrichment Score: 3.24** | **Count** | **P_Value** | **Benjamini** |
|  | INTERPRO | Glycophorin | 4 | 3.1E-5 | 2.6E-2 |
|  | OMIM_DISEASE | Malaria, resistance to | 4 | 3.4E-4 | 6.6E-2 |
|  | PIR_SUPERFAMILY | glycophorin | 3 | 1.9E-3 | 7.1E-2 |
|  | UP_KEYWORDS | Sialic acid | 4 | 5.6E-3 | 8.0E-2 |
|  | |  |  |  |  |
| **Annotation Cluster 4** | | **Enrichment Score: 2.1** | **Count** | **P_Value** | **Benjamini** |
|  | UP_KEYWORDS | Ubl conjugation pathway | 26 | 8.5E-4 | 1.9E-2 |
|  | INTERPRO | Zinc finger, RING/FYVE/PHD-type | 18 | 9.8E-3 | 6.5E-1 |
|  | GOTERM_MF_DIRECT | ubiquitin-protein transferase activity | 15 | 1.0E-2 | 6.0E-1 |
|  | UP_KEYWORDS | Ligase | 15 | 1.2E-2 | 1.3E-1 |
|  | GOTERM_MF_DIRECT | ligase activity | 13 | 1.4E-2 | 6.8E-1 |
|  | GOTERM_BP_DIRECT | protein ubiquitination | 15 | 1.7E-2 | 7.3E-1 |
